# Effect of dietary chickpea structures on the small intestine metabolome and microbiome: a randomised crossover trial

**DOI:** 10.1038/s41467-026-77714-7

**Published:** 2026-09-15

**Authors:** Nadia Fernandes, Mingzhu Cai, Frederick J. Warren, Anthony Duncan, Hannah Harris, Jose Ivan Serrano Contreras, Katarzyna Sidorczuk, Andres Bernal, Andres Castillo, Katerina Petropoulou, Dominic Blunt, Natalia Perez-Moral, Isabel Garcia-Perez, Cathrina Edwards, Falk Hildebrand, Elaine Holmes, Julien Wist, Gary Frost

**Affiliations:** 1 Section for Nutrition, Department of Metabolism, Digestion and Reproduction, Imperial College London, London, UK; 2 Kennedy Institute of Rheumatology, University of Oxford, Oxford, UK; 3 Quadram Institute Bioscience, Norwich Research Park, Norwich, UK; 4 Earlham Institute, Norwich Research Park, Norwich, UK; 5 Australian National Phenome Centre and Centre for Computational and Systems Medicine, Health Futures Institute, Murdoch University, Harry Perkins Building, Perth, WA Australia; 6 School of Systems Engineering and Computing, Universidad del Valle, Cali, Colombia; 7 Department of Imaging, Charing Cross Hospital, Imperial NHS Trust, London, UK; 8 School of Biological Sciences, University of East Anglia, Norwich, UK; 9 School of Medicine, The Hong Kong University of Science and Technology, Clear Water Bay, Hong Kong, China; 10 Chemistry Department, Universidad del Valle, Cali, Colombia

**Keywords:** Metabolomics, Complexity

## Abstract

The human gastrointestinal tract (GIT) is central to human physiology, yet our understanding of the metabolite, microbial, and hormonal spatiotemporal dynamics in humans is limited. How these environments respond to complex food structures and homeostatic mechanisms they generate is unknown in humans. In this randomised study, ten participants consumed chickpea-based meals with intact versus broken cellular structures, with sampling of oral, gastric, duodenal and ileal fluids. The primary outcome was the postprandial enteroendocrine hormone responses (GIP, GLP-1 and PYY); secondary outcomes were GIT metabolite profiles (^1^H NMR) and ileal microbiota (shotgun metagenomics). We observed that food structure markedly altered the spatiotemporal trajectories of metabolites and dominant microbe, driving distinct enteroendocrine hormone responses. Broken-cell meals produced early GIT glucose and maltose peaks associated with rapid GIP responses, whereas intact-cell meals elevated duodenal amino acids and ileal sugars, amino acids and formate, enhancing GLP-1 and PYY responses. Microbial profiles exhibited rapid spatial reorganisation, with evidence that oral bacterial strains transmitted to the ileum, where their abundance was associated with local metabolites and PYY responses. These findings identify food structure as a determinant of postprandial gastrointestinal metabolism and enteroendocrine responses and suggest an underappreciated route for oral bacteria to reach the ileum via the food bolus and impact metabolism. ISRCTN registration: ISRCTN18097249.

## Abstract (web summary)

Here, using spatially time-resolved mapping of the human gut, the authors characterize how food structure shapes metabolites, the microbiome and hormones linked to glucose and appetite, and suggest that oral bacteria influence the ileal microbiome.

## Introduction

The gastrointestinal tract (GIT) is an interconnected, spatially resolved organ with unidirectional flow, where different anatomical regions communicate with each other. The primary function of the GIT is the digestion of food and the absorption of nutrients, while maintaining a barrier against harmful molecules and bacteria, with the major food digestion occurring in the upper intestine. Furthermore, the GIT has a major role in fundamental homeostatic processes such as immunity, appetite regulation and glucose homeostasis^1,2^. To fulfil these varied functions, both food and the resident microbiome come together to create a complex and dynamic environment, regulated through an extensive sensing system in the GIT.

Spatially resolved areas of the GIT play specific roles not only in digestion but in the homeostatic signals they create, with over 20 hormones being secreted by endocrine cells in epithelium^3^. For example, gastric inhibitory polypeptide (GIP) is mainly released from enteroendocrine K cells, found mostly in the duodenum, whereas glucagon-like peptide-1 (GLP-1), peptide tyrosine tyrosine (PYY) and oxyntomodulin (OXM) are released by L enteroendocrine cells prevalent in the ileum, colon and rectum^4^. The product of food digestion by either mammalian enzymes or microbial action is a complex array of nutrients and metabolites, which are sensed through enteroendocrine cell receptors as well as transporter networks on the epithelial surface across the GIT^5^. Food structure plays an important role in digestion and nutrient release and thus acts as a critical regulator of enteroendocrine hormones. Disruption of legume cellular structures led to early gastric and duodenal peaks in glucose and maltose alongside higher GIP response, whereas intact cellular structures retained a large amount of undigested nutrients in the duodenum, possibly allowing more nutrients reach the distal region of the small intestine^1^. An integrated understanding of food structure on metabolite dynamics and enteroendocrine signalling across the GIT is required.

Despite its importance, the microbiome and metabolic processes located in the small intestine are far less explored than those in the distal gut, due to the inherent sample collection challenges in the former^2,6^. 16S rRNA barcoding studies using capsule sampling^7^ and aspirates^8^ reveal a small intestinal microbiome dominated by *Streptococcus, Veillonella, Fusobacterium, Prevotella and Haemophilus*, with spatially variable compositions across the ileum, reflecting differences in the metabolic, pH and oxygen environments of these regions^6^. Analysis of duodenal aspirates by shotgun and 16S sequencing has confirmed that the microbiome of the duodenal lumen is dominated by Proteobacteria, Firmicutes and Actinobacteria, and that it is distinct from stool^9,10^. Evidence supports the interconnectivity of microbial communities across the distinct regions of the gastrointestinal tract, with strain-level transmission observed between the oral cavity and faecal microbiome. This suggests that the oral-gut axis is more permeable than previously assumed, allowing microbial translocation along the GIT despite the physiological and immunological barriers imposed by gastric acidity, digestive enzymes, bile salts, mucosal defences and immune surveillance, which collectively maintain compartmentalisation between the oral and intestinal microbiomes^11^. However, the prevalence and temporal dynamics of oral microbial strains in the ileum are unknown, as well as their function.

Since no studies to date have resolved the spatiotemporal metabolic, nor microbial trajectories and consequences on enteroendocrine hormone secretion, we developed a methodology to assess these in high temporal resolution across the oral, gastric, duodenal and ileal tract. This study provides a spatiotemporally resolved map of human digestion, showing how the food matrix modulates metabolic and microbiota dynamics and enteroendocrine signalling in the small intestine.

## Results

### Overall study design and aims

**Fig. 1:**
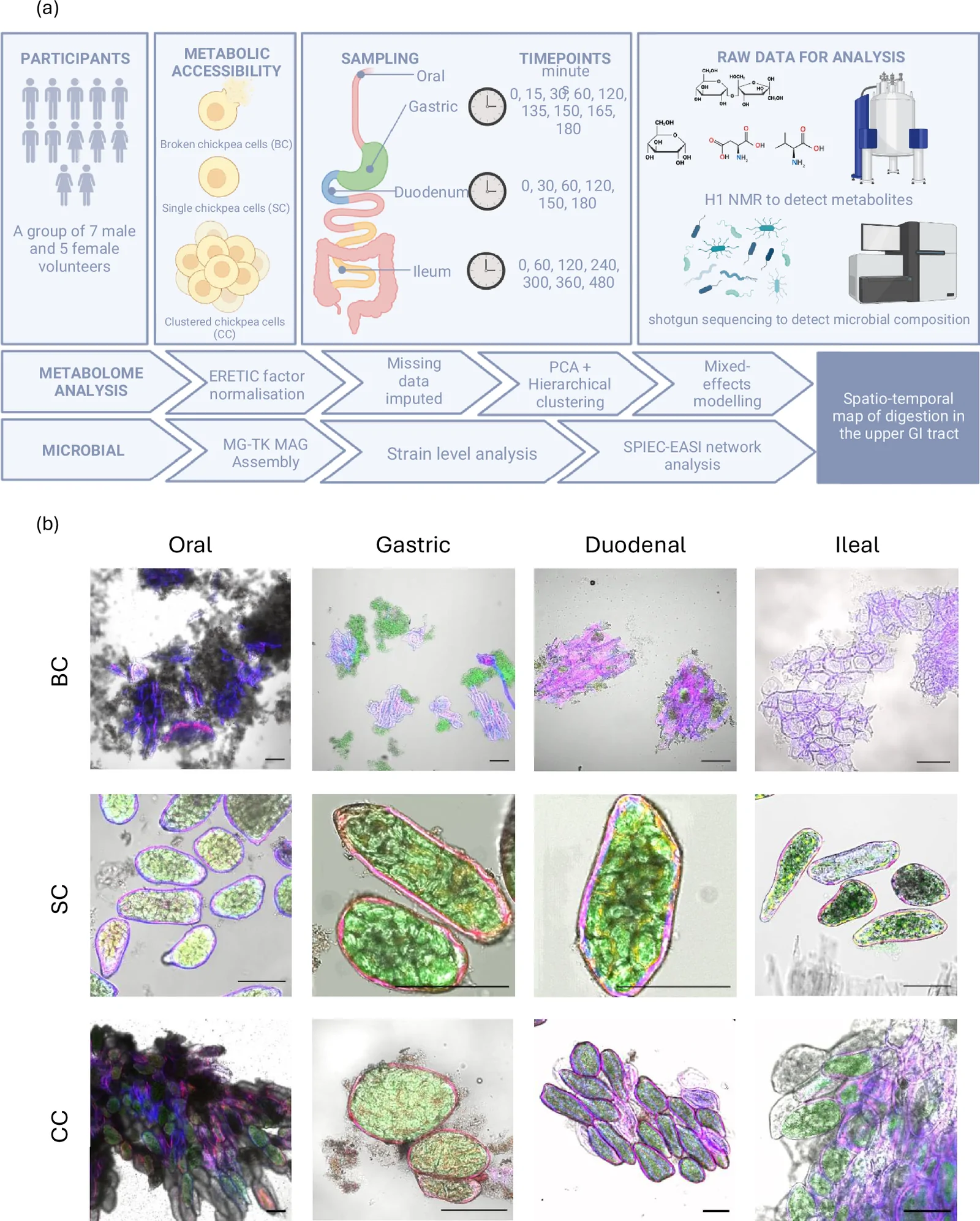
Experimental design. **a** Healthy adults (10 for oral, gastric and duodenal datasets; 8 for the ileal dataset) participated in a crossover study comparing chickpea meals with three food structures: broken cells (BC), single cells (SC) and cell clusters (CC). Each participant received the interventions on separate study visits. Naso-enteral tubes were used to collect gastric, duodenal and ileal contents before and after meal consumption. Samples underwent ^1^H-NMR metabolomics and shotgun metagenomics. Metabolites were quantified from NMR peak areas, and missing non-oral measurements were imputed using missForest. Hierarchical clustering used Euclidean distance and complete linkage. PCA used centred and scaled metabolite concentrations. Metabolite concentrations were analysed using linear mixed-effects models accounting for repeated participant measurements. Microbial reads were assembled and analysed at the strain and network levels. Metabolite and microbial modalities were integrated for spatiotemporal analysis. The study comprised one clinical experiment, it was not independently repeated. Oral metabolites served as pre-gastrointestinal controls. Created in BioRender. F, N. (2025) https://BioRender.com/a42q103. **b** Representative confocal microscopy images of digesta aspirates collected from the mouth, stomach, duodenum and ileum of participants following consumption of a meal containing broken cells (BC), single cells (SC) and cell clusters (CC). Staining highlights cell wall components (blue), protein (red) and starch (green). Scale bar = 100 µm.

We aim to study the spatial trajectories of enteroendocrine hormones and the roles of metabolites across the human upper intestinal tract (oral cavity to the ileum) of 10 healthy individuals. This aim is broken down into two parts: firstly, we develop a spatiotemporal map of postprandial metabolites across the upper GIT, using an adapted naso-enteric sampling tube, to understand the impact of the cellular structure of food on luminal molecular profiles and enteroendocrine hormone responses. Secondly, as metabolites are partly driven by microbiotas, we integrate the ileal metabolome and microbiome profiles to elucidate the host-microbiome interplay in processing food in the upper gut. To assess the impact of food structure on metabolite profiles in the gastrointestinal tract, we compared three chickpea-based meals with distinct structural integrity: broken cell (BC), clustered cells (CC) and single cells (SC). We used chickpea as a food model, as the intact chickpea cell wall has been demonstrated to serve as a digestion barrier to modulates nutrient release in the upper small intestine^12^. We use a panel of 35 key quantified metabolites of food digestion and microbial metabolism representing a portfolio of carbohydrate (glucose, maltose, sucrose, ciceritol, stachyose, raffinose and trigonelline), protein digestion (alanine, valine, tryptophan, glutamine, methionine, aspartate, asparagine, tyrosine, phenylalanine and histidine), bile acids (TCDCA, GCDCA, TDCA, GDCA, TCA and GCA), as well as short chain fatty acids (SCFAs) (butyrate, propionate, acetate, fumarate and formate). The methodology is summarised in Fig. 1, and details pertaining to the study volunteers and analytical methods can be found in the “Extended Methods” section.

### Participants and diets

Ten healthy adults (four female and six male; aged 30.8 ± 2.4 years; BMI 24.9 ± 0.8 kg m^−2^) were recruited to participate in this study, which comprised three experimental phases targeting different regions of the GI tract: Phase 1, collection of gastric and duodenal samples; Phase 2, collection of ileal samples; and Phase 3, collection of oral samples. Phases 1 and 2 were conducted as inpatient visits, and Phase 3 was a short outpatient visit at the NIHR Imperial Clinical Research Facility (CRF), Hammersmith Hospital in London, UK. Participant characteristics and inclusion and exclusion criteria are provided in Supplementary Tables 1 and 2, respectively, and the study design and sampling protocol are summarised in Supplementary Fig. 1. All ten participants completed Phase 1. Eight completed Phase 2, with two withdrawals after Phase 1 due to the burden of intubation. Eight participants completed Phase 3; two additional healthy volunteers were recruited to maintain a total of ten participants for oral sampling. A CONSORT diagram is summarised in Supplementary Fig. 2.

In each sub-study, participants consumed BC, SC or CC in a randomised crossover order. All meals were matched for energy and macronutrient content. Oral, gastric, duodenal and ileal samples were collected at pre-determined timepoints for microscopic, metabolomic and microbiota analysis, with blood collected in parallel for enteroendocrine hormones analyses (Fig. 1a).

Using confocal microscopy with fluorescent staining of cell walls, proteins and starch, we tracked meal structural features across the mouth, stomach, duodenum and ileum of participants (Fig. 1b). The imaging data revealed that nutrient release was strongly dependent on the integrity of cellular boundaries. When cell walls remained intact in SC and CC meals, starch and protein were retained within the cellular matrix, resulting in delayed digestion and delivery of entrapped nutrients to the ileum. In contrast, BC meals released a larger proportion of starch and protein earlier in the proximal GI tract, leaving predominantly empty cell wall fragments in ileal aspirates.

### Metabolites as endogenous markers of bolus transit

**Fig. 2:**
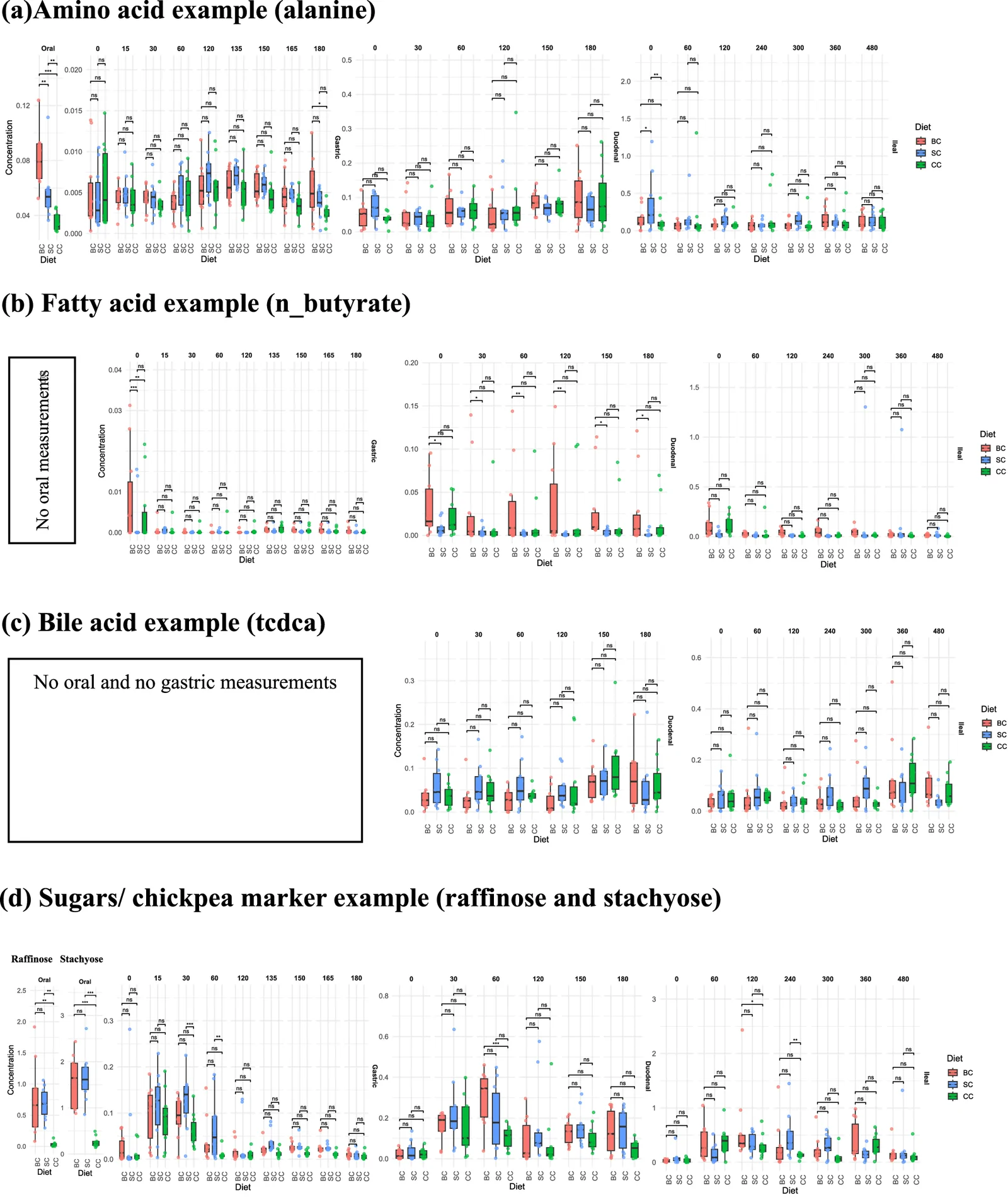
3D metabolite concentrations following three dietary interventions along the gastrointestinal tract. Concentrations (mmol/L) of **a** amino acids, **b** fatty acids, **c** bile acids and **d** sugars and chickpea markers are shown for Diet BC, corresponding to broken cells, Diet SC, corresponding to single cells and Diet CC, corresponding to cell clusters. Each point represents one metabolite concentration measurement from an individual participant. Repeated measurements were accounted for using a linear mixed-effects model with diet, timepoint and their interaction as fixed effects and participant identity as a random intercept. For oral measurements, diet was the only fixed effect because only one timepoint was available. Boxes show medians and interquartile ranges, whiskers extend to the most extreme value within 1.5 × the interquartile range, with all observations shown individually. Repeated measurements were analysed using linear mixed-effects models (concentration ~ diet × timepoint + (1|patient_id)) with diet, timepoint and their interaction as fixed effects and participant as a random intercept. Pairwise estimated-marginal-mean contrasts were Holm-adjusted within each timepoint. *P ≤ 0.05, **P ≤ 0.01 and ***P ≤ 0.001; ns, not significant.

Oligosaccharides, including stachyose, raffinose and ciceritol, are synthesised in the plant cytosol^13^. Also, trigonelline, a product of niacin metabolism and a biomarker of legume intake. As these compounds are not digested or adsorbed in the small intestine, we use them as markers of intestinal flow from the oral cavity to the ileal region (Fig. 2d and Supplementary Fig. 3d (blue box)). The summed stachyose + raffinose (SR) concentrations relative to the gastric baseline peaked in the stomach within 15–30 min postprandially, within 30–60 min in the duodenum (relative to local baseline), and at 60 min in the ileum (Fig. 2d). However, the timing of the peak concentration of SR varied across participants (Supplementary Figs. 4p, 5p and 6p). Approximately half of the participants exhibited a peak at 60 min relative to the ileal baseline, while the remainder showed later maxima (Supplementary Fig. 6p). Overall, the largest SR concentration fold change was observed in the ileum. Trigonelline followed a similar pattern, with the highest concentrations at 15–30 min in the stomach and 30–60 min in the duodenum (Supplementary Fig. 7m). In the ileum, trigonelline levels varied considerably between individuals. Ciceritol also peaked at 15–30 min in the stomach and at 30–60 min in the duodenum (Supplementary Fig. 7ai), with substantial inter-individual variability in the ileum.

### Food structure influences the oral-phase metabolic environment

The most abundant metabolite identified in the oral cavity prior to the bolus being swallowed was β-maltose (10–50 mM, Supplementary Fig. 7t), the dominant product of starch digestion, which showed a two-fold increase in BC compared to CC and SC after ingestion (~35 mM vs. 17 mM, respectively). The absence of intact cell walls in BC enhanced the susceptibility of starch to salivary amylase, leading to increased maltose release during the oral phase in this diet compared with SC and CC^14^, consistent with findings that intact chickpea cell walls limit starch bioaccessibility by slowing starch gelatinisation and restricting α-amylase access^12^.

Similarly, amino acids such as valine, tryptophan and isoleucine were present at comparable levels in salivary fluid following ingestion of BC and SC but lower after ingestion of CC. Alanine was highest in BC and the lowest in CC (Supplementary Fig. 7d). This pattern aligns with the known role of cell wall structure in modulating nutrient bio-accessibility. The lower salivary concentrations of amino acids following CC ingestion likely reflect the reduced immediate release of intracellular solutes during oral processing due to greater structural integrity.

Oligosaccharides (SR, trigonelline and ciceritol; Supplementary Fig. 7l, m, ai) were detected at lower levels in saliva after ingestion of CC compared to BC and SC. In the oral phase, SCFAs were 8–10 times lower than in the ileum (i.e. ~0.05 mM for propionate, and ~0.5 mM for acetate, Supplementary Fig. 7c, e). Oral acetate and propionate were lowest in BC compared to SC and CC, possibly reflecting the longer chewing time required for intact structures, resulting in greater molecular release.

### Food structure does not alter gastric emptying rates

Markers of meal flow, such as trigonelline and SR (Fig. 2d and Supplementary Fig. 8p, j), did not show significant differences in retention time across food structures (appearing in the duodenum within 30 min), suggesting a comparable rate of gastric emptying among the experimental meals. Previous studies suggested that a larger particle size of a solid meal reduced delay gastric emptying and thus reduce glycaemic response^15^; however, our observations at the microstructure level suggest minimal effect. Most metabolites exhibited a notable decline in concentration as the food bolus transitioned from the oral to the gastric phase. For instance, the β-maltose concentration decreased from ~35 mM (oral) to ~3 mM (gastric) in BC meals (Supplementary Figs. 3d and 7t), likely reflecting dilution by gastric secretions, a mixture of mainly water and hydrochloric acid, consistent with MRI studies showing ~50% intragastric volume increase within 40 min postprandially^16^. To control for potential confounding effects of fluid intake, volunteers were given a fixed volume of water with the chickpea meals during Visits 1 (oral) and 2 (gastric and duodenal), and no additional drinking was allowed during the sampling period. In Visit 3 (ileum), drinking was permitted but controlled as needed due to the extended 8-h sampling protocol.

### Food structure modulates nutrient release in the gastric phase

Differences in carbohydrate concentration between food structures observed in the oral phase were maintained in the gastric phase (Supplementary Fig. 7t): β-maltose, the marker of carbohydrate digestion, reached its peak in the stomach 15 min after the meal entry, with higher levels observed in BC compared to SC and CC. SR and ciceritol levels remained comparable in BC and SC but were noticeably lower in CC. Previous studies found that salivary amylase can remain active in the stomach when protected by a viscous bolus^17^. However, laboratory studies of cellular chickpea porridge meals suggest that these do not form a cohesive bolus, and that there is limited continuation of starch digestion by salivary amylase in the stomach^12^. Our data also suggest that the compact structure of CC further resists mechanical breakdown in the stomach, resulting in reduced nutrient release relative to BC and SC. Amino acids and SCFAs too were detected at lower concentrations in the stomach (0.01–0.05 mM), with a prolonged release phase initiated after 120 min, particularly for valine, asparagine, histidine and SCFAs, not differing substantially between intact and broken food structures (Fig. 2a, b and Supplementary Fig. 3a, b).

### Nutrient release and absorption in the duodenum

The duodenum is the first segment of the small intestine for food digestion, nutrient release and absorption. SR (Fig. 2d) increased in the duodenum from 30 min, peaked around 60 min and gradually returned to baseline over 180 min, indicating the timeframe of food retention in the duodenum. Higher levels of SR were observed in BC compared to CC postprandially at 60 min, as observed in the gastric region (Supplementary Fig. 7l). Starch digestion products, β-maltose and glucose (Supplementary Figs. 3d and 7r–t), were the dominant metabolites of chickpea digestion in duodenal fluids. Differences between food structures were most pronounced at 60 min, where BC resulted in 1 mM β-glucose and 5 mM β-maltose, representing a 3–4-fold increase compared to SC and CC, in alignment with the expected bioavailability for all three preparations. Amino acid concentrations increased from the gastric to the duodenal phase, particularly after 120 min postprandial (~0.05–0.1 mM). Amino acids, including valine, alanine, glutamine and histidine appeared higher in SC and CC than in BC (Fig. 2a and Supplementary Fig. 3a), confirming previous observations^1^. Duodenal bile acid profiles were modulated by food structure, with taurocholic acid (TCA) and glycocholic acid (GCA) exhibiting higher maxima in SC than in BC during 30–120 min (Fig. 2c, Supplementary Figs. 3c and 7y, z). Other bile acids, including glycochenodeoxycholic acid (GCDCA), taurodeoxycholic acid (TDCA) and glycodeoxycholic acid (GDCA), were comparable across meals. SCFAs were more abundant in the duodenum compared to the gastric phase (Supplementary Figs. 3c and 7w, x), with significant differences observed between food structures for butyrate (Supplementary Fig. 7a) that shows increasing levels for BC and CC during 120–180 min.

### Microbial metabolism and nutrient dynamics in the ileum

The ileum is the final segment of the small intestine and plays a key role in integrating digestive, microbial and hormonal signals. We observed that the stachyose + raffinose (SR) marker of intestinal flow peaked in ileal samples around ∼60 min post-ingestion (Fig. 2d). This is consistent with findings by Dagbasi et al.^18,19^, who showed that food-derived metabolite arrival in the ileum from ∼60 min contributes to PYY secretion through L-cell activation in the distal gut^18^.

We next examined how food structure influenced the arrival of digestible nutrients to the ileum. Meals composed of single (SC) or clustered (CC) chickpea cells led to higher concentrations of β-glucose and β-maltose in the ileum compared to broken-cell (BC) meals (Supplementary Figs. 3d and 7s, t). Again, this reflects the reduced starch digestibility in the proximal gut due to intact cell walls in SC and CC, enabling more digestible carbohydrate to reach distal regions. Similarly, amino acid concentrations were generally higher in the ileum than in the duodenum, with SC samples showing particularly elevated levels of valine, glutamine and methionine, tyrosine, phenylalanine, tryptophan and histidine at 120 min.

Short-chain fatty acids (SCFAs) in the ileum were highest in the fasted state and dropped sharply after food intake (Fig. 2b and Supplementary Fig. 3b). From 240 min onwards, SCFAs gradually increased, more specifically in the SC group, where we observed elevated n-butyrate, propionate and acetate. Since these profiles reflect delayed fermentation and downstream microbial activity, substantial inter-individual variation was observed (Supplementary Fig. 6). Formate, which was consistently higher in the ileum than in gastric or duodenal samples, showed only minimal postprandial fluctuation, suggesting it may represent a highly regulated microbial or host-derived metabolite in this region.

We also observed spatial and structural differences in bile acid dynamics across the gut. Total bile acid concentrations increased progressively from the duodenum to the ileum, peaking between 300–360 min (Fig. 2c and Supplementary Fig. 3c). Among primary bile acids, CC meals yielded higher TCA and GCA concentrations at 60 and 120 min. In contrast, TCDCA and GCDCA were elevated in SC compared to CC and BC at later timepoints. For secondary bile acids, CC samples exhibited higher levels of TDCA than SC during late-phase ileal sampling (Supplementary Fig. 6c). These differences likely reflect both the timing of substrate delivery and the extent of microbial activity (deconjugation), modulated by food microstructure.

As expected, substantial inter-individual variability was observed across all metabolites, in baseline levels, peak timing and postprandial magnitudes (Supplementary Figs. 4–8). Variability was more pronounced in microbiota-related metabolites (e.g. SCFAs) compared to food-derived metabolites (e.g. carbohydrates and amino acids). Also, ileal metabolites displayed greater variability compared to other GIT regions. This may be attributed to differences in microbial species and activities among individuals.

### Relationship between metabolite dynamics and enteroendocrine hormone response

**Fig. 3:**
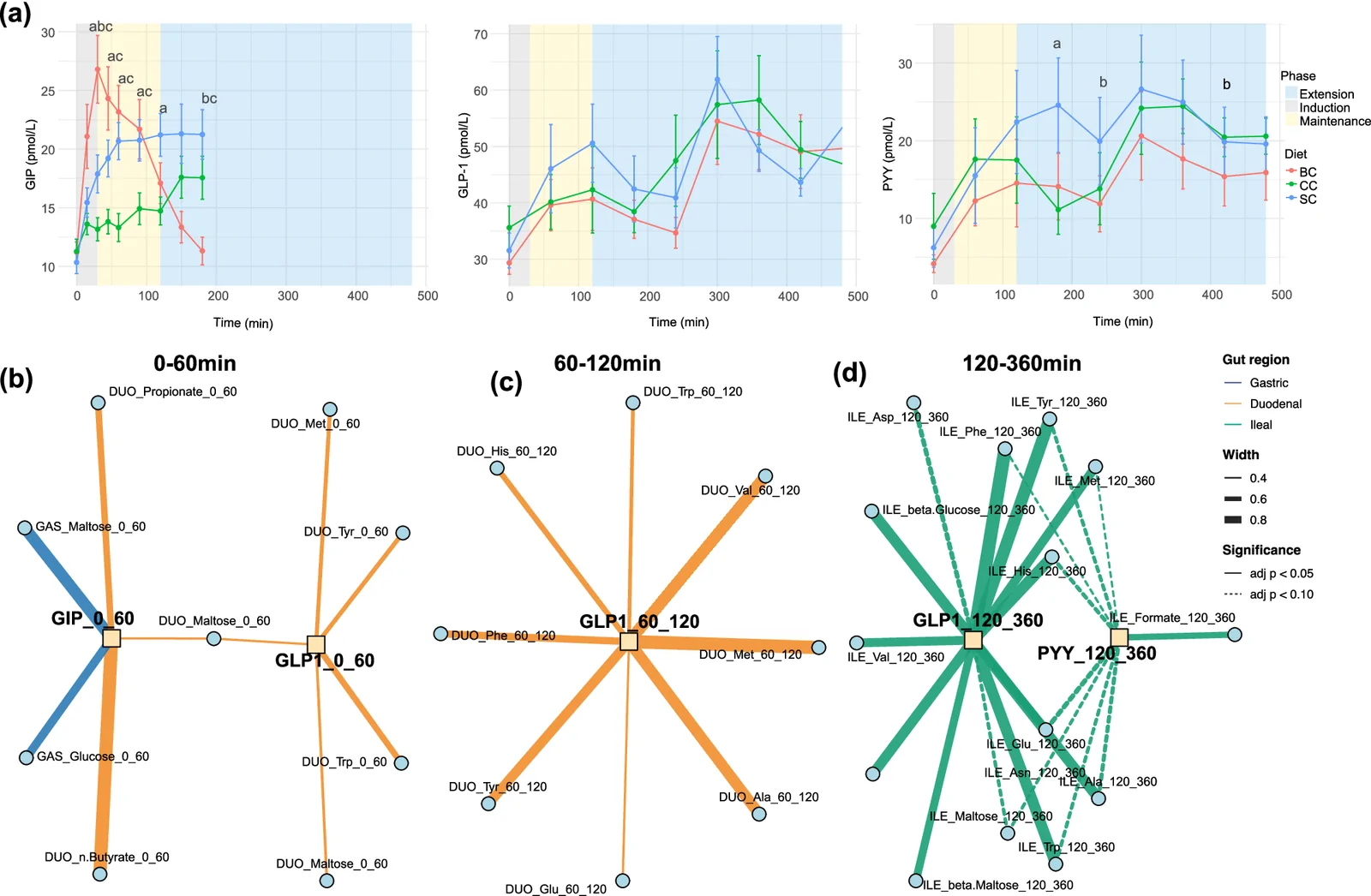
Enteroendocrine hormones and spatiotemporal trajectory of metabolites along the gastrointestinal tract. **a** Time-course changes in GIP, GLP-1 and PYY following the consumption of chickpea meals (blue: Intact single cells (SC), green: cell clusters (CC), red: broken cells (BC)). Data are presented as mean ± SEM. Two-way RM ANOVA showed significant meal × time effects for GIP (*P* < 0.001) and a significant diet effect for PYY (*P* = 0.0248), and time-point annotations show significant differences (adj. *P* < 0.05, Tukey’s) based on post hoc pairwise comparisons. Different letters (a, b, c) indicate significant differences between groups at the same time point: a - SC vs CC; b - SC vs BC; c- CC vs BC. GIP plot was adapted from Cai et al.^1^. **b**–**d** Temporal metabolite-hormone association networks across gut regions. Spearman correlation networks showing associations between regional luminal metabolites and gut hormone responses during the 0–60 min (**b**), 60–120 min (**c**) and 120–360 min (**d**) postprandial periods. Edge colour indicates the gut region in which the metabolite was measured (gastric, duodenal or ileal). Edge width reflects correlation strength. Edge line type indicates statistical significance after FDR correction (solid, adjusted *P* < 0.05; dashed, adjusted *P* < 0.10). Only associations passing the plotting threshold are shown. Gastric, duodenal and ileal metabolites are indicated with a prefix ‘GAS_’, ‘DUO_’ and ‘ILE_’, and standard three-letter abbreviations are used for amino acids.

We further investigated how metabolite dynamics across the upper GIT influence enteroendocrine hormone responses. Plasma concentrations of GIP, GLP-1 and PYY showed meal structure-dependent responses (Fig. 3a). Two-way ANOVA showed a significant diet × time interaction for GIP (*P* < 0.001) and a significant main effect of diet for PYY (*P* = 0.0248). BC elicited a rapid and higher GIP response with 0–60 min, whereas SC and CC produced prolonged more GLP-1 and PYY at later timeframes 120–480 min. Pairwise comparisons showed that PYY was significantly higher after SC than CC at 240 min (*P* = 0.0471) and 420 min (*P* = 0.0216) (Fig. 3a).

For interpretability, we arbitrarily divided the postprandial window into three time segments: 0–60 min (hormone induction phase), 60–120 min (hormone maintenance phase) and 120–360 min (hormone extension phase). Network correlation analyses were performed within each timeframe to determine which metabolites in which regions primarily impact the release of enteroendocrine hormones (Fig. 3 bcd).

During 0–60 min (Fig. 3b), the correlation analysis revealed significant associations between gastric glucose and maltose and duodenal maltose with GIP and GLP-1 responses (Fig. 3b). indicating that rapid starch digestion was the main driver for incretin response following BC meals.

During 60–120 min (Fig. 3c), associations with GLP-1 shifted towards duodenal amino acids, including valine, alanine, glutamate, methionine, tyrosine, phenylalanine and tryptophan (Fig. 3c). These suggested that intact food structures prolonged protein digestion in the duodenum, contributing to sustained GLP-1 secretion.

Postprandial 120–360 min was the time when nutrients peaked and was retained in the ileum (Fig. 2 and Supplementary Fig. 9a). Aligned with this, breath hydrogen profiles within 420–480 min showed increased for CC and SC but decreased for BC, suggesting from 420 min foods began to exit the ileum and enter the colon (Supplementary Fig. 9). During 120–360 min (Fig. 3d), the network analysis revealed the ileal metabolites play a major role in GLP-1 and PYY responses. For GLP-1, a group of amino acids (valine, alanine, glutamate, methionine, aspartate, asparagine, tyrosine, phenylalanine, tryptophan and histidine) and sugars (maltose and glucose) were associated with GLP-1. A similar but less-prominent pattern was observed for PYY. Notably, the strongest association for PYY was observed with formate, a microbiota-derived metabolite, suggesting that PYY secretion may be more responsive to microbiota fermentation signalling in the ileal environment.

In summary, food structure markedly influenced the trajectories of metabolites along the upper GIT and enteroendocrine hormone responses. BC meals enhanced carbohydrate exposure in the stomach and duodenum and were associated with stronger early GIP and GLP-1 release. In contrast, SC and CC meals more effectively sustained GLP-1 and PYY responses, likely through delayed delivery of sugars and amino acids to the distal intestine and increased microbiota-derived metabolites formate.

### The microbiome composition of the ileum is dynamic

**Fig. 4:**
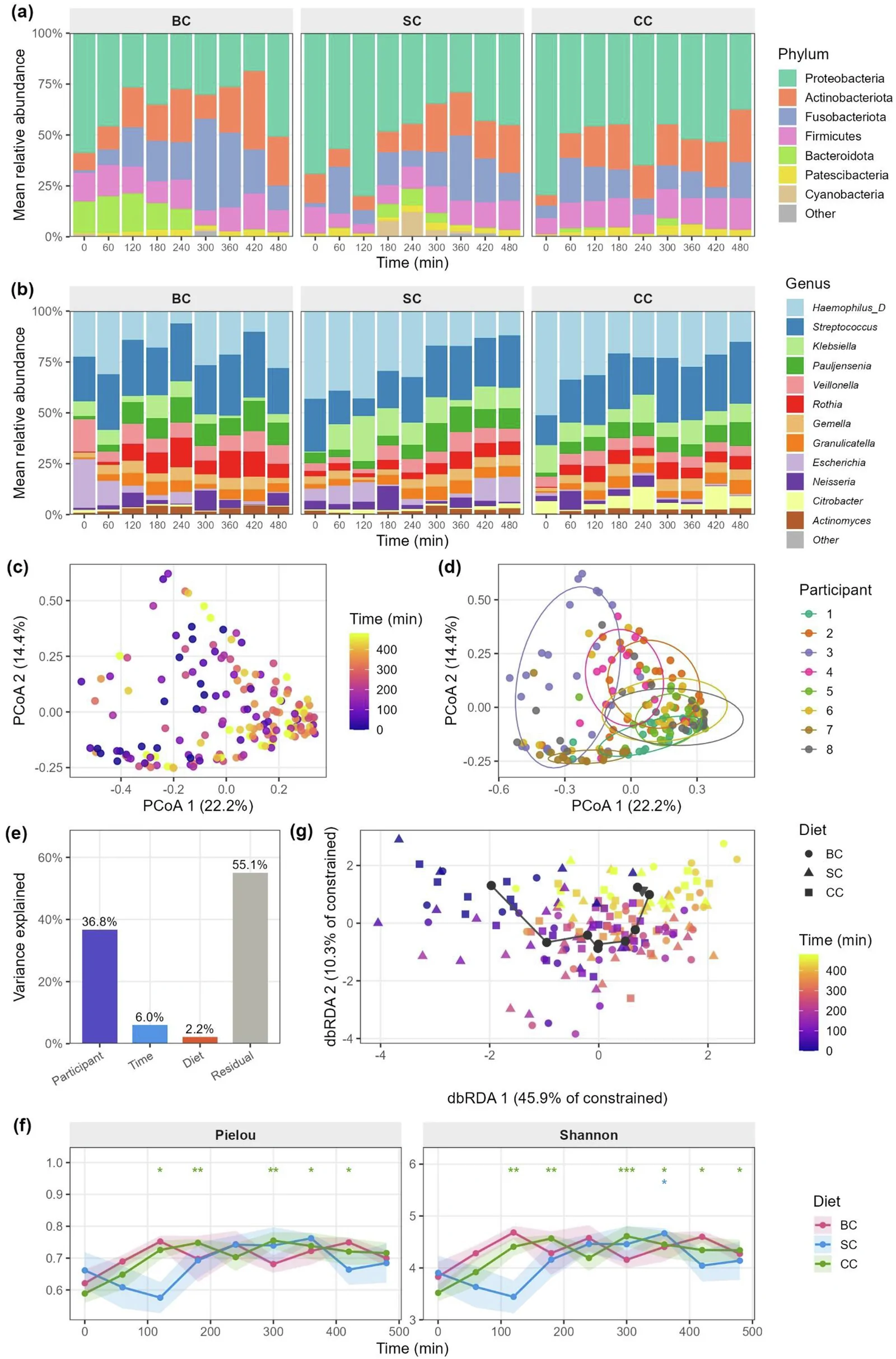
Dynamic restructuring of the ileal microbiome during digestion and its modulation by food structure. **a** Mean relative abundance of the eight most abundant bacterial phyla (remainder grouped as 'Other') across sampling time, shown separately for each meal; GTDB Firmicutes_A/B/C are aggregated with Firmicutes for display. Bars represent the mean across participants within each meal × timepoint. **b** As in (**a**), at the genus level, showing the twelve most abundant genera. **c** Principal coordinates analysis (PCoA) of Bray–Curtis dissimilarities among all ileal samples, with points coloured by sampling time. **d** The same ordination coloured by participant; ellipses denote 68% (1 s.d.) normal data ellipses per participant. **e** Variance partitioning of community composition (PERMANOVA, adonis2 on Bray–Curtis dissimilarities, 999 permutations restricted within participant): participant identity, sampling time and diet each explained a significant fraction of variation (participant *R*^2^ = 36.8%, *F*_7, 150_ = 14.31, *P* = 0.001; time *R*^2^ = 6.0%, *F*_8, 150_ = 2.04, *P* = 0.001; diet *R*^2^ = 2.2%, *F*_2, 150_ = 2.96, *P* = 0.001; residual 55.1%). Group dispersions did not differ significantly (betadisper: meal *P* = 0.25, time *P* = 0.27. **f** Shannon diversity and Pielou’s evenness over time for each meal, shown as the mean ± s.e.m. (line and shaded ribbon). Asterisks denote significant change from each diet’s fasted (0 min) baseline, coloured to match the corresponding meal, from two-sided estimated marginal means contrasts of each timepoint against baseline within each meal, adjusted for multiple comparisons by Dunnett’s method (*P ≤ 0.05, **P ≤ 0.01 and ***P ≤ 0.001). **g** Distance-based redundancy analysis (dbRDA; on Bray–Curtis dissimilarities) constraining on sampling time while conditioning out participant identity, thereby isolating the within-subject temporal signal that is otherwise masked by inter-individual variation in (**c**). Points represent samples, coloured by time and shaped by meal; the grey line connects the centroids of successive timepoints, with the arrow indicating the direction of postprandial progression. All analyses *n* = 8.

**Fig. 5:**
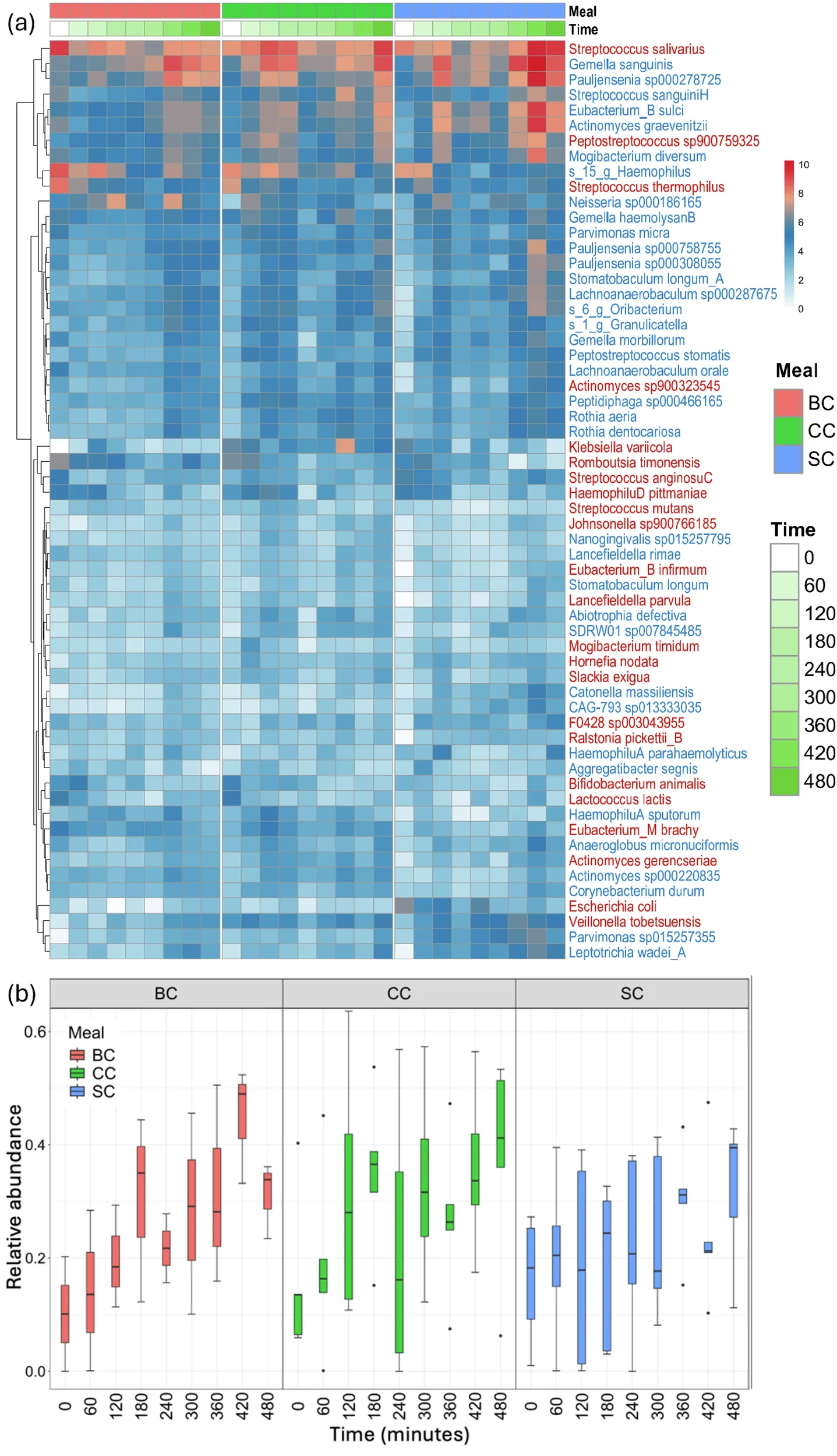
Variation in diversity and abundance of oral and ileal strains. **a** Relative abundance (log_2_ transformed) of the 50 most abundant species in the terminal ileum over time during consumption of each of the 3 diets. Species names highlighted in red did not show evidence of oral–ileal strain sharing; species names highlighted in blue did show evidence of oral–ileal strain sharing. **b** Proportion of the total bacterial abundance in the ileum attributable to strains shared with the oral cavity shown over time for each of the meals consumed. Box plots show the median (centre line), the 25th and 75th percentiles (lower and upper bounds of the box), and whiskers extending to the most extreme values lying within 1.5 × the interquartile range; data points beyond the whiskers are outliers and are plotted individually. The minima and maxima of the plotted data are the lowest and highest individual values shown for each box. All analyses *n* = 5 participants.

Analysis of the microbiome composition of the ileum during consumption of a meal demonstrates the highly dynamic nature of the microbial ecosystem. In the fasted state, the microbial community of the ileum of the volunteers is dominated by the genera *Haemophilus* and *Streptococcus*, including contributions from facultative anaerobes which may be considered from oral or upper gastrointestinal tract taxa, such as *Gemella, Veillonella, Peptostreptococcus, Actinomyces, Rothia* and oral *Streptococcus* species (Fig. 4 ab and Supplementary Fig. 10)^6^. We also note in our volunteers in the fasted state the presence of strict anaerobes generally characteristic of the colonic environment, such a *Bifidobacterium, Veillonella, Bacteroides* and *Faecalibacterium prausnetzii* (Fig. 4ab)^6^. Following ingestion of a meal we observe rapid changes in the community composition of the ileal microbiome (Fig. 4). Although the variance of the ileal microbiome is dominated by inter-individual variation (Fig. 4c), the microbial community structure and composition of the ileum changes postprandially, with 6% (*F* = 2.04, *P* = 0.001) of the total variance explained by changes over time (Fig. 4e). There is a clear time trajectory in the shift in microbial composition (Fig. 4g), and a small but statistically significant (*R*^2^ = 2.2%, *F* = 2.96, *P* = 0.001) impact of meal type. The arrival of the food bolus in the ileum, between 60 and 120 min, is associated with a decrease in microbial numbers^18^, but an increase in microbial community diversity. Diversity changed significantly over time (Shannon, *F*_8, 134_ = 4.40, *P* < 0.0001; Pielou, *F*_8, 134_ = 4.00, *P* = 2.8 × 10^−4^) and food structure significantly affected overall Shannon diversity (main effect of meal, *F*_2, 134_ = 3.19, *P* = 0.044), although the meal × time interaction was not significant for either metric (Shannon, *P* = 0.076; Pielou, *P* = 0.17) (Fig. 4f and Supplementary Fig. 10). There is a later secondary increase in microbial diversity at between 360 and 480 min for all three preparations, which is likely due to the arrival in the ileum of the second standard pasta-based meal given in the study and coincides with the second peak in SR (Figs. 2 and 4). The arrival of the meals in the ileum changes the relative abundance of several species (Fig. 5a). Species such as *Rothia aeria* and *Rothia denticariosa* increase in relative abundance following consumption of a meal, while species such as *Klebsiella varicola*, *Romboustia timonensis* and *Haemophilus parainfluenzae* are observed to decrease. The species with increased relative abundance are typically found in the oral cavity, while those which decrease are more commonly considered colonic anaerobes. We hypothesise that this may be indicative of the bolus carrying oral bacteria into the ileum, replacing the resident colonic bacteria.

### Evidence of strain sharing between oral and ileal compartments

To test the hypothesis that the oral and ileal microbial communities may mix while consuming a meal, we acquired strain-resolved metagenomes of saliva samples from five of the participants in this study, allowing direct comparison of strain sharing between oral and ileal compartments on a participant-by-participant basis. Figure 5b shows the relative abundance of strains shared between the oral and ileal compartments, as a proportion of total abundance in the ileum. Across the five participants for which we had paired data, we identified 106 strains that were shared between the oral and ileal samples (see Supplementary Data 1 taxonomic details of the shared strains). To quantify the proportional contribution of strains shared between oral and ileal samples, we analysed microbial abundance in the ileum during meal ingestion (Fig. 5b). We observe an increase in the proportion of oral strains in the ileal microbiome following ingestion of a meal, indicating that the arrival of the bolus in the ileum is associated with translocation of bacteria from the oral cavity. In the fasted state, around 10% of the total bacterial abundance in the ileum can be attributed to oral species. This rises with ingestion of the meals, with a first peak at around 120 min corresponding to the arrival of the chickpea meal in the ileum, and a second peak at 420–480 min following lunch to values for the as high as 50% of the total microbial community (Fig. 5b). There is a statistically significant (*p* < 0.05) increase in the proportion of shared strains in the ileum post-prandially for each of the three meals. Figure 4c highlights taxa (named in blue) where identical strains of the same species were found in the oral and ileal samples (Supplementary Data 1). Several oral species, most notably *Gemella sanguinis*, are observed to increase in relative abundance the ileum. Other highly prevalent shared species include *Mogibacterium diversum*, *Actinomyces graevnitzii* and *Streptococcus sanguinis* (Fig. 5a).

### Interactions between oral microbial strains, metabolites and enteroendocrine hormones in the ileum

**Fig. 6:**
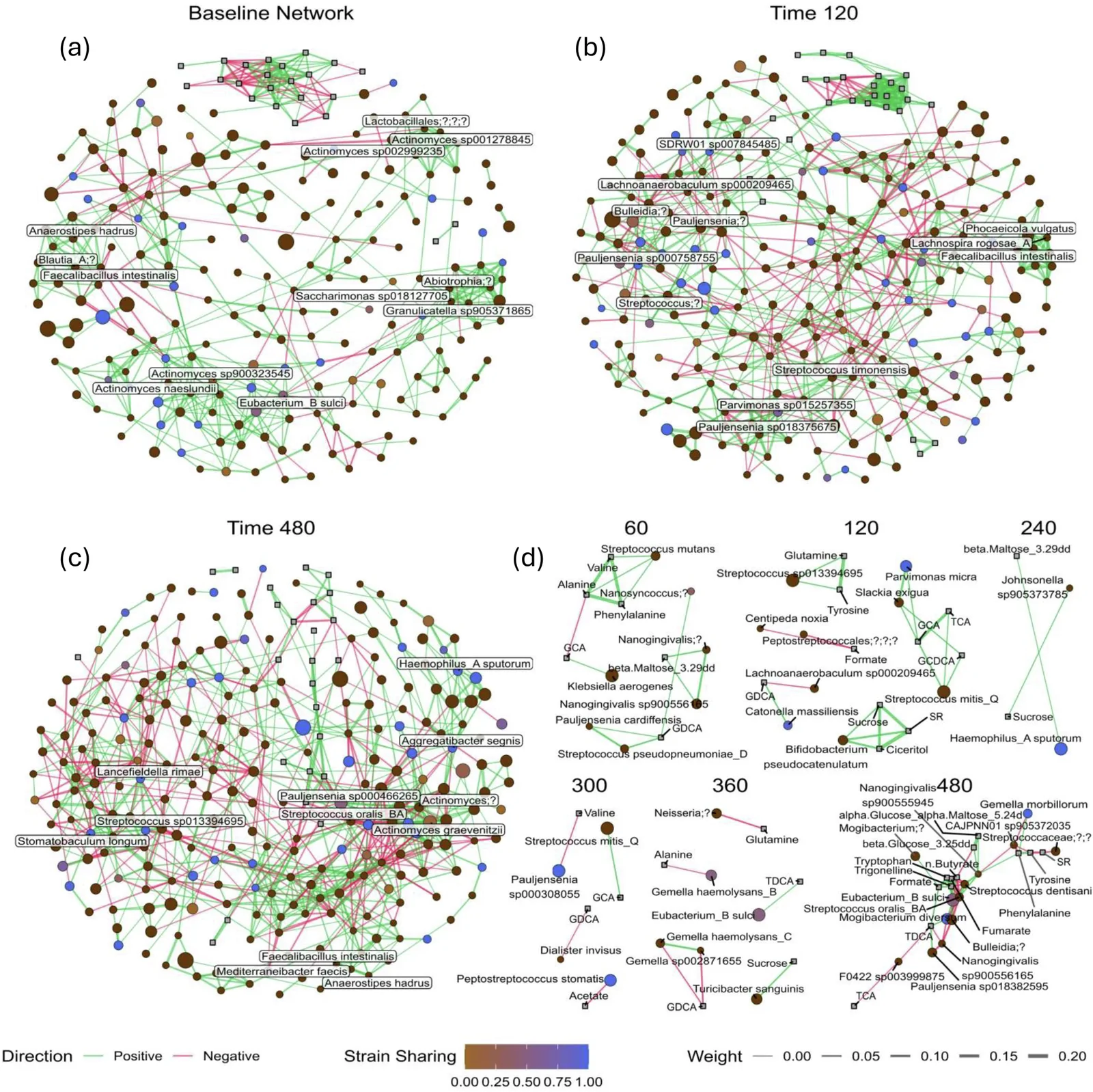
Co-occurrence analysis between ileal species and metabolites over time. Dense co-occurrence networks showing all microbial taxa detected, all interactions and all metabolites in the terminal ileum at **a** baseline, **b** 180- and **c** 420-min following ingestion of a meal. **d** The sparse networks are filtered to show only the metabolite-microbe interactions, and only for the timepoints where there are significant microbe-metabolite interactions. Nodes are coloured by the degree of strain sharing between oral and ileal compartments. Nodes in red are not shared, while nodes in blue are shared between the oral and ileal compartments.

Co-occurrence networks constructed from the strain resolved microbiome data and metabolites at baseline, 120 and 480 min show a lack of interactions between metabolites and microbial species at baseline (Fig. 6a–d), and most of the species observed had a low proportion of strain sharing between oral and ileal compartments (the colour coding of the nodes indicates the proportion of instances where a strain within a species is found to be shared between the oral and ileal compartments in an individual participant). The species with a higher proportion of strain sharing are mainly clustered around *Eubacterium sulci*, which showed negative associations with other parts of the network. A distinct, tightly clustered module of colon-associated strict anaerobes centred on *Faecalibacillus intestinalis*, with no evidence of strain sharing with the oral cavity. The baseline network has a high degree of modularity, with clear clustering of species, and the modularity reduces over time. At 120 min the network became more diverse, with a wider distribution of nodes showing evidence of strain sharing between oral and ileal compartments. At 480 min the network was more tightly integrated, with the majority of hubs within the clusters showing evidence of strain sharing. The cluster of colonic anaerobes centred on *F. intestinalis* observed at 120 min is much less tightly connected, and the abundances of the nodes are much lower in contrast to the shared nodes.

Microbe–metabolite associations were most prominent at 60–120 and 360–480 min, aligning with the meal arrival in the ileum (Fig. 6d). These associations involved SCFAs (acetate, n-butyrate and formate), amino acids (glutamine, phenylalanine), and various bile acids. During early timepoints, associations were primarily observed with non-adherent, shared strains, suggesting that microbial metabolism of freely available substrates (e.g. sugars, amino acids) may precede visible particle degradation. This aligns with previous findings of early GLP1 release in the absence of visible microbial colonisation. At later timepoints (360–480 min), associations increasingly involve strains with oral origins, including *E. sulci* and *M. diversum*, highlighting a potential role for these transmitted taxa in shaping ileal metabolite profiles.

We further investigated associations between microbiome composition in the ileum and hormone release (Supplementary Fig. 12). Only a few taxa were identified that correlated to GLP-1 release, mainly with species that did not show evidence of strain sharing, such as *Abiotrophia defective* and *Pauljensenia bouchesdurhonensis*. In contrast, we observed a larger number of associations between PYY release and ileal microbiome composition. While species that were negatively associated with PYY release were colonic species with no evidence for strain sharing, many of the species positively associated with PYY release were shared with the oral cavity. There was some overlap observed between the species associated with individual metabolites and hormone release, for example, *E. sulci* was associated with higher n-butyrate levels and with higher PYY release.

### Metabolite and microbial patterns in the ileum influence plasma enteroendocrine hormone profile

**Fig. 7:**
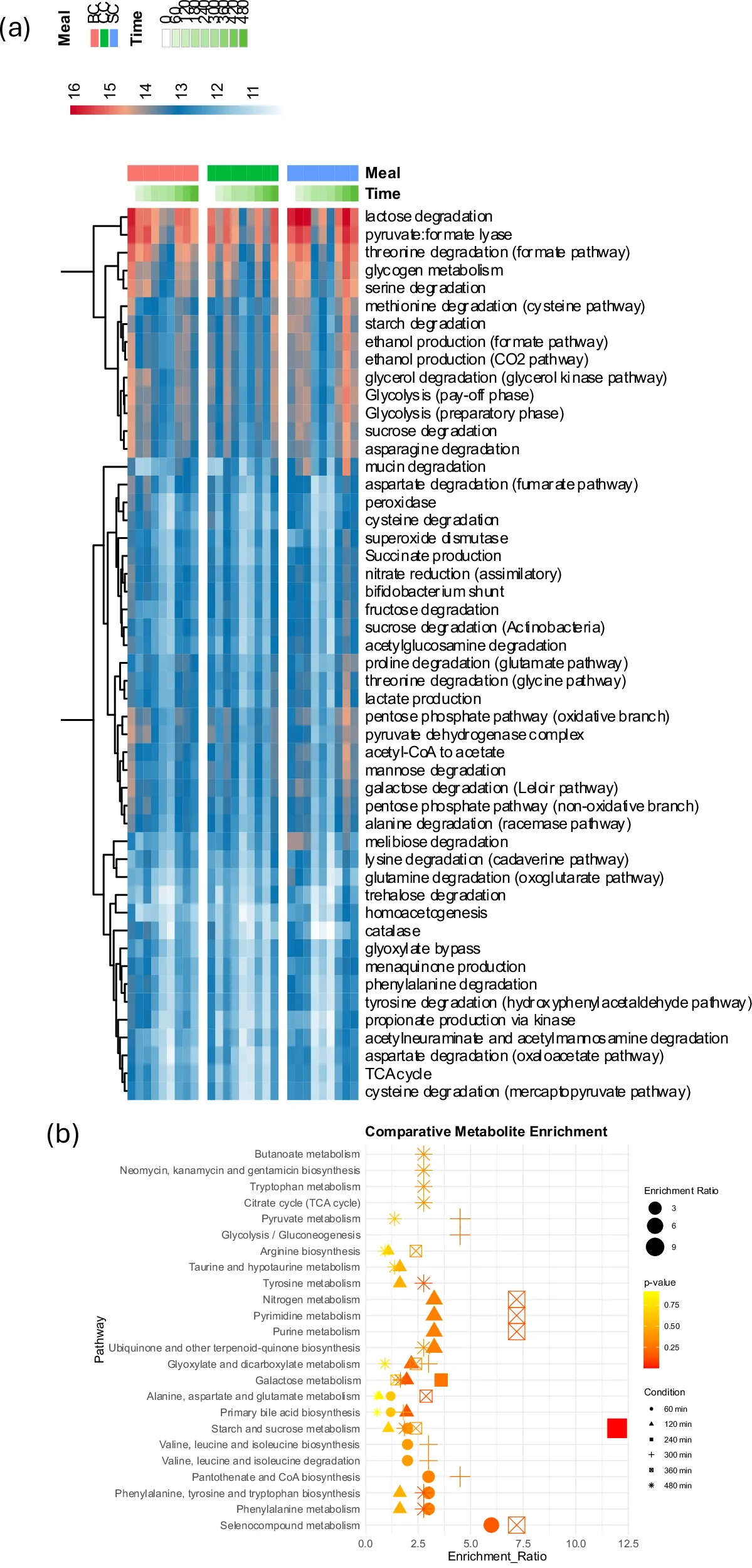
Microbiome- and metabolome-based functional analysis. **a** Heatmap showing KEGG pathways predicted using the microbiome data. **b** KEGG-based metabolic enrichment analysis across timepoints. A subset of metabolites, identified through significant microbe-metabolite associations in network analysis, was analyzed to reveal pathway-level changes at each timepoint. At 60, 120, 240, 300, 360 and 480 min, selected metabolites were tested for pathway over-representation in MetaboAnalyst using a custom background containing all measured metabolites. Enrichment ratio was calculated as observed pathway hits divided by expected hits and is represented by dot position and size, dot shape indicates time point and colour represents the unadjusted *P*-value. MetaboAnalyst also reported Holm- and FDR-adjusted *P* values; no pathway reached adjusted *P* < 0.05.

As well as taxonomic changes over time, we also identified shifts in functional pathways within the microbiome following arrival of the bolus (Fig. 7a). There are shifts in the abundance of several microbial pathways related to carbohydrate metabolism (starch, lactose and sucrose degradation, glycolysis) and SCFAs production (lactate production, acetyl-CoA to acetate and formate production via pyruvate formate lyase activity) over time for each of the meals.

As a complement to this analysis, metabolite set enrichment analysis (Fig. 7b) of microbiome-associated metabolites (Fig. 6d) revealed temporally coordinated shifts in ileal metabolic activity following chickpea ingestion (timepoint 0). At 60 min, phenylalanine metabolism, aromatic amino acid biosynthesis and pantothenate and CoA biosynthesis were enriched, indicating early microbial involvement in amino acid processing and cofactor production; selenocompound metabolism was also enriched at this time, though this may be attributable to the metabolite subset captured in the NMR panel. By 120 min, pathways linked to primary bile acid biosynthesis, galactose metabolism and glyoxylate/dicarboxylate metabolism were activated, suggesting early host-microbial coordination around bile acid signalling and sugar utilisation. At 240 min, strong enrichment of starch, sucrose and galactose metabolism was observed shortly after the first ileal sugar peak (Fig. 2), consistent with microbial engagement in carbohydrate breakdown and utilisation. At 300 min, pantothenate and CoA biosynthesis re-emerged, accompanied by glycolysis/gluconeogenesis and pyruvate metabolism, reflecting microbial energy harvesting from residual substrates. By 360 min, purine, pyrimidine and nitrogen metabolism dominated, consistent with microbial nucleotide synthesis and recycling; selenocompound metabolism was also enriched at this time, though again this may reflect the metabolite subset captured in the NMR panel. Finally, at 480 min, phenylalanine metabolism, aromatic amino acid biosynthesis and tyrosine metabolism were again prominent, marking a late-phase shift toward aromatic amino acid transformation likely driven by reduced carbohydrate availability and the use of amino acids as alternative substrates and biosynthetic precursors. Collectively, these results suggest that following a chickpea meal, the ileal microbiome engages in a sequential program of amino acid, carbohydrate and nucleotide metabolism, reflecting dynamic host-microbe co-metabolism across distinct postprandial phases.

Exploratory diet-stratified SEM analyses suggested meal-specific association patterns (Supplementary Fig. 14). BC displayed more taxon-metabolite associations than SC or CC, including positive associations of multiple taxa with aspartate and asparagine and predominantly negative associations with acetate and propionate. SC associations were concentrated around formate and bile acids, whereas CC showed few taxon-metabolite associations but broader positive associations of amino acids and carbohydrate resonances with PYY and GLP-1. Formate was positively associated with PYY under all three meal conditions. These patterns suggest that food structure may influence the coupling among ileal microbial, metabolic and hormonal responses. However, they remain hypothesis-generating because models were fitted separately by diet, and no associations survived the repeated-measures sensitivity analysis.

## Discussion

Our work describes for the first time the flow of metabolites in the GIT from the oral cavity to the ileum. Studies of the GIT metabolites and microbiota have relied mainly on indirect methods of assessment, such as stool or animal models. Recent studies, enabled by gut intubation and sampling methodologies, examined metabolite profiles in isolated GIT segments, such as the ileum^18^ and the duodenum^20^. A comprehensive picture across the entire upper GIT has been lacking.

Our study addresses this gap by providing a continuous mapping of metabolite flow from the oral cavity through to the stomach, duodenum and ileum. This offers an integrated perspective on digestion and nutrients availability across the GIT, which is crucial for understanding the nutrient sensing and the way the human GIT communicates across the GIT. Sensing of nutrients by chemosensory cells across the GIT plays a key role in transmitting food-related signals to organs and tissues that generate physiological regulatory responses. Spatially specific enteroendocrine cells (EECs) across the GIT respond to luminal nutrient stimuli and secrete enteroendocrine hormones^21^, for example, K-cells predominately located in the duodenum release GIP^22^, while L-cells residing in the ileum and colon release GLP-1 and PYY^23^. The distribution and flow of nutrients through distinct GIT regions influence the release patterns of these enteroendocrine hormones, which regulate local digestive processes and control key physiological functions such as insulin secretion and appetite control^23,24^.

By tracking metabolite concentrations across multiple GIT regions, we show a major impact of food structure on enteroendocrine hormones driven by the molecular environment and the ileum microbiome. Current dietary strategies to combat non-communicable disease focus on specific food or nutrient intake but overlook the role of food structure. Previous studies have shown that processed and unprocessed diets, matched for macronutrient and energy density, have different effects on appetite-regulating hormones and body weight^25^. One proposed mechanism is that the loss of cellular structure in the processed diet may alter nutrient release across the GIT and influence enteroendocrine secretion. Our ^1^H NMR profiling and quantification analysis identified key metabolites modulated by food structure- including carbohydrates, amino acids, bile acids and SCFAs.

To further understand how food structure shapes metabolic exposure along the GIT, we examined the spatial and temporal peak concentrations of key metabolites across the small intestine. Food structure had a marked effect on the timing and regional distribution of peak metabolite concentrations along the small intestine. In the duodenum, trigonelline, a chickpea-derived marker, reached peak levels earlier in clustered cell (CC) meals, followed by broken cell (BC) and then single cell (SC) structures, reflecting differences in digestion and transit. Similar temporal patterns were observed for stachyose and raffinose. In the ileum, amino acids and SCFAs were already present in the fasted state, suggesting they may represent background microbial fermentation products prior to meal arrival. Postprandially, amino acid concentrations increased from approximately 240 min onward, particularly following CC and BC meals, likely due to the delayed delivery of nutrient-rich digesta. Bile acids peaked later than other metabolites, with SC and BC meals showing more defined peak timing, whereas CC meals produced a highly variable bile acid profile, indicative of heterogeneous microbial processing. These observations highlight the importance of food matrices in shaping not only the composition but also the timing of nutrient exposure throughout the gut. Together, these patterns demonstrate that food structure dictates both the site and timing of nutrient sensing, with downstream effects on glycaemic response and hormone secretion.

BC released maltose/glucose rapidly in the mouth, stomach and duodenum, whereas SC and CC increased carbohydrate contents in the ileum, explaining different glycaemia responses reported between intact vs broken meals^26^. CC and SC generally released nutrients more slowly/gradually than BC, which may explain the prolonged satiety of intact meals reported previously^1,27^. SC led to higher waves of SCFAs in the ileum than BC, likely due to its increased nutrient availability in the ileum, which interacts with the local microbiome and enhances fermentation. We also observed higher levels of conjugated bile acids (BAs) in SC/CC than BC in the duodenum and ileum. One possible explanation is the interaction between plant cell wall components and bile acids, which may enhance bile acid retention^28^. BAs could activate TGR5 and stimulate GLP-1 secretion, which would improve glucose metabolism^29^. These findings provide mechanistic insights into how different food structures shape distinct luminal metabolite environments and affect metabolism, particularly glycaemia and appetite regulation. Our observations suggest that enteroendocrine hormone GIP release is closely associated with the rapid availability of carbohydrate in the stomach and duodenum in the BC, whereas the second peak of PYY and GLP-1 are related to the availability of amino acids and carbohydrates in the ileum.

We observed high variability in the metabolite flow across individuals, especially in microbiome-related metabolites and in the ileum region. This variability underscores the complexity of individual responses to dietary intake and the value of personalised nutrition approaches. The ileum, despite hosting a diverse array of bacterial species, has been less studied than the large intestine. Previous work has described the small intestinal microbiome primarily through fasted-state sampling and genus-level 16S rRNA analyses, limiting insight into dynamic changes or strain-level resolution^6^. Our study overcomes these limitations by capturing real-time, strain-resolved microbial profiles during digestion. While our fasted-state ileal communities reflected expected taxa such as *Streptococcus* and *Haemophilus*, we extended this static picture by showing that ingestion of a meal rapidly alters the microbial landscape, including the transient enrichment of oral-derived strains. These findings reveal the small intestine as a functionally dynamic environment, where microbial composition and activity shift in response to food structure and timing. In the fasted state, our results are qualitatively comparable to other studies, with a community dominated at the phylum level by Proteobacteria and the Firmicutes and at the genus level by *Streptococcus, Veillonella* and *Haemophilus*, but containing a tightly associated community of species more associated with the colonic microbiome^6,7^. In contrasting our findings to other studies, it is important to consider the sampling site and sequencing technology. Previous studies using luminal catheter aspirates and sampling capsules^7,9^ reveal a high degree of interindividual variability, although dominated by Firmicutes and Proteobacteria. Our sampling approach is unique in reliably controlling the sampling time and area (terminal ileum), where in the fasted state, we see small but measurable levels of strict anaerobes such as Bacteroides, which was observed by Leite et al. in the samples they define as 'Furthest Distance', denoting the extent of their sampling approach^10^.

Our findings can be contrasted against the small number of recent studies which have characterised the small intestinal (primarily duodenal) microbiome in disease states^30^. Recent studies have demonstrated that across a range of conditions, including obesity, SIBO and irritable bowel syndrome, the duodenal microbiota is relatively stable under fasted conditions, but rapidly increases in species richness following addition of a nutrient bolus^31^, closely matching our findings that microbial diversity rises following ingestion of a meal, as predicted by metabolic modelling of small intestinal microbiome dynamics^32^. Ectopic colonisation of the small intestine by oral bacterial species has been causally linked to mucosal inflammation in susceptible hosts, and overgrowth of oral-associated bacteria in the small intestine has been described in environmental enteric dysfunction^33,34^ and coeliac disease, where there is evidence for altered fibre fermentation in the small intestine^35^.

Our findings significantly extend this view of the ileal microbiome to encompass dynamics during ingestion of a meal. Data from mouse models has suggested that the small intestinal microbiome is cyclical and responds to meals^36^, but it was not clear if the same occurs in humans. We demonstrate that ingestion of a meal results in what in the literature was considered to be an 'ileal segment specific microbiome'^6^ being replaced by a microbiome which we have demonstrated through strain sharing analysis to originate in the oral cavity and to be enriched in strains identified in the saliva of the participants. We propose that these microbes are carried into the ileum via the bolus, where they are metabolically active. Previous studies have indicated that in ileostomy patients, there may be links between a dynamic microbial community and metabolite profiles in the ileum^37^, and in the ileum of healthy participants taking a probiotic supplement^38^. Here, we demonstrate that associations form between the abundance of oral microbial strains and microbially derived metabolites, which represent the strongest microbe-metabolite associations following passage of the food bolus. Oral-ileal translocation of microbes may have implications for a range of health conditions where overgrowth of oral bacteria in the small intestine and shifts in small intestine microbiome composition have been associated with disease status. Examples include small intestinal oral bacterial overgrowth (SIOBO), where overgrowth of oropharyngeal species, including *Lactobacillus*, *Streptococcus*, *Veillonella*, *Prevotella* and *Gemella,* has been associated with stunting in children^33^ and in Coeliac disease, where alterations in small intestinal microbiome metabolism of carbohydrates has been observed^34^. In these patient groups, there may be greater host susceptibility to the establishment of oral bacteria in the small intestine following passage with the food bolus. Furthermore, we identify associations between the abundance of oral microbial strains in the ileum and the release of the PYY hormone. This suggests a previously unknown mechanism whereby microbes from the oral cavity travel within the bolus to the ileum, where they influence the molecular environment of the ileum and hormone release.

Although the catheter passed through the nasal cavity and upper GIT before reaching the ileum, catheter-associated microbial contamination should be minimal. Oral-associated taxa increased in a meal-locked manner after arrival of the chickpea bolus and again after the standard lunch (Supplementary Fig. 11), rather than showing the time-invariant or progressive pattern expected from passive contamination. In addition, ileal microbiome composition followed a coherent temporal trajectory during digestion, and bacterial read fractions remained high throughout sampling. Ileal samples were also collected 24 h after tube placement. Thus, although low-level contamination cannot be fully excluded, it is unlikely to account for the observed postprandial microbial patterns.

### Strength and limitation

Repeated within-subject gastrointestinal (GI) sampling, performed in parallel with blood collection, enabled uniquely spatiotemporal profiling of luminal metabolites and their dynamic effects on enteroendocrine hormone responses. The study was conducted in a highly controlled clinical research environment, which minimised variability; under these conditions, a sample size of ten participants provided sufficient power to investigate our primary objectives on GIT metabolites and enteroendocrine hormones GIP, GLP-1 and PYY^1,12,17^. The study has several limitations. As a secondary objective, we attempted to profile the microbiota across the upper GIT; however, duodenal microbial biomass was low, resulting in insufficient reads for carrying out strain-resolved metagenomic analysis. In addition, oral and ileal samples were collected from the same participants on different study occasions, which may have underestimated their similarities due to day-to-day microbial variability. Lastly, although the repeated-measures crossover design provided high temporal resolution within participants, the metabolome and microbiome analyses were performed in a relatively small cohort. Further validation in larger cohorts will be required.

## Methods

### Overview of study outcomes and methods

The study was approved by the Health Research Authority and London-Camden and King’s Cross Research Ethics Committee (REC 19/LO/0962). The study has been prospectively registered at ISRCTN (https://www.isrctn.com/ISRCTN18097249, registration submitted on 22 January 2020). The study has received written informed consent from all participants for the use of samples and data for the present work. This study complies with all relevant ethical regulations.

The primary outcome was the impact of food cellular structure on the spatiotemporal profiles of luminal metabolites across the upper GIT and their association with enteroendocrine hormone responses (GIP, GLP-1 and PYY). The secondary outcome was the association between the GIT metabolome and microbiome profiles. Our laboratory has made significant progress in addressing the gaps identified above, with in vivo sampling of the small intestine of healthy individuals by using nasoenteric tubes to retrieve the chyme from intact regions of the GI tract^20,39^. Most recently, these samples have been analysed using ^1^H NMR, 16S sequencing and qPCR to study hormone release and microbial modifications of bile acids brought about by diet^18^. Within this paper, we have leveraged ^1^H NMR and shotgun sequencing to develop a pipeline characterising temporal profiles of metabolite bioavailability and high-resolution microbiome dynamics in nutrient-matched yet matrix-varied food, across the whole length of the intact small intestine.

### Test meals

Chickpea materials were provided by Quadram Institute Bioscience (Norwich Research Park, Norwich). Chickpeas were soaked and cooked using a Vorwerk Thermomix Version 5, following a procedure (Supplementary Text 1) to achieve three structures: broken cells, intact single cells and cell clusters. The cooked chickpeas were mixed with 15 g of Stutes no sugar-added blackcurrant jam and 115 g of Harley’s no added-sugar raspberry jelly to add flavour. Each test meal provided 41.16 g available carbohydrate, 6.58 g dietary fibre, 11.02 g protein, 3.74 g fat for a total 246 kcal. The amount of water served was adjusted to achieve the same portion size (760 g).

### Human study design

Ten healthy participants aged 18–65 years with a BMI of 18.5–30 kg m^2^ were recruited from the healthy volunteer database of NIHR Imperial Clinical Research Facility, based on the inclusion and exclusion criteria (Supplementary Table 1). Recruitment started on 11 February 2020, and sample collection was completed on 27 April 2022. Participant characteristics are provided in Supplementary Table 2. The protocol included one 4-day visit (Phase 1), three 3-day visits (Phase 2), and a 1-h study visit (phase 3) at NIHR Imperial Clinical Research Facility (CRF) at Hammersmith Hospital. A minimum wash-out period of 7 days was required between each main visit to avoid cross-over effects. A consort diagram showing participant flow through the study is provided in Supplementary Fig. 2.

Sex/gender was recorded by participant self-report during screening. The gender of participants was determined based on self-report. Sex/gender was considered during recruitment to include both female and male participants. The study was not designed or powered to detect sex- or gender-specific effects, and therefore no post hoc sex- or gender-stratified analyses were performed.

The study followed a double-blinded, randomised crossover design. Randomisation was performed by an independent researcher using a sealed envelope system (Sealed Envelope 2022). Both investigators and participants remained blinded until the completion of the study and data analysis.

As no prior studies had investigated the spatiotemporal metabolome and microbiome of the human gut in regard to food structure, sample size calculation was not feasible. Using similar intubation methods, Cai et al.^1^ and Dagbasi et al.^18^ investigated duodenal and ileal metabolite profiles, respectively and observed significant differences between dietary interventions with ten subjects. Thus, ten participants were recruited for this study.

### Phase 1 study—collection of gastric and duodenal samples

Ten participants (four female and six male) attended a 4-day inpatient visit at the NIHR Imperial Clinical Research Facility (CRF). The day before the study visit, participants were asked to refrain from caffeine, alcohol and strenuous exercise. Participants were also requested to fast overnight. On day 1, participants received two enteral feeding tubes placed in their stomach and small intestine (duodenum), respectively. Two naso-enteric tubes were placed by a trained medical doctor using the CORTRAK 2 Enteral Access System (Avanos Medical, UK). Tube position was verified by aspirate pH testing and review of the CORTRAK tracing after initial placement and on a daily basis before each intervention. On days 2, 3 and 4, participants received three chickpea test meals in a randomized order. Water was provided with the test meal only and was withheld thereafter to minimise dilution of gastrointestinal samples. Gastric and duodenal samples were taken before and after the test meals for 180 min (*T* = − 10, 0, 15, 30, 45, 60, 90, 120, 150 and 180 min). Blood samples were collected through a cannula placed in the antecubital fossa used for measuring enteroendocrine hormones GIP, GLP-1 and PYY. On day 4, enteral tubes were removed, and participants were discharged. All participants adhered the protocol and received intended interventions. Participants received £500 for completing Phase 1 study.

### Phase 2 study—collection of ileal samples

Two participants withdrew the Phase 1 study (one due to the burden of intubation and one due to the time commitment), leaving eight participants (four female and four male) who entered the Phase 2 trial. Eight participants attended three 3-day inpatient visits at NIHR Imperial CRF with a minimum 7-day washout period between study visits. Participants were asked to refrain from caffeine, alcohol and strenuous exercise before attending the visits. Participants were also requested to fast overnight. On each visit, participants received one of these chickpea-based meals, in randomised order. On day 1, a nasoenteric tube was inserted into the participants’ terminal ileum under fluoroscopy guidance^39^ by a consultant radiologist at the Imaging Department of Charing Cross Hospital. Day 2 allowed participants to acclimatize to the diet and environment. Tube position was verified by aspirate pH testing and fluoroscopy prior to ileal sampling. On day 3, ileal samples were collected before breakfast and every 60 min until 480 min. Participants were permitted to consume water ad libitum during the 480-min sampling period to maintain hydration and tolerability of prolonged intubation. Blood samples were collected at the same intervals for measuring the enteroendocrine hormones GLP-1 and PYY. All eight participants adhered the protocol and received the intended interventions. Participants received £350 for the visit.

### Phase 3 study—collection of oral samples

The recruitment target for the Phase 2 study was 10 participants. Those who completed phases 1 and 2 were preferentially invited to take part in Phase 3. Eight participants responded and attended a 1-h visit at the NIHR Imperial CRF. In addition, two new participants were recruited from the healthy volunteer database of NIHR Imperial CRF to take part in the Phase 3 visit only. During the visit, participants were instructed not to brush their teeth or use mouthwash for at least 2 h before arriving. They were asked not to consume any food, drink, chewing gum or tobacco. Two millilitres of saliva were collected as a baseline. Participants received £50 for completing the Phase 3 study.

The chickpea meals were prepared using the same protocol as in phases 1 and 2 and then weighed out to approximately 12–18 g to achieve equivalent macronutrient content (each meal provided 1.0 g of starch). Participants were asked to chew the chickpea meals, served in a randomised order, to the urge to swallow, stop chewing and provide us with oral samples. The oral samples were immediately stored at −80° for further analysis. All ten participants adhered the protocol and received intended interventions.

### Metabolite extraction

Oral, gastric, duodenal and ileal samples were centrifuged at room temperature for 15 min at 3000 × *g* to remove particulates. The resulting supernatant was collected, and 450 μl of the supernatant was used for metabolite extraction. Metabolites were extracted by adding 1 ml methanol, 2 ml chloroform and 1 ml water (ratio 1:2:1). Samples were vortexed for 1 min and centrifuged at 4° for 20 min at 3000 × *g*. After centrifugation, samples were separated into the aqueous and the organic phase. The aqueous phase was evaporated in a Speed Vacuum concentration (Eppendorf Concentrator Plus) to dryness and stored at –80 °C for further analysis.

### NMR spectroscopic analysis

The aqueous phase of the GIT samples was re-constituted in 640 μL of H2O and sonicated for 10 min. Samples were checked after sonication, and there was no visible precipitation. Sixty microliters of NMR buffer [1.5 M KH_2_PO_4_ buffer (pH 7.4, 100% of deuterium oxide (D_2_O), 2 mM sodium azide and 1 mM TSP (3-trimethylsilyl-[2,2,3,3,-2H4]-propionic acid sodium salt)] was mixed with 540 μL of samples and the mixture was further transferred to 5 mm NMR tubes. Quality controls were prepared by pooling 100 μl of each sample, independently for oral, gastric, duodenal and ileal samples. A total of 30 oral samples, 169 gastric samples, 116 duodenal samples and 174 ileal samples were analyzed. No technical replicate acquisitions were performed.

^1^H NMR spectroscopy was performed at 300 K on a Bruker 600 MHz spectrometer 66 (Bruker Biospin, Karlsruhe, Germany). It followed a standard one-dimensional pulse sequence with saturation of the water resonance RD – gz,1 – 90° – t – 90° – tm – gz,2 – 90° – ACQ (noesygppr1d) where RD is the relaxation delay, 90° represents the applied 90° radio frequency (rf) pulse, t is an interpulse delay set to a fixed interval of 4 μs, RD was 2 s and tm (mixing time) was 100 ms. Water suppression was achieved through irradiation on the water signal during RD and tm. Each 21 spectrum was acquired using 4 dummy scans followed by 64 scans and collected into 64 K data points. The receiver gain was 90.5. A spectral width of 20,000 Hz was used for all the samples. Prior to the Fourier transformation, the FIDs were multiplied by an exponential function corresponding to a line broadening of 0.3 Hz.

### NMR data processing

Multivariate statistical analysis was performed on spectral data. Each spectrum (~24 K spectral variables) was automatically phased, baseline corrected, digitized over δ −0.5 to 10 and imported into MATLAB (R2021a, Mathworks, Inc.; Natwick, USA), using in-house scripts. Spectral regions corresponding to the internal standard (δ −0.5 to 0.5) and water (δ 4.7–4.9) were excluded.

### Metabolite identification

Statistical spectroscopic tools such as statistical total correlation spectroscopy (STOCSY) and subset optimisation by reference matching (STORM) were used to identify spectra signals. Internal and external databases such as the Human Metabolome Data Base (HMDB; http://hmdb.ca/) and the Biological Magnetic Resonance Data Bank (BMRB; http://www.bmrb.wisc.edu) were used for assignment purposes. Arguably, these assignments have not been confirmed, as it would require spiking.

### Metabolite quantification

From the 1H-NMR metabolite profiles, we chose 35 metabolites to be quantified, based on the most likely metabolites encountered in consumed legumes. These include five sugars (stachyose, raffinose, maltose and glucose), metabolic acids like SCFAs (n-butyrate, propionate, formate, fumarate and acetate), nine amino acids (valine, alanine, glutamine, methionine, histidine, aspartate, asparagine, tyrosine and phenylalanine), six bile acids (TCA, GCA, TDCA, GDCA, TCDCA and GCDCA) and metabolic markers of chickpeas such (trigonelline, tryptophan and ciceritol).

The quantification of metabolites was achieved using in-house developed routines written in both JavaScript and R. NMR spectra were processed according to the Bruker IVDr standard procedures^40^. No further processing deemed necessary. Normalisation is not required prior to quantification and can be applied on the extracted concentrations when necessary. The following steps were followed for quantification:

1) The metabolites of interest were grouped in spectral regions of interest (SROI).

2) For each SROI, a list of signals was established with attributes that define their characteristic patterns (chemical shift, linewidth, multiplicity and scalar coupling). All the patterns present in an SROI were included, even for unknown signals.

3) Those parameters were used as initial input for a gradient descent optimisation (Levenberg–Marquardt) algorithm to minimise the difference between the experimental and the fitted pattern.

4) The resulting models were graded using a comprehensive heuristic, including metrics specific to the optimisation error and metrics capturing the correlation of integrals registered for patterns known to belong to the same compound.

5) Models with lower grades were visually inspected using a suite of interactive web tools build in JavaScript^41^.

6) The resulting areas under the curve were then converted to concentrations using the ERETIC internal standard and calibration procedure^42^ that is part of the Bruker IVDr protocol.

Steps 1–2 were repeated for the 2 different sample matrix types, i.e. for both the ileum and chickpea samples.

During meal preparation, metabolites may be affected by processing loss (mechanical damage, leaching), thermal degradation, enzymatic degradation, oxidation, prolonged storage times and complex chemical interactions. Hence, we have built in multiple layers of redundancy by tracking molecule families. For example, in addition to using trigonelline, tryptophan and ciceritol as metabolic markers for chickpeas, we also used stachyose and raffinose to track the passage of chickpeas through the GI-tract. These two small soluble cell wall components are resistant to small degradational enzymes but are released rapidly in the oral cavity. Furthermore, for the GI-samples, it was easier to robustly quantify stachyose-raffinose as a joint peak in the NMR data as opposed to two separate molecules.

### Metabolite modelling

Metabolites were quantified in all the samples, but the metagenomic sequencing was restricted to ileal samples, as gastric samples were expected to be sterile, and duodenal samples, though processed, exhibited extremely low microbial abundance and were consequently discarded.

For metabolite analysis, data imputation was performed using the MissForest algorithm to account for inconsistent timepoints across the 10 gastric, 10 duodenal and eight ileal participants. Oral samples were excluded from this imputation due to extreme data sparsity, with only eight oral metabolites quantified compared to 28 in the gastric, duodenal and ileal datasets. Additionally, oral metabolite profiles differed substantially from gastric timepoint 0, and data were available for only 8 of the 10 volunteers. Nevertheless, oral samples were analysed separately, and two additional participants were included to compensate for missing data points in this subset.

Principal component analysis (PCA) (Fig. 1b) was conducted following imputation, using centred and scaled metabolite concentrations to assess participant-level variation and potential outliers. Metabolite concentrations were averaged across all participants per timepoint and diet, and the resulting means were visualised in three-dimensional graphs (Supplementary Fig. 3). Box-and-whisker plots, available in the Supplementary Information (Fig. 2 and Supplementary Fig. 7), provide further details on participant variance. Diet and time-dependent differences in metabolite concentration were analysed using linear mixed-effect models with diet, timepoint and their interaction as fixed effects and participant identity as a random intercept. Oral models included diet as the only fixed effect because only one timepoint was available. Pairwise diet contrasts at each timepoint were calculated from estimated marginal means, with Holm correction for the three within-timepoint comparisons.

The GI location (gastric, duodenal and ileal) and timepoint of peak concentration for each metabolite, averaged across metabolite families within each diet, were identified and represented in a scatter plot (Supplemental Fig. 8). This qualitative visualisation highlights the temporal and spatial distribution of peak concentrations for amino acids, bile acids, chickpea markers, fatty acids and sugars following ingestion. Finally, oral sample data (Fig. 2 and Supplementary Fig. 7) were analysed separately from the gastric, duodenal and ileal datasets to illustrate inter-individual variability, upper and lower concentration limits and median concentrations.

### DNA extraction and metagenomic sequencing

DNA extraction and metagenomic sequencing was only done for ileal samples, as gastric samples were too acidic and duodenal sample quality was too low to retrieve metagenomes. DNA extraction was carried out per the manufacturer’s instructions with the MP Bio fast DNA spin kit for soil (MP Biomedical, Solon, USA). Ileal aspirates were transferred to Lysing Matrix E tubes, where 980 µL of sodium phosphate buffer and 120 µL of MT buffer were added. The samples were homogenised in the FastPrep24 bead-beating instrument (MP Biomedicals, Solon, USA) for 3 min (3 runs of 60 s each, with 5 min rest on ice in between). Afterwards, samples were centrifuged at 14,000 × *g* for 15 min, and the sample supernatant was transferred into clean 2 mL microcentrifuge tubes with 250 µL of protein precipitate solution. The tubes were mixed by shaking by hand 10 times before centrifugation at 14,000 × *g* for a further 10 min. The supernatant was transferred to 5 mL tubes with a resuspended binding matrix (1 mL of silica slurry that binds DNA from lysates), and the tubes were inverted for 2 min allowing the DNA to bind. The tubes were placed in a rack and allowed to settle for 1 h. Then, 500 µL of the supernatant was removed, and the binding matrix was resuspended in the remaining supernatant. The washing steps involved the transfer of 700 µL of the mixture to a SPIN filter centrifuged at 1000 × *g* for 2 min to empty the catch tube and transfer the leftover mix. This process was repeated until all the mixture had been added to the SPIN filter. The SPIN filter was washed with 500 µL of ethanol-based wash solution SEWS-M. The samples were centrifuged at 14,000 × *g* for 5 min, and the catch tube was emptied. Then, samples were centrifuged a second time (Dry spin) at 14,000 × *g* for 5 min to remove the residual wash solution. The catch tube was replaced with a clean tube (1.5 mL LoBind Eppendorf^®^ tubes), and samples in the SPIN filter were air-dried for 10 min at room temperature and 5 min at 37 °C. Then, 65 µL of DNA extraction solution was added to the Binding Matrix and incubated at room temperature for 5 min before being centrifuged at 6600 × *g* for 2 min with lids open to bring eluted DNA into the tube. The SPIN Filter was discarded, and the eluted DNA was stored at −20 °C.

Genomic DNA was normalised to 5 ng/µL with elution buffer (10 mM Tris HCl). A miniaturised reaction was set up using the Nextera DNA Flex Library Prep Kit (Illumina, Cambridge, UK). 0.5 µL Tagmentation Buffer 1 (TB1) was mixed with 0.5 µL Bead-Linked Transposomes (BLT) and 4.0 µL PCR-grade water in a master mix, and 5 µL was added to each well of a chilled 96-well plate. About 2 µL of normalised DNA (10 ng total) was pipette-mixed with each well of Tagmentation master mix and the plate was heated to 55 °C for 15 min in a PCR block. A PCR master mix was made up using 4 µL Kapa2G buffer, 0.4 µL dNTP’s, 0.08 µL Polymerase and 4.52 µL PCR-grade water, from the Kap2G Robust PCR kit (Sigma-Aldrich, Gillingham, UK), and 9 µL added to each well in a 96-well plate. About 2 µL each of P7 and P5 of Nextera XT Index Kit v2 index primers (catalogue No. FC-131-2001 to 2004; Illumina, Cambridge, UK) were also added to each well. Finally, the 7 µL of Tagmentation mix was added and mixed. The PCR was run at 72 °C for 3 min, 95 °C for 1 min, 14 cycles of 95 °C for 10 s, 55 °C for 20 s and 72 °C for 3 min. Following the PCR reaction, the libraries from each sample were quantified using the methods described earlier and the high-sensitivity Quant-iT dsDNA Assay Kit. Libraries were pooled following quantification in equal quantities. The final pool was double-SPRI size selected between 0.5 and 0.7× bead volumes using KAPA Pure Beads (Roche, Wilmington, US). The final pool was quantified on a Qubit 3.0 instrument and run on a D5000 ScreenTape (Agilent, Waldbronn, DE) using the Agilent Tapestation 4200 to calculate the final library pool molarity. qPCR was done on an Applied Biosystems StepOne Plus machine. Samples quantified were diluted 1 in 10,000. A PCR master mix was prepared using 10 µL KAPA SYBR FAST qPCR Master Mix (2×) (Sigma-Aldrich, Gillingham, UK), 0.4 µL ROX High, 0.4 µL 10 μM forward primer, 0.4 µL 10 μM reverse primer, 4 µL template DNA, 4.8 µL PCR-grade water. The PCR programme was: 95 °C for 3 min, 40 cycles of 95 °C for 10 s, 60 °C for 30 s. Standards were made from a 10 nM stock of Phix, diluted in PCR-grade water. The standard range was 20, 2, 0.2, 0.02, 0.002 and 0.0002 pmol. For the ileal samples, the pooled library was then sent to Novogene (Cambridge, UK) for sequencing using an Illumina NovaSeq 6000 instrument with paired-end 150 bp sequencing, with sample names and index combinations used. Demultiplexed FASTQ’s were returned on a hard drive. A sequencing depth of ~12.6 GB per sample was achieved. For the oral samples, the pooled library was sequenced by Novogene (Cambridge, UK) using an Illumina Novaseq X instrument with paired-end 150 bp sequencing to a sequencing depth of ~25 GB per sample.

### Metagenomic data processing

The raw data were processed using the MG-TK pipeline (formerly MATAFILER)^43^. Specifically, reads were filtered based on quality using sdm (simple demultiplexer). Reads matching the human or chickpea genome were identified with Kraken2 and filtered out. The raw data contained an average of 86.4 million reads, with an average of 65.4 million reads remaining after filtering for host and diet (chickpea) DNA and sequence quality control. Samples from the same individual were co-assembled using MEGAHIT and binned using SemiBin. Reads were mapped to the assembly with Bowtie 2. Genes were predicted with Prodigal, and SNP calling was performed using bcftools. MAGs were clustered into metagenomic species (MGS) and then taxonomically profiled based on marker genes using GTDB-tk and the LCA algorithm. For each high-quality MGS, a phylogenetic tree was calculated based on SNP-resolved gene orthologues using IQ-Tree. Phylogenetic trees were then used for strain-level analysis following the procedure published previously^44^. Briefly, this approach assumed a presence of one dominant strain per sample and a case of strain sharing was defined if a pair of samples from the same individual formed a monophyletic clade and the distances between them fell within a tree-specific phylogenetic distance threshold. For each MGS and participant at a given timepoint, the strain sharing ratio have been calculated as a proportion of samples with the same strain among all oral and ileal samples. The pipeline has been used an assembly-dependent manner, so the counts are reads mapping to marker genes in metagenome assembled genome bins. Raw reads have been normalised for gene length/copy number.

Networks combining species and metabolite data for each time point were produced using SPIEC-EASI. Each network used parameters nlamba 100, lamba.min.ratio 1e-1, method 'glasso', sel.criterion 'bstars' and pulsar parameter rep.num 100. Species or metabolites which were observed in 20% or fewer of the samples were filtered before network construction. For visualisation vertices with degree 0 were removed.

### Enteroendocrine hormone assays

Blood samples were collected into lithium heparin tubes with DPP-IV (10 µl ml^−1^ blood, Merck Millipore), aprotinin (10,000 kIU ml^−1^ blood, Nordic Pharma) and AEBSF (pefabloc, 1 mg ml^−1^ blood) for GIP, GLP-1 and PYY analysis. Plasma GIP concentrations were measured by Human GIP ELISA kits from (EZHGIP-54K, Merck). All these assays were performed as per the manufacturer's instructions. GLP-1 and PYY concentrations were measured by the in-house RIA method^45^. Statistical differences between groups were analyzed using repeated-measure two-way ANOVA followed by Tukey’s post-hoc test.

### Statistics and reproducibility

The study followed a randomized, double-blinded crossover design in which participants received chickpea-based meals with three different cellular structures in randomized order. Biological replication was provided by participants, with repeated measures collected across diet, gastrointestinal region and time point. No statistical method was used to predetermine sample size, as no previous study had generated comparable spatiotemporal metabolome and microbiome data across the oral, gastric, duodenal and ileal regions of the human gastrointestinal tract. The sample size was selected based on the feasibility of repeated human gastrointestinal intubation and on previous studies using similar sampling approaches.

Randomisation of meal order was performed by an independent researcher using a sealed-envelope system. Participants and investigators were blinded to meal allocation until completion of the study and data analysis. All participants who completed each study phase were included in the corresponding analyses. Two participants withdrew after Phase 1 because of the burden of intubation or time commitment and therefore did not contribute samples to Phase 2. Metagenomic sequencing analysis was restricted to ileal samples because gastric samples were expected to have very low microbial biomass, and duodenal samples yielded insufficient microbial DNA/read depth for strain-resolved metagenomic analysis.

For community composition (beta diversity), community analyses were performed in R version 4.6.0 using phyloseq 1.56.0, vegan 2.7-3, nlme 3.1-169, emmeans 2.0.3, ggplot2 4.0.3 and patchwork 1.3.2. Principal coordinates analysis (PCoA) was performed on Bray–Curtis dissimilarities. Effects of participant, sampling time and meal on community composition were tested by PERMANOVA (adonis2, vegan), fitting participant, time (as a categorical factor) and meal sequentially (by = 'terms') with 999 permutations. Homogeneity of multivariate dispersions was confirmed by betadisper with ANOVA. To isolate the within-subject temporal signal otherwise masked by inter-individual variation, distance-based redundancy analysis (dbRDA; capscale on Bray–Curtis dissimilarities) was performed, constraining on sampling time while conditioning out participant identity. Significance of the time constraint was assessed by a permutation test with permutations blocked within participant (999 permutations).

For Alpha diversity, Shannon diversity and Pielou’s evenness were computed on relative abundances (vegan). Each index was modelled with a linear mixed-effects model (nlme::lme) with fixed effects of meal, time (categorical) and their interaction and a random intercept per participant. Within each meal, each postprandial timepoint was contrasted against the fasted (0 min) baseline using emmeans, with exact Dunnett (multivariate-t) adjustment for multiplicity.

### Reporting summary

Further information on research design is available in the Nature Portfolio Reporting Summary linked to this article.

## Supplementary information

Supplementary Information

Reporting Summary

Description of Additional Supplementary Files

Transparent Peer Review File

Supplementary Dataset 1

## Data Availability

The enteroendocrine hormone and metabolomic data generated in this study have been deposited in Zenodo at: https://doi.org/10.5281/zenodo.20679139. Metagenomic sequencing data is available through the NCBI SRA through project code PRJNA1240928.

## Code availability

Custom codes used in this study are available from https://github.com/Nerdobyte/Diet_Metabolome_Microbiome. The code has been deposited in Code Ocean: Provisional DOI is https://doi.org/10.24433/CO.3003445.v1.
