# Supplementary material for: Effect of dietary chickpea structures on the small intestine metabolome and microbiome: a randomised crossover trial
Source: Nat Commun. 2026 Sep 15;17:10840. doi: 10.1038/s41467-026-77714-7 (PMC13672355; doi:10.1038/s41467-026-77714-7)

## Supplementary Text 1. Meal preparation

Meal Preparation

**Meals:** Chickpeas were the main ingredient of the test meals. Whole chickpeas Cicer arietinum L., Kabuli type, Argentine variety (7mm diameter) were supplied by AGT Poortman Ltd. These were dehulled through an abrasive process by holding 200 g batches of dry chickpea (as supplied) in a Steelbrush dehuller (Westrup pre-cleaning debranner, coarse blue roll) for 30s, then sieved on a 3mm grid to exclude fragments of testa. The resulting dehulled chickpea material was weighed out into 75.0 g portions (by a researcher independent to the study), sealed and labelled. These dehulled chickpea portions were the main ingredient for the 'broken cell' (Broken) and 'intact separated cell' (Intact-S) chickpea meals, which were freshly prepared on the morning of each study visit using standardised processing protocols to deliver different structures. For the 'cell cluster' (Intact-C) meal, the dehulled chickpeas were further processed by milling to obtain intact tissue particles (1.6-2.0 mm) containing whole cells, before portioning out. These coarse particles were obtained by trickle feeding the dehulled chickpeas through a Buhler miag Vario mill set up with a Roll Gap of 1.8 mm and Roller speed 220:450 rpm on 24.5 mm diameter break rolls 5 flutes/cm break rolls in Sharp-Sharp disposition for the first break. Output material was analytically sieved to collect target size fraction (≥ 1.6 and < 2.0 mm), and the oversize material (≥ 2.0 mm) was then re-milled according to the same setting as used for the first break, but with a smaller Roll Gap (0.8 mm), to increase recovery of the target size fraction. This coarsely milled chickpea material (≥1.6 and <2.0 mm 'semolina') was the main ingredient for the 'cell-cluster' meal, and was weighed into 75 g portions in preparation for the study visits.

Each chickpea ingredient portion was then processed further, as described below, using a Thermomix® TM5 (Vorwerk) food processor to deliver freshly cooked meals with different structures to the study participants.

For the 'Broken cell/BC' and 'Separated cells/SC' meal types, the pre-weighed 75 g portion of de-hulled chickpeas was rinsed with drinking water from the tap in a colander until the water ran clear, and then immersed in 1L tap water under cover overnight for ~16 h. The following morning, the soaked chickpeas were then drained, rinsed briefly with fresh tap water, and drained thoroughly before transferring to the Thermomix. For the BC meal, additional tap water was then added to the soaked chickpea to achieve a total weight of 637.5 g. This mixture was then blended (forward direction, speed setting 10) for 3 min at 37 °C, which breaks the chickpea cotyledon cells, before further heating and cooking at 95 °C for 28 min, with gentle stirring (blender in reverse setting, speed setting 4). A portion (~490 g) of the cooked mixture was then weighed into a bowl and seasoned with a 115 g pot of jelly (Hartleys' no added sugar raspberry jelly) and 15 g of sugar-free jam (Stutes no added sugar blackcurrant jam), to aid palatability, and served with drinking water (~270 mL) and served immediately to participants while still warm (above 60 °C).

The SC meal, additional tap water was added after the overnight soak to achieve a total weight of 375.0 g, and then covered, heated and cooked at 95 °C for 80 min, with gentle stirring (blender in reverse direction, speed setting 5). A portion (~280 g) of the cooked mixture was then weighed into a bowl, seasoned as described above, and served immediately while still warm, together with drinking water (~480 mL).

For the 'Clustered cells/CC' meal type, the soaked semolina were drained and transferred into the Thermomix together with additional tap water to achieve a total wet weight of 300 g. This was then cooked for 50 min at 95 °C with gentle stirring (reverse, speed setting 1). A serving of ~120 g drained cooked chickpea solids was then transferred into a bowl, seasoned as above, and served immediately while warm together with drinking water.

For all meals, the volume of water served in the drinking glass was adjusted based on the cooked meal weight, so that all meals provided 30 g total starch from chickpea, the same amount of seasoning and similar total mass (~760 g).

**Supplementary Table 1. Inclusion and exclusion criteria for participants**

| Inclusion Criteria | Exclusion Criteria |
|---|---|
| Male or female | Abnormal ECG |
| Age between 18-65 years (inclusive) | Screening blood results outside of normal reference values. |
| | Fasting glucose 3.9-5.6 mmol/L |
| | Haemoglobin M125-170/ F114-150 g/L |
| | White blood cell count 4.2-11.2 x 109/L |
| | Red blood cell count 3.73-4.96 million/mcL |
| Body mass index (BMI) of 18-30 kg/m2 | Weight change of ≥ 5kg in the preceding 2 months |
| Willingness and ability to give written informed consent and willingness and ability to understand, to participate and to comply with the study requirements | Current Smokers |
| | History of substance abuse and/or excess alcohol intake |
| | Cancer |
| | Gastrointestinal disease e.g., inflammatory bowel disease or irritable bowel syndrome, kidney disease, liver disease and pancreatitis |
| | Pregnancy |
| | Diabetes or cardiovascular disease |
| | Participant in a research study or blood donation 12 weeks prior |
| | Started new medication within the last 3 months likely to interfere with glucose and energy metabolism, appetite regulation and hormonal balance, including: anti-inflammatory drugs or steroids, antibiotics, androgens, phenytoin, erythromycin, or thyroid hormones. |

**Supplementary Table 2. Baseline characteristics for participants**

| Variable | Baseline measurements |
|---|---|
| Gender (M:F) | 6:4 |
| Age (years) | 30.8 ± 2.411 |
| Height (m) | 1.70 ± 0.021 |
| Weight (kg) | 72.07 ± 3.201 |
| BMI (kg/m2) | 24.91 ± 0.821 |
| Body Fat (%) | 17.35 ± 1.911 |

Results presented as Mean ± SEM

## Supplementary Figure

### Supplementary Figure 1. Summary of study protocol and sample collection.

Created in BioRender. Cai, M. (2026) https://BioRender.com/vkw7mc4

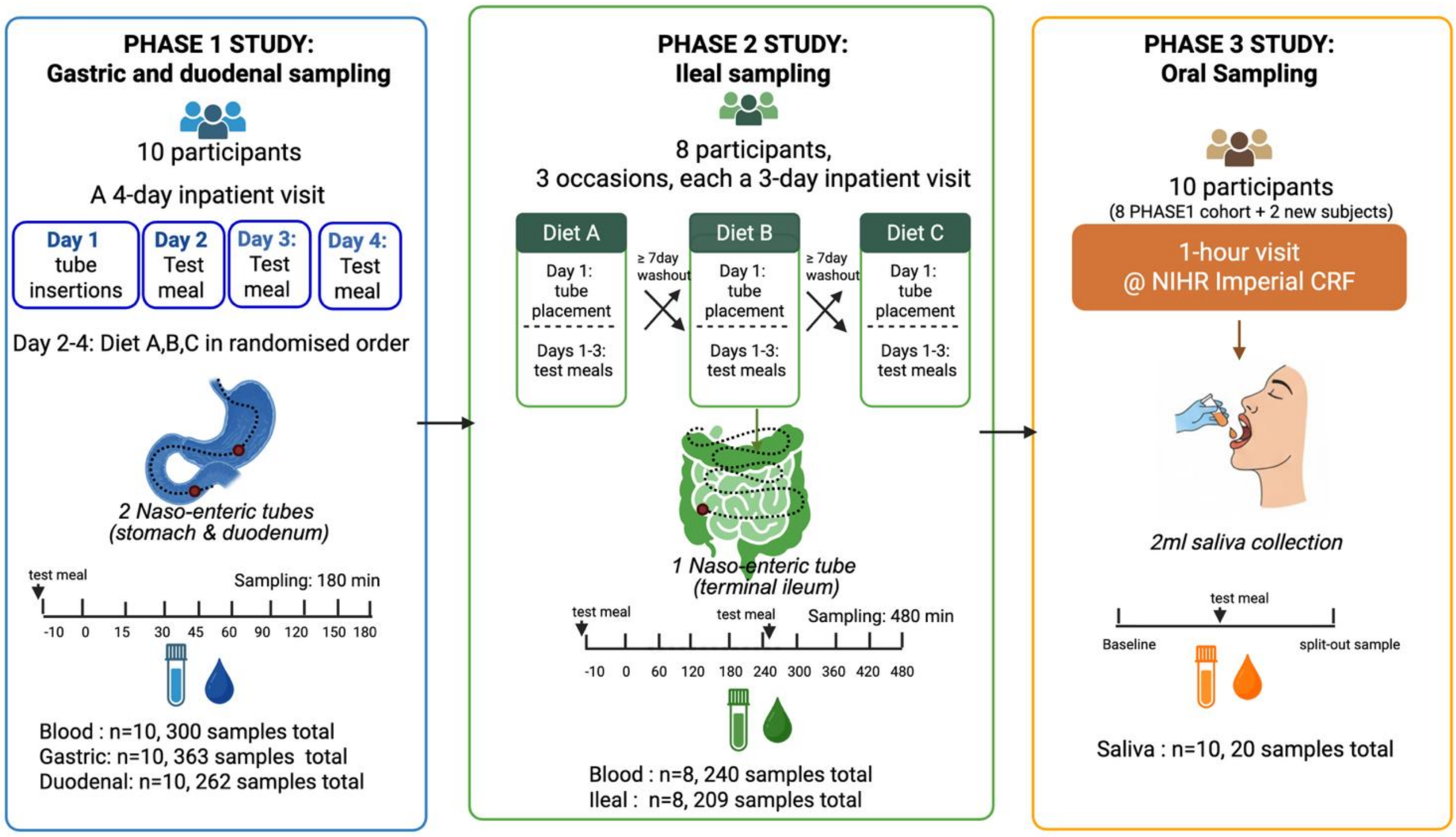

**Supplementary Figure 2. CONSORT flow diagram illustrating the study procedures.**

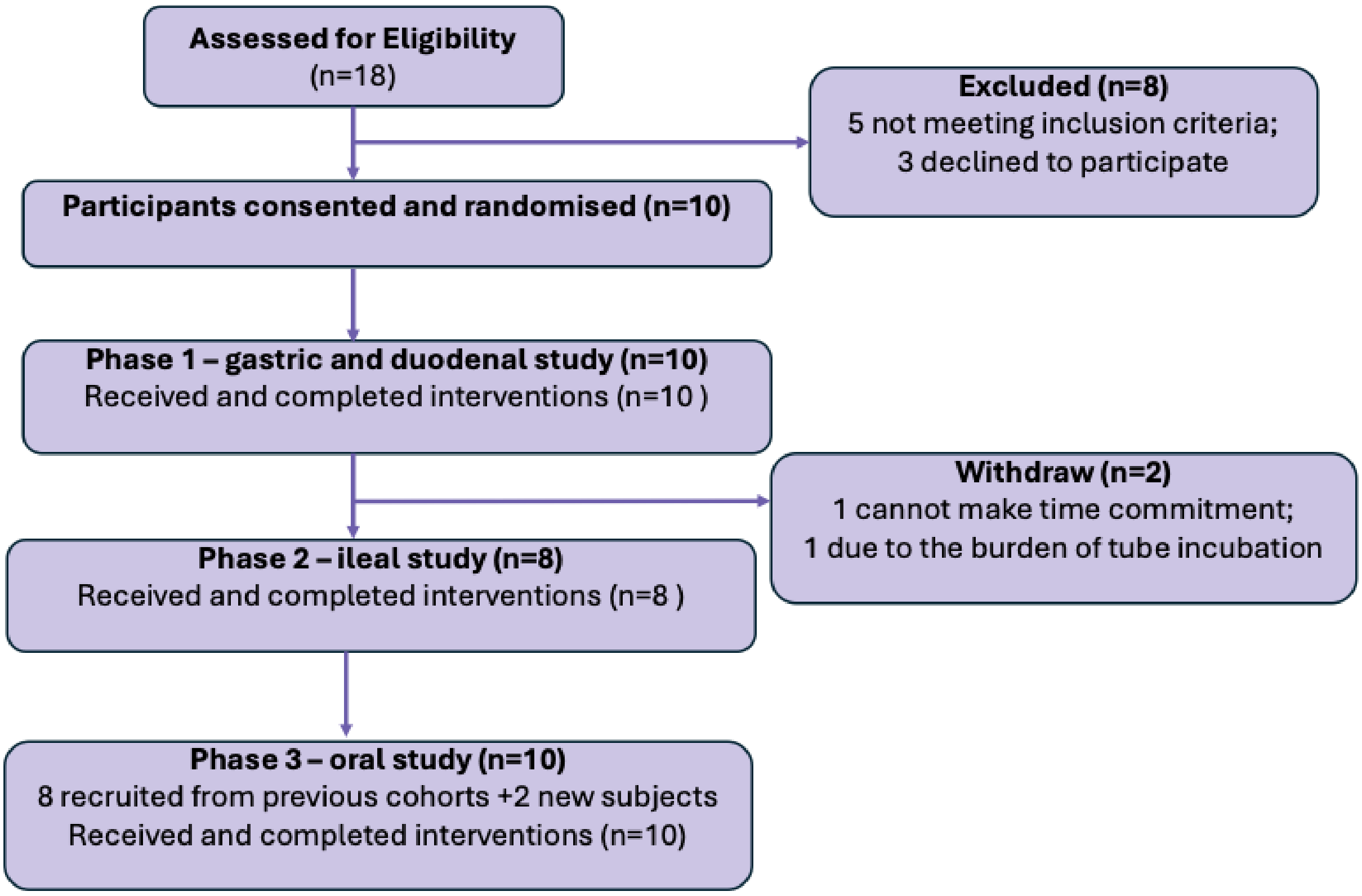

**Supplementary Figure 3: 3D graph tracking of metabolite concentrations across 3 different diets along the GI-tract.** Diet BC (pink) corresponds to broken cells, diet SC (blue) corresponds to single cells and diet CC (green) corresponds to cell clusters. Z-axis shows samples in the following order: three gastric samples, three duodenal samples, and ileal samples. Inset blue box (dashed) within sugars are chickpea markers.

**(a)**

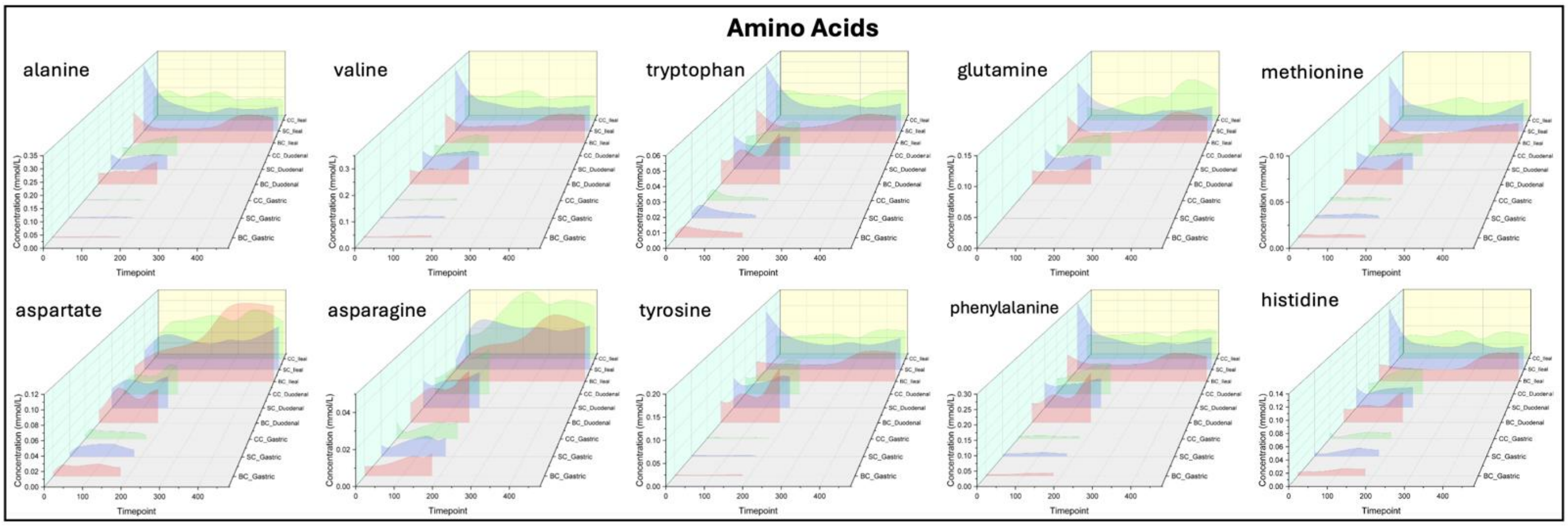


**(b)**

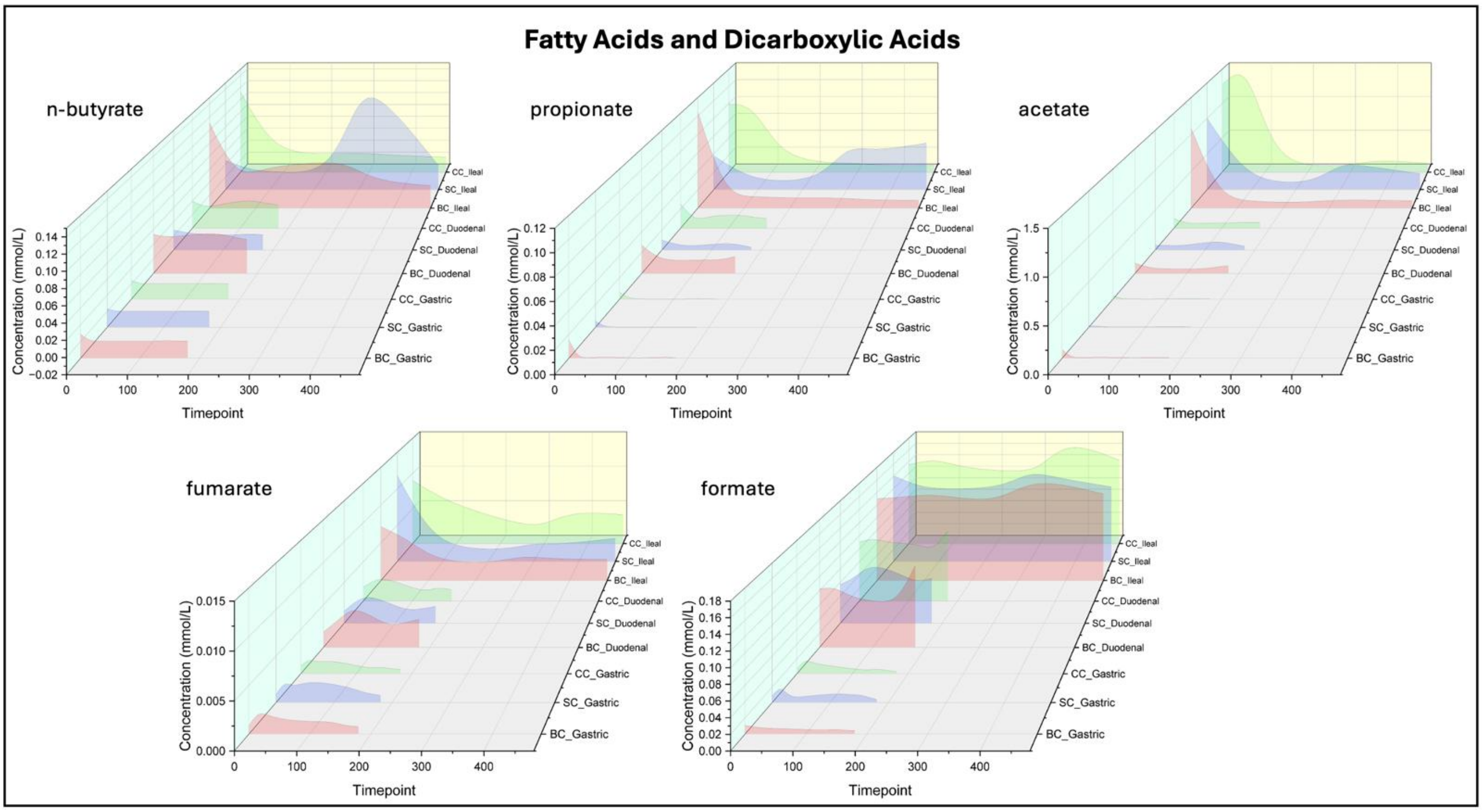


**(c)**

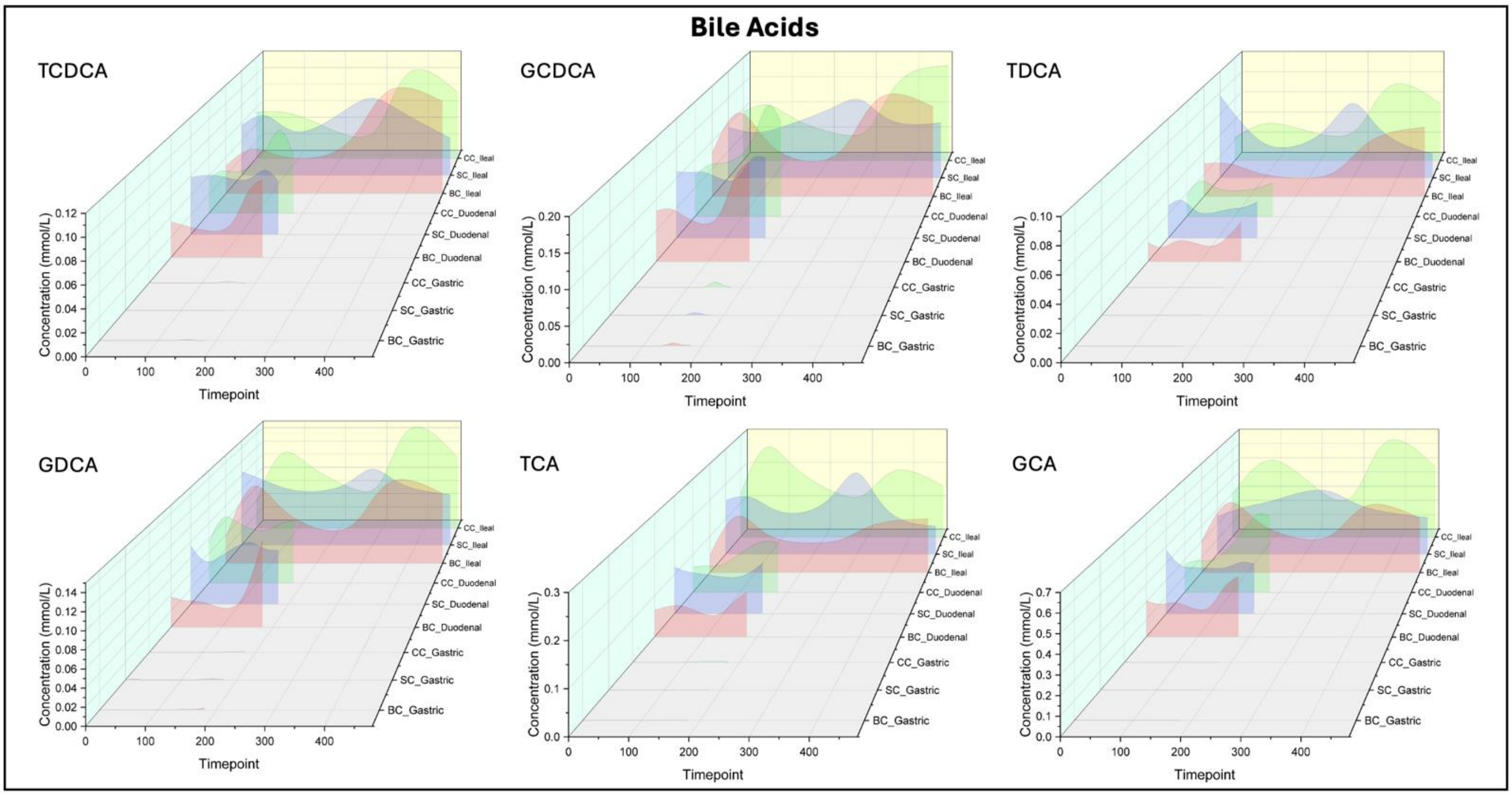
Bile Acids
TCDCA
GCDCA
TDCA
GDCA
TCA
GCA
Concentration (mmol/L)
Timepoint
0
100
200
300
400
CC_Ileal
SC_Ileal
BC_Ileal
CC_Duodenal
SC_Duodenal
BC_Duodenal
CC_Gastric
SC_Gastric
BC_Gastric

(d)

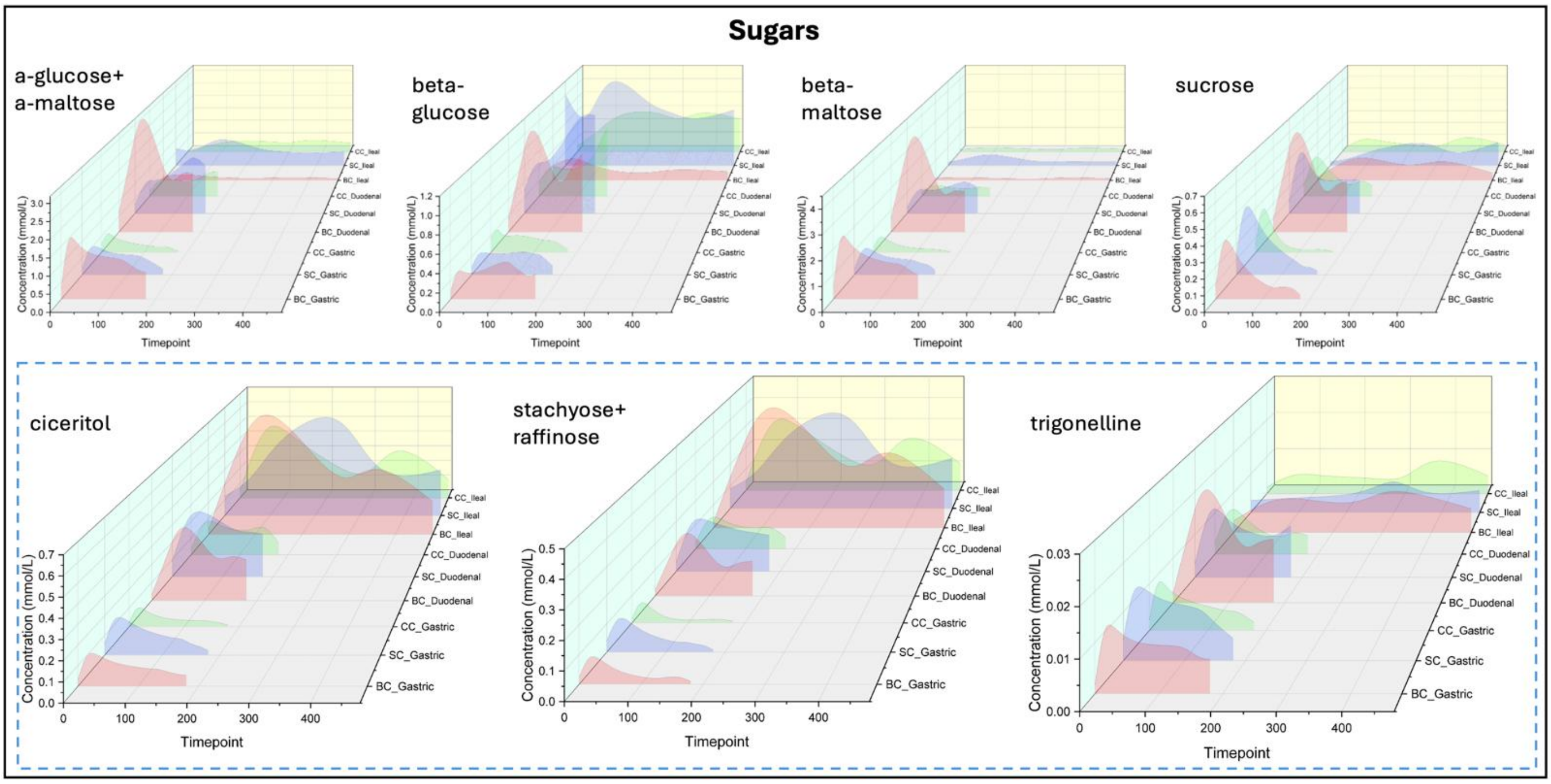
Sugars
a-glucose+
a-maltose
beta-
glucose
beta-
maltose
sucrose
ciceritol
stachyose+
raffinose
trigonelline
Concentration (mmol/L)
Timepoint
0
100
200
300
400
CC_Ileal
SC_Ileal
BC_Ileal
CC_Duodenal
SC_Duodenal
BC_Duodenal
CC_Gastric
SC_Gastric
BC_Gastric

**Supplementary Figure 4: Per individual metabolic concentration in mmol/L plotted against time after meal consumption, within gastric samples.** Green curves are diet BC, Red curves are diet CC, Black curves are diet SC.

(a)

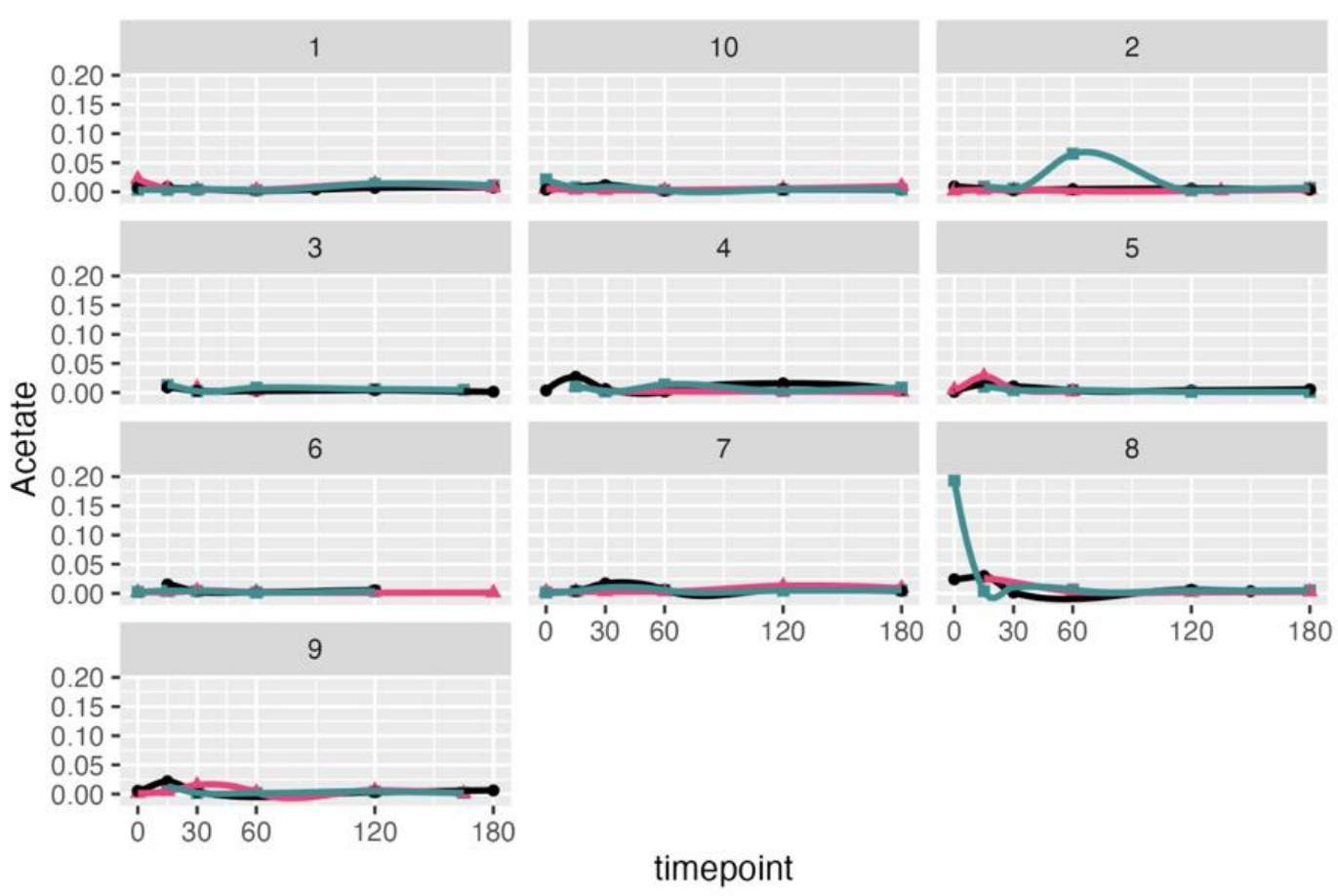


(b)

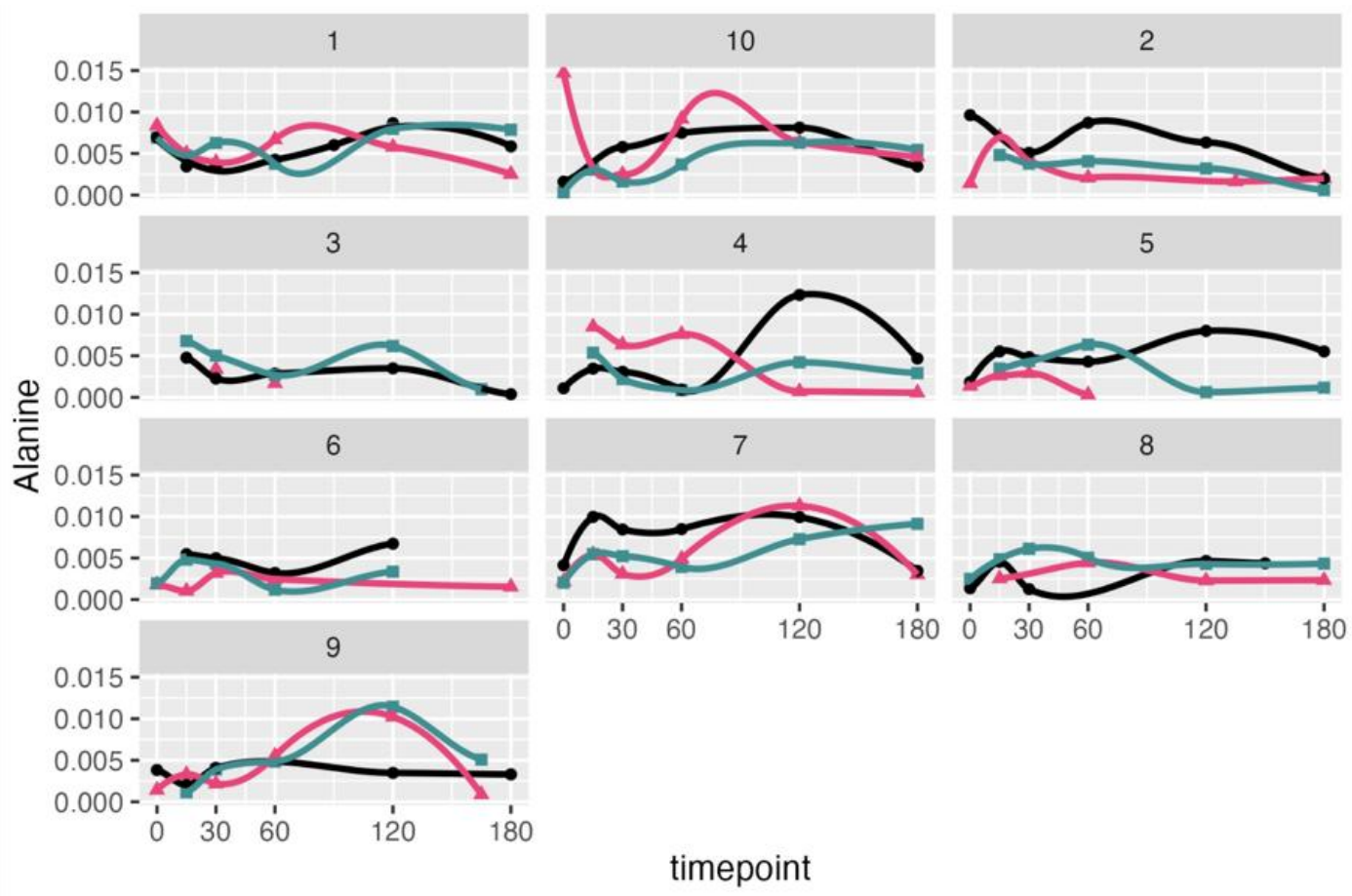


(c)

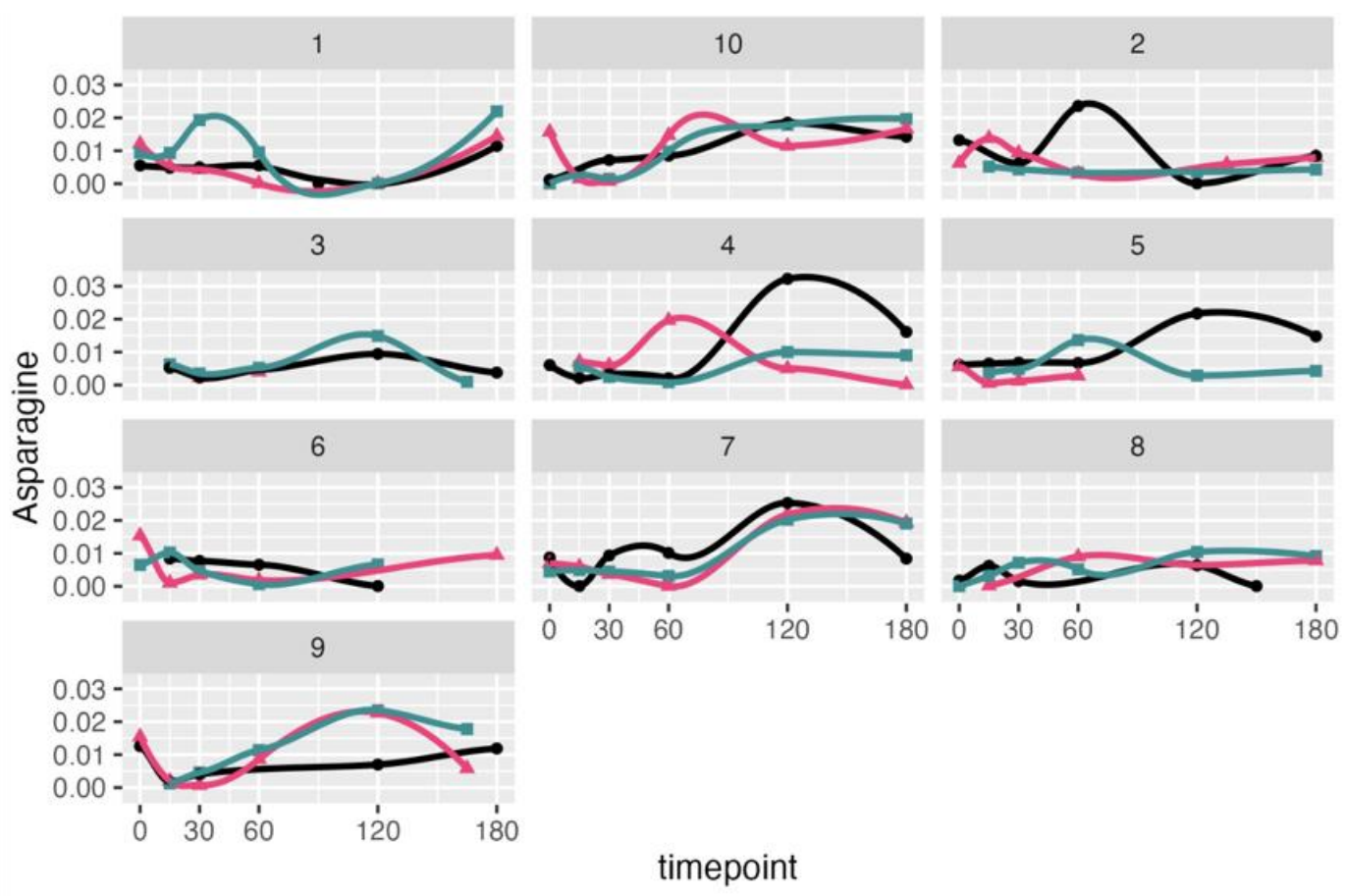


(d)

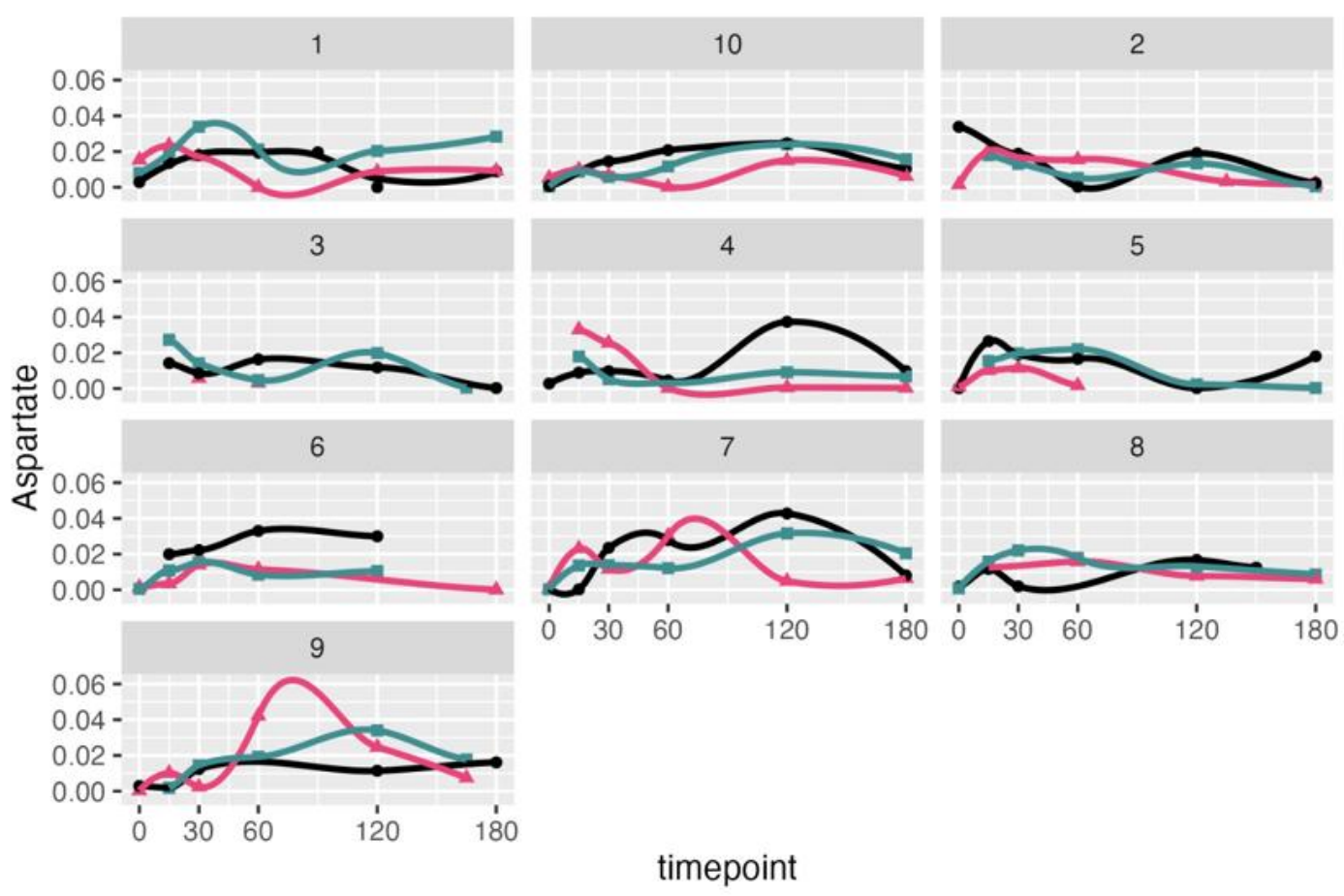


(e)

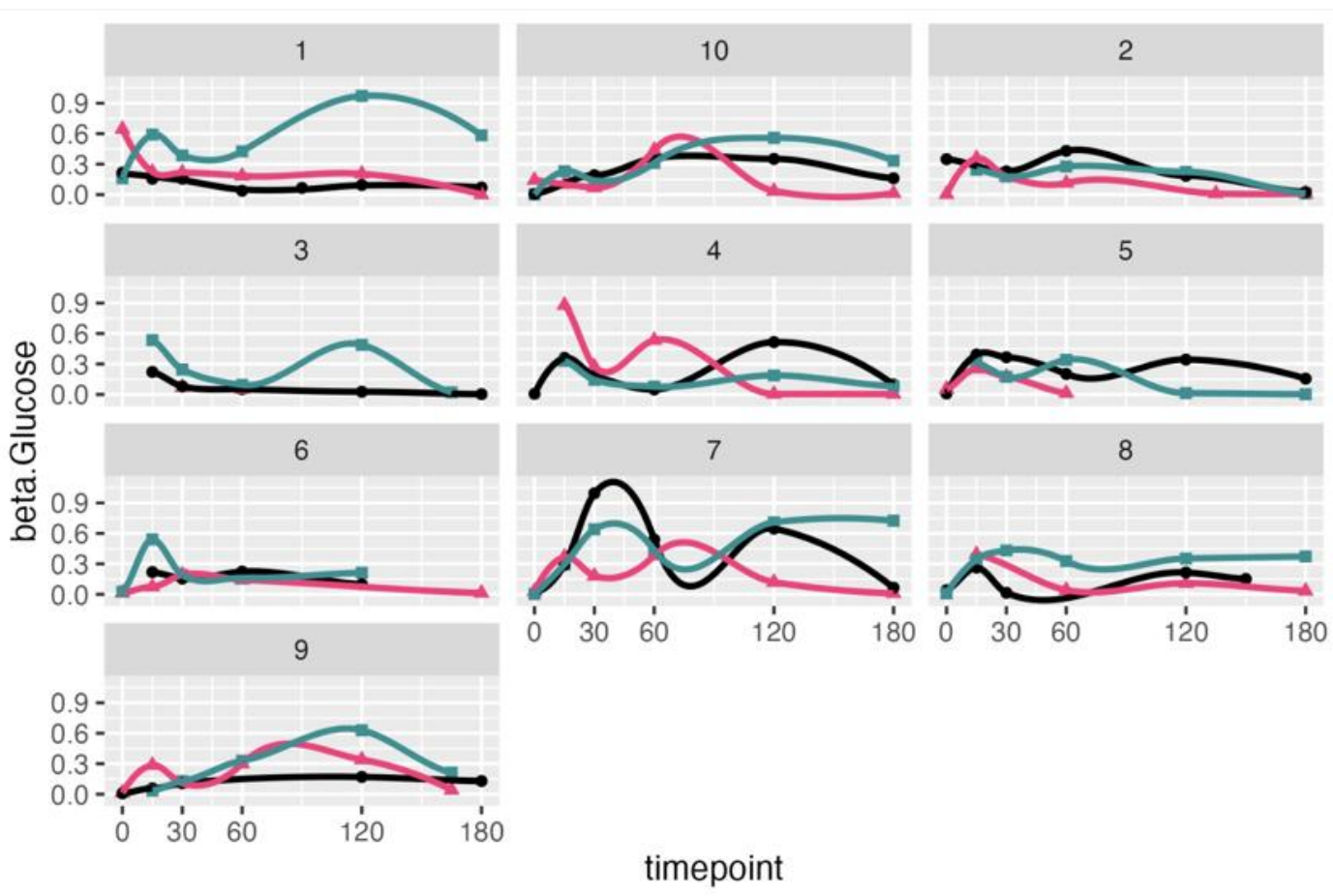


(f)

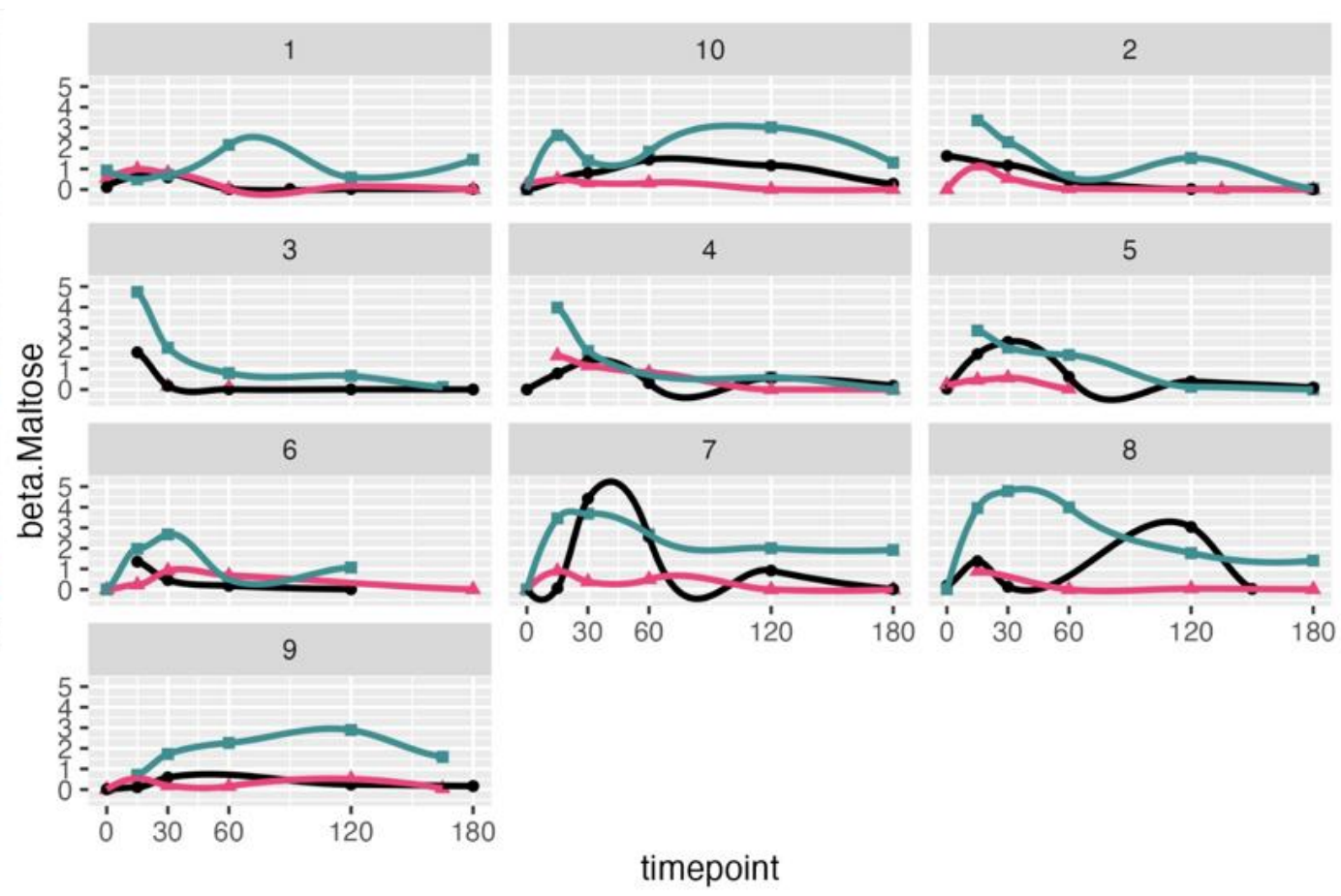

(g)

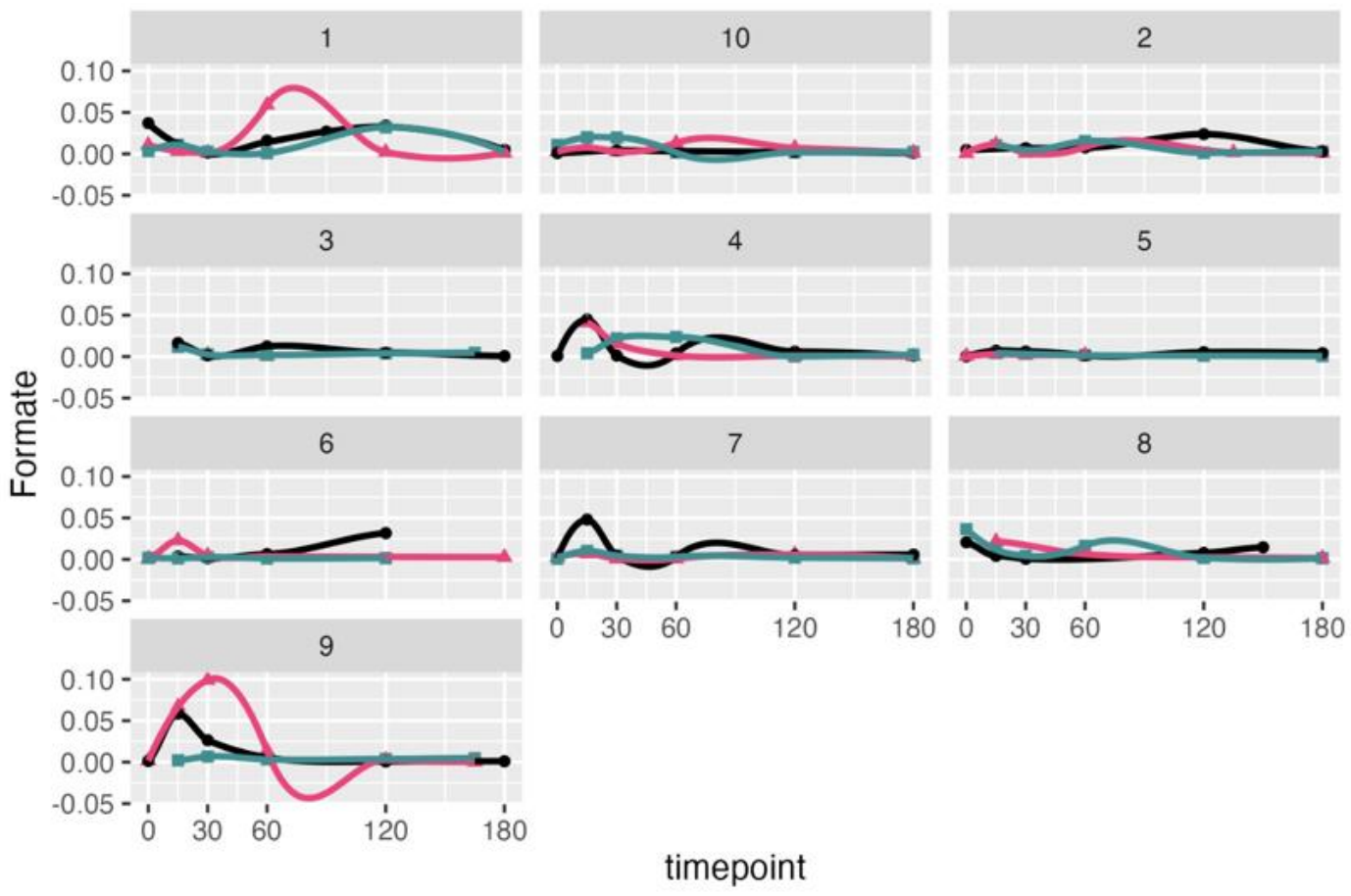
Formate
1
10
2
3
4
5
6
7
8
9
0.10
0.05
0.00
-0.05
0
30
60
120
180
timepoint

(h)

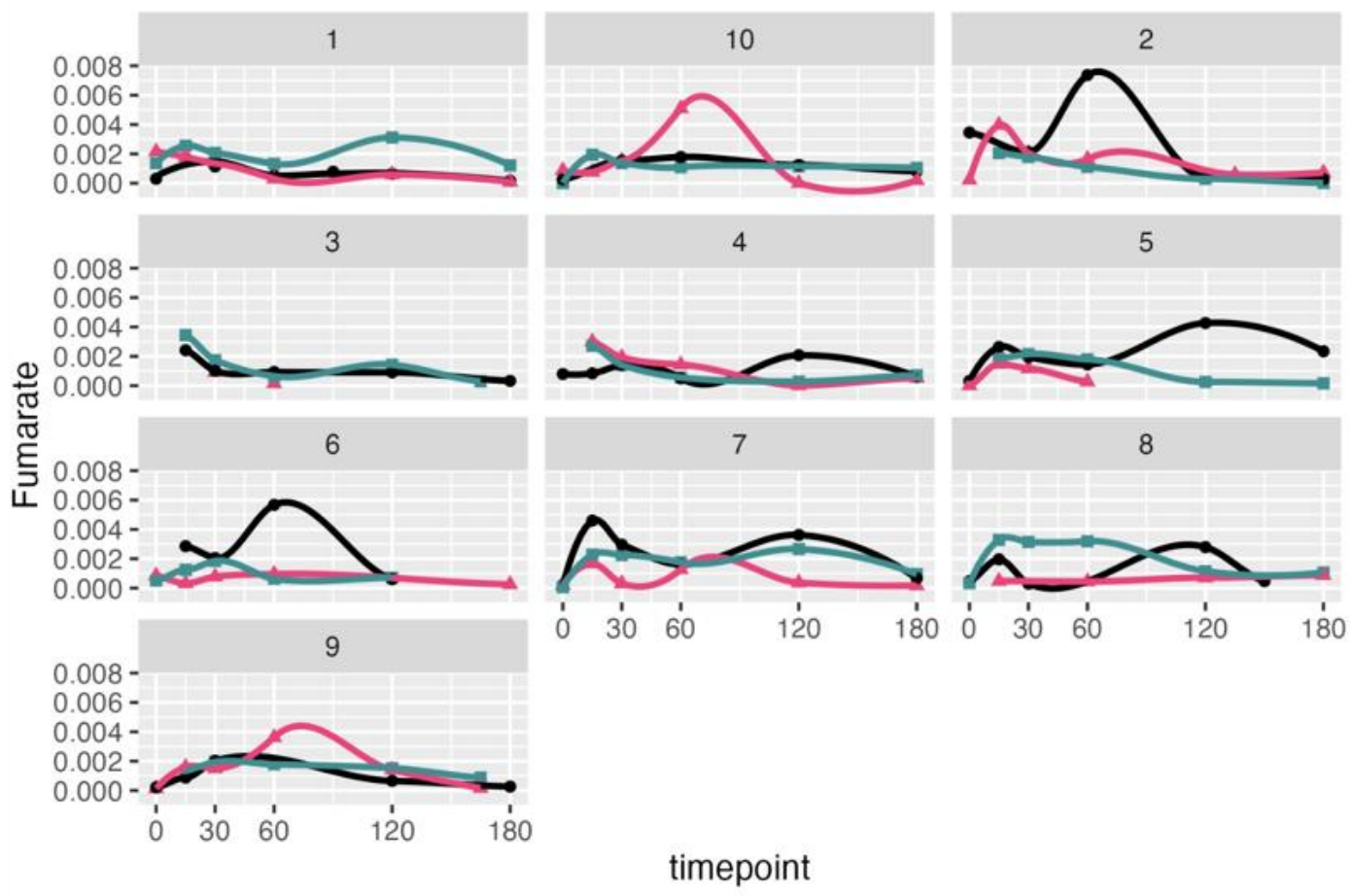
Fumarate
1
10
2
3
4
5
6
7
8
9
0.008
0.006
0.004
0.002
0.000
0
30
60
120
180
timepoint

(i)

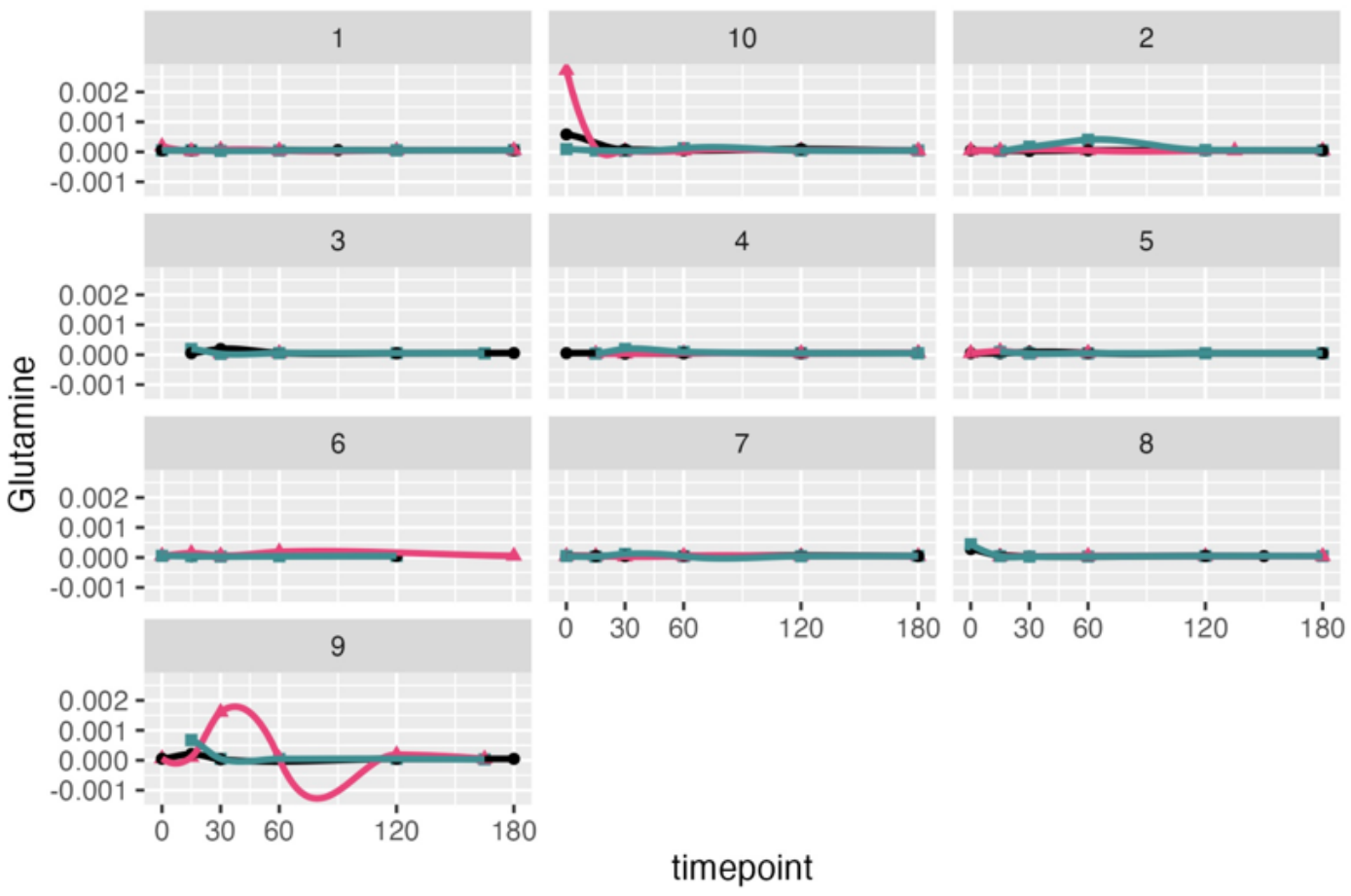
Glutamine
1
10
2
3
4
5
6
7
8
9
0.002
0.001
0.000
-0.001
0
30
60
120
180
timepoint

(j)

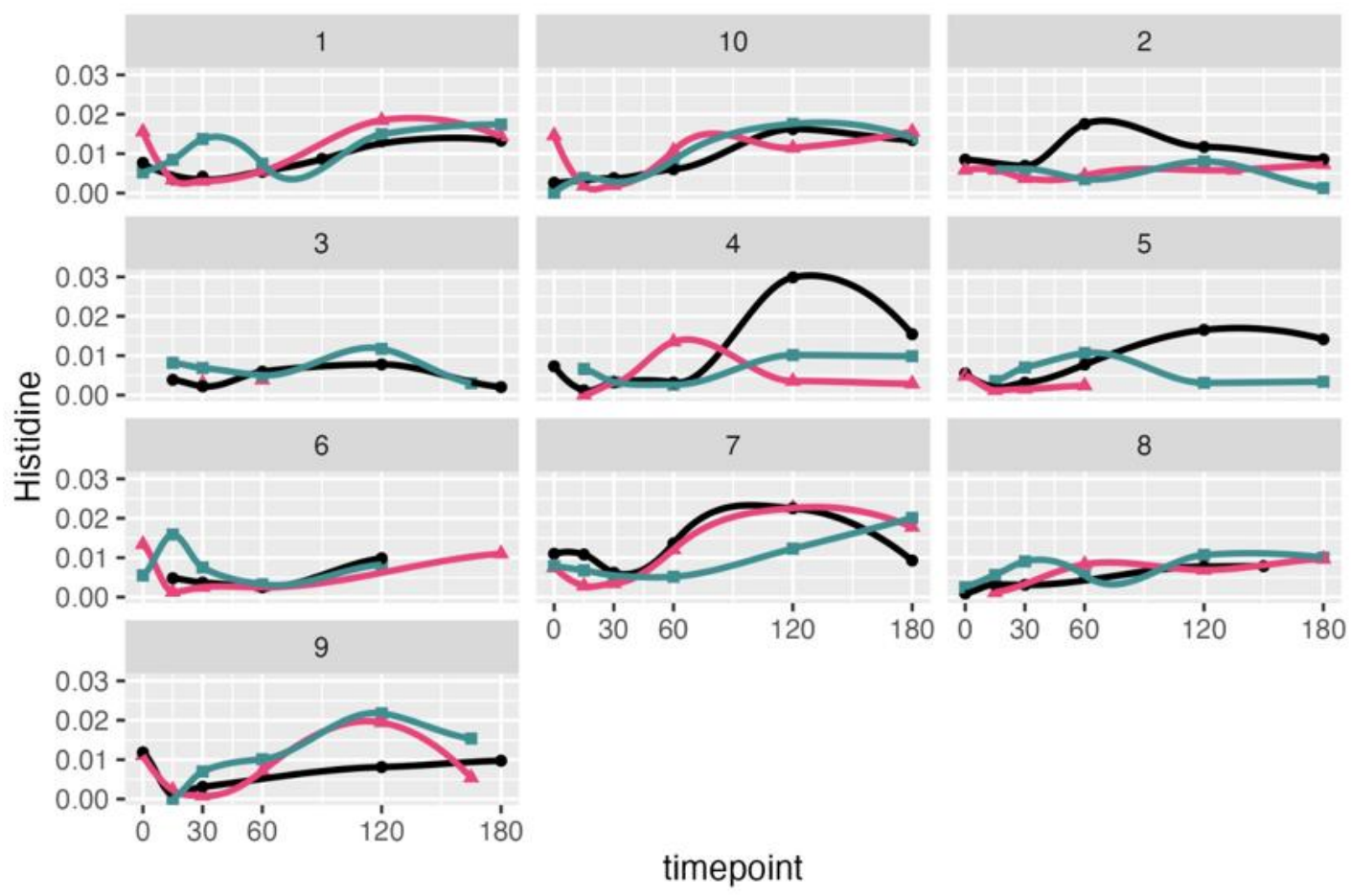
Histidine
1
10
2
3
4
5
6
7
8
9
0.03
0.02
0.01
0.00
0
30
60
120
180
timepoint

(k)

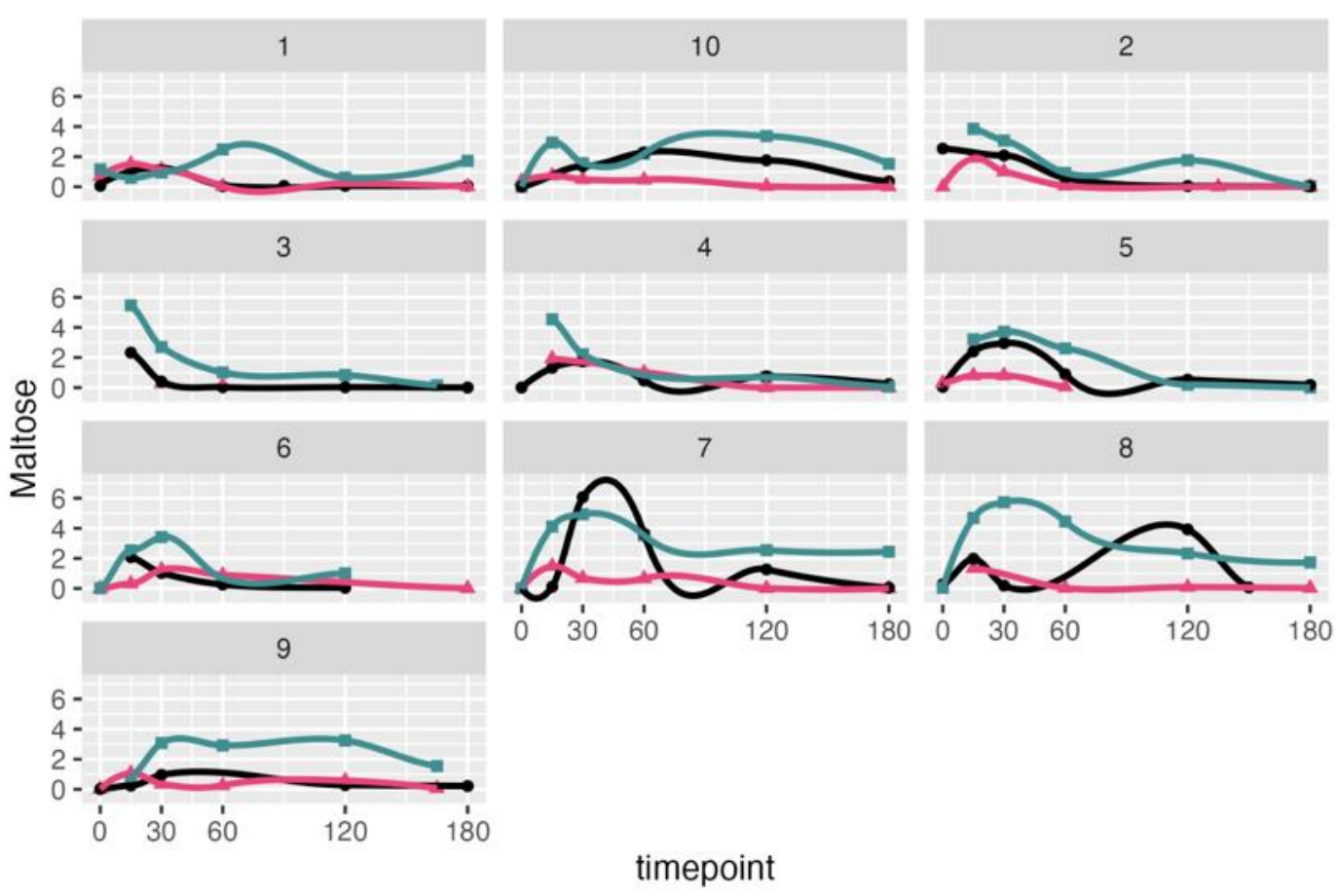
Maltose
1
10
2
3
4
5
6
7
8
9
6
4
2
0
0
30
60
120
180
timepoint

(l)

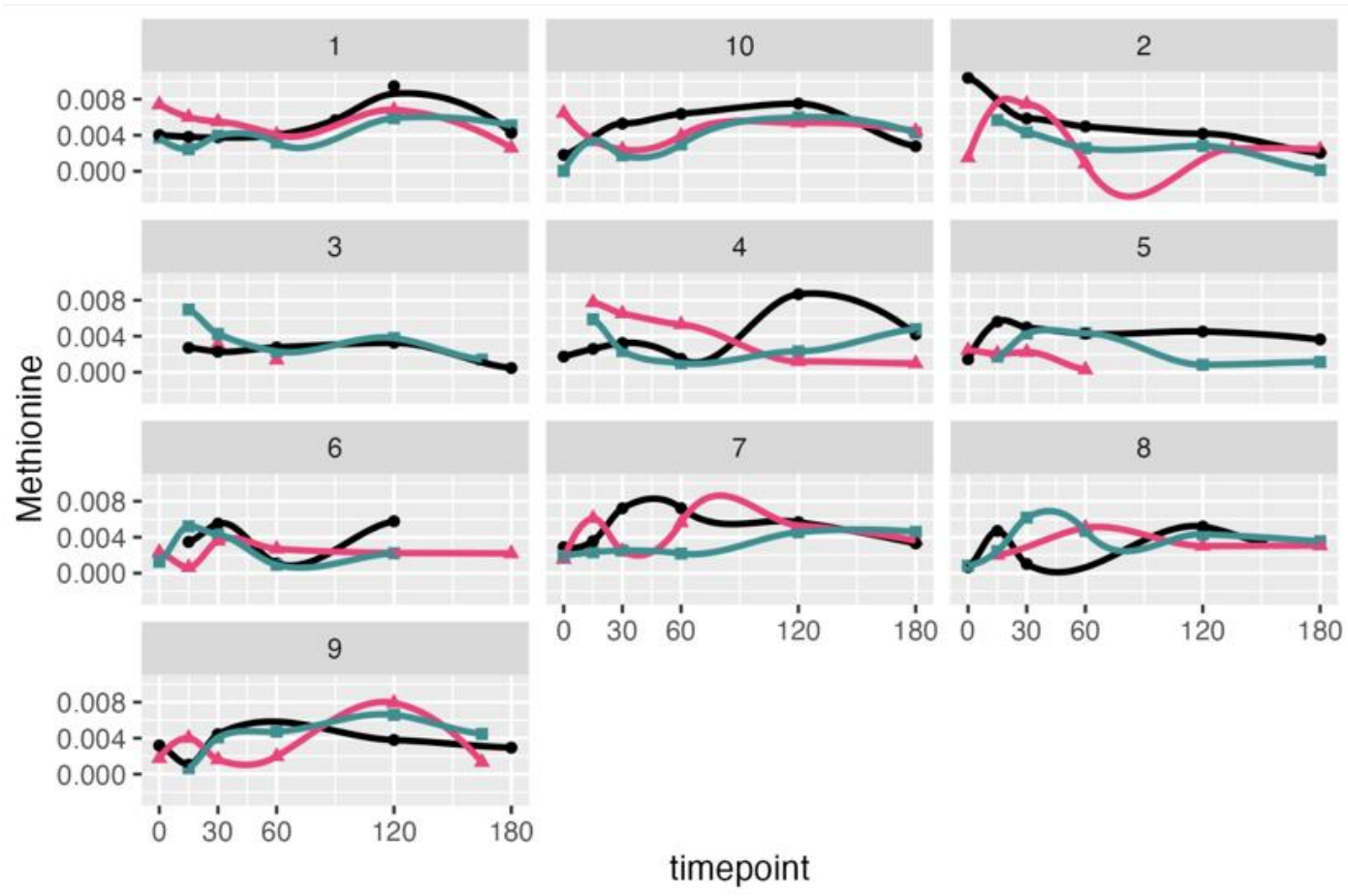
Methionine
1
10
2
3
4
5
6
7
8
9
0.008
0.004
0.000
0
30
60
120
180
timepoint

(m)

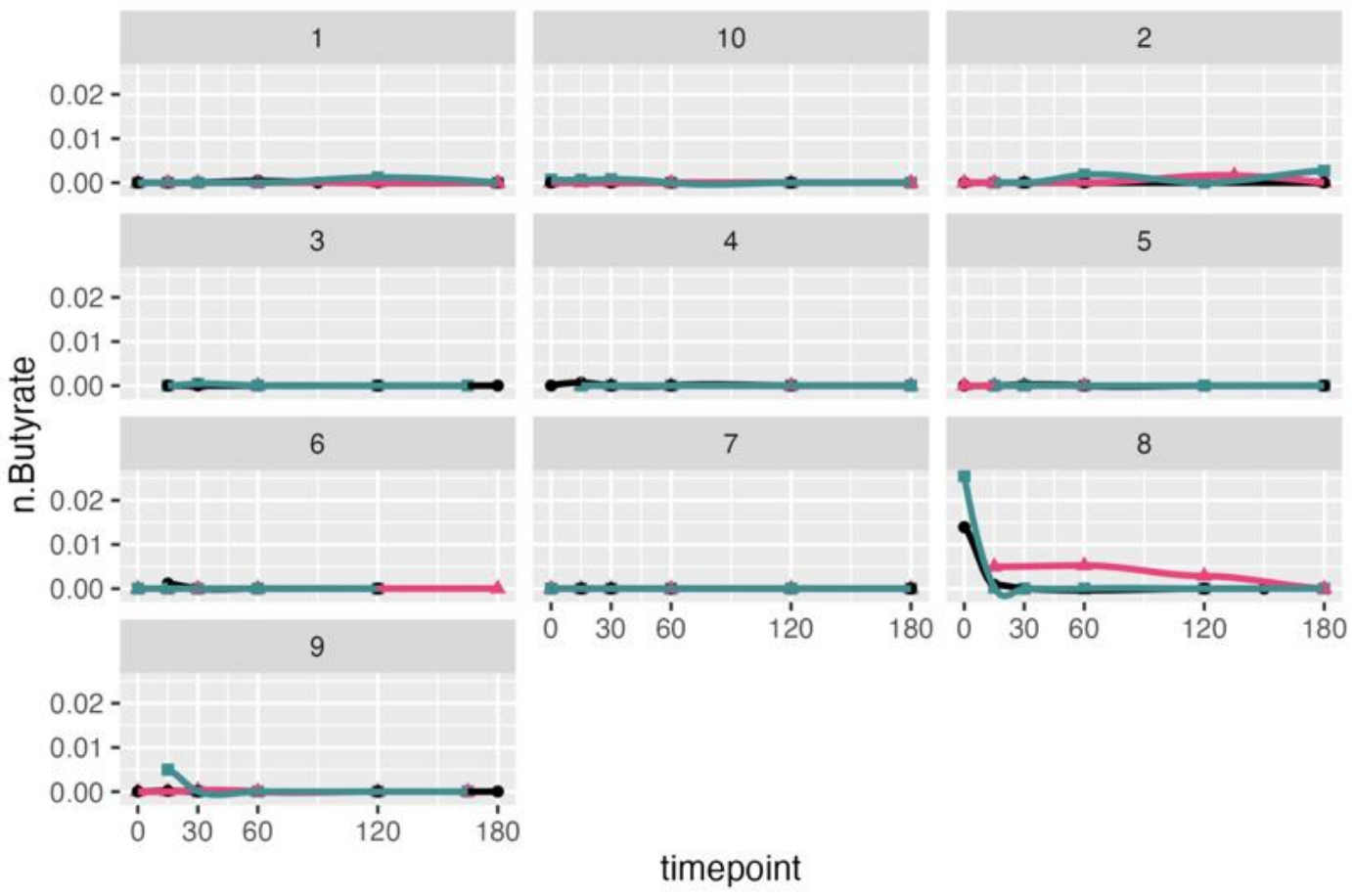

1
10
2
3
4
5
6
7
8
9
n.Butyrate
0.02
0.01
0.00
0 30 60 120 180
timepoint


(n)

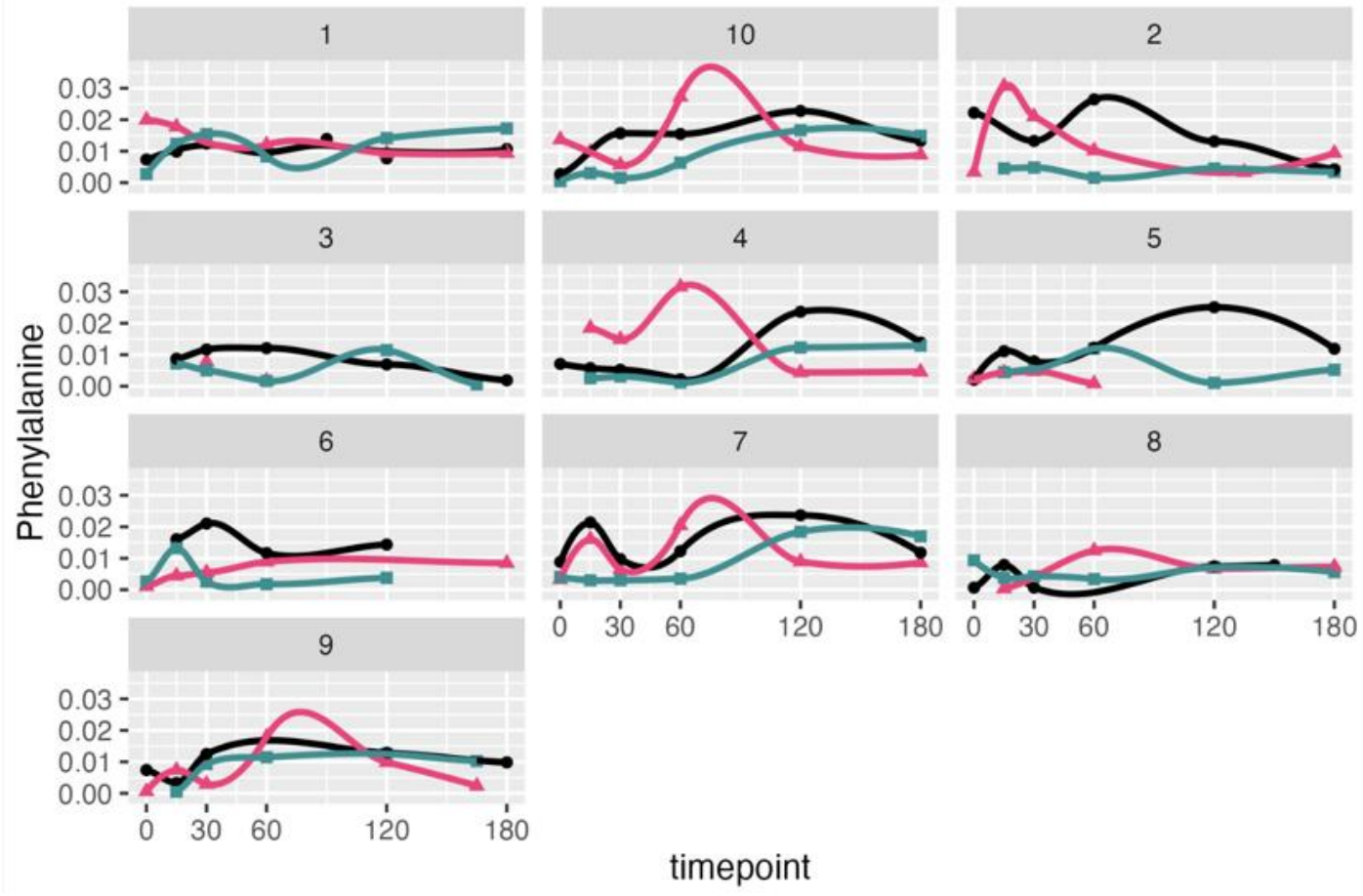

1
10
2
3
4
5
6
7
8
9
Phenylalanine
0.03
0.02
0.01
0.00
0 30 60 120 180
timepoint


(o)

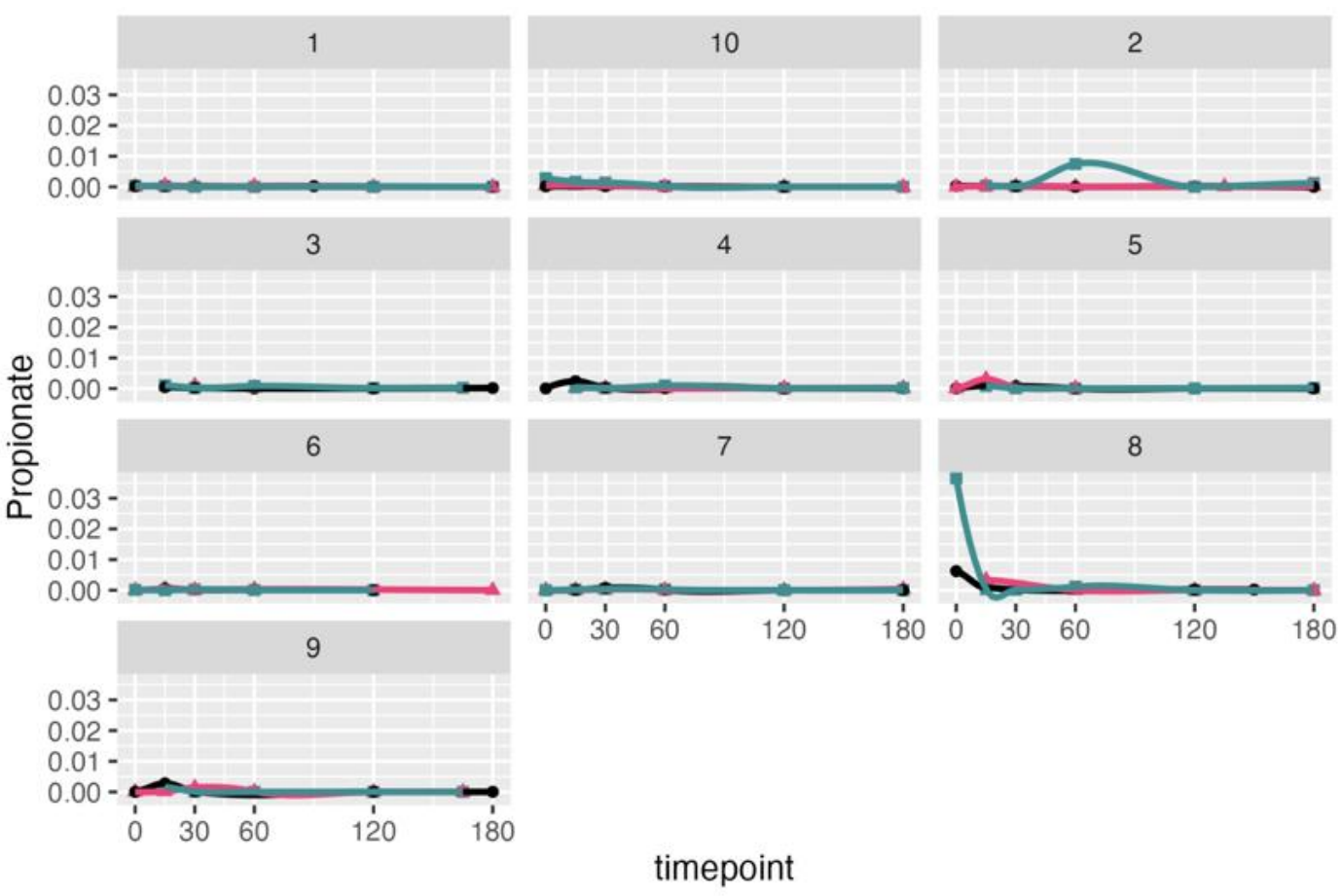

1
10
2
3
4
5
6
7
8
9
Propionate
0.03
0.02
0.01
0.00
0 30 60 120 180
timepoint


(p)

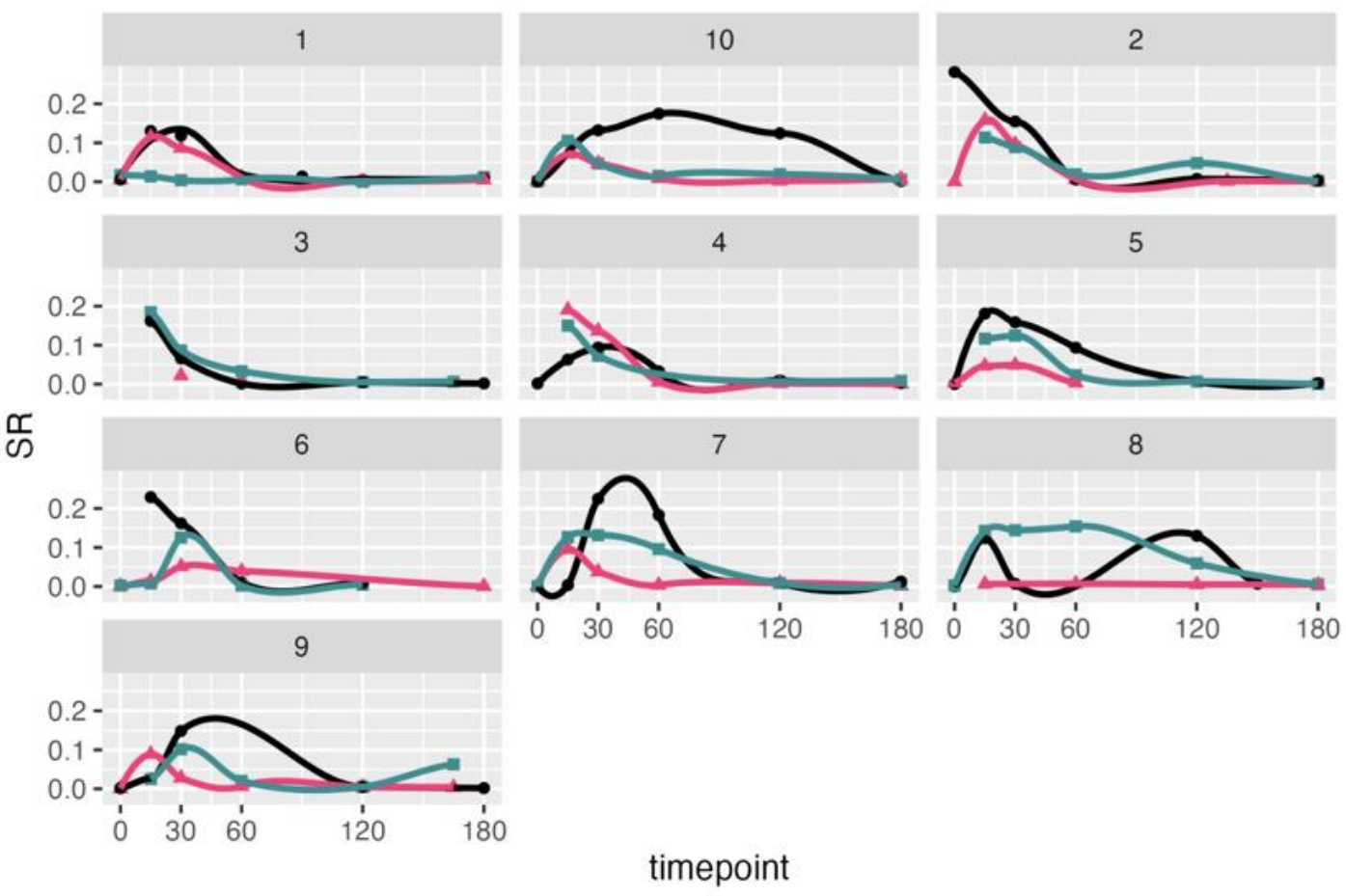

1
10
2
3
4
5
6
7
8
9
SR
0.2
0.1
0.0
0 30 60 120 180
timepoint


(q)

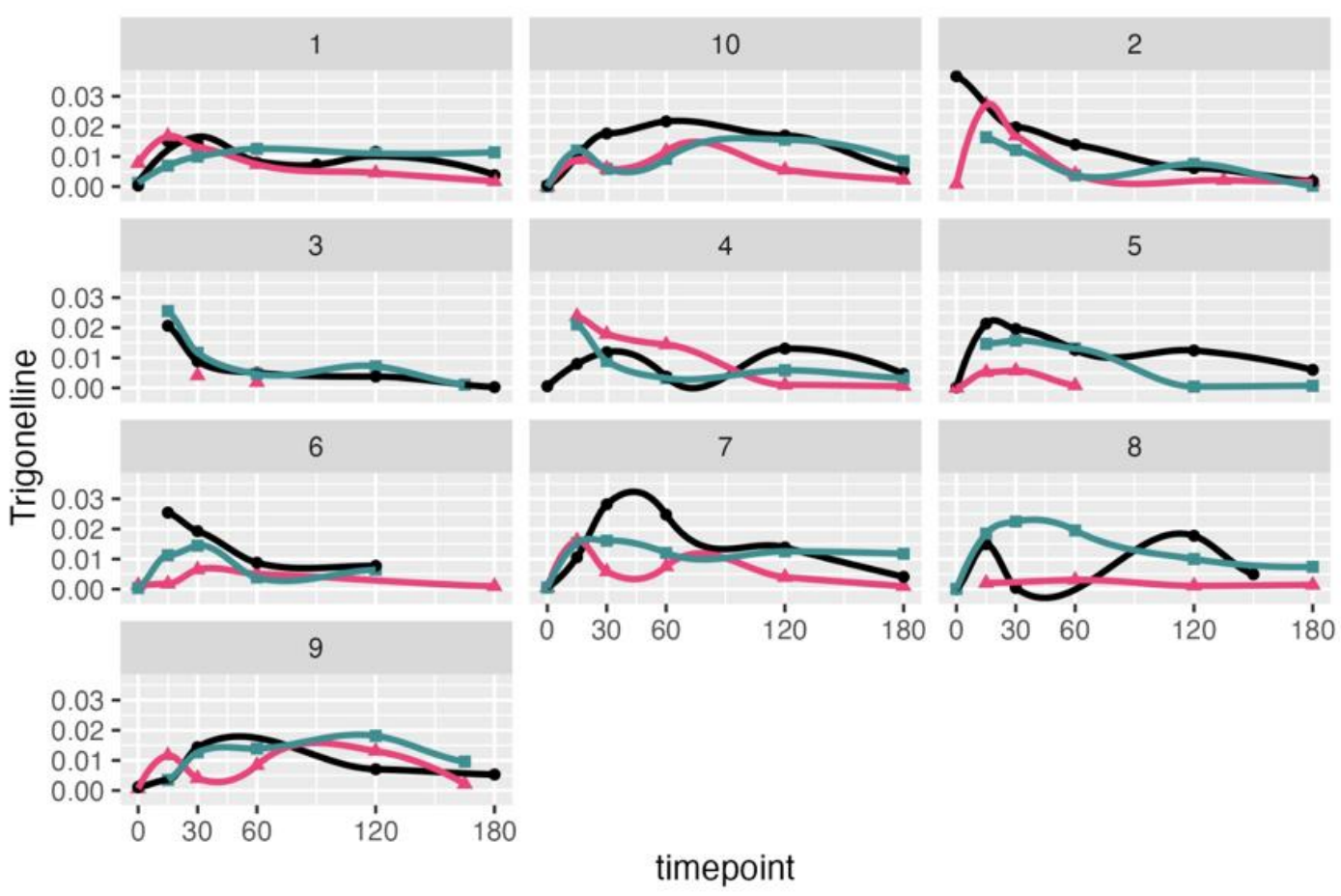

1
10
2
3
4
5
6
7
8
9
Trigonelline
0.03
0.02
0.01
0.00
0 30 60 120 180
timepoint


(r)

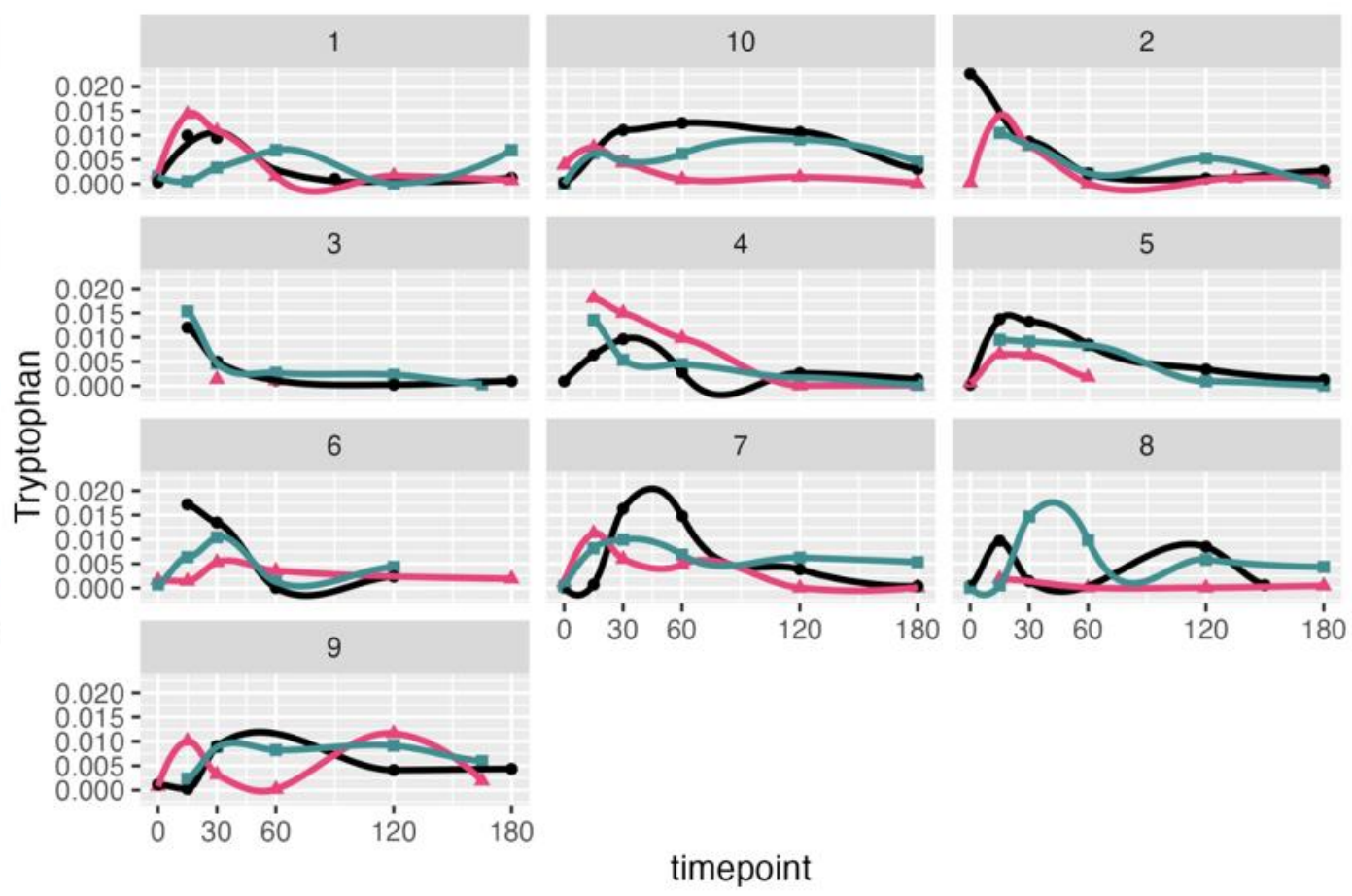

1
10
2
3
4
5
6
7
8
9
Tryptophan
0.020
0.015
0.010
0.005
0.000
0 30 60 120 180
timepoint

**(s)**

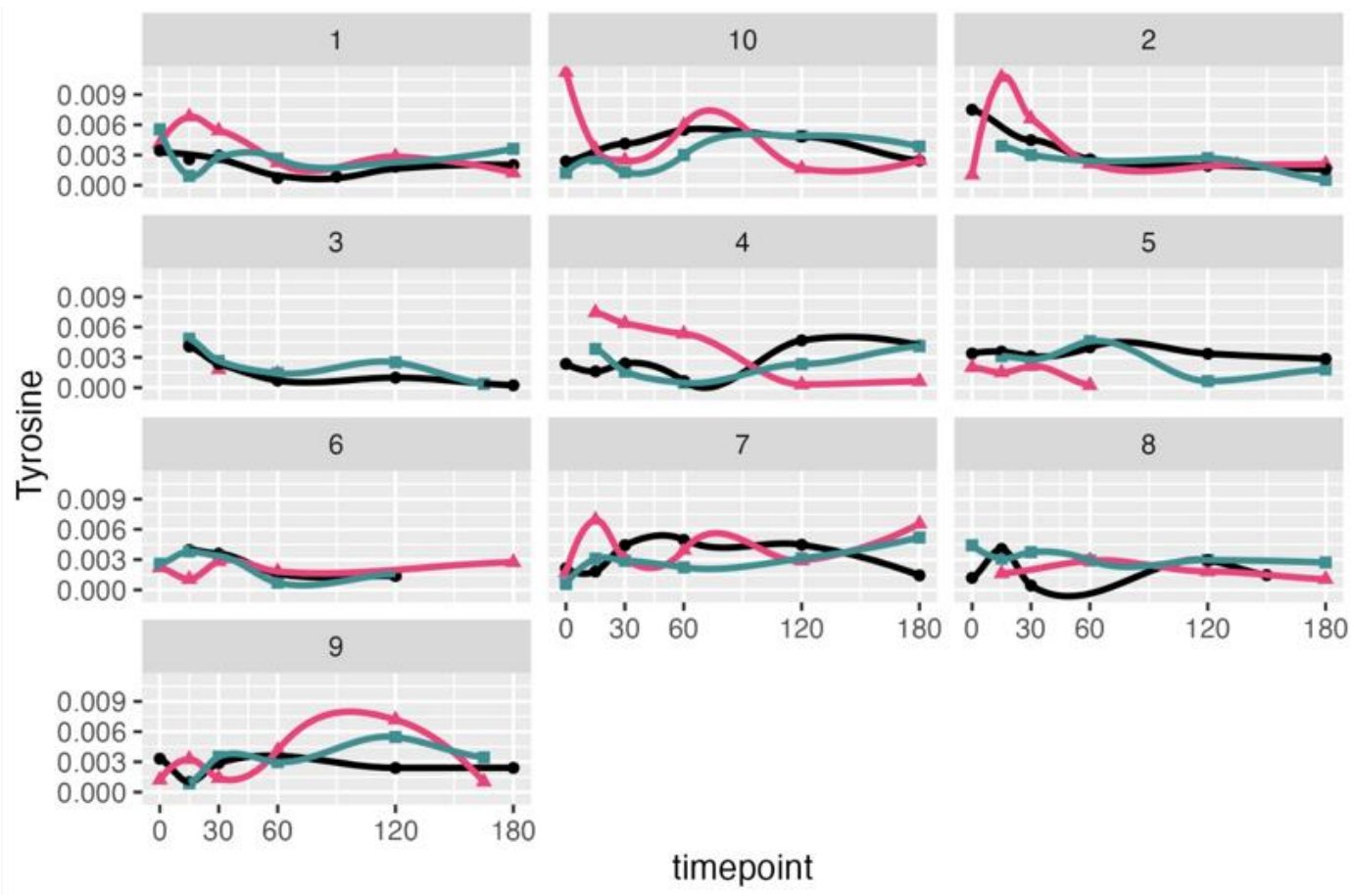

Tyrosine
timepoint
1
10
2
3
4
5
6
7
8
9
0.009
0.006
0.003
0.000
0
30
60
120
180


**(t)**

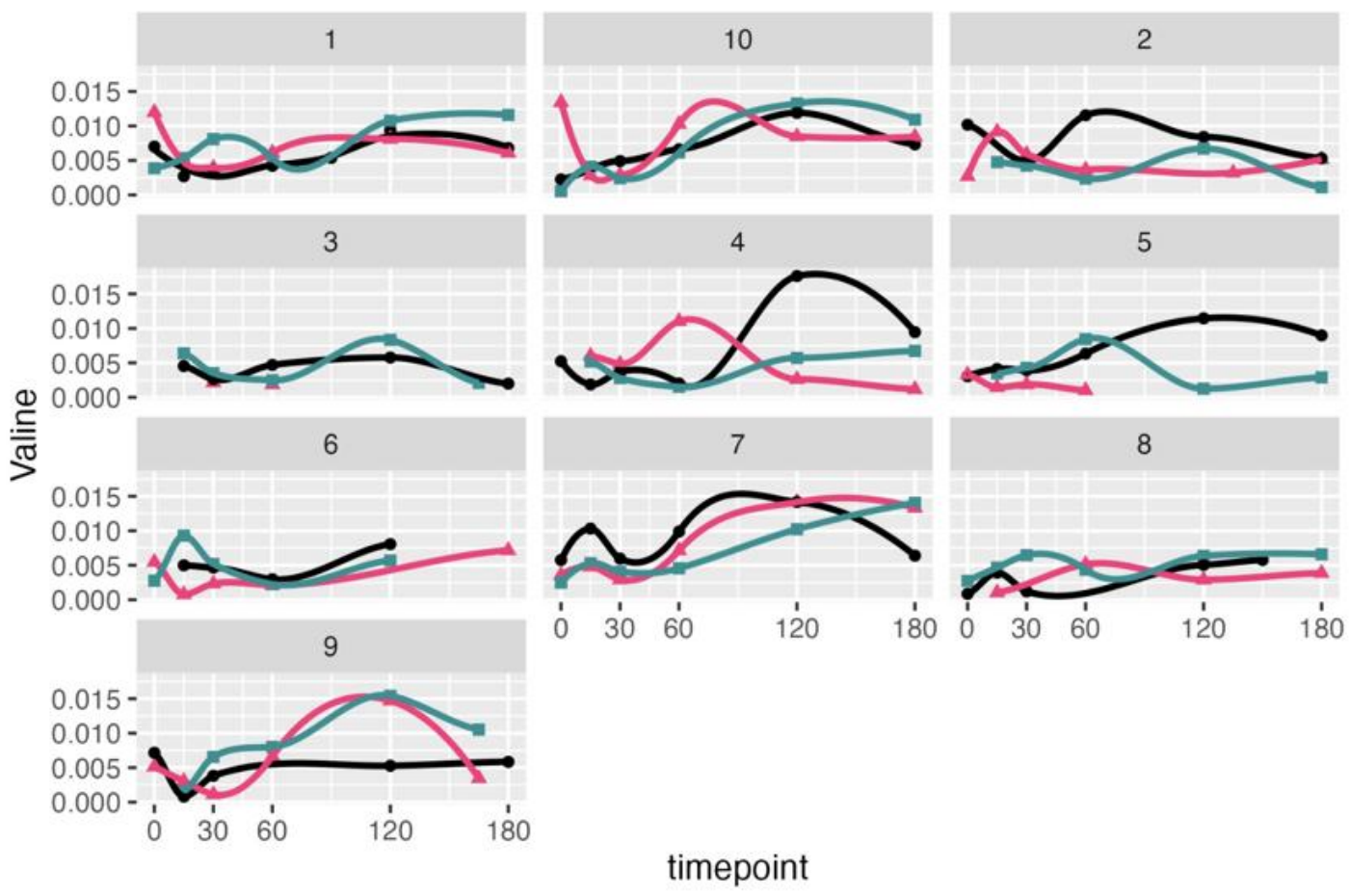

Valine
timepoint
1
10
2
3
4
5
6
7
8
9
0.015
0.010
0.005
0.000
0
30
60
120
180

**Supplementary Figure 5: Per individual metabolic concentration in mmol/L plotted against time after meal consumption, within duodenal samples.** Green curves are diet BC, Red curves are diet CC, Black curves are diet SC.

(a)

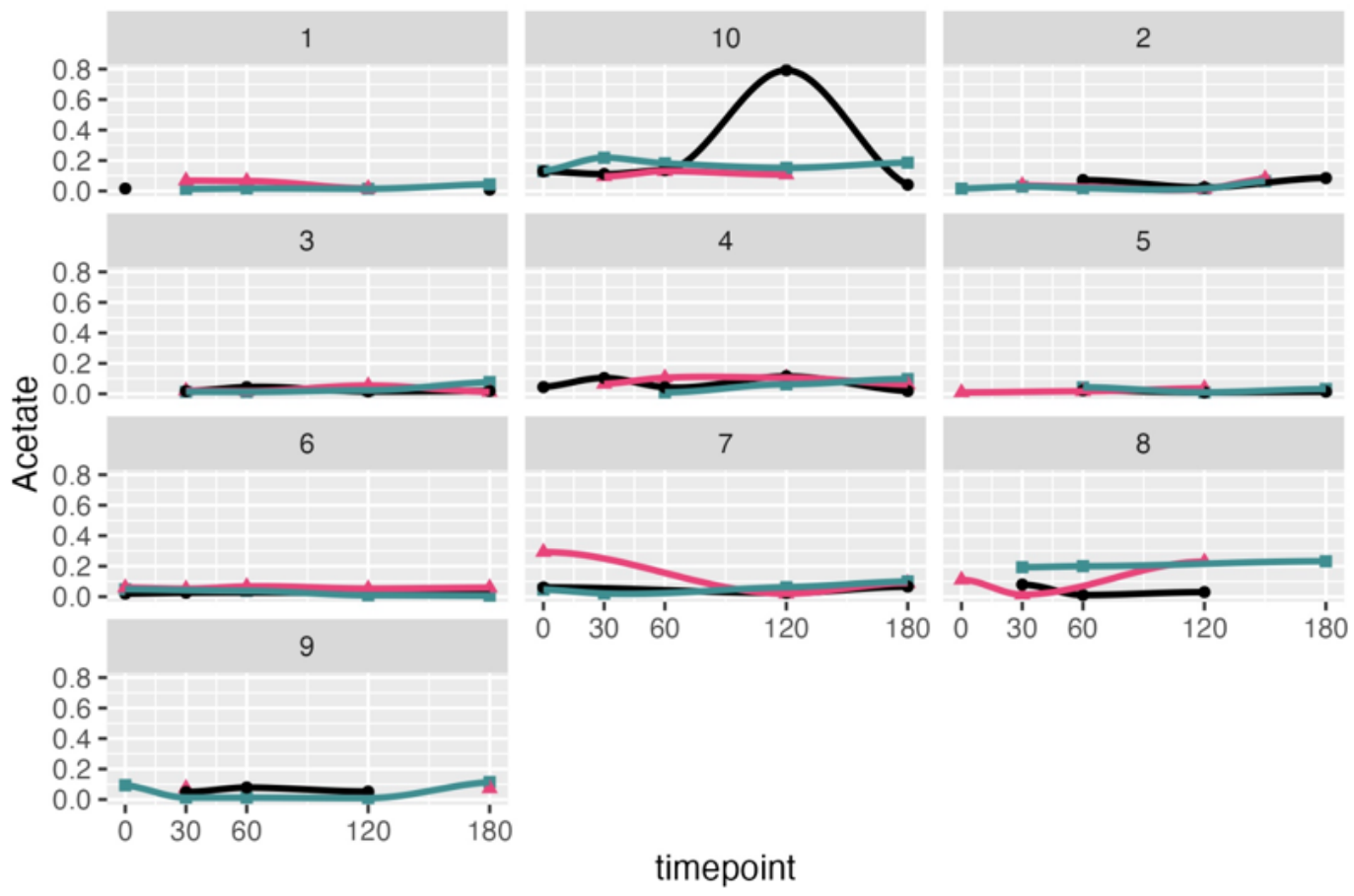


(b)

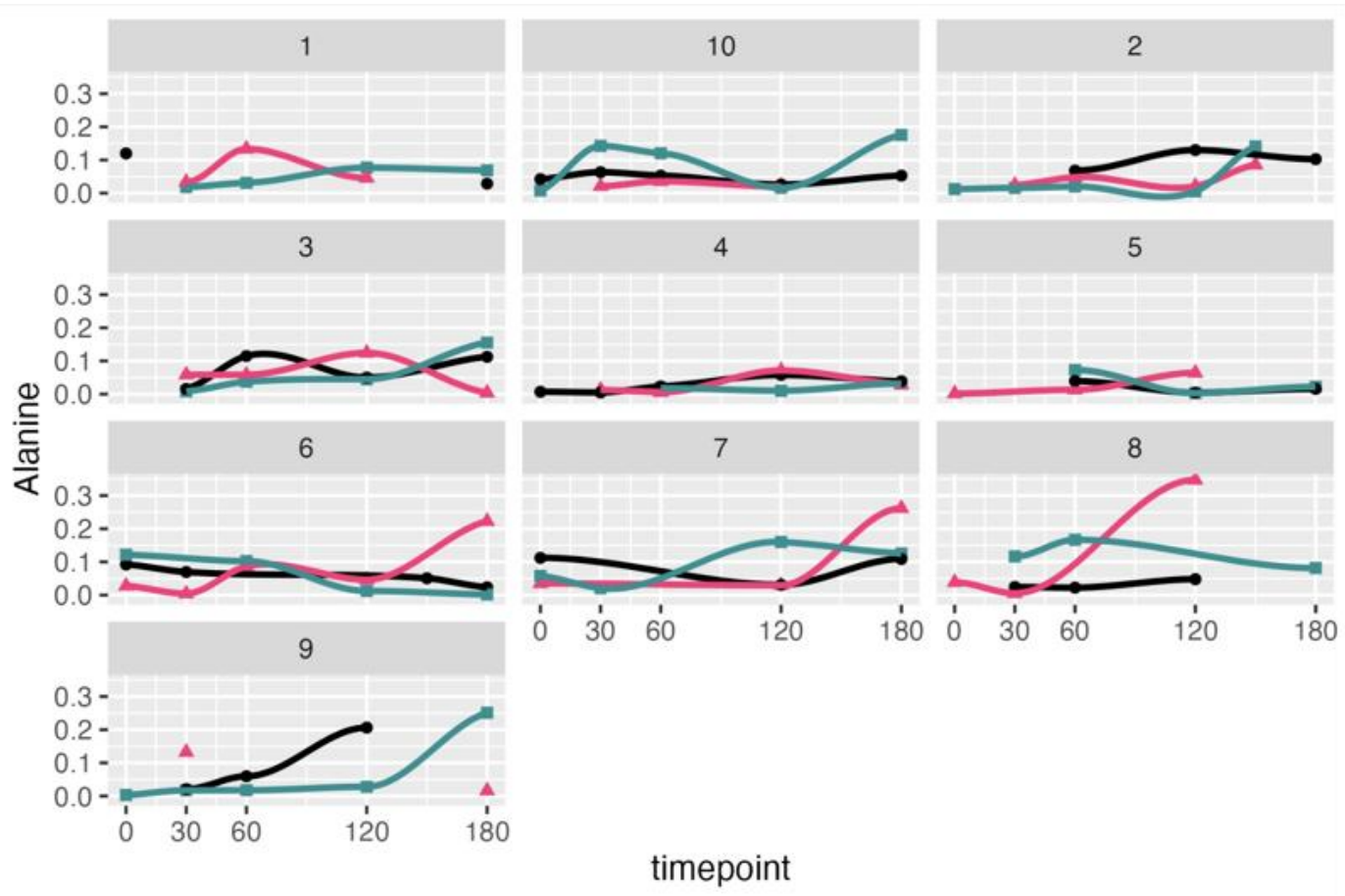


(c)

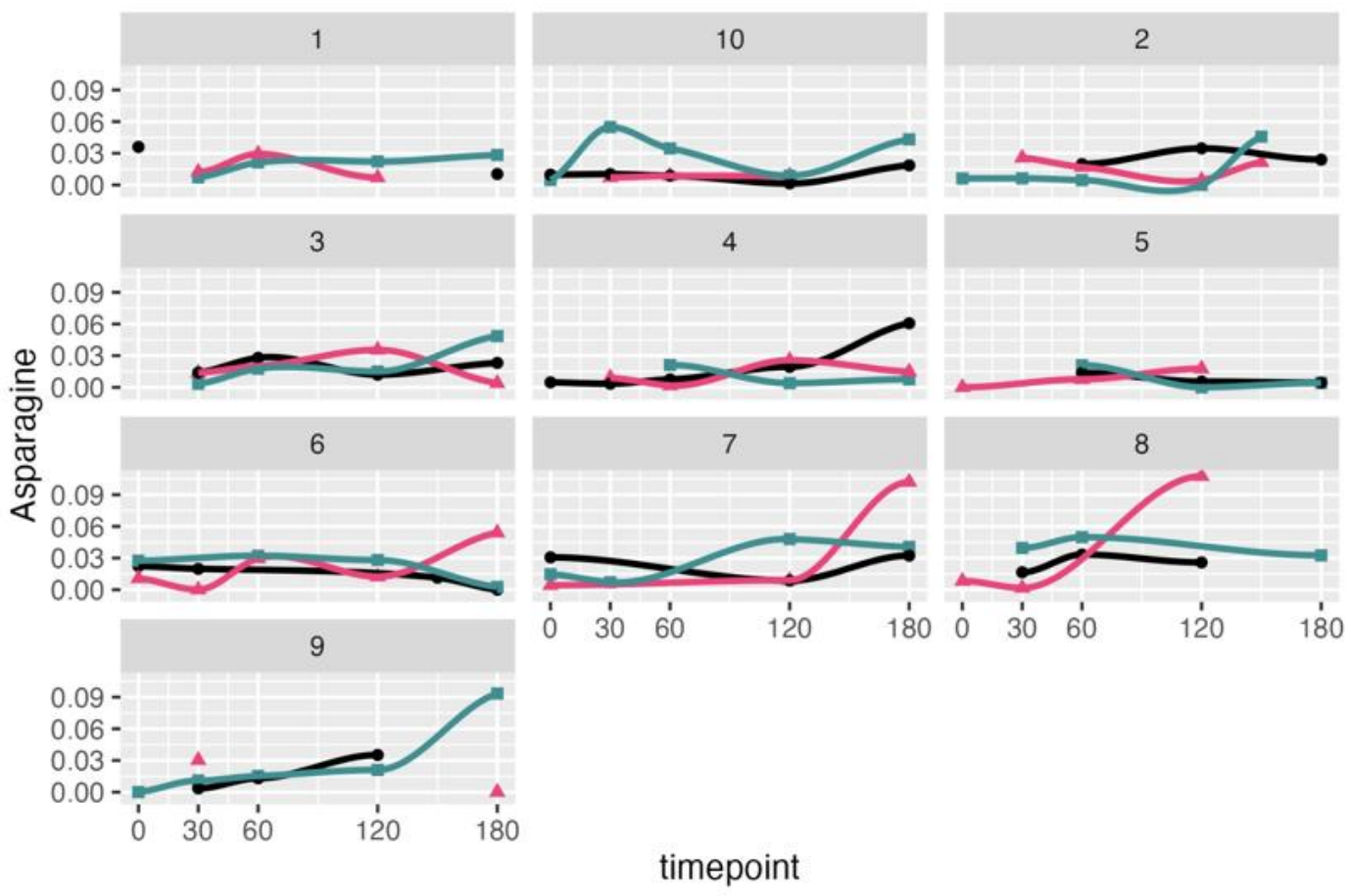


(d)

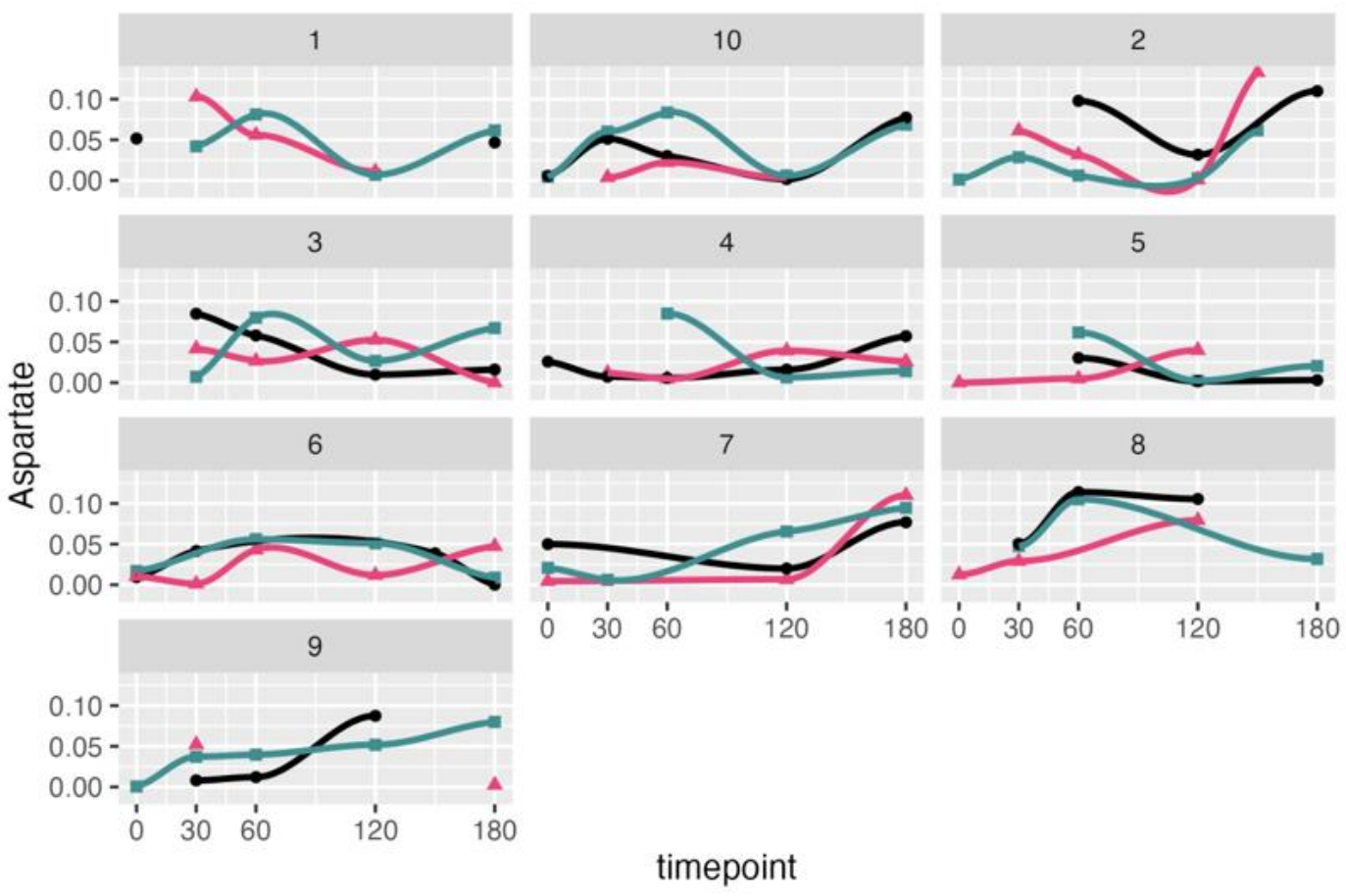


(e)

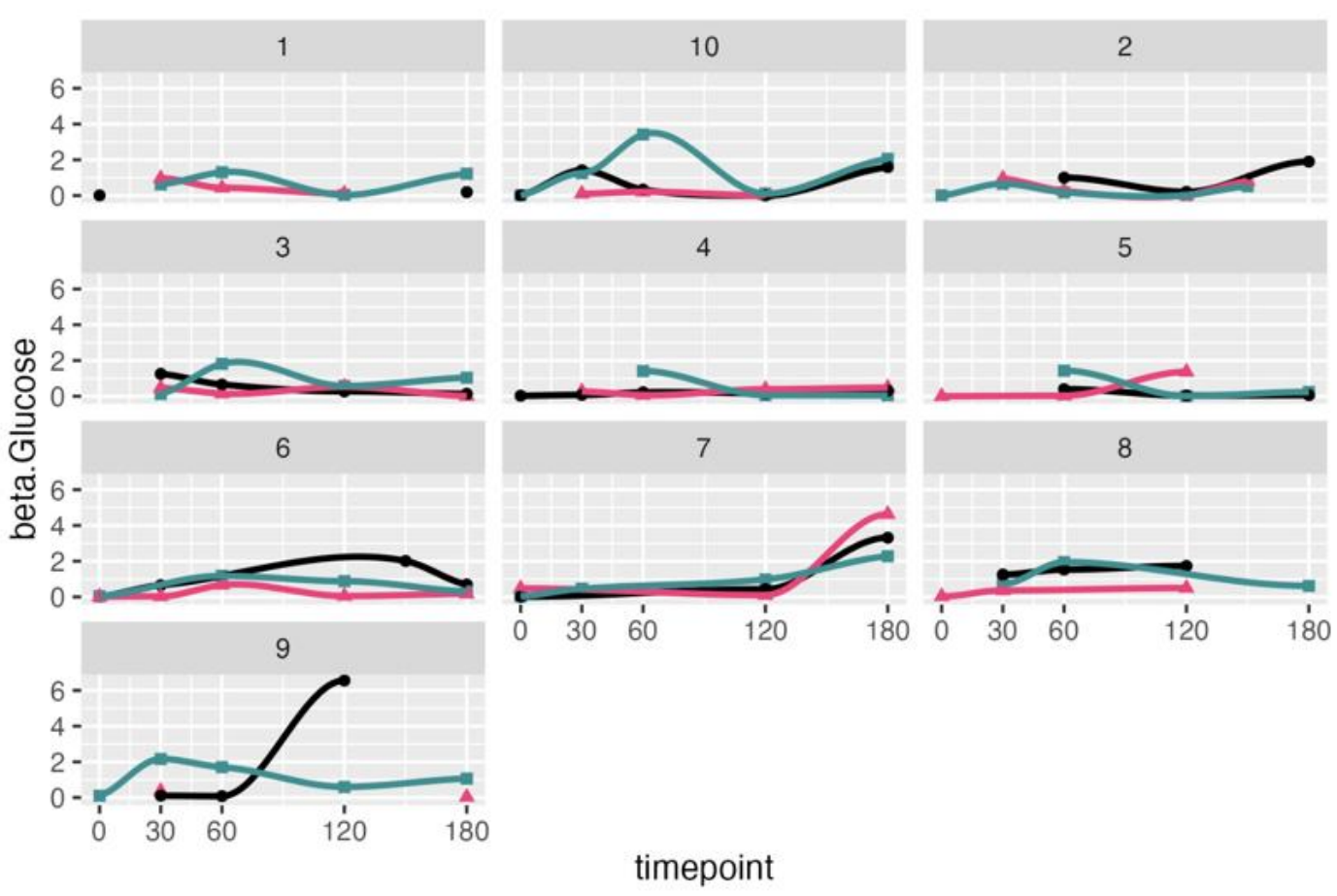


(f)

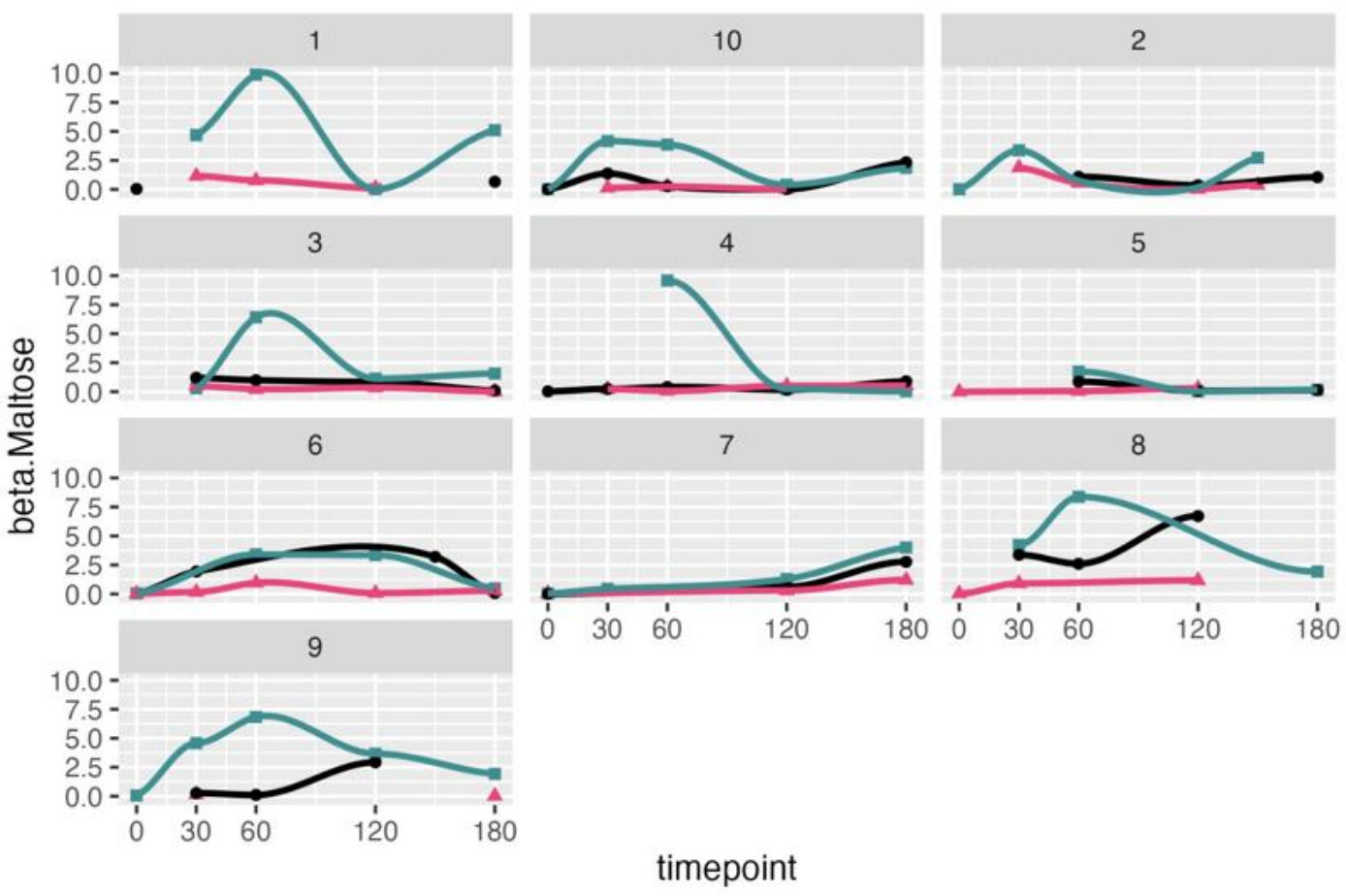

(g)

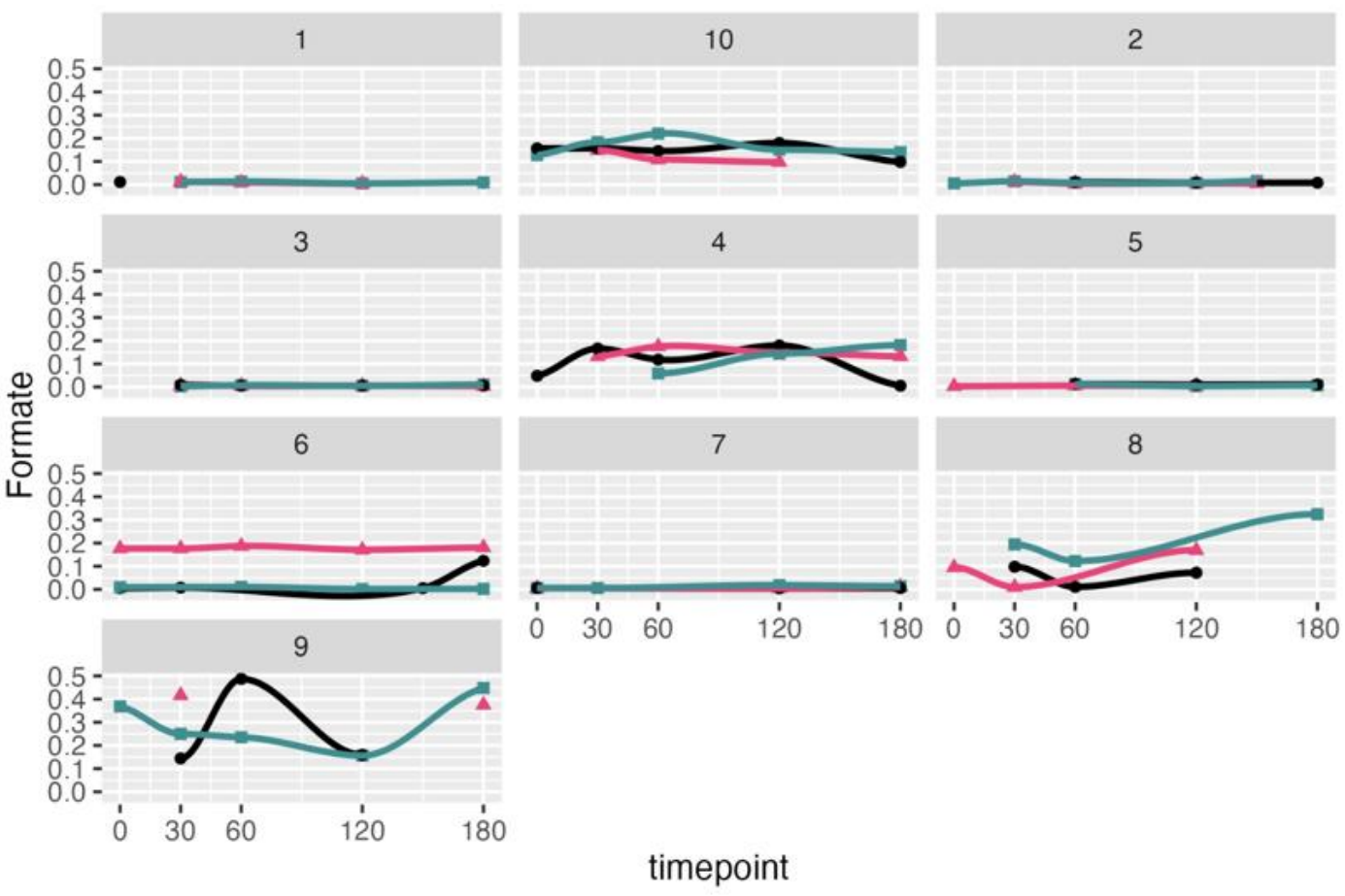

1
10
2
3
4
5
6
7
8
9
Formate
0.5
0.4
0.3
0.2
0.1
0.0
0
30
60
120
180
timepoint


(h)

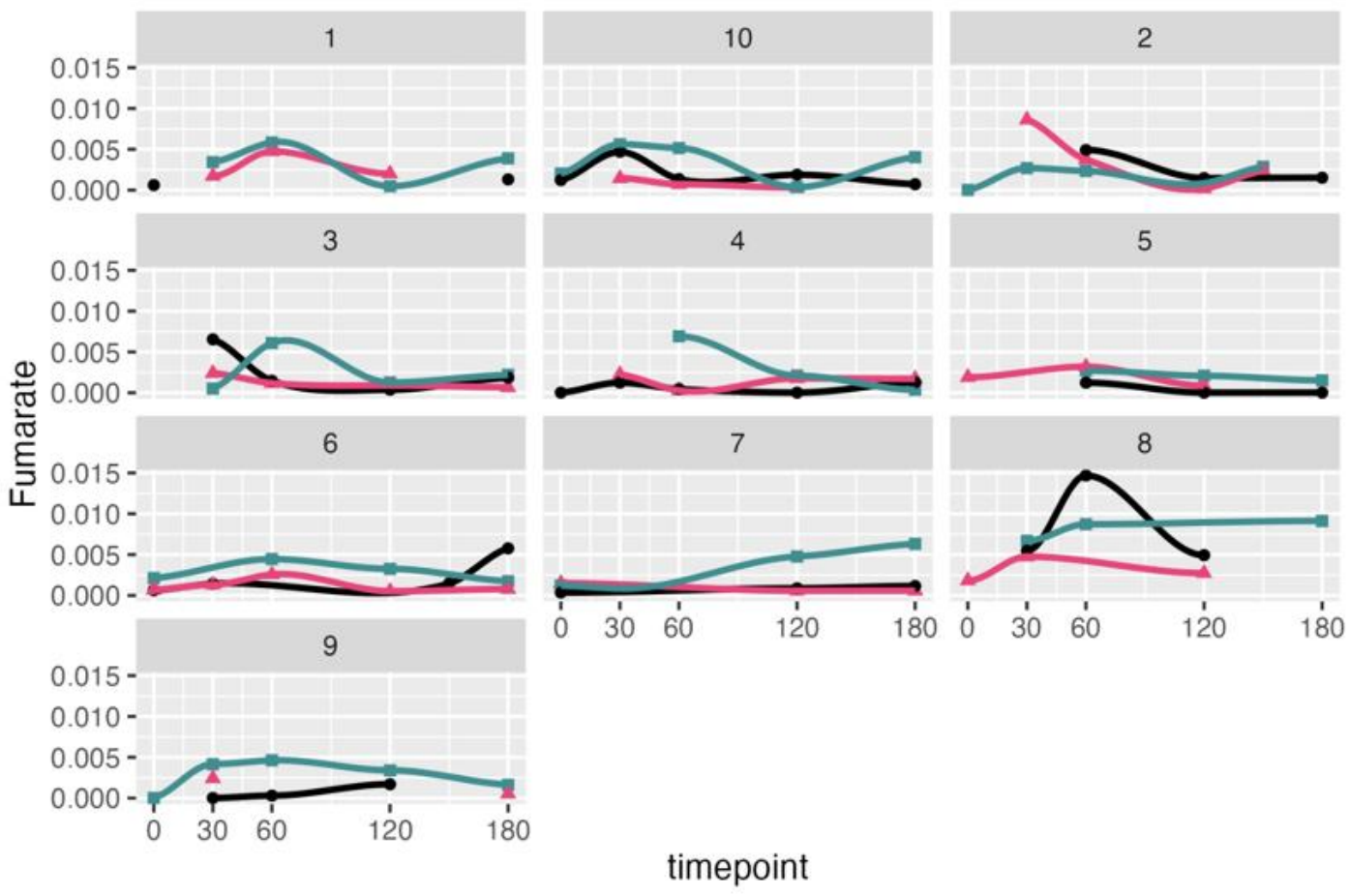

1
10
2
3
4
5
6
7
8
9
Fumarate
0.015
0.010
0.005
0.000
0
30
60
120
180
timepoint


(i)

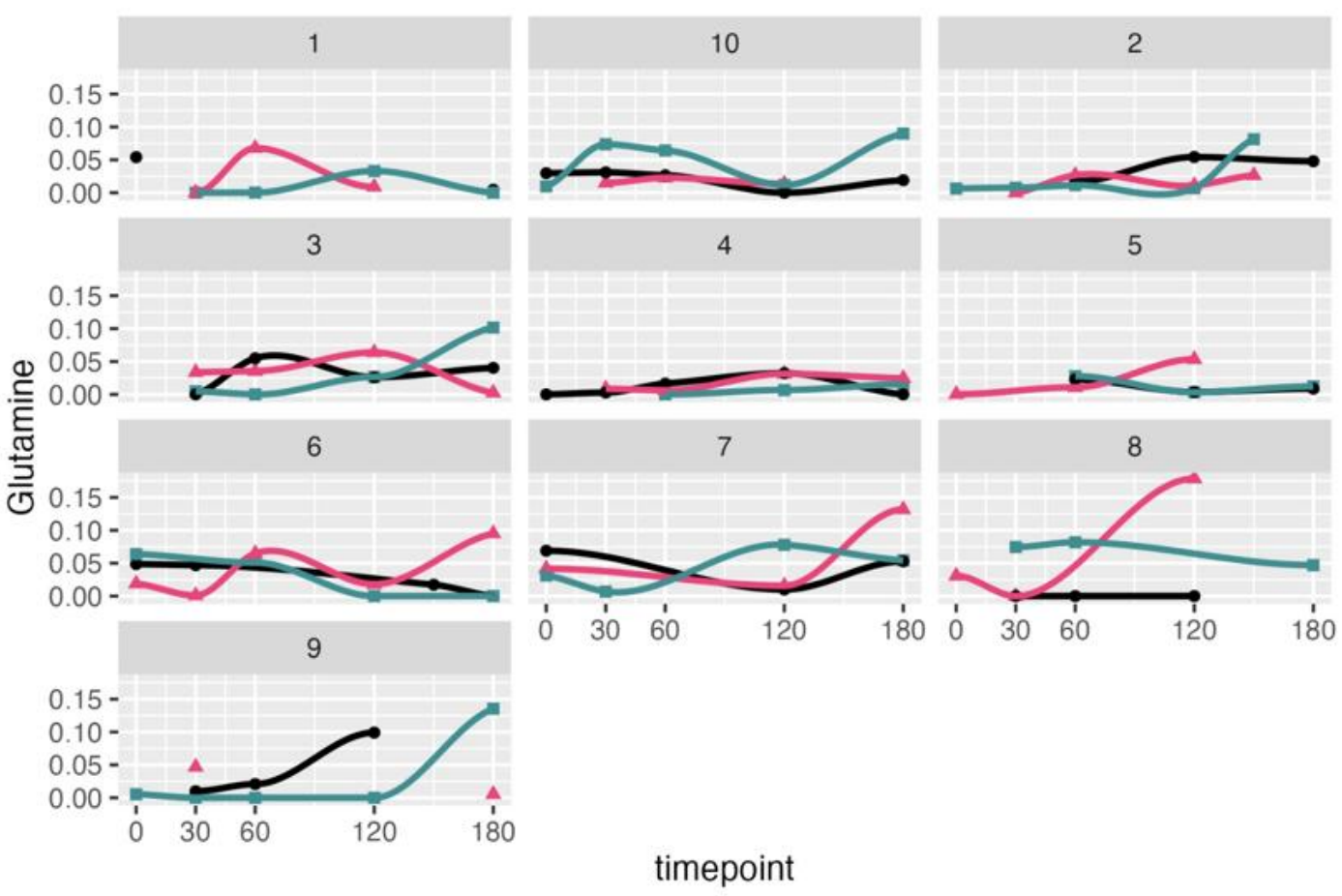

1
10
2
3
4
5
6
7
8
9
Glutamine
0.15
0.10
0.05
0.00
0
30
60
120
180
timepoint


(j)

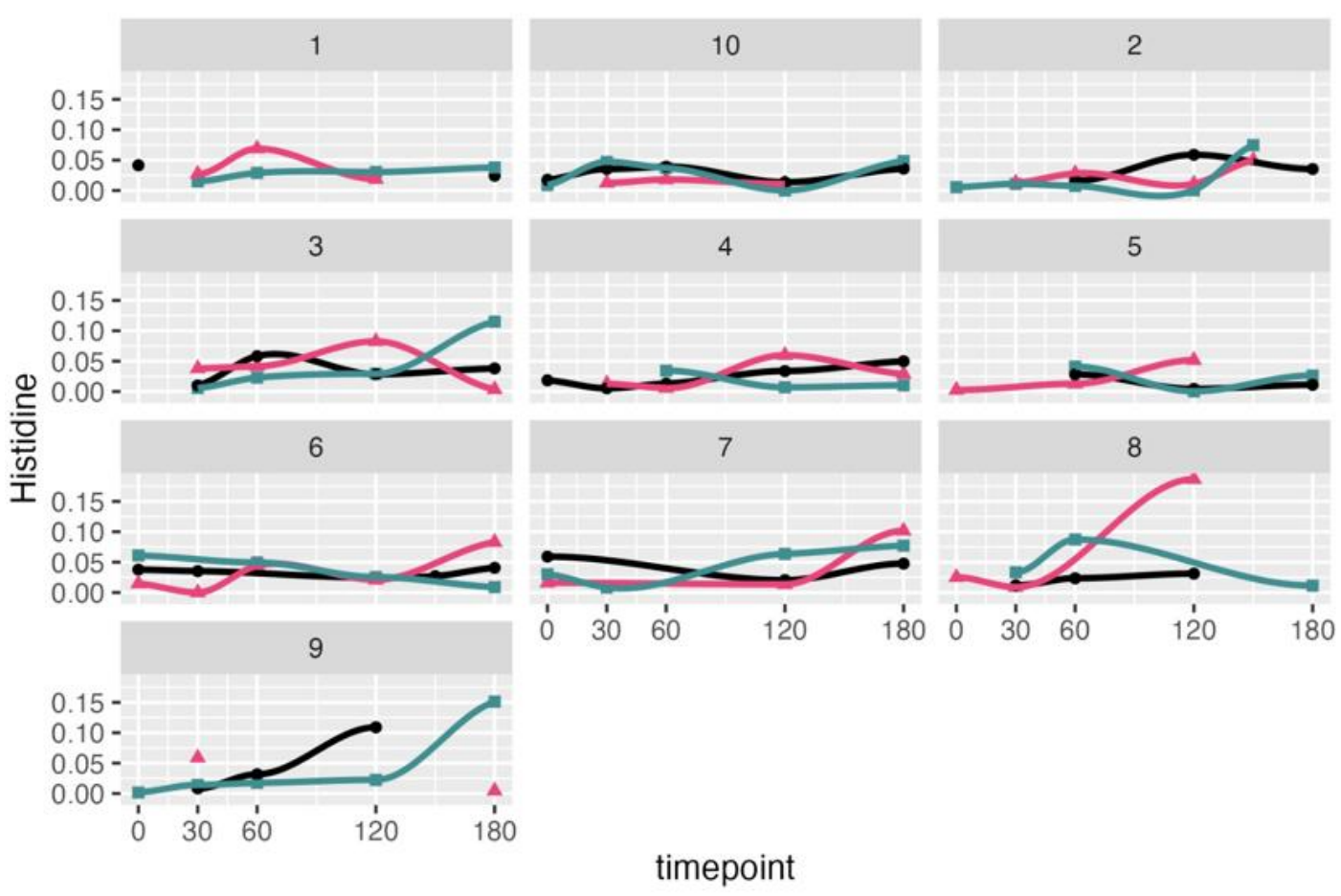

1
10
2
3
4
5
6
7
8
9
Histidine
0.15
0.10
0.05
0.00
0
30
60
120
180
timepoint


(k)

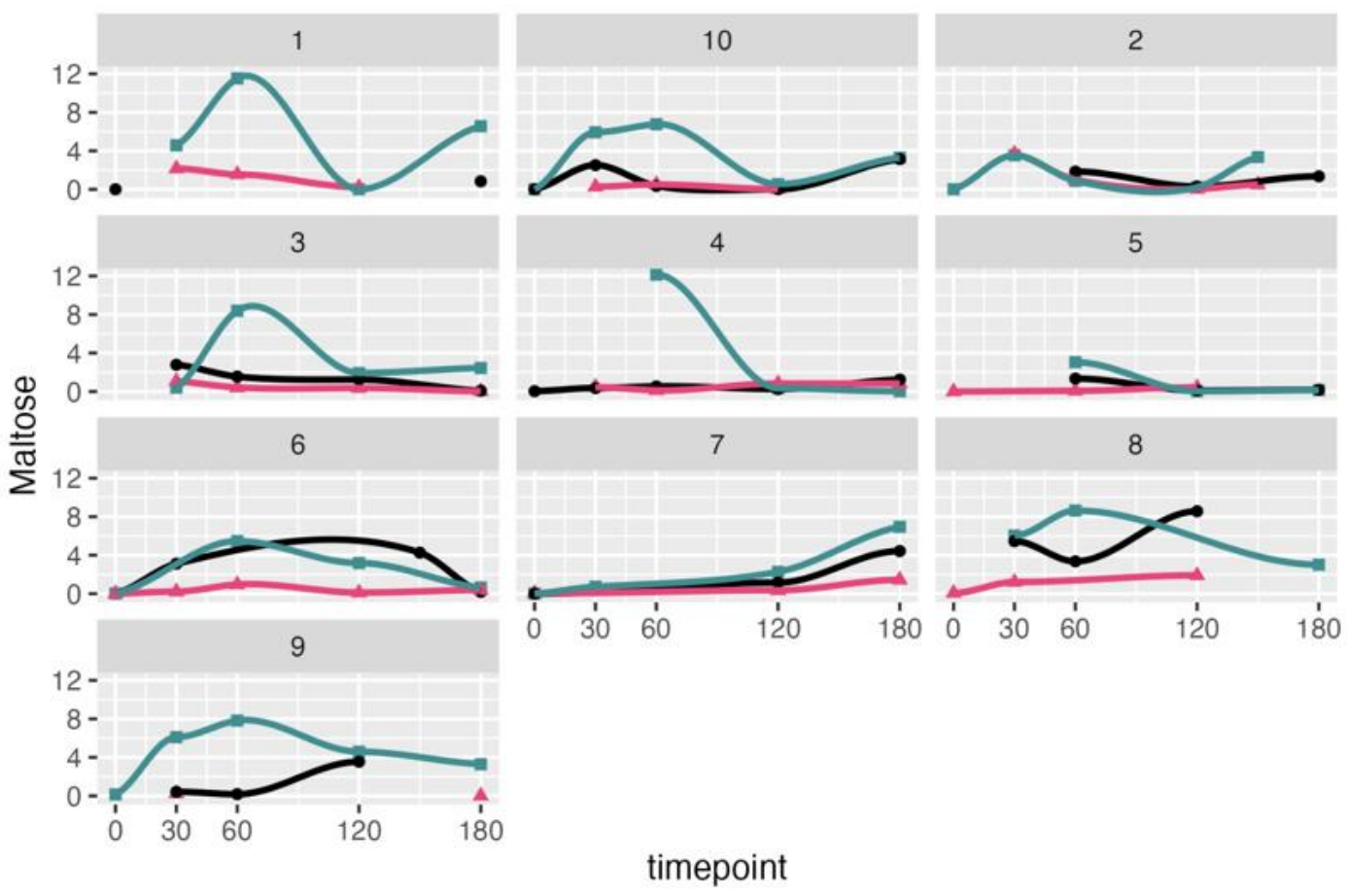

1
10
2
3
4
5
6
7
8
9
Maltose
12
8
4
0
0
30
60
120
180
timepoint


(l)

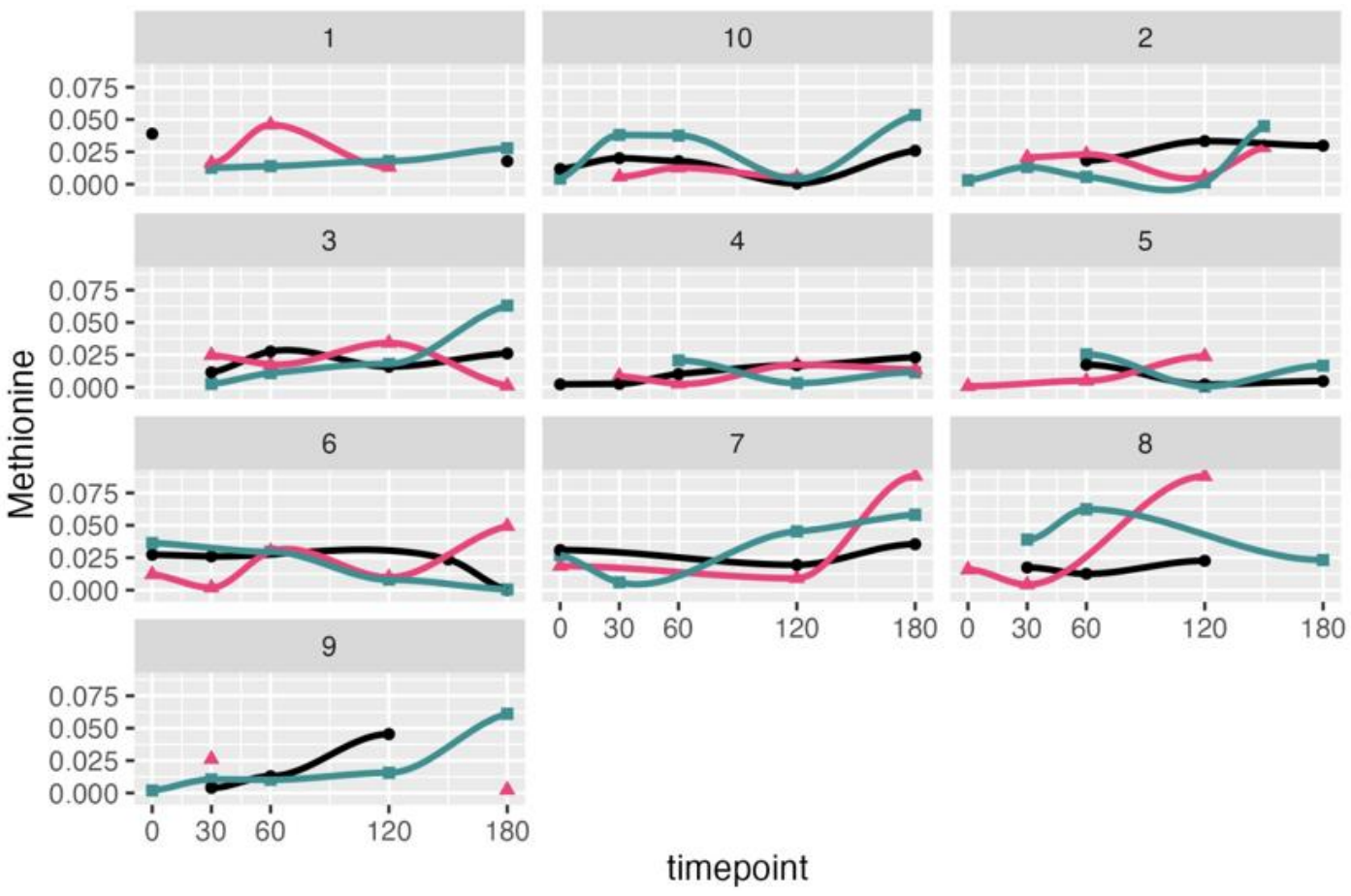

1
10
2
3
4
5
6
7
8
9
Methionine
0.075
0.050
0.025
0.000
0
30
60
120
180
timepoint

**(m)**

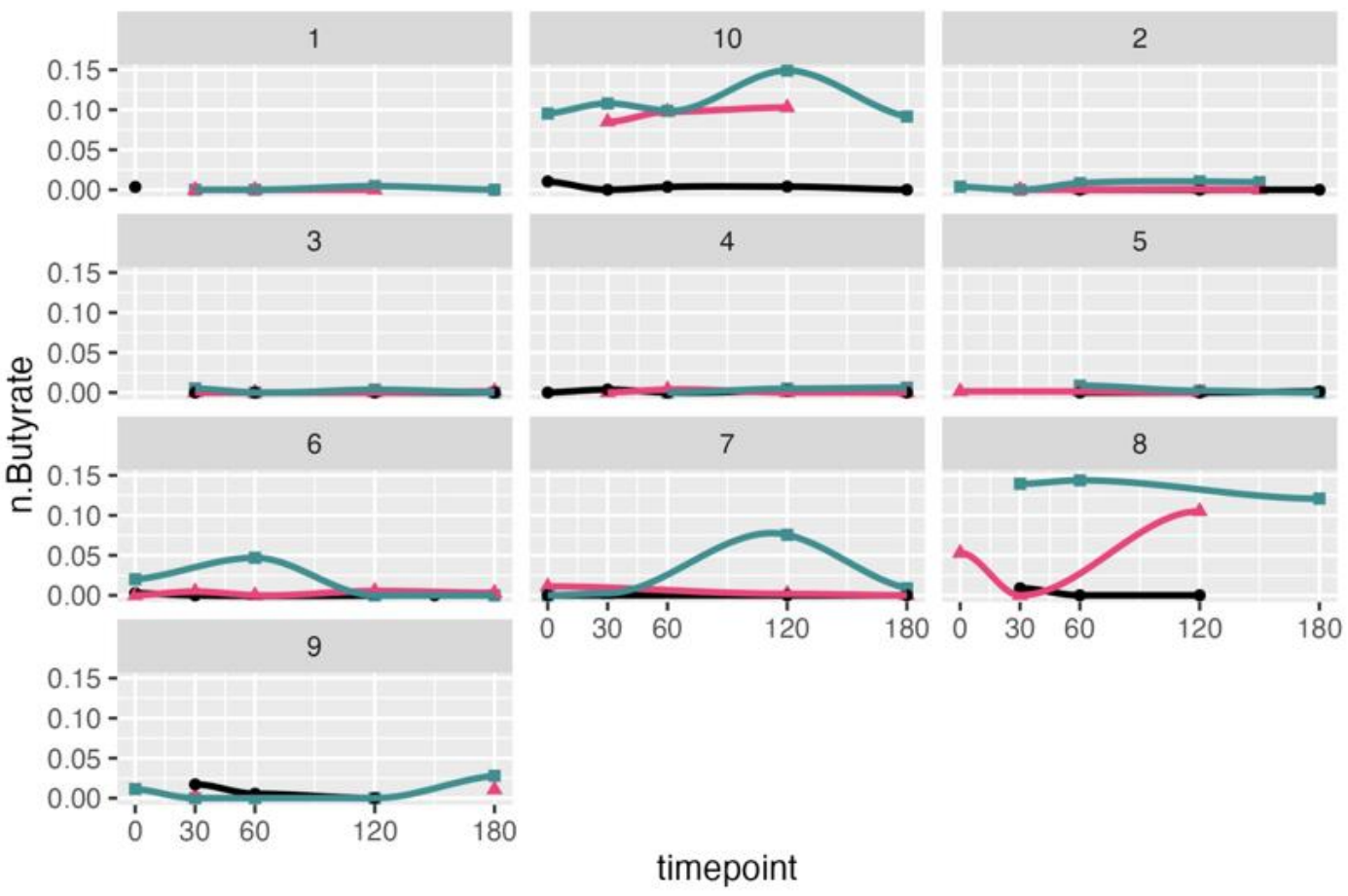
1
10
2
3
4
5
6
7
8
9
n.Butyrate
0.15
0.10
0.05
0.00
0
30
60
120
180
timepoint

**(n)**

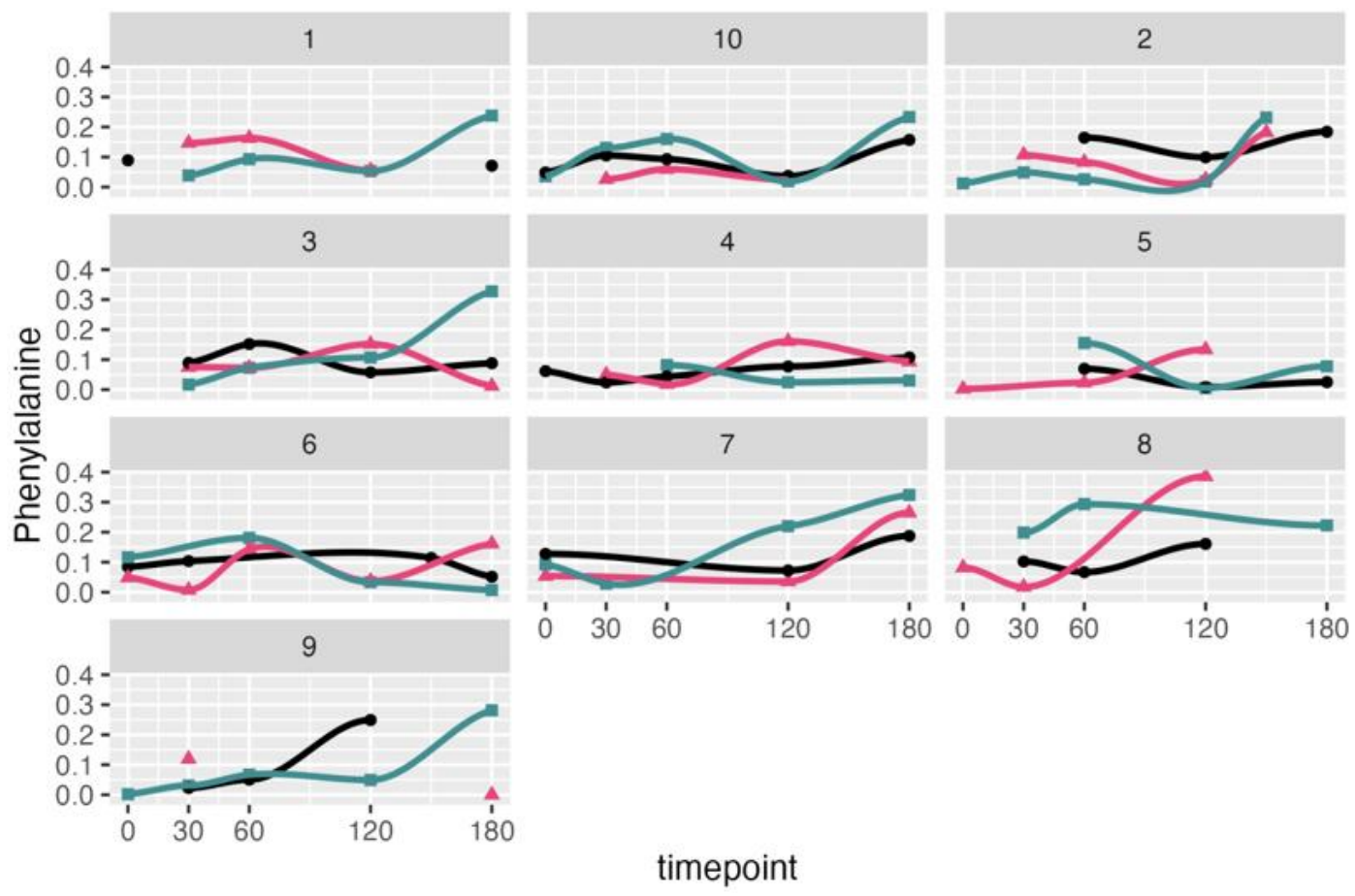
1
10
2
3
4
5
6
7
8
9
Phenylalanine
0.4
0.3
0.2
0.1
0.0
0
30
60
120
180
timepoint

**(o)**

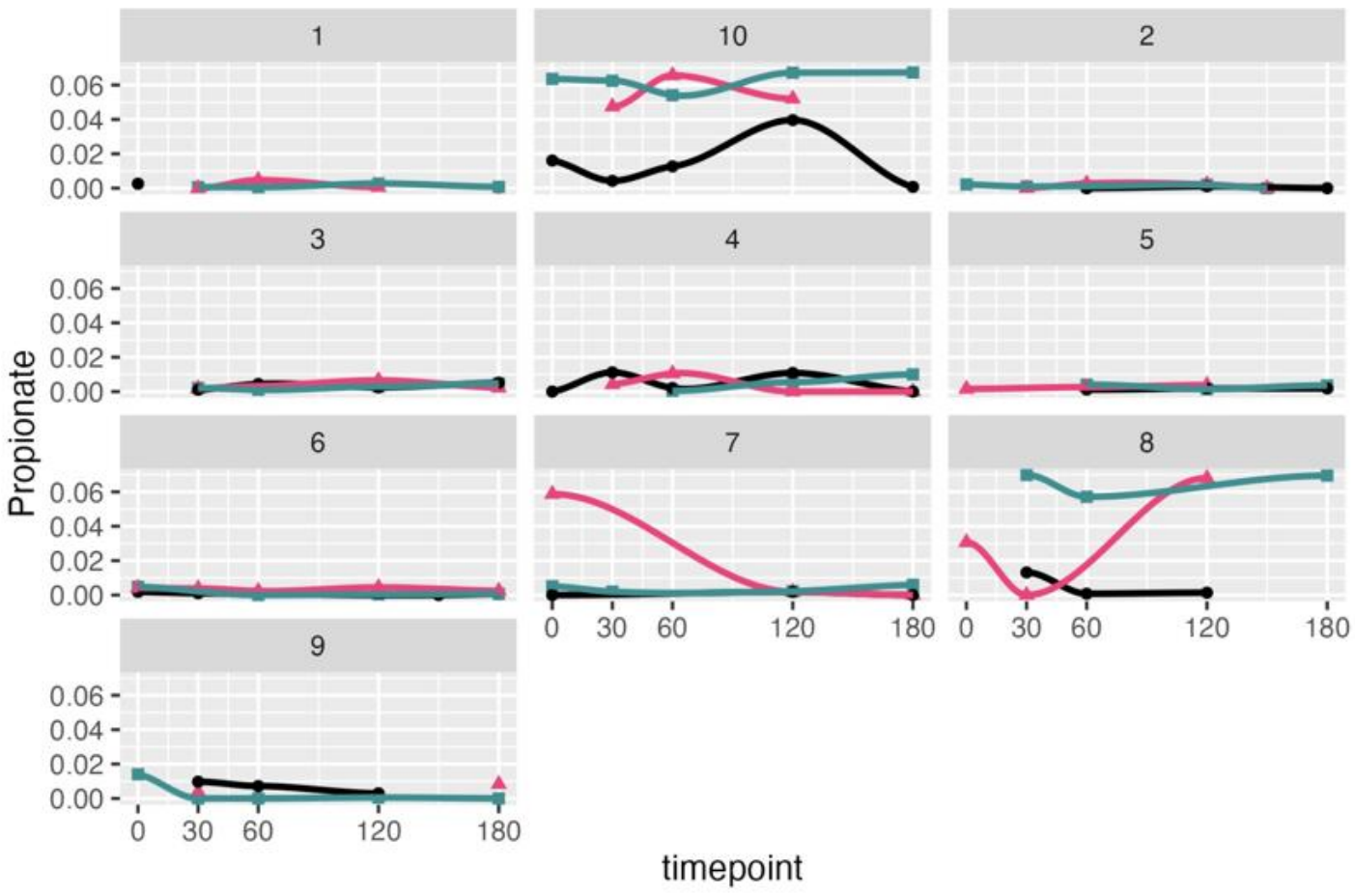
1
10
2
3
4
5
6
7
8
9
Propionate
0.06
0.04
0.02
0.00
0
30
60
120
180
timepoint

**(p)**

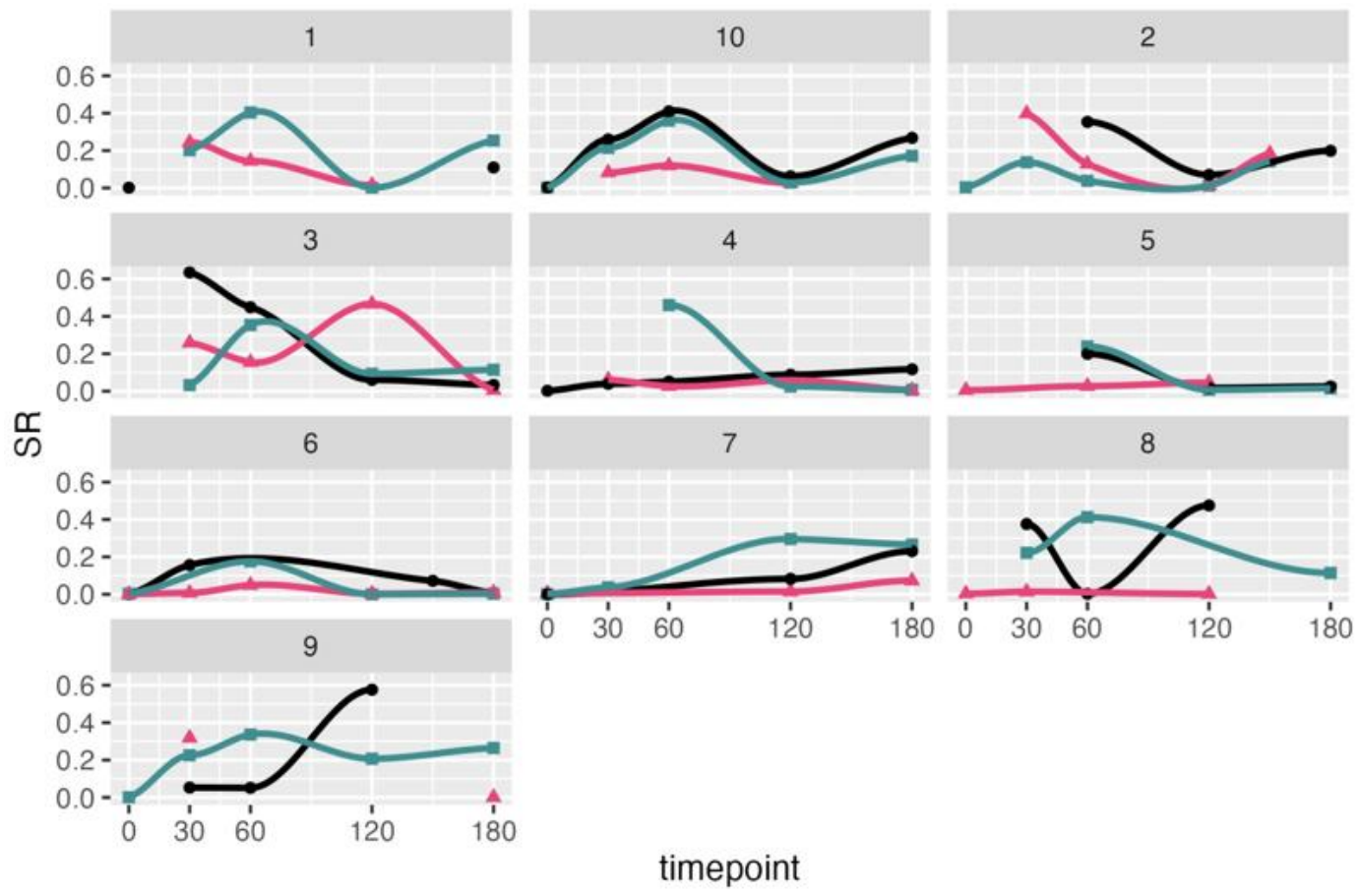
1
10
2
3
4
5
6
7
8
9
SR
0.6
0.4
0.2
0.0
0
30
60
120
180
timepoint

**(q)**

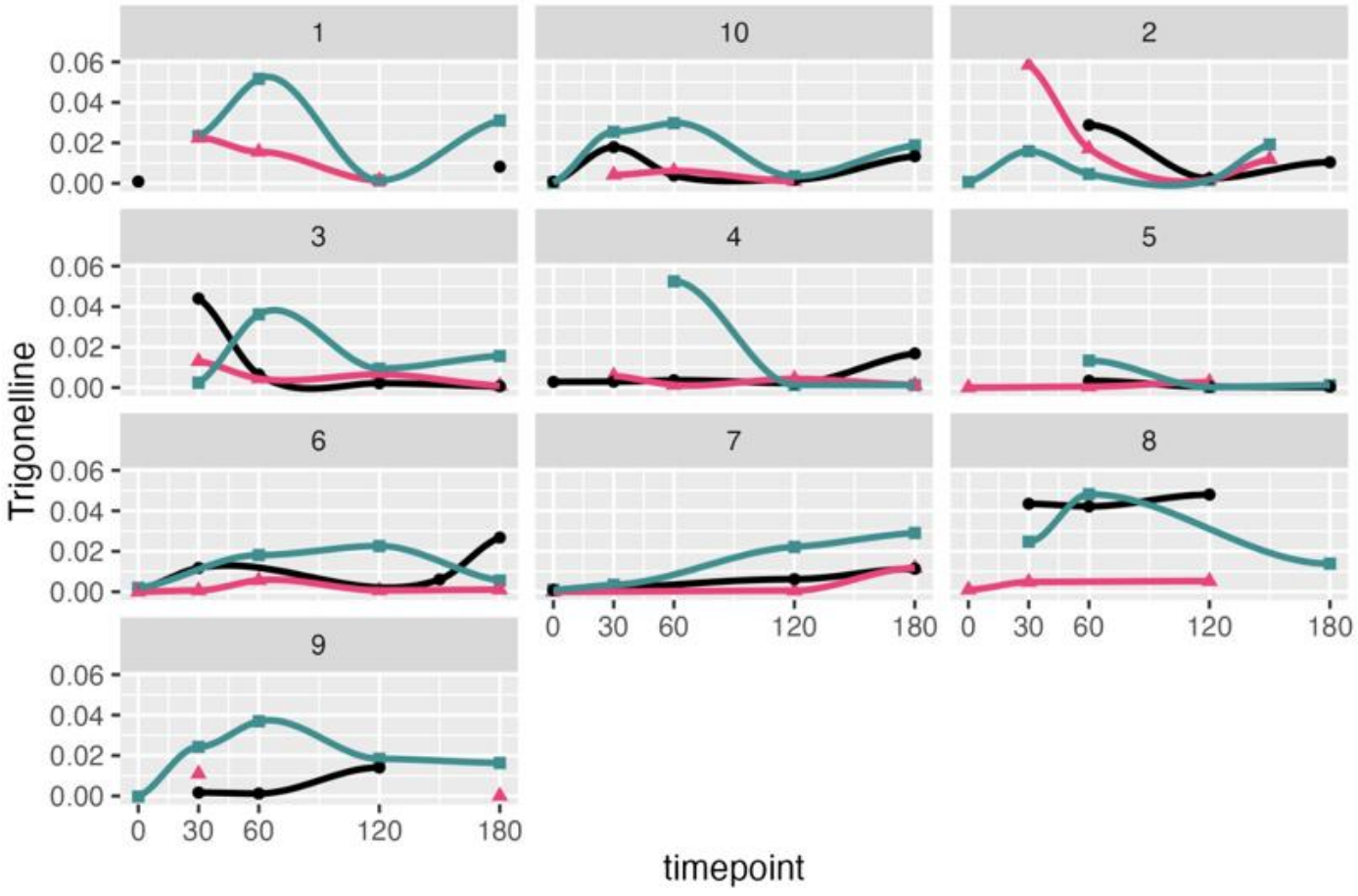
1
10
2
3
4
5
6
7
8
9
Trigonelline
0.06
0.04
0.02
0.00
0
30
60
120
180
timepoint

**(r)**

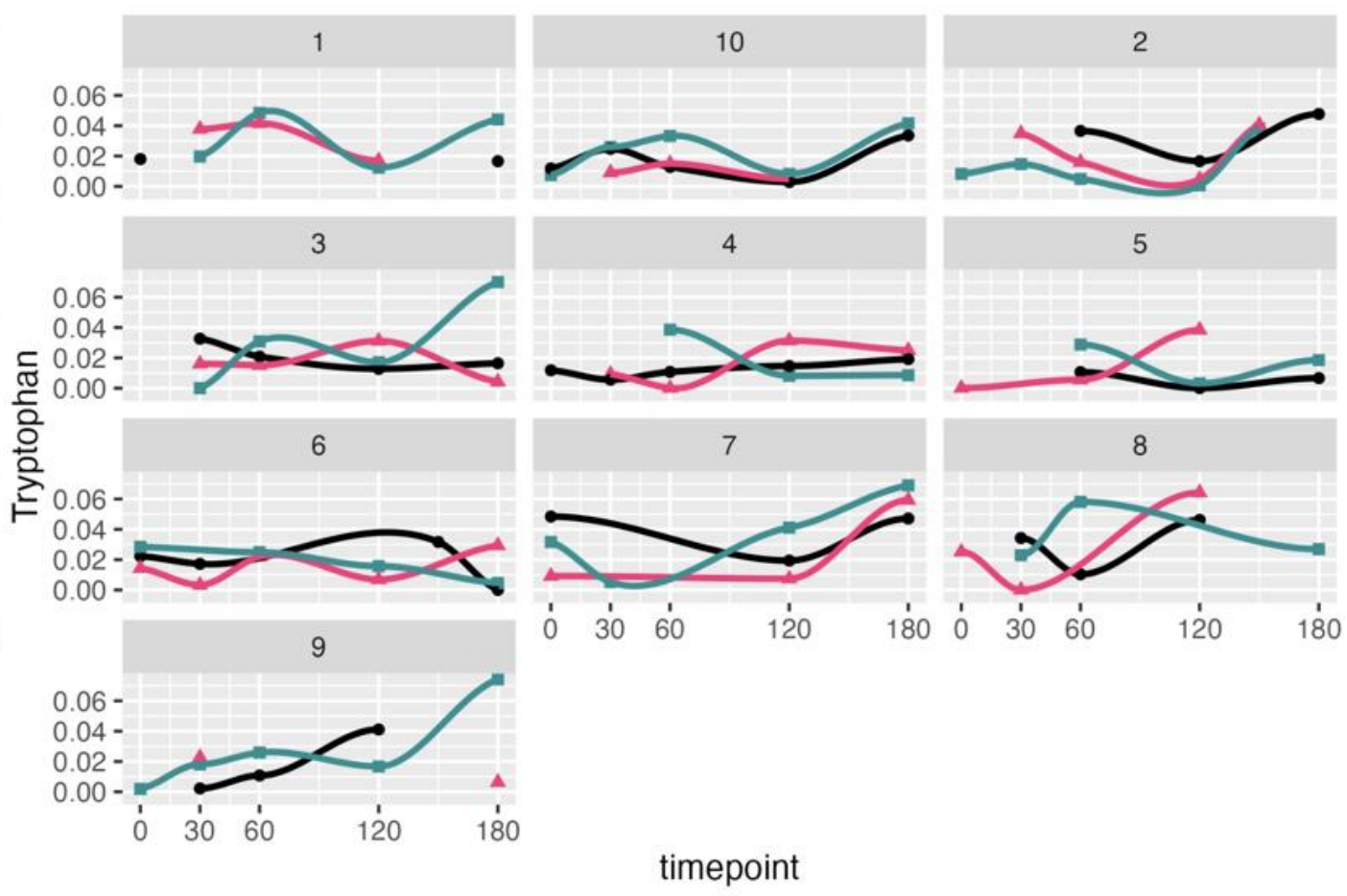
1
10
2
3
4
5
6
7
8
9
Tryptophan
0.06
0.04
0.02
0.00
0
30
60
120
180
timepoint

**Supplementary Figure 6: Per individual metabolic concentration in mmol/L plotted against time after meal consumption, within ileal samples.** Green curves are diet BC, Red curves are diet CC, Black curves are diet SC.

**(a)**

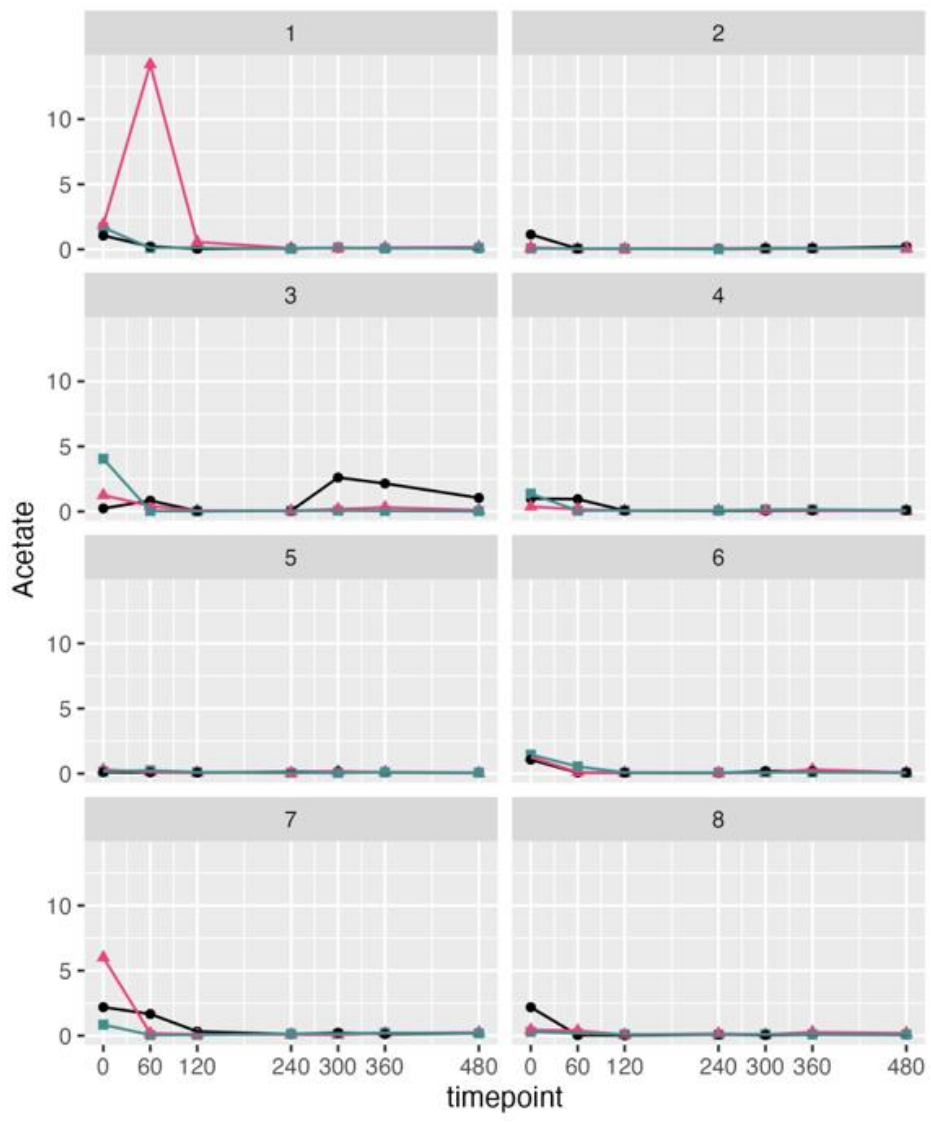


**(b)**

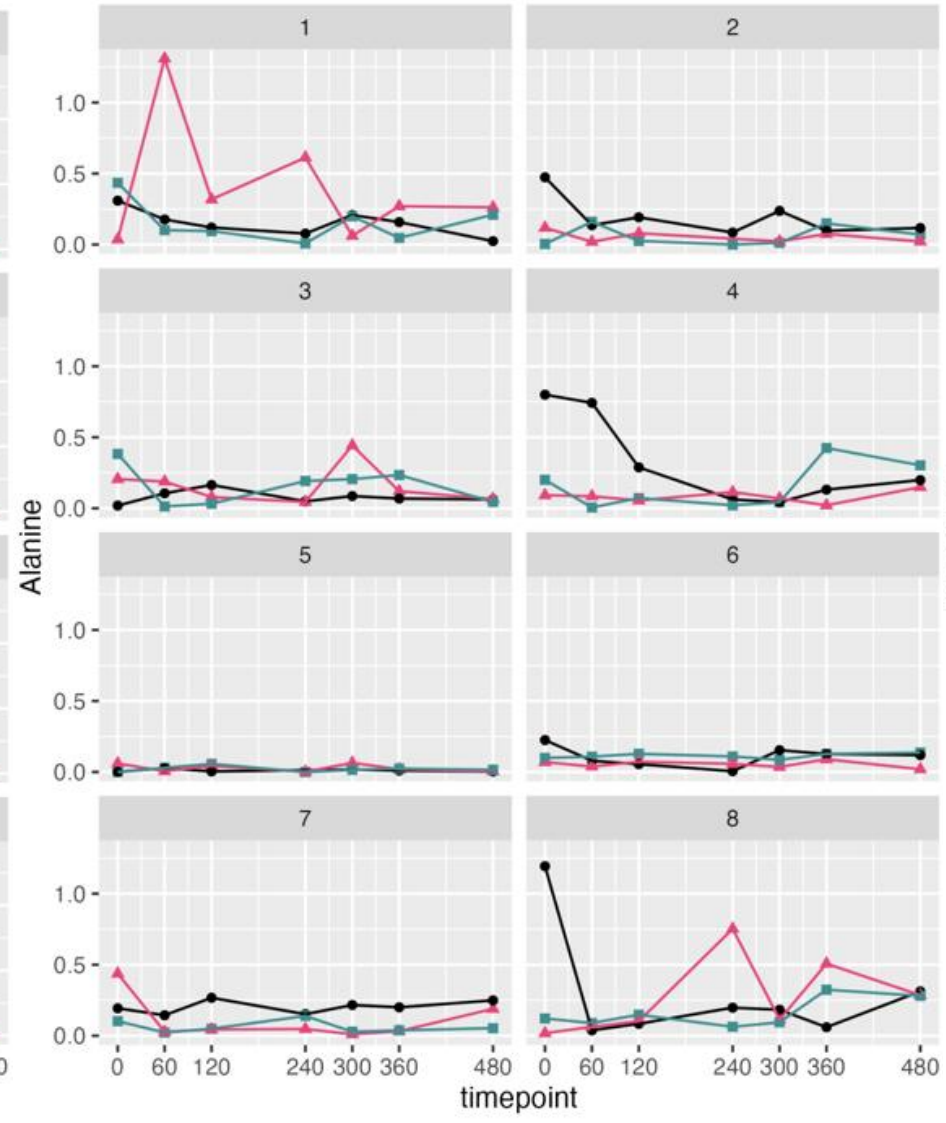


**(c)**

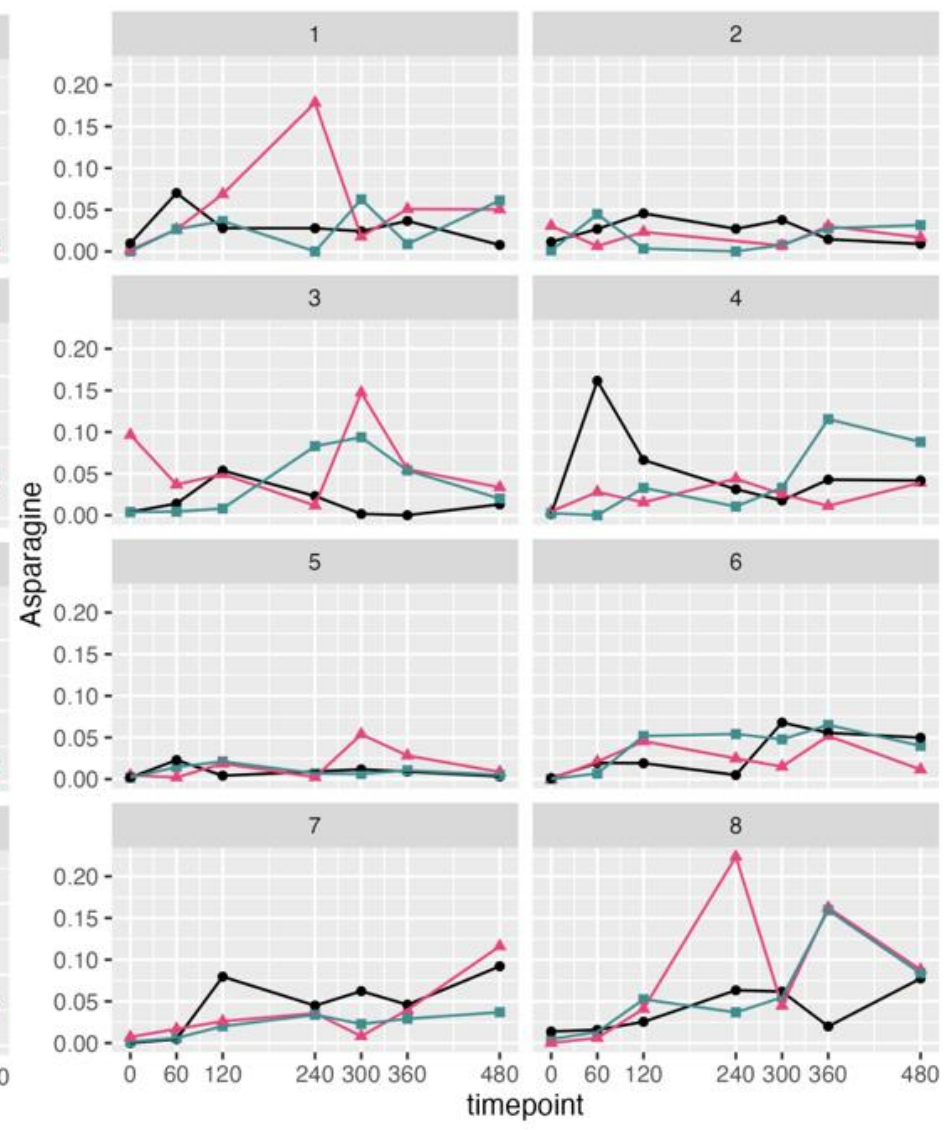


**(d)**

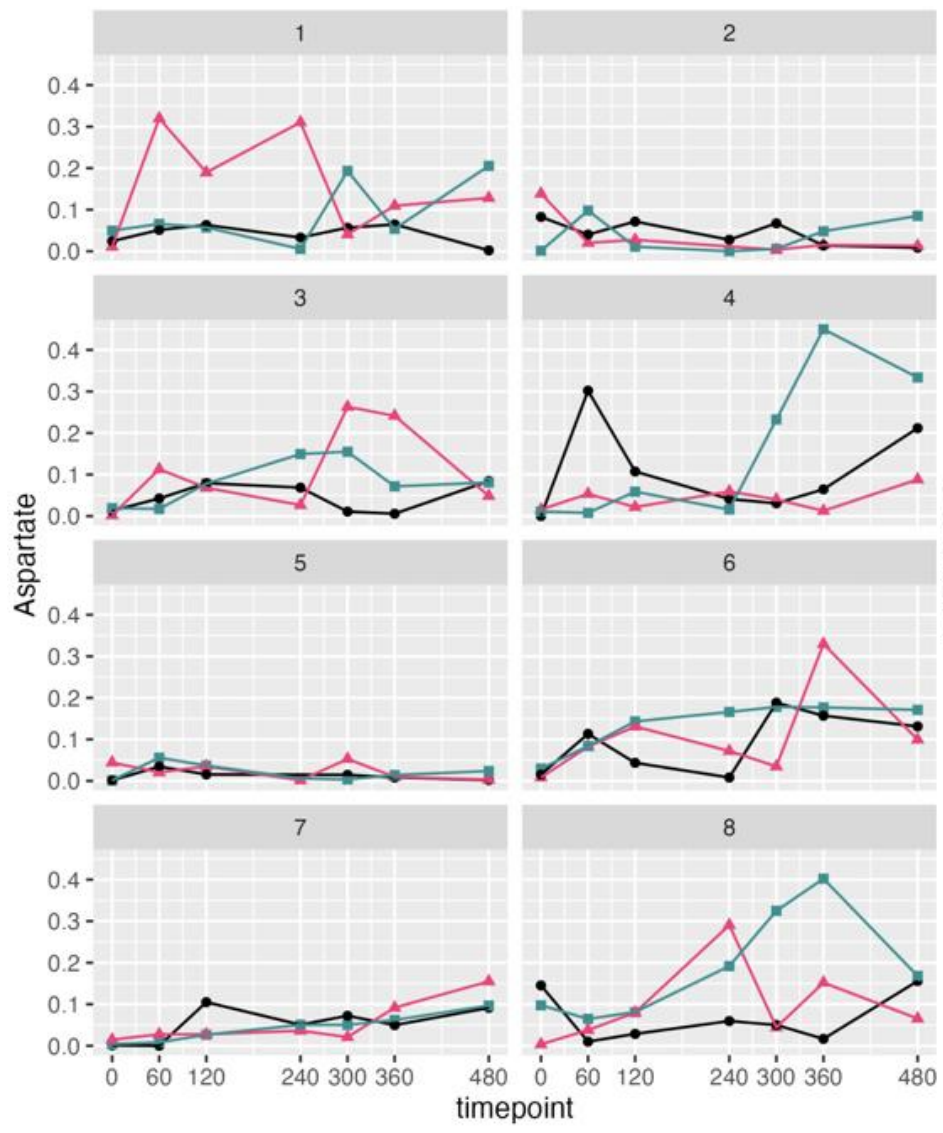


**(e)**

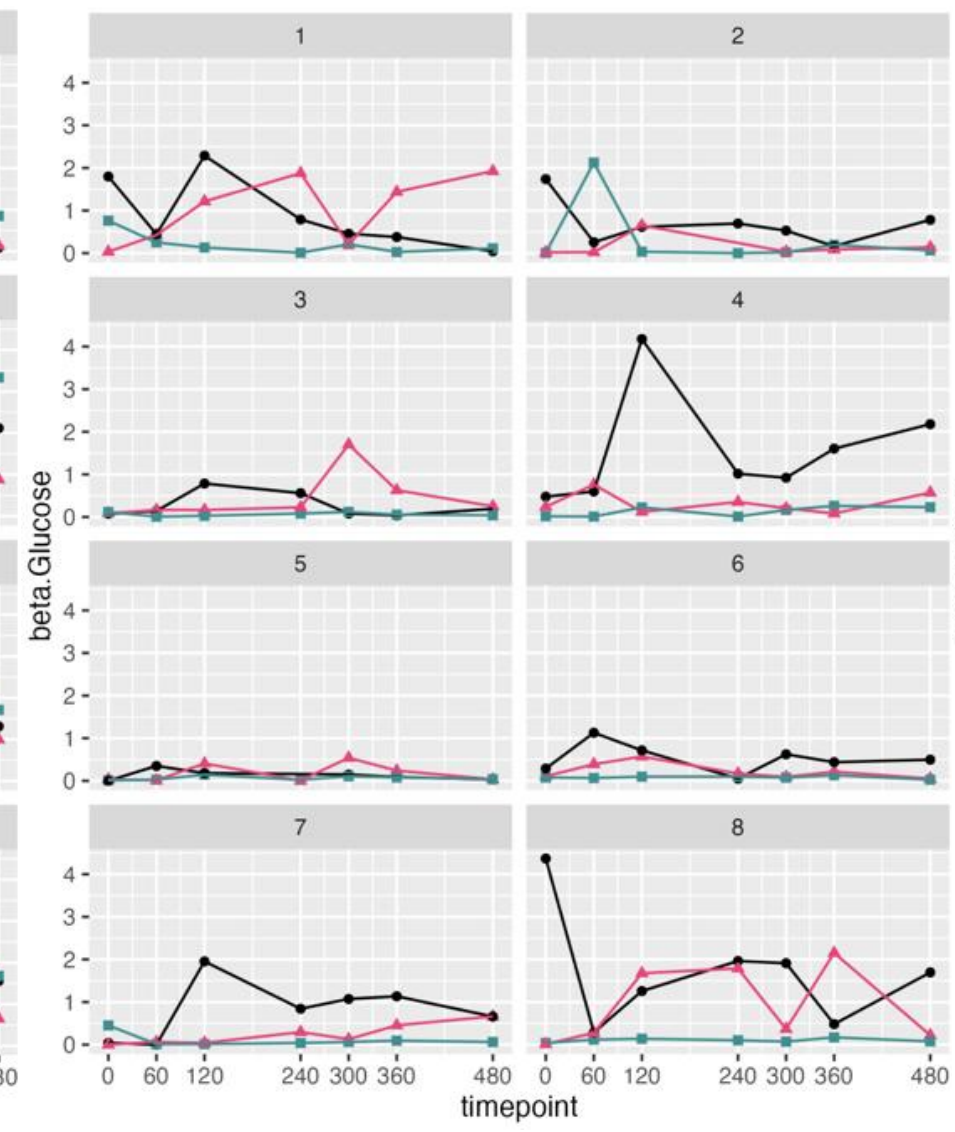


**(f)**

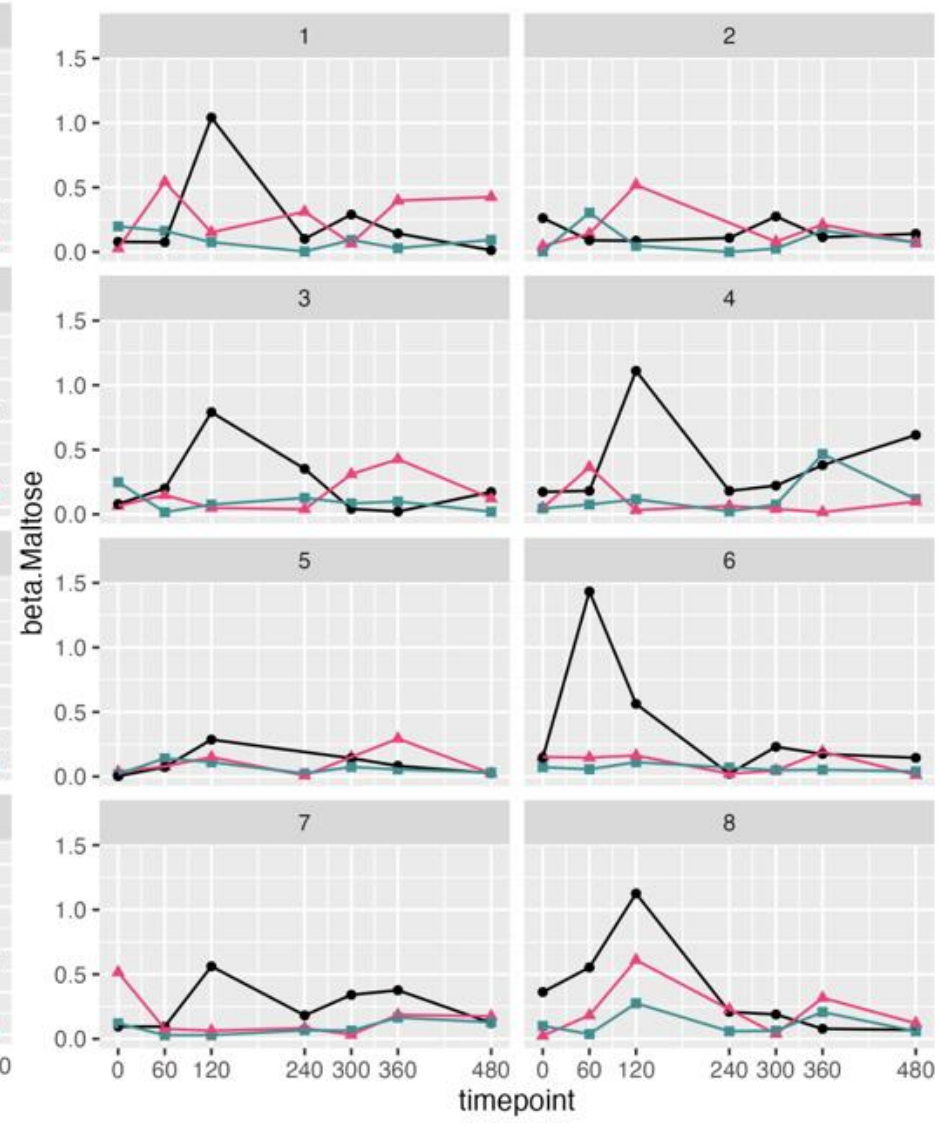

**(g)**

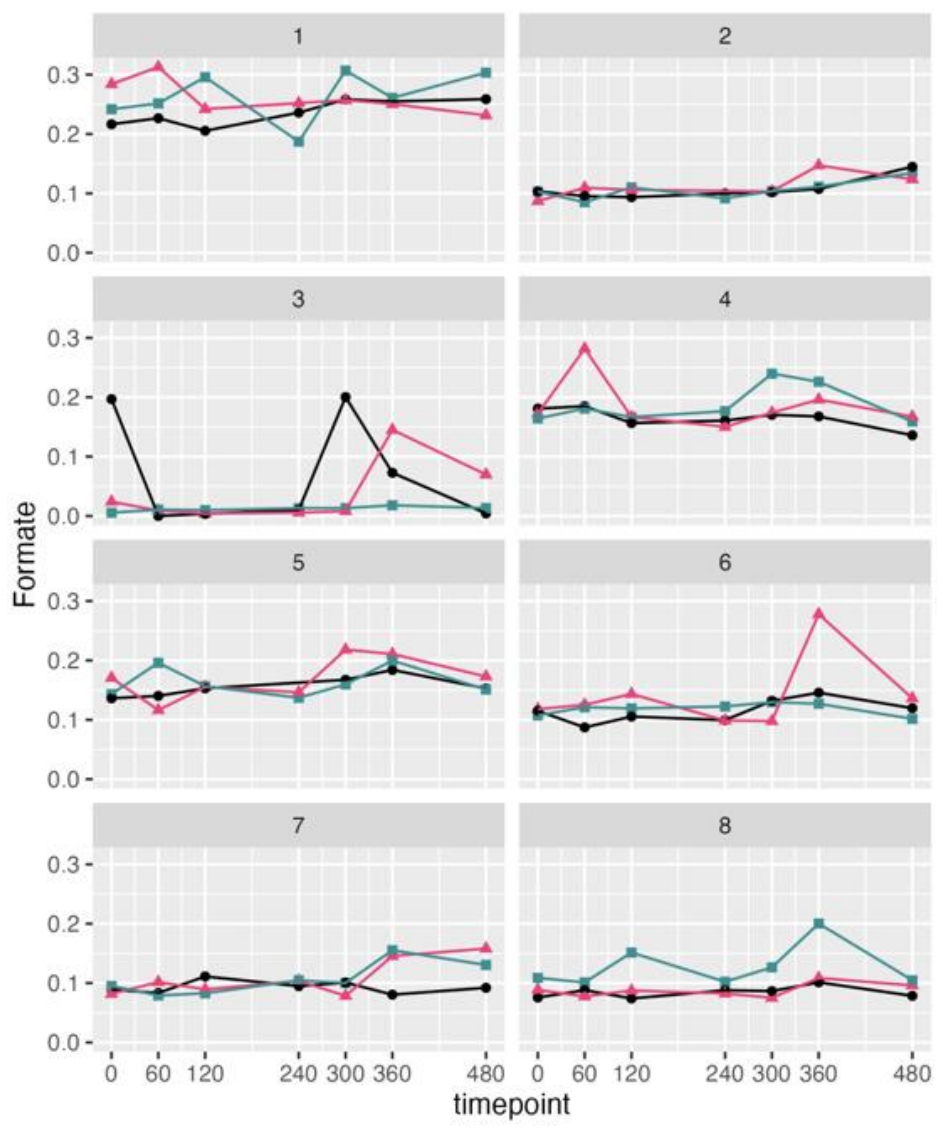
Formate
timepoint

**(h)**

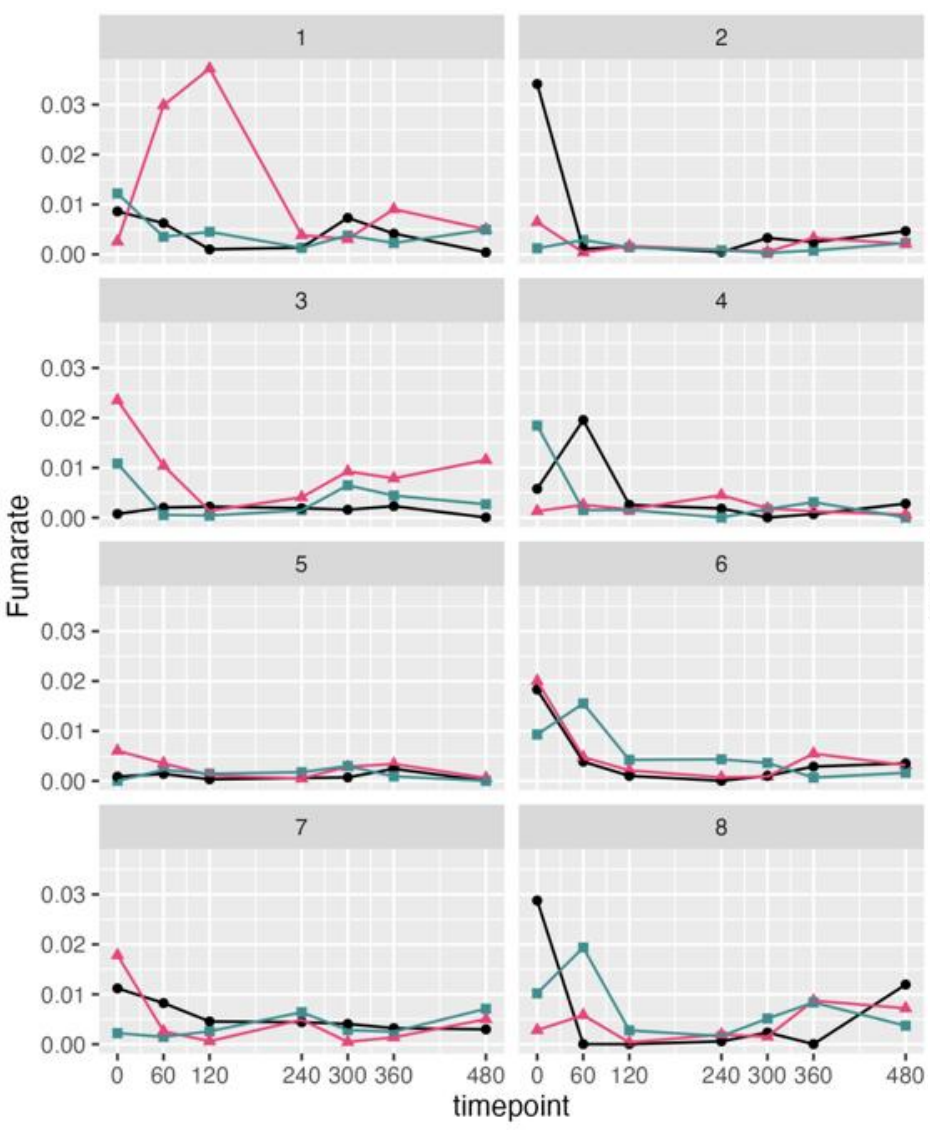
Fumarate
timepoint

**(i)**

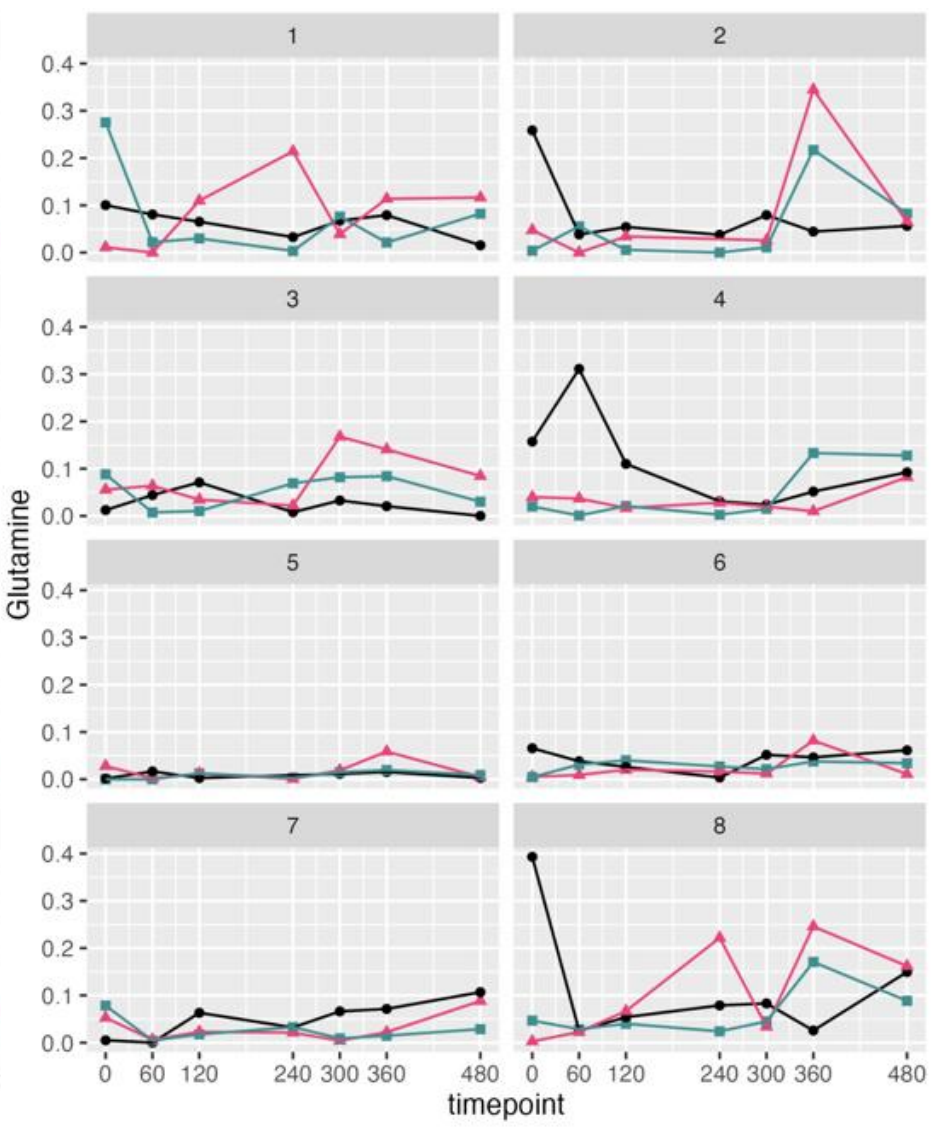
Glutamine
timepoint

**(j)**

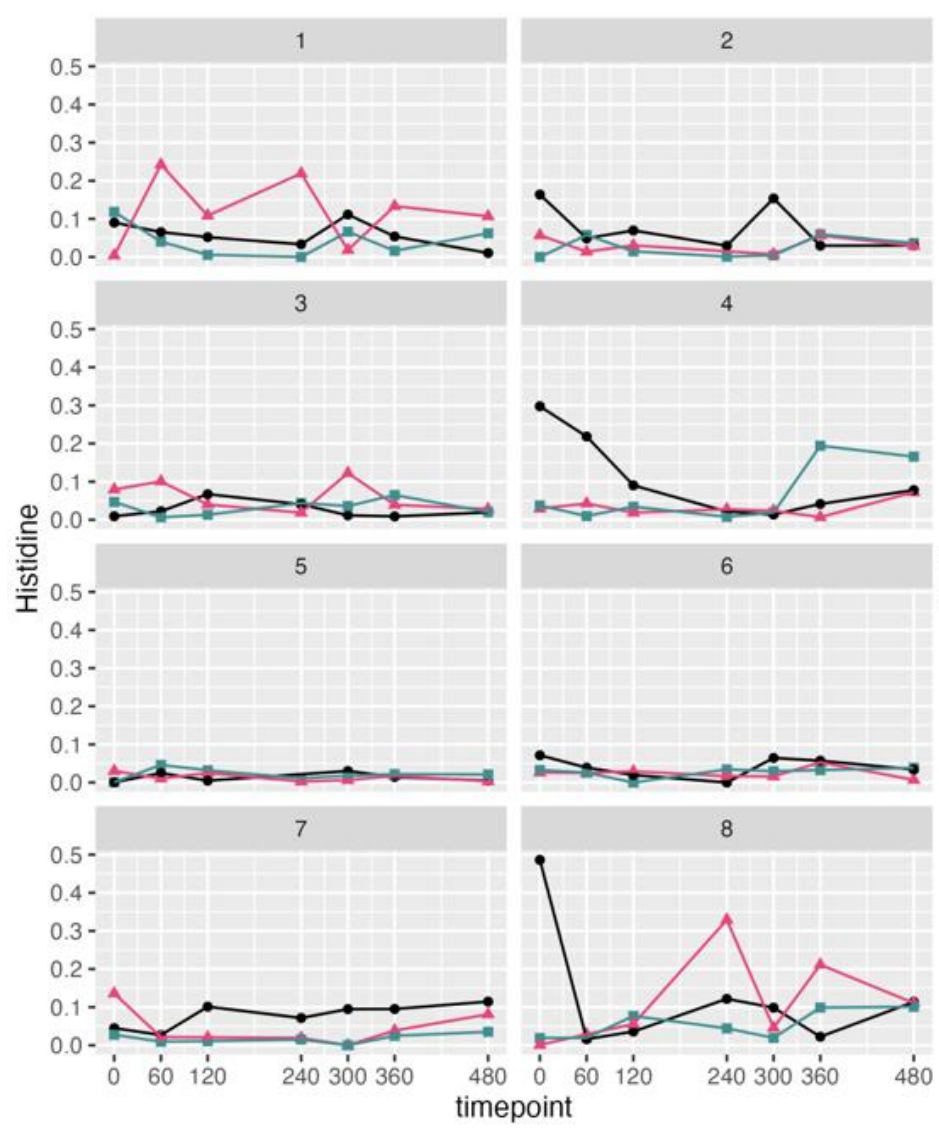
Histidine
timepoint

**(k)**

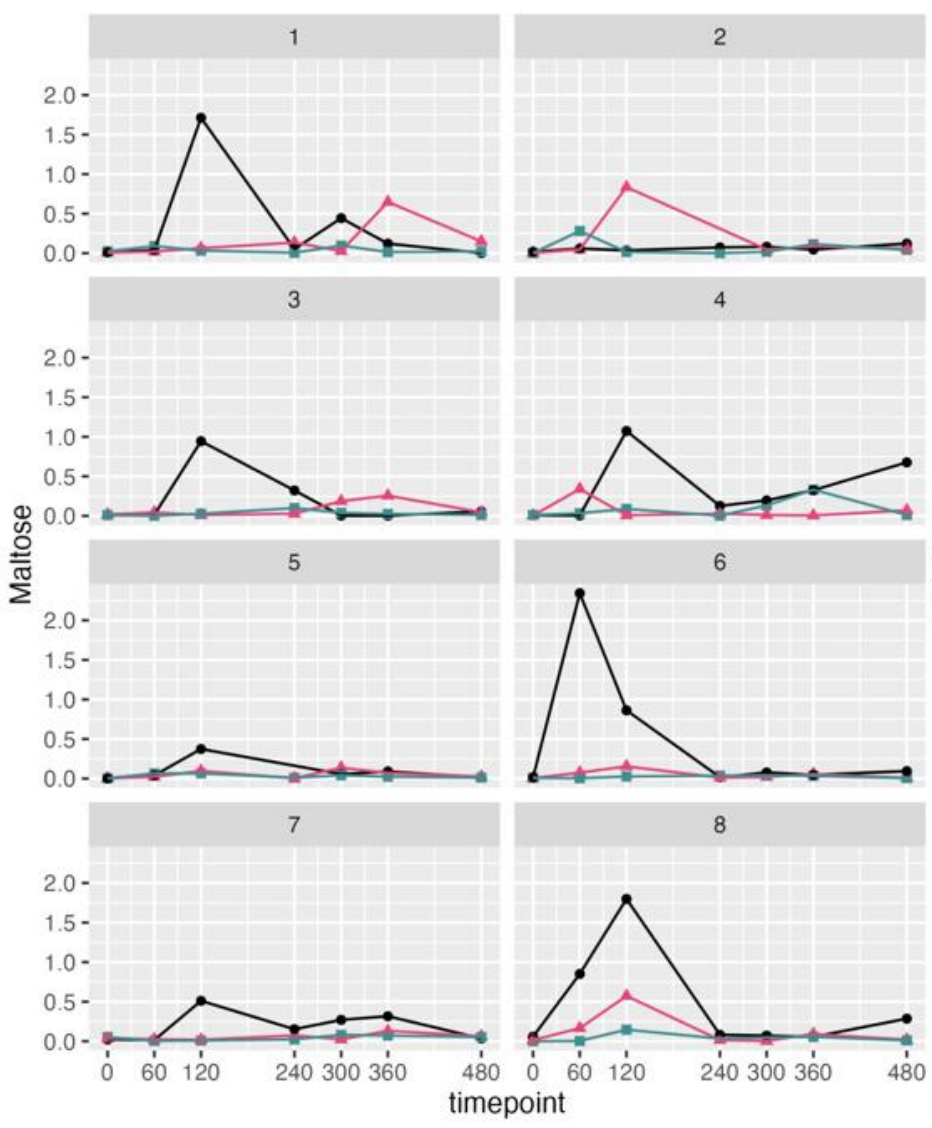
Maltose
timepoint

**(l)**

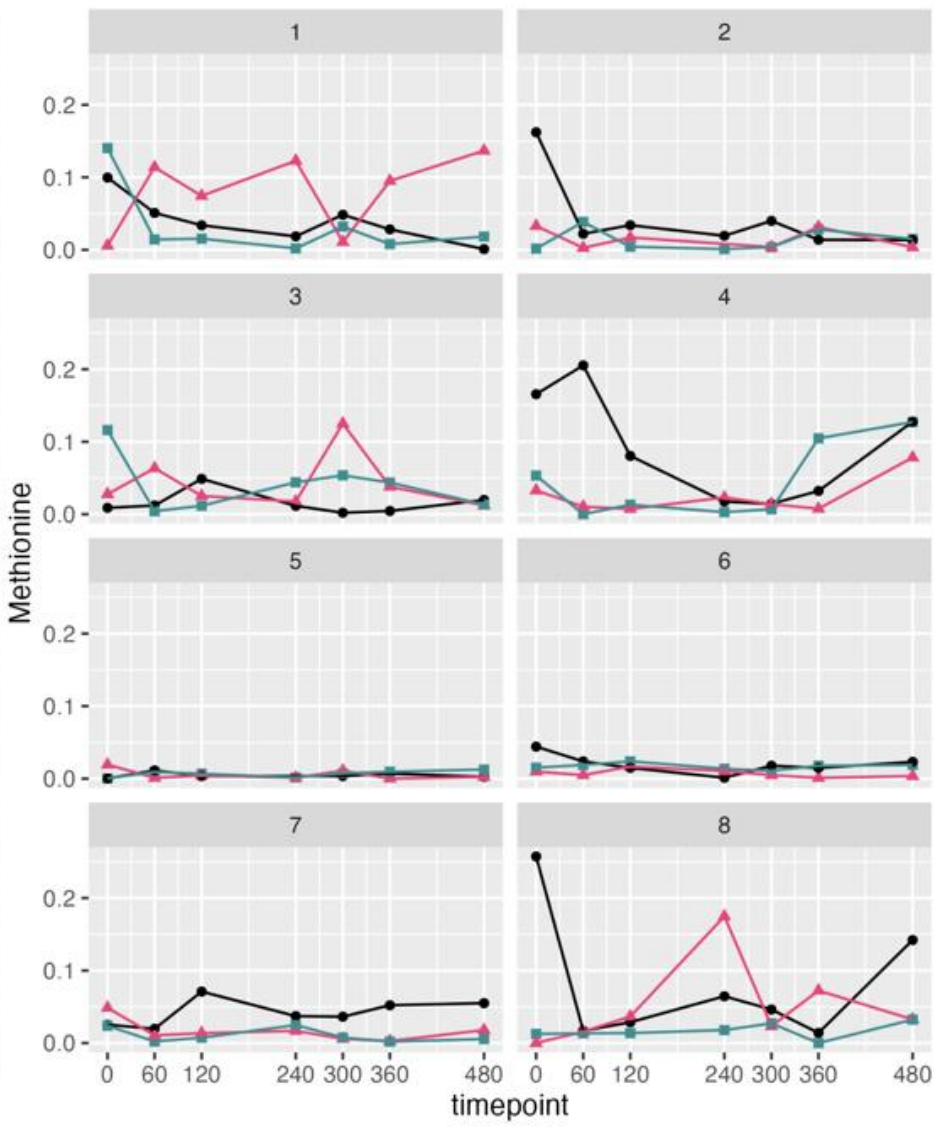
Methionine
timepoint

**(m)**

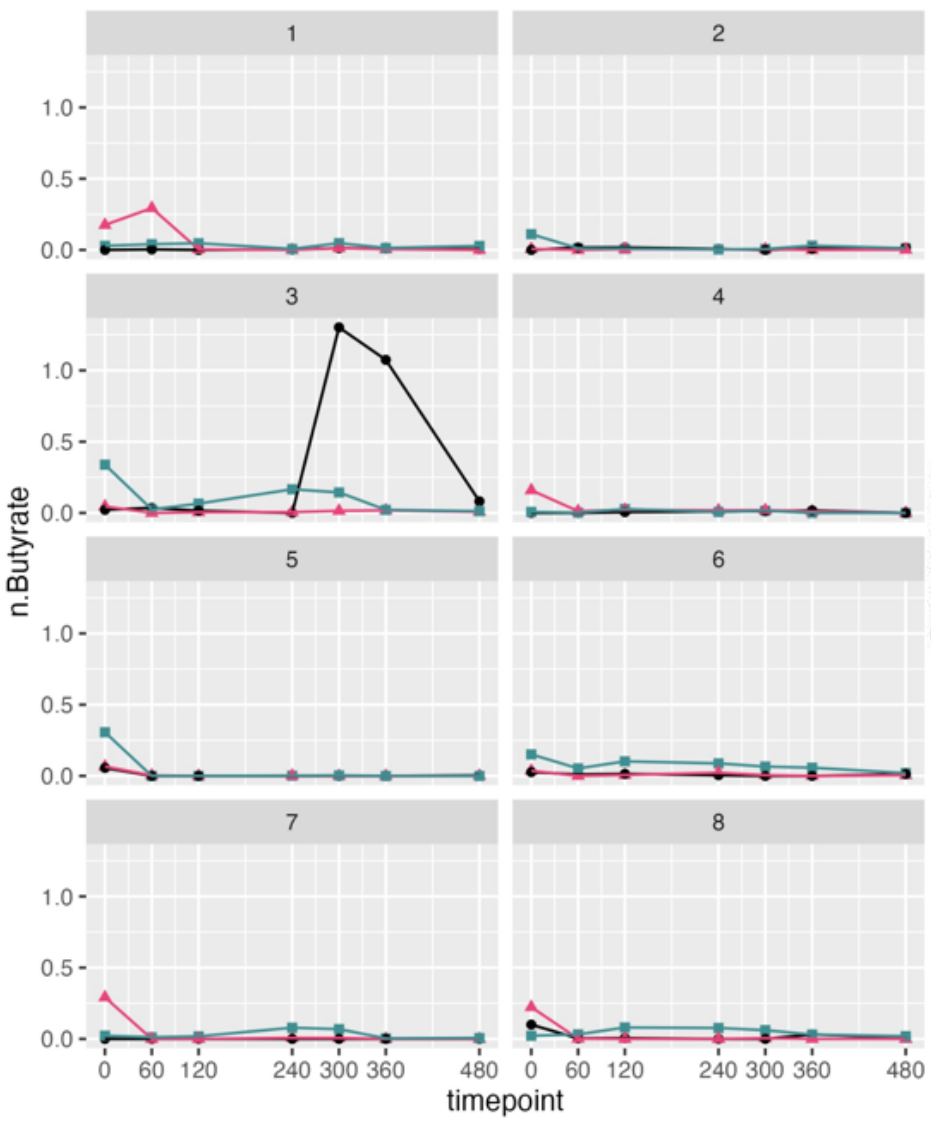
1
2
3
4
5
6
7
8
n.Butyrate
timepoint
0 60 120 240 300 360 480

**(n)**

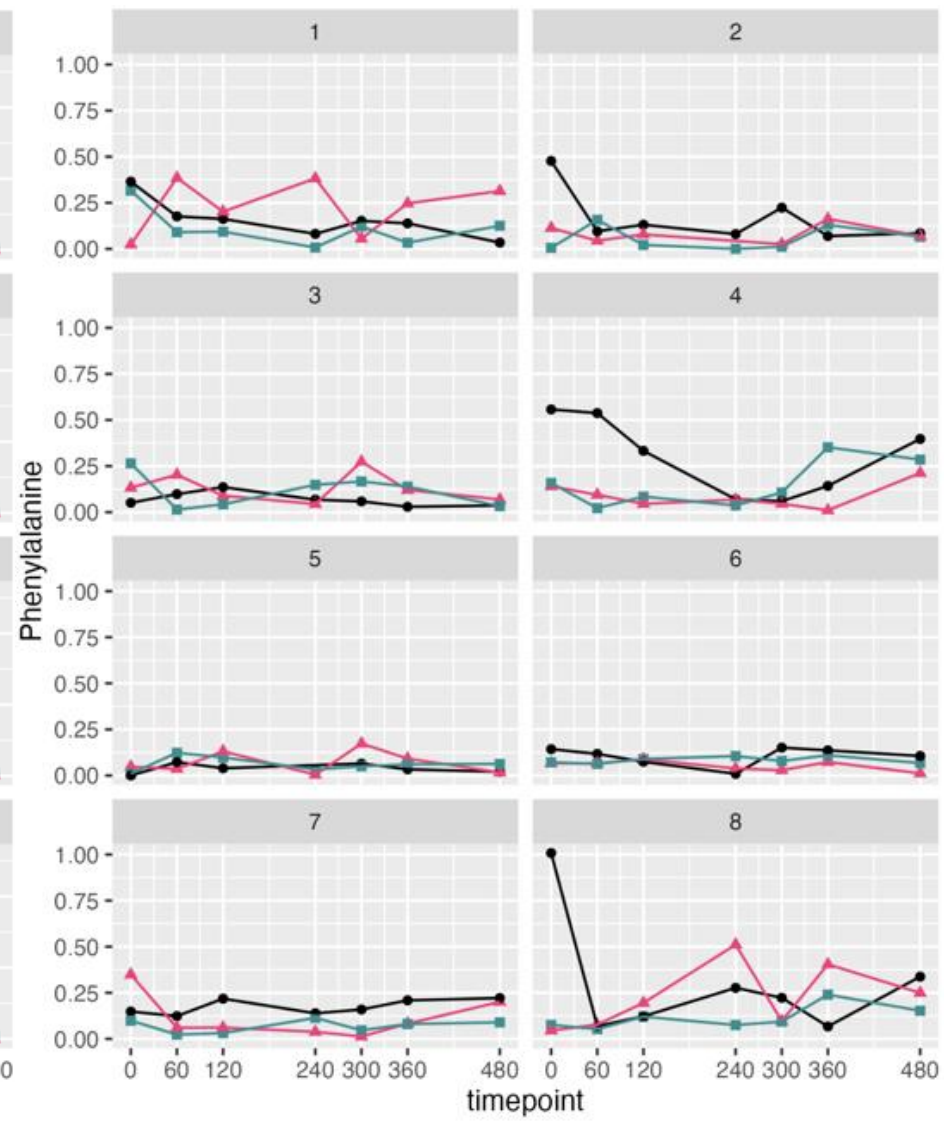
1
2
3
4
5
6
7
8
Phenylalanine
timepoint
0 60 120 240 300 360 480

**(o)**

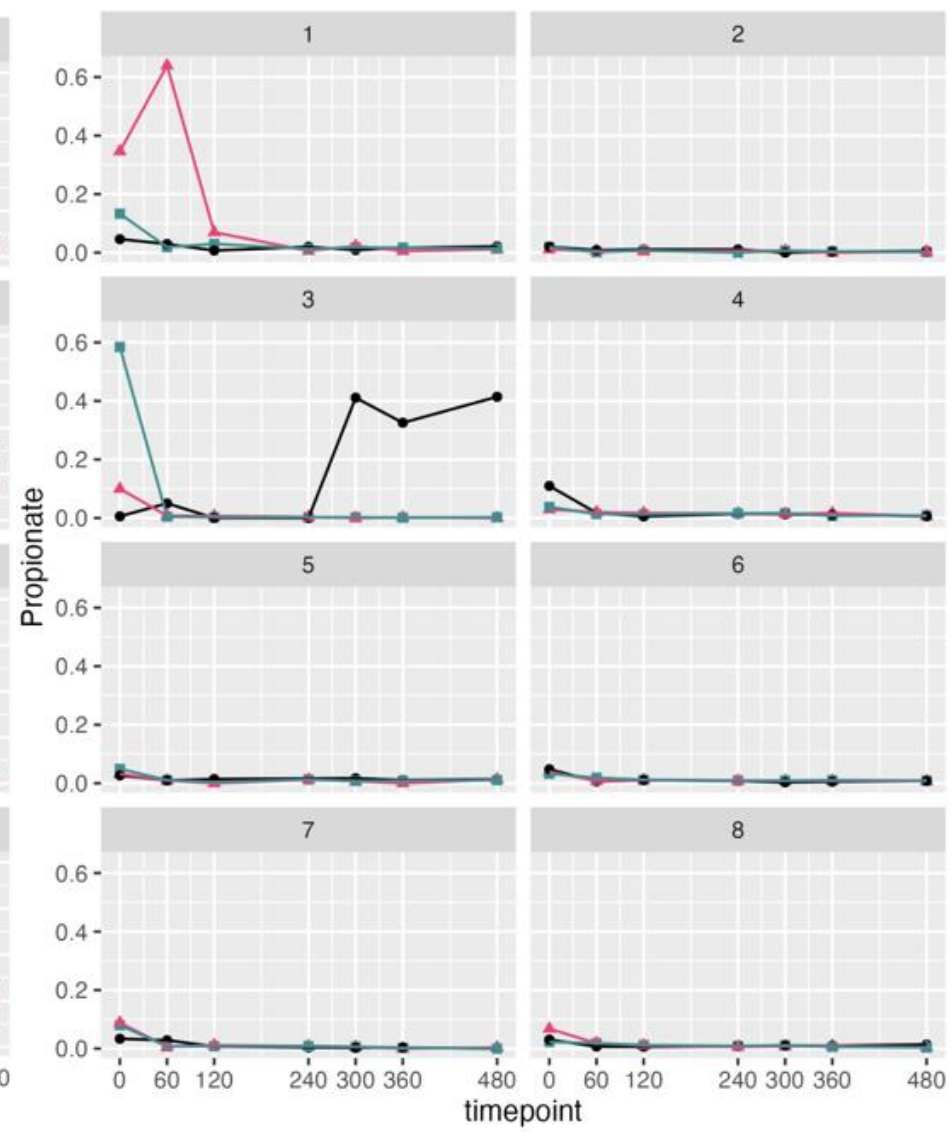
1
2
3
4
5
6
7
8
Propionate
timepoint
0 60 120 240 300 360 480

**(p)**

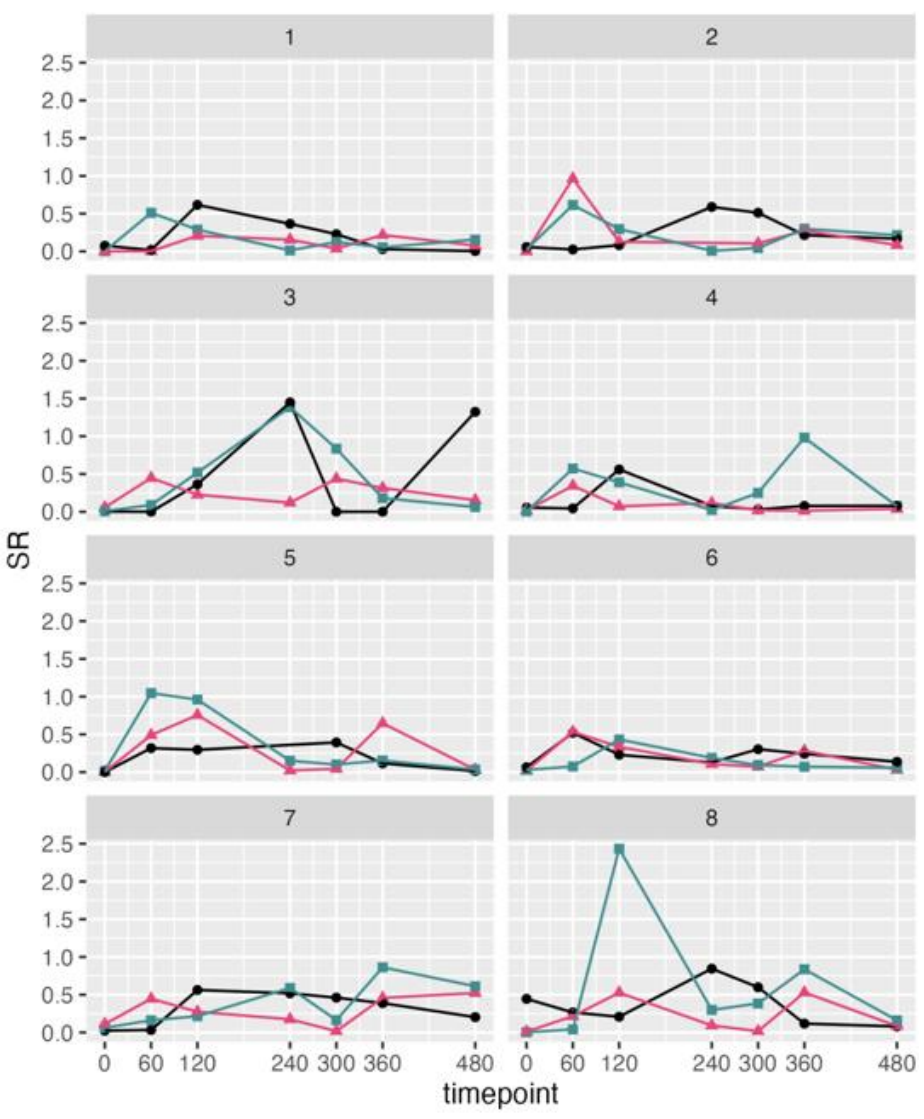
1
2
3
4
5
6
7
8
SR
timepoint
0 60 120 240 300 360 480

**(q)**

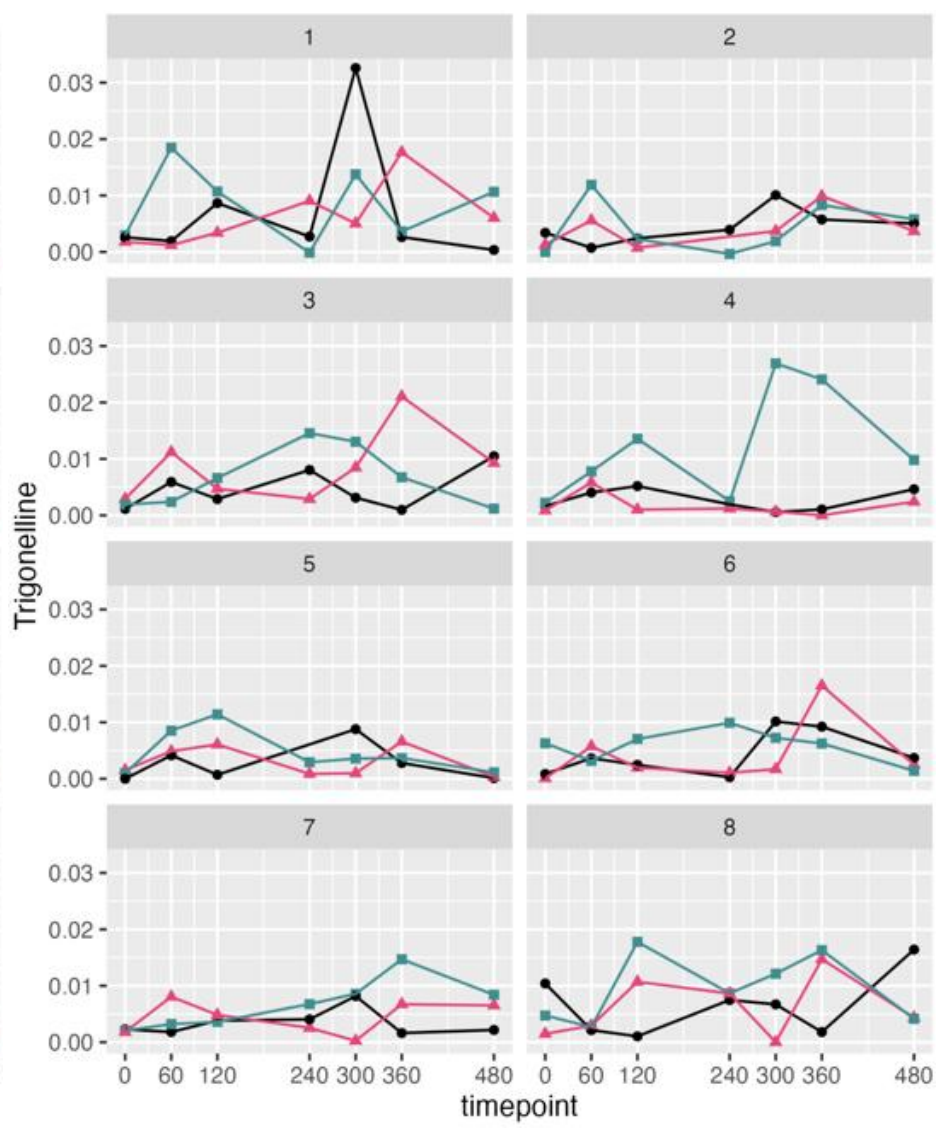
1
2
3
4
5
6
7
8
Trigonelline
timepoint
0 60 120 240 300 360 480

**(r)**

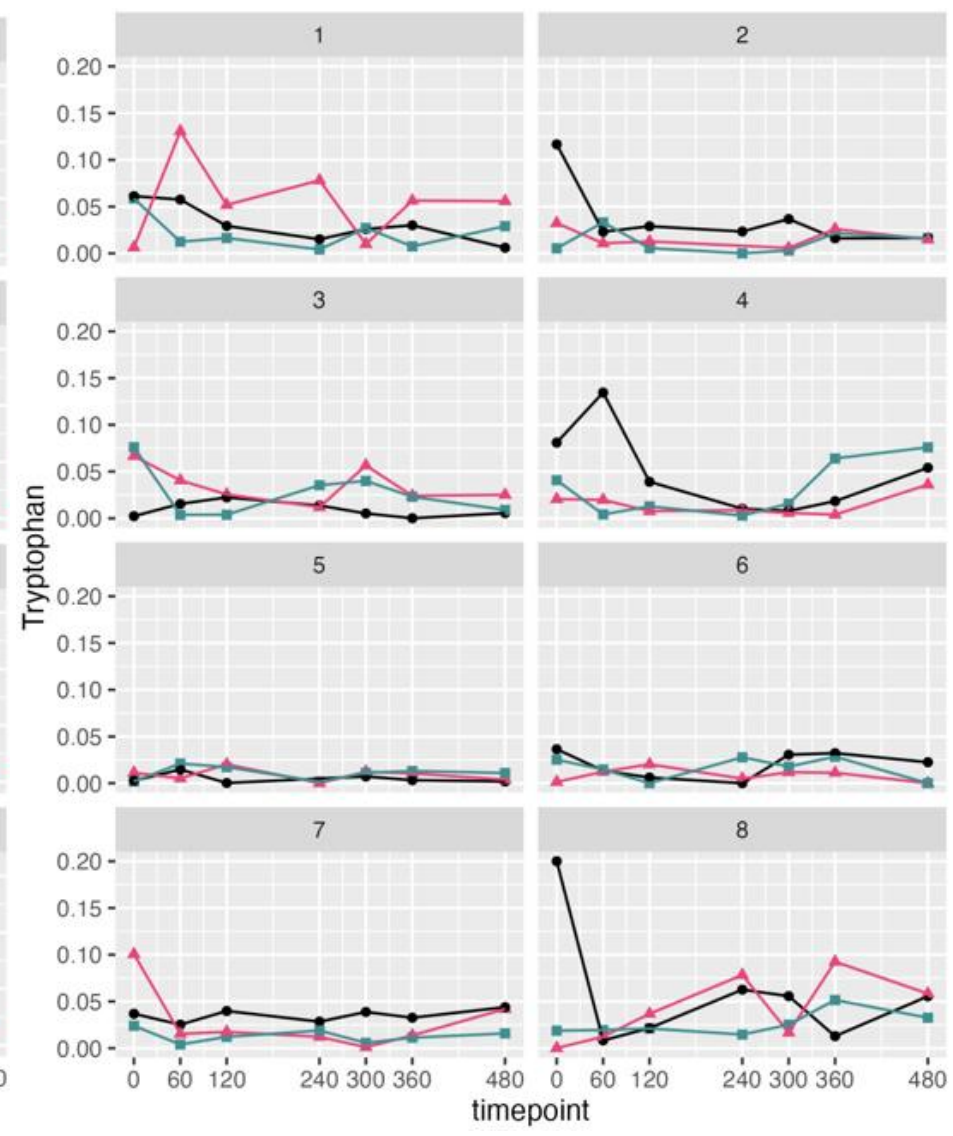
1
2
3
4
5
6
7
8
Tryptophan
timepoint
0 60 120 240 300 360 480

**(s)**

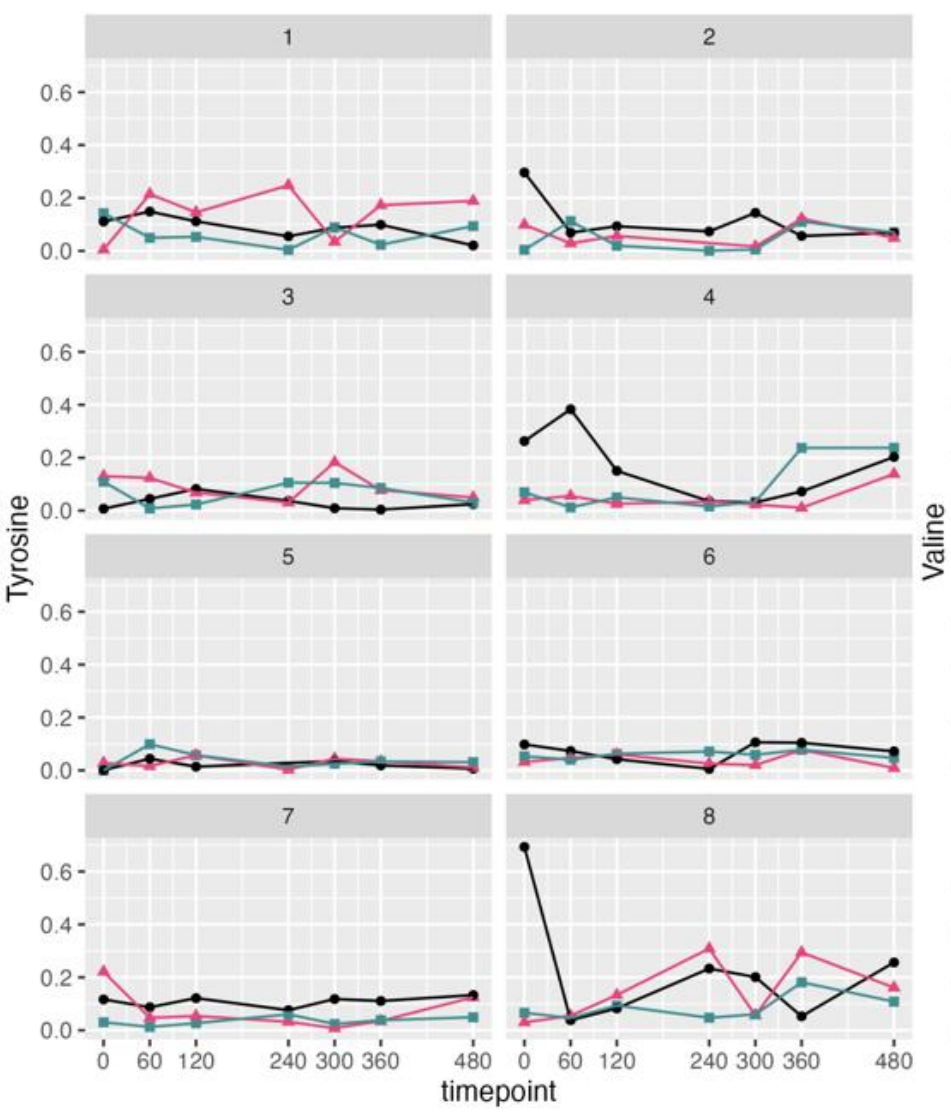
1
2
3
4
5
6
7
8
Tyrosine
timepoint
0 60 120 240 300 360 480

**(t)**

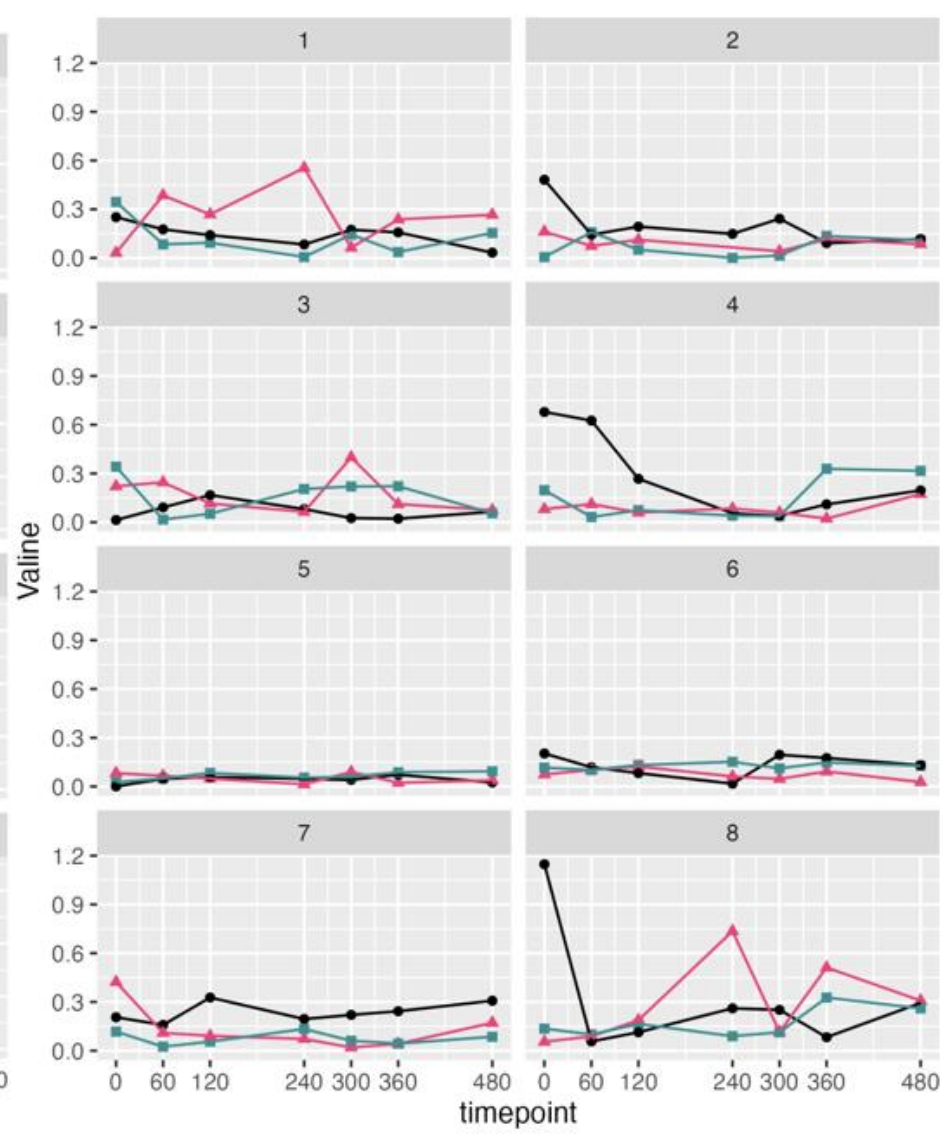
1
2
3
4
5
6
7
8
Valine
timepoint
0 60 120 240 300 360 480

**Supplementary figure 7: Metabolite concentrations across diets, gastrointestinal locations and timepoints.** Each point represents one participant measurement. Boxes show medians and interquartile ranges, whiskers extend to the most extreme value within 1.5 × the interquartile range, with all observations shown individually. Repeated measurements were analysed using linear mixed-effects models (concentration ~ diet * timepoint + (1 | patient_id)) with diet, timepoint and their interaction as fixed effects and participant as a random intercept. Pairwise estimated-marginal-mean contrasts were Holm-adjusted within each timepoint. *$P \leq 0.05$, **$P \leq 0.01$ and ***$P \leq 0.001$; ns, not significant.

**(a)** n_butyrate

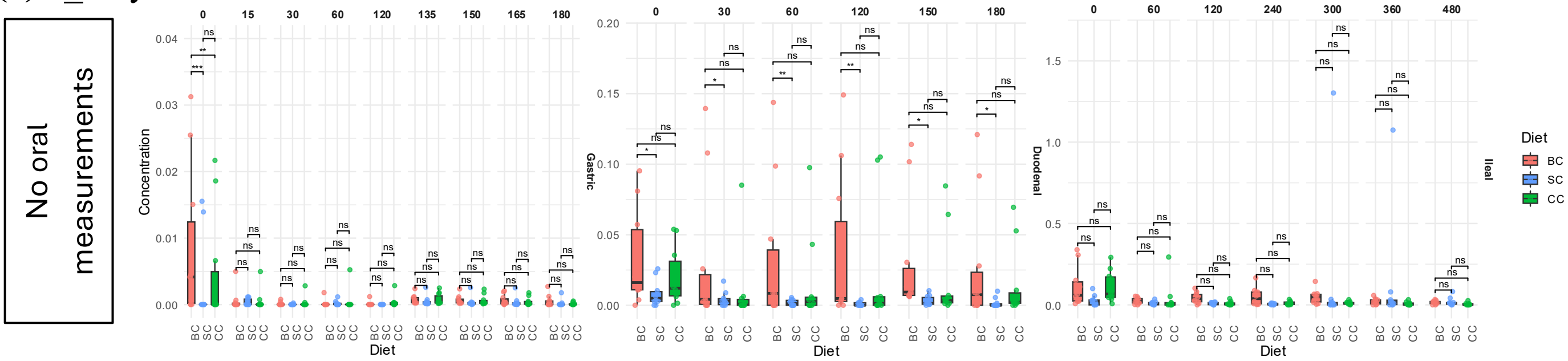


**(b)** valine

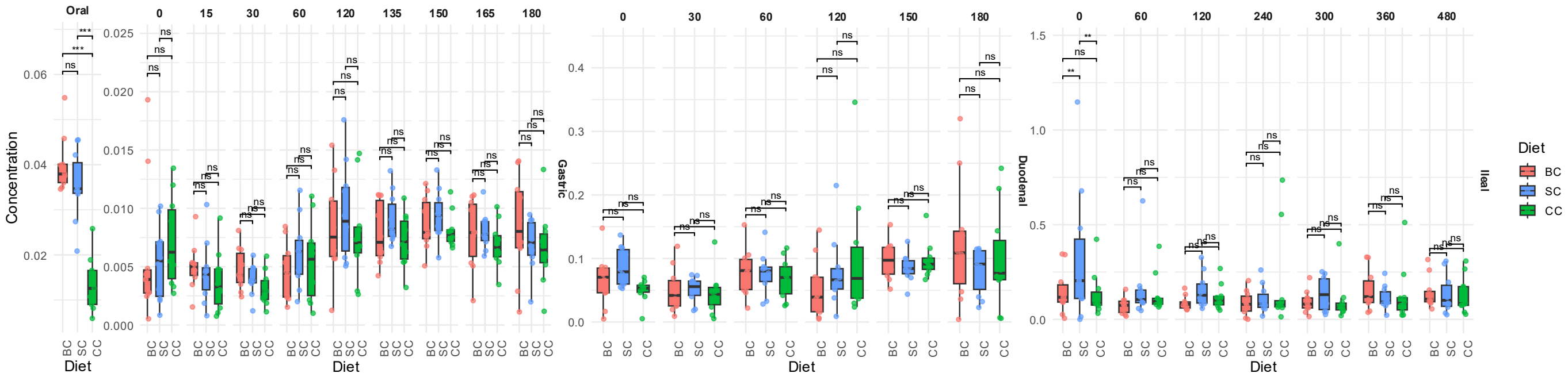


**(c)** propionate

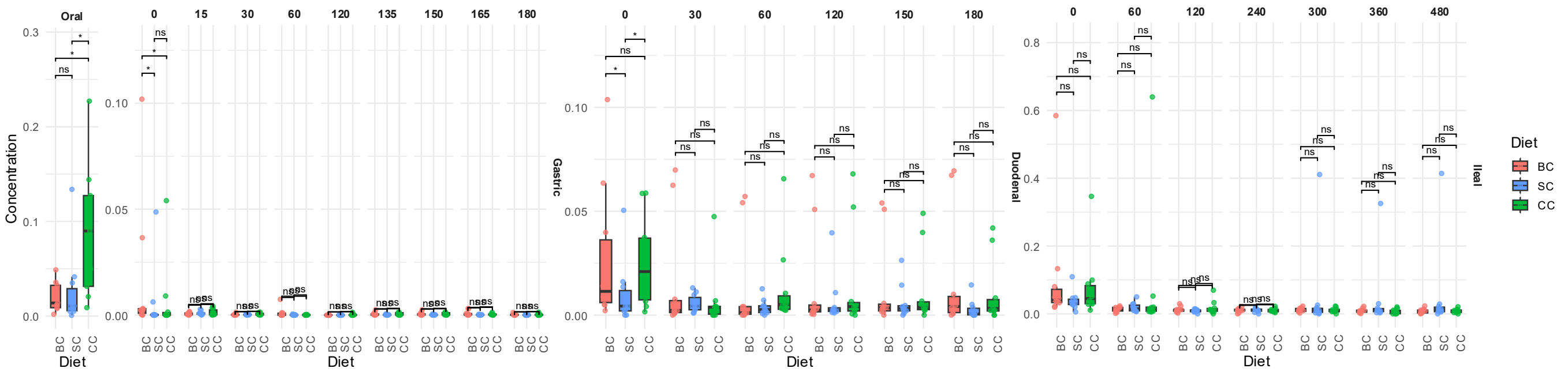


**(d)** alanine

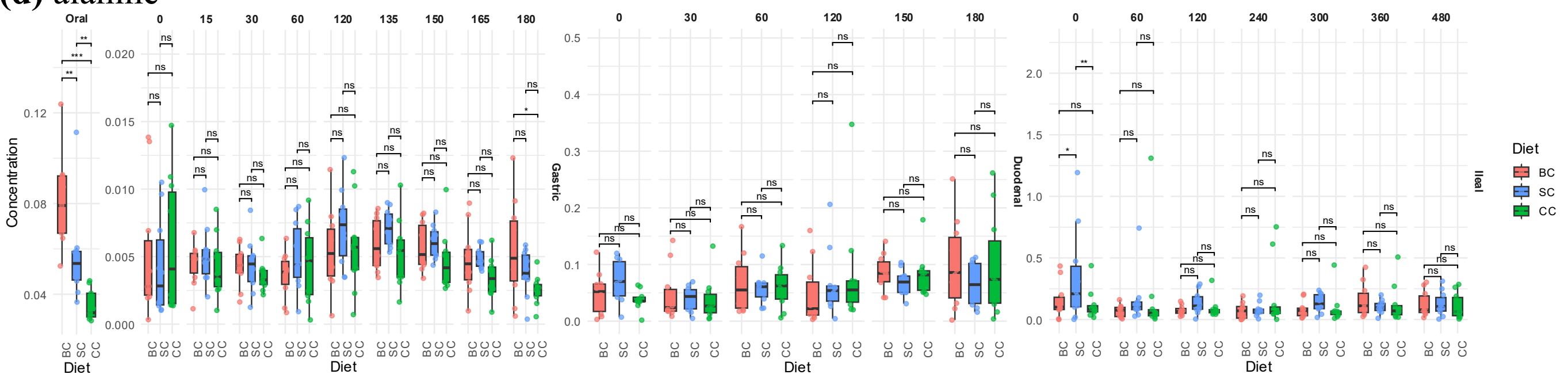

**(e)** acetate

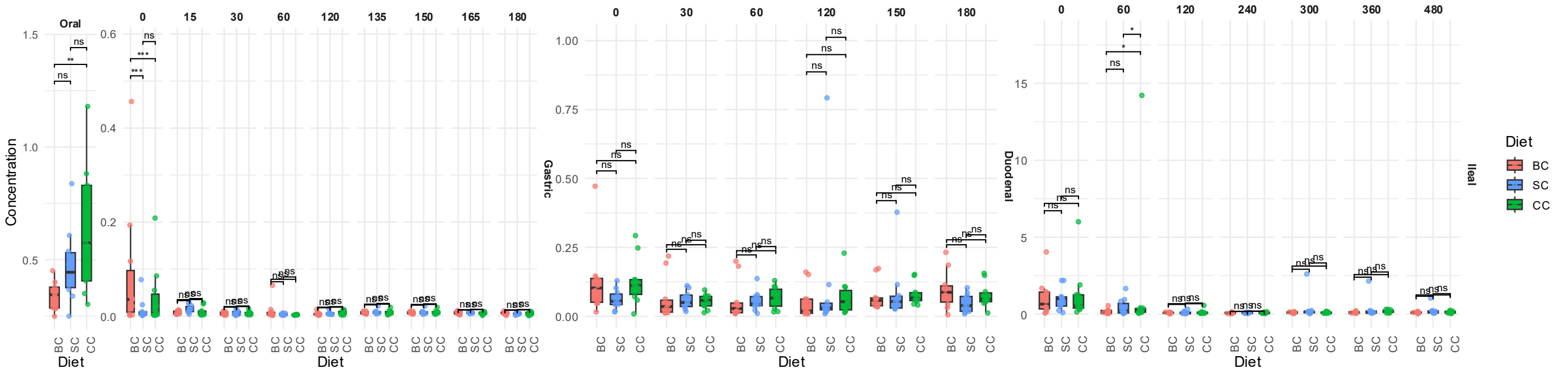


**(f)** glutamine

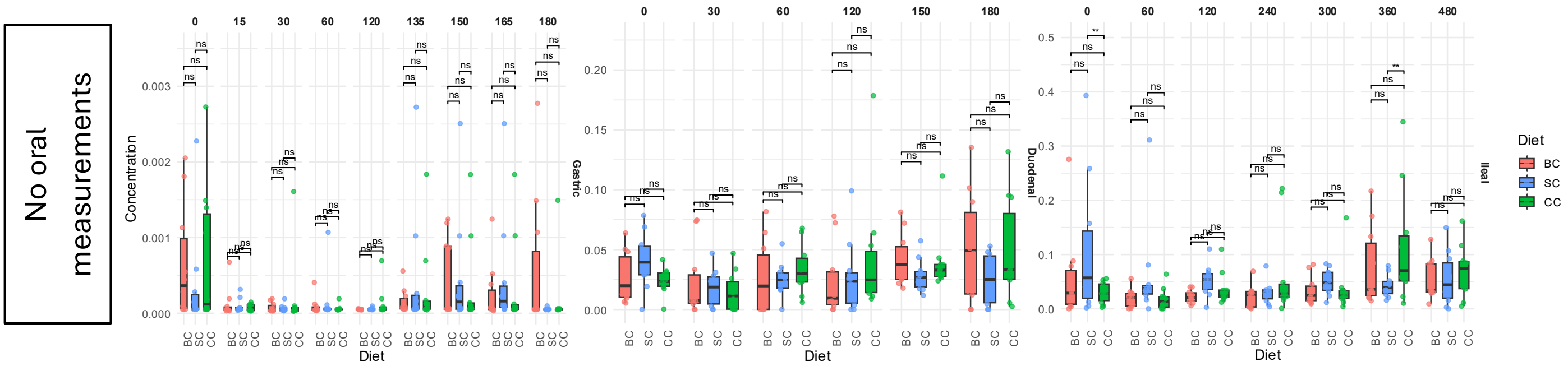


**(g)** methionine

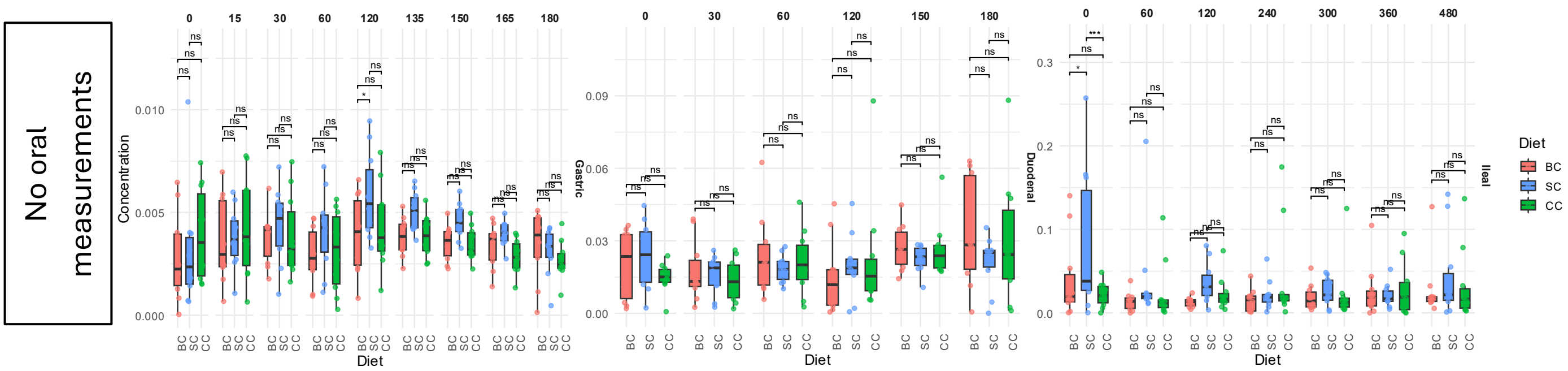


**(h)** aspartate

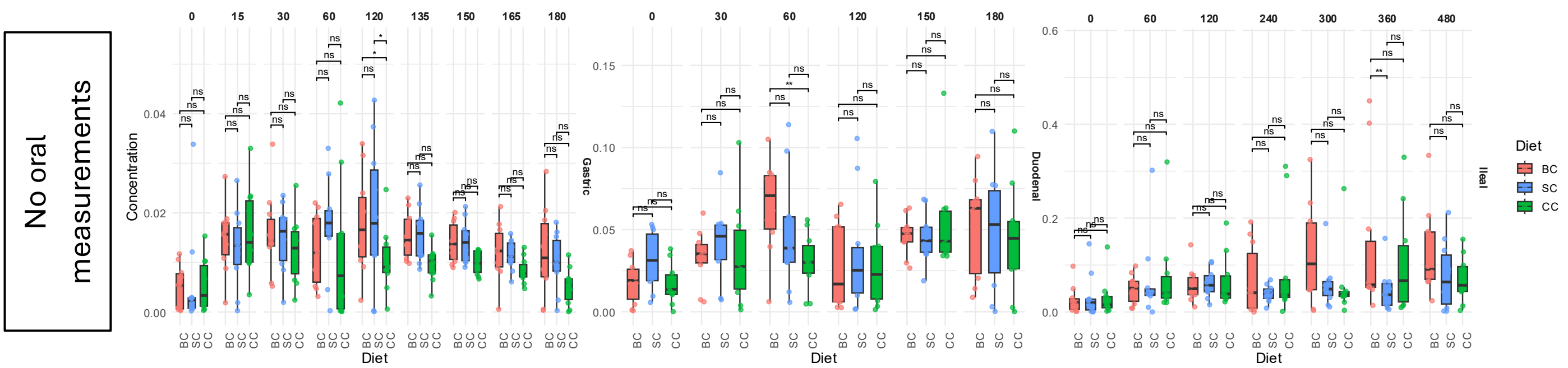

**(i)** asparagine

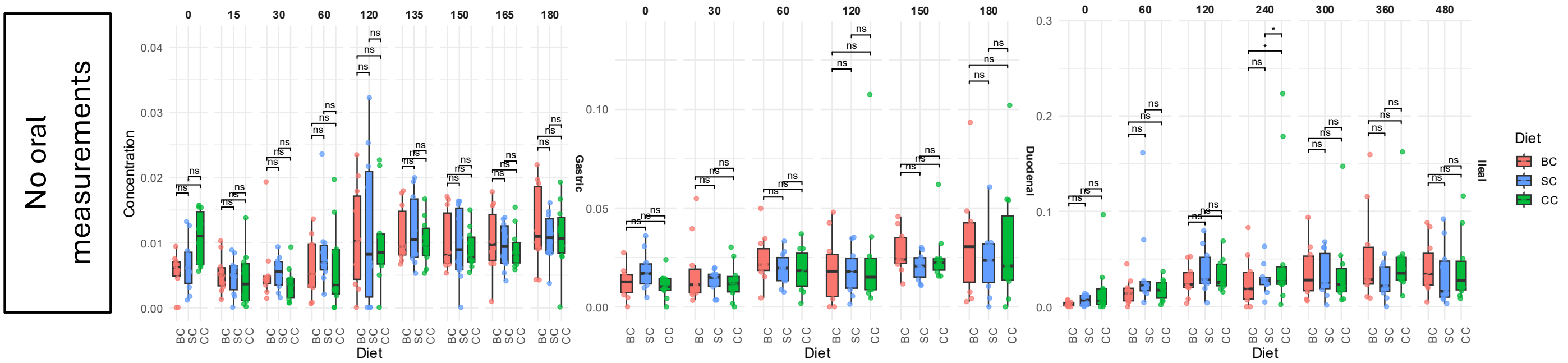


**(j)** tyrosine

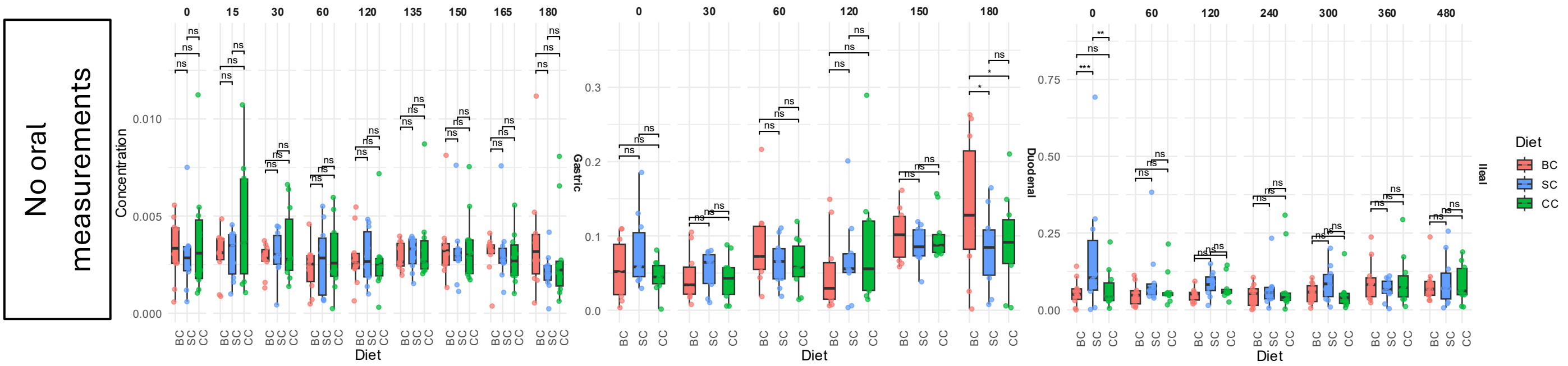


**(k)** phenylalanine

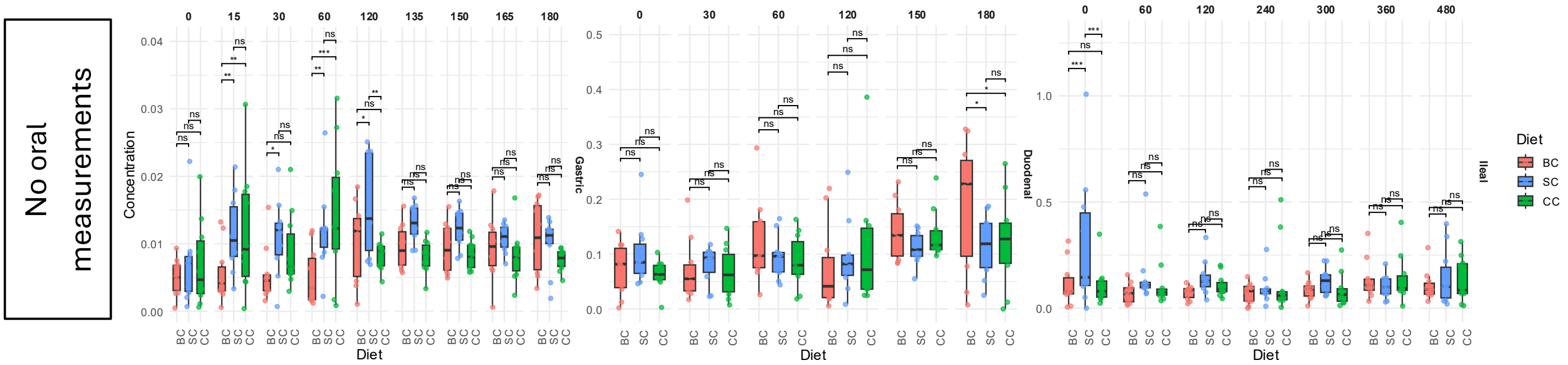


**(l)** Raffinose and stachyose

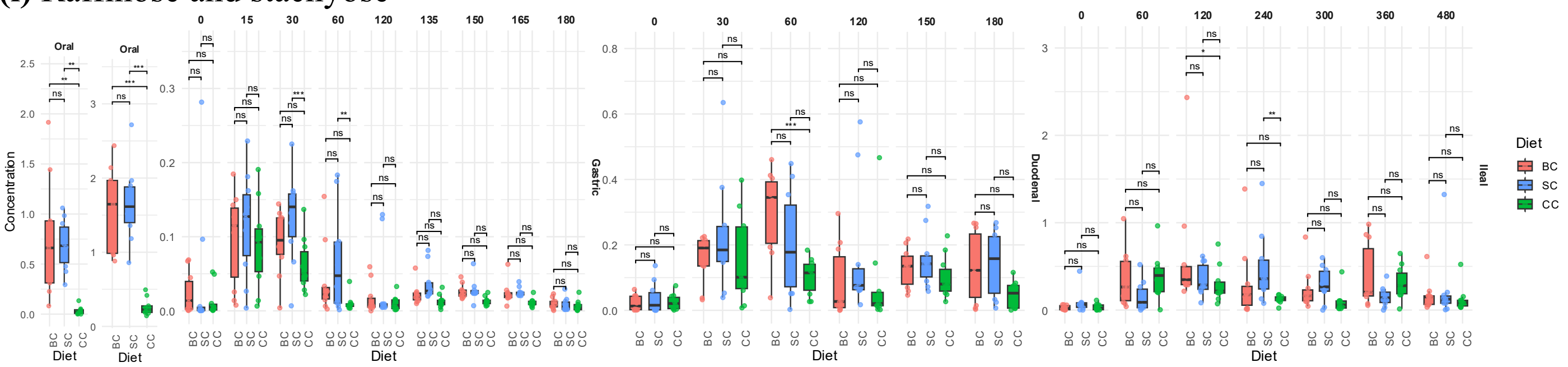


**(m)** trigonelline

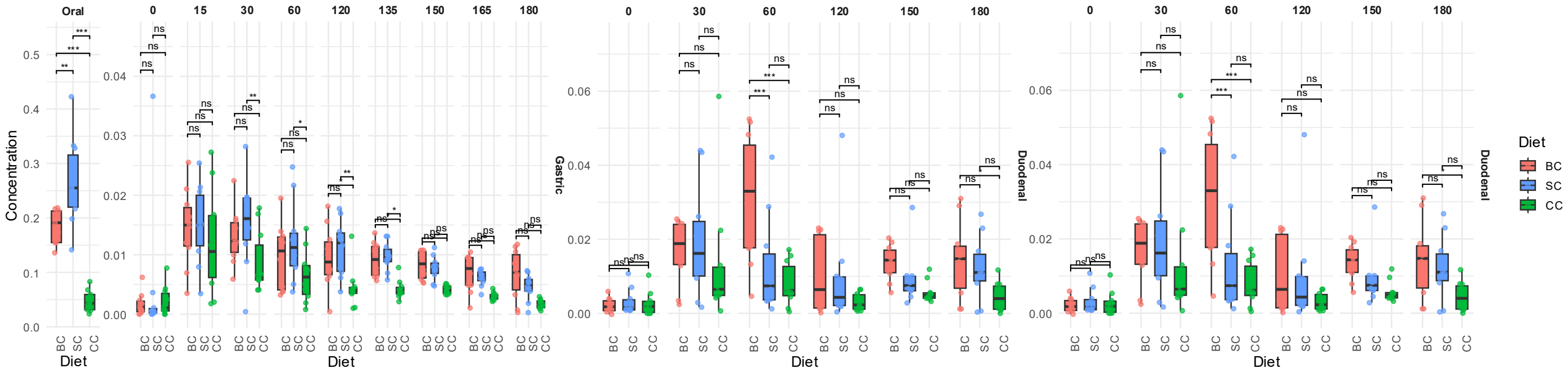


**(n)** fumarate

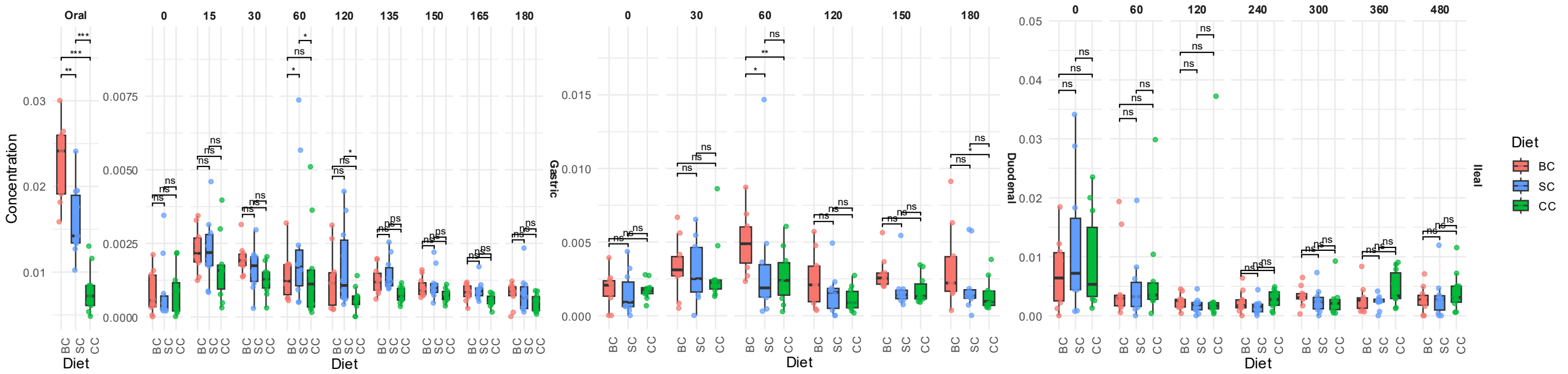


**(o)** tryptophan

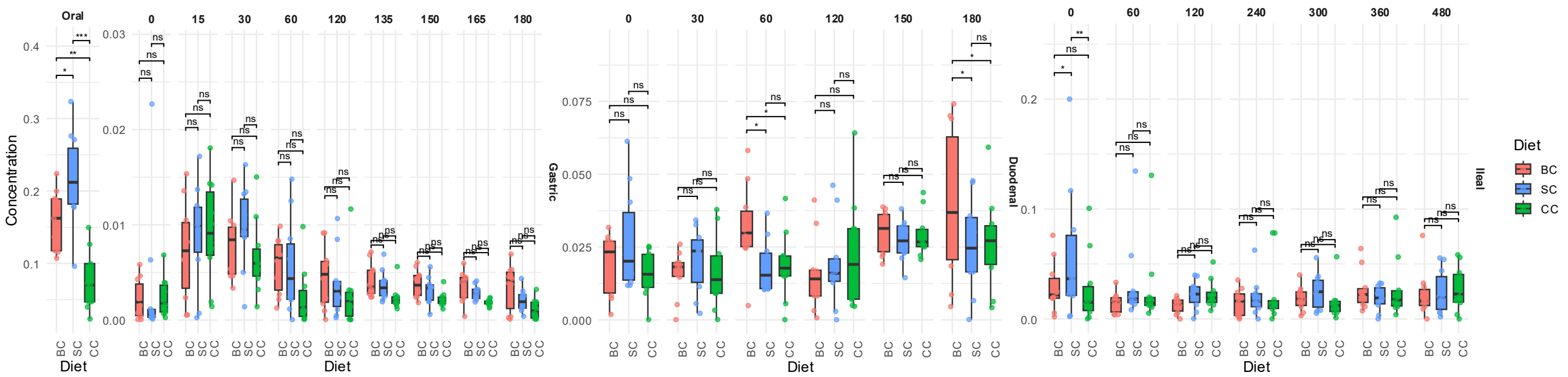


**(p)** histidine

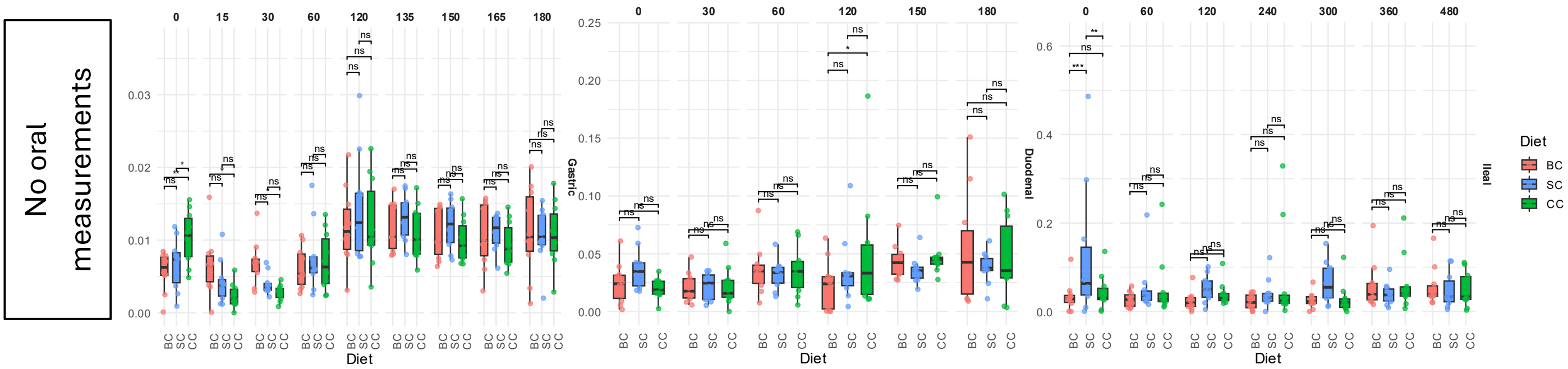


**(q)** formate

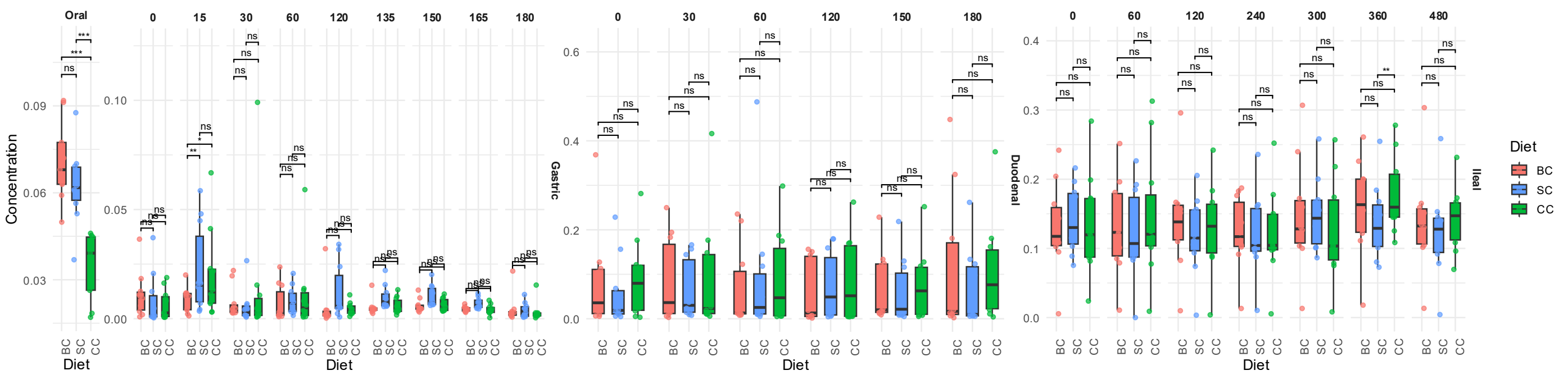

**(r)** alpha_glucose_alpha_maltose

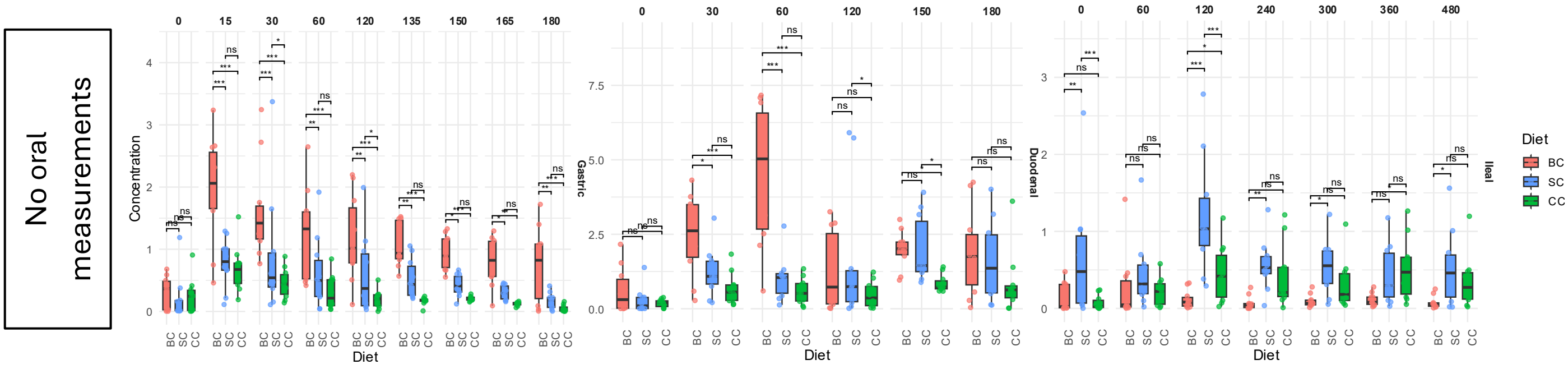


**(s)** beta_glucose

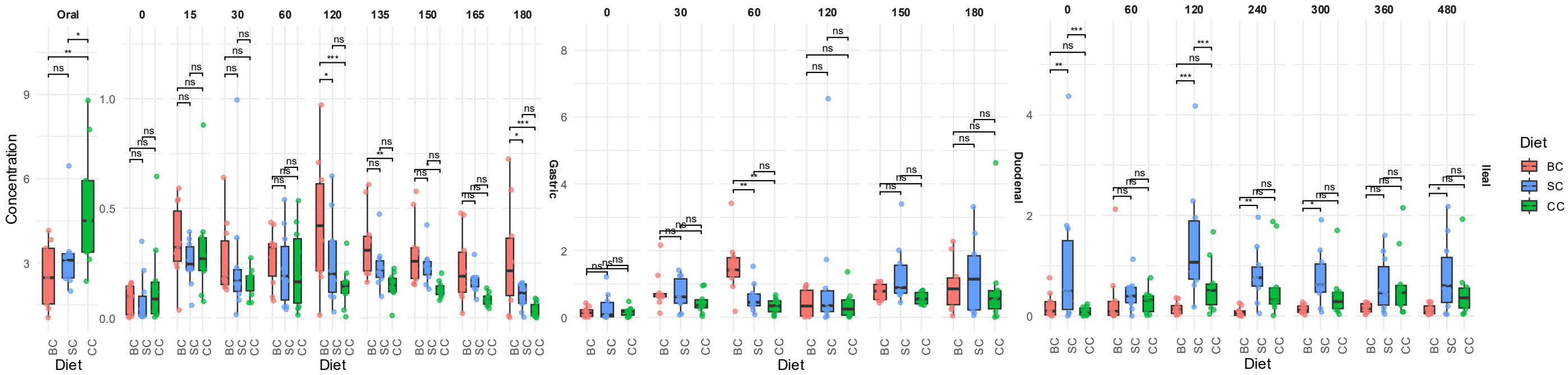


**(t)** beta_maltose

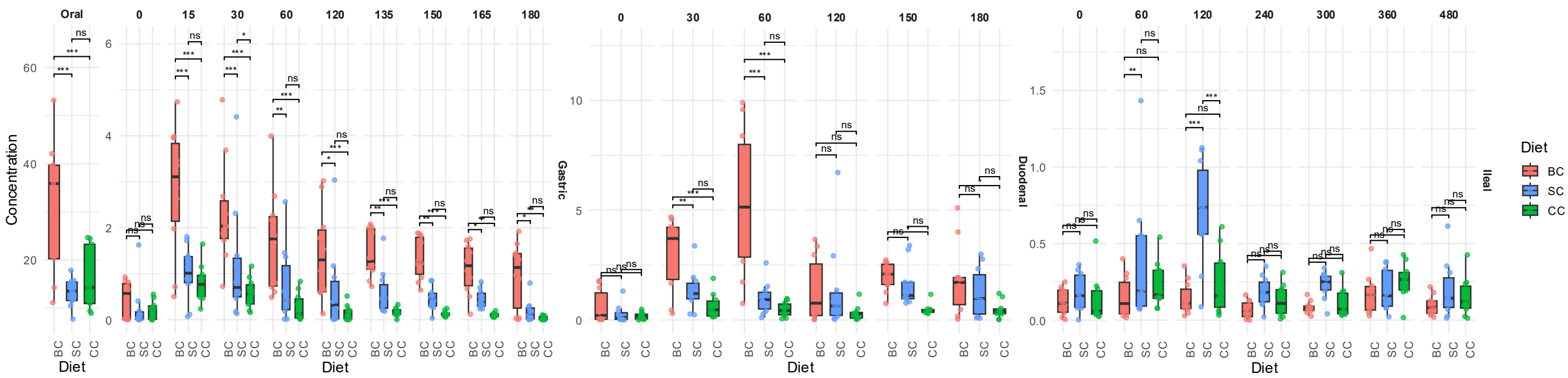


**(u)** bile acid: tcdca

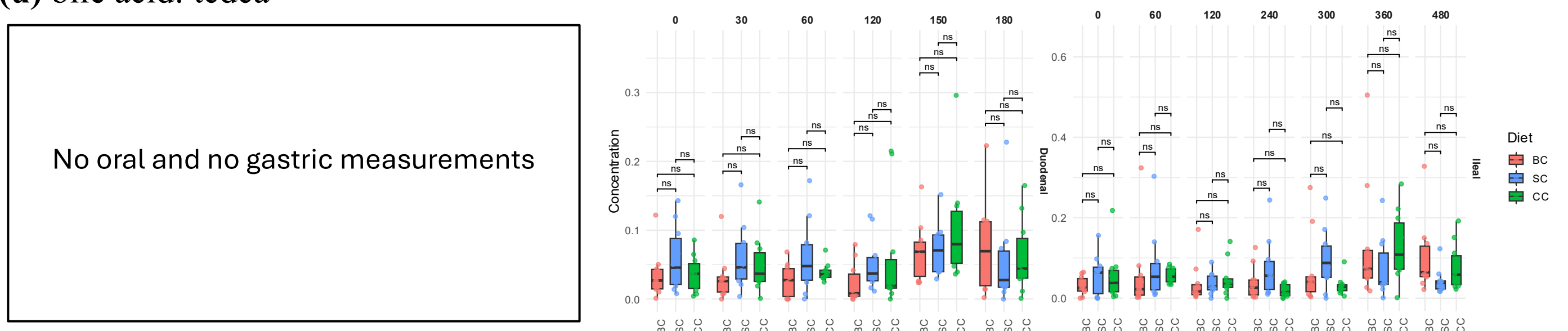

(v) bile acid: gcdca

No oral and no gastric measurements

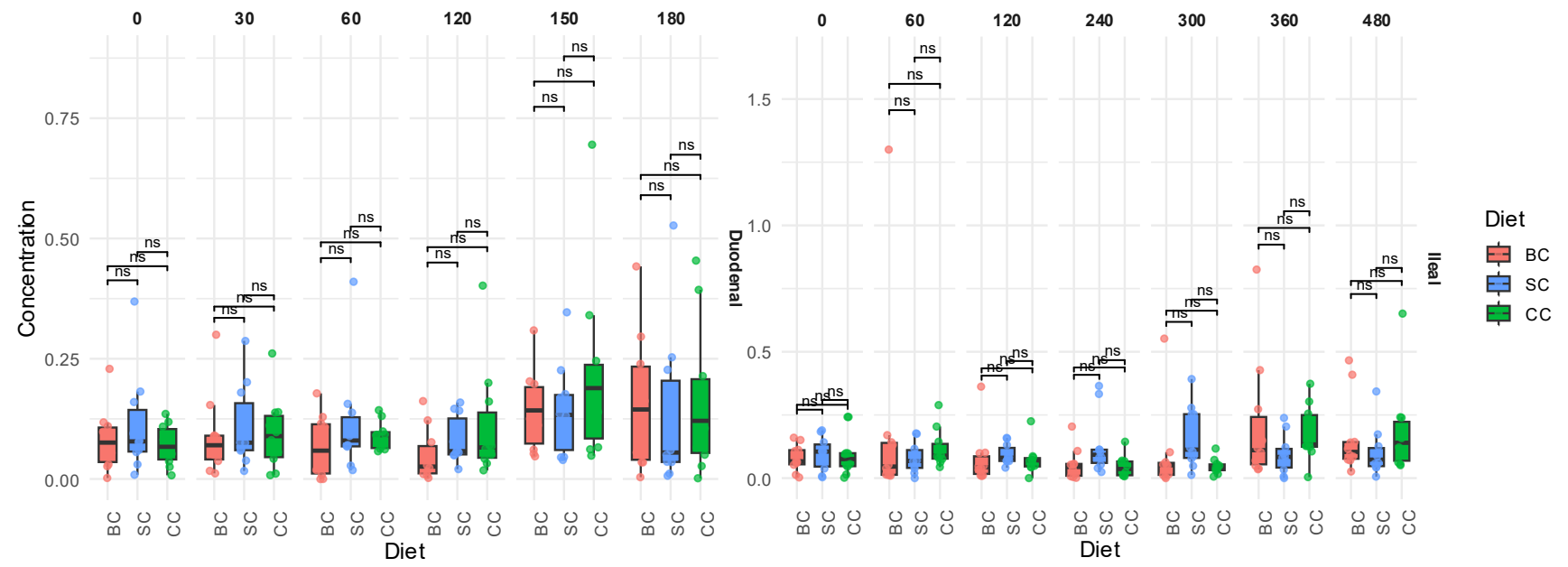


(w) bile acid: tdca

No oral and no gastric measurements

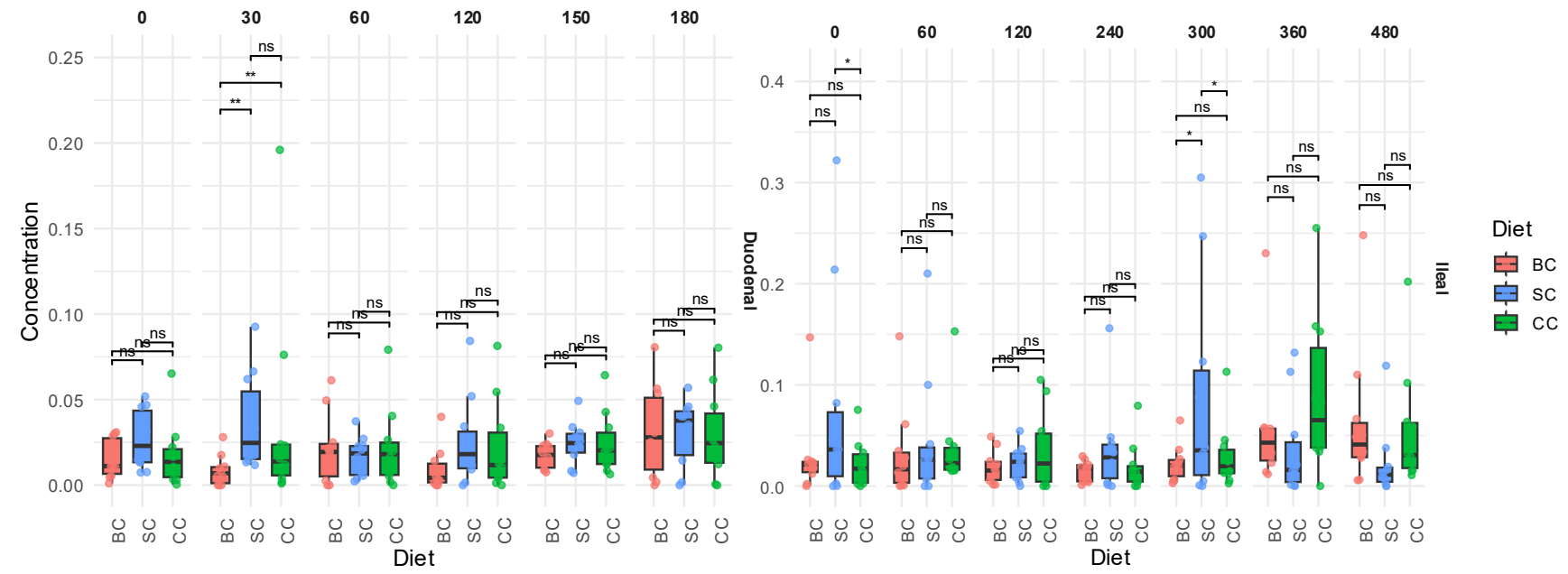


(x) bile acid: gdca

No oral and no gastric measurements

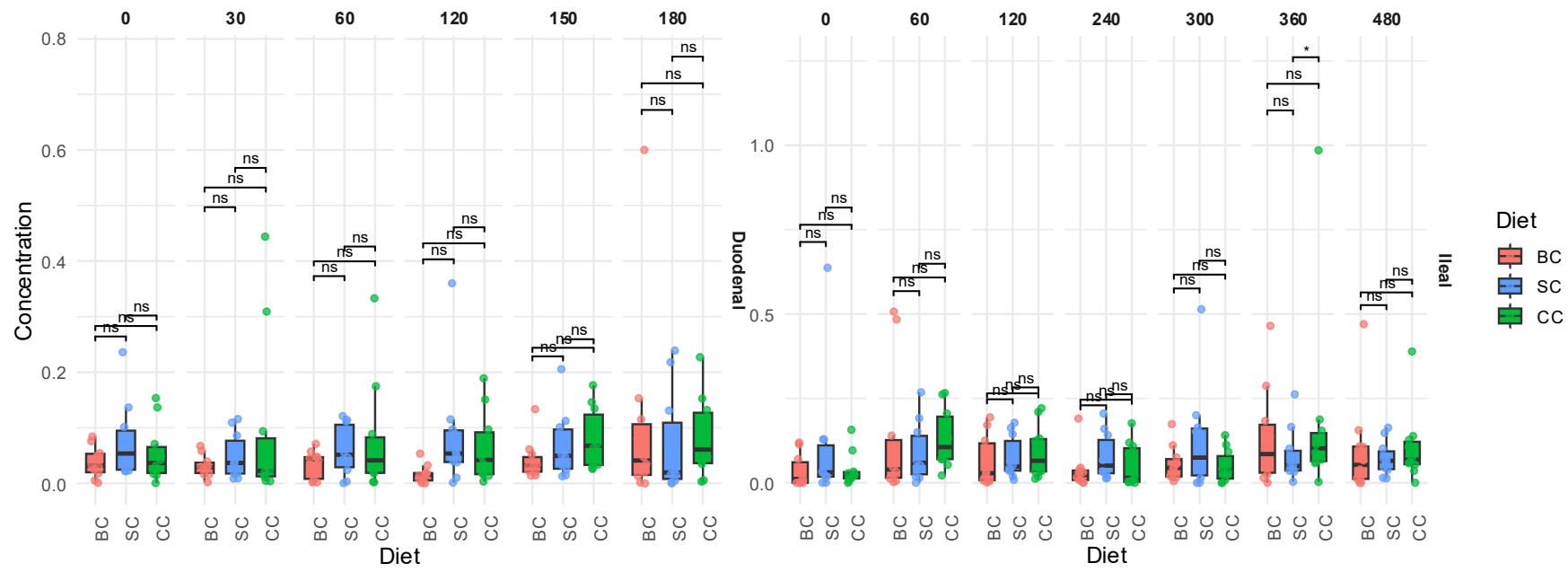


(y) bile acid: tca

No oral and no gastric measurements

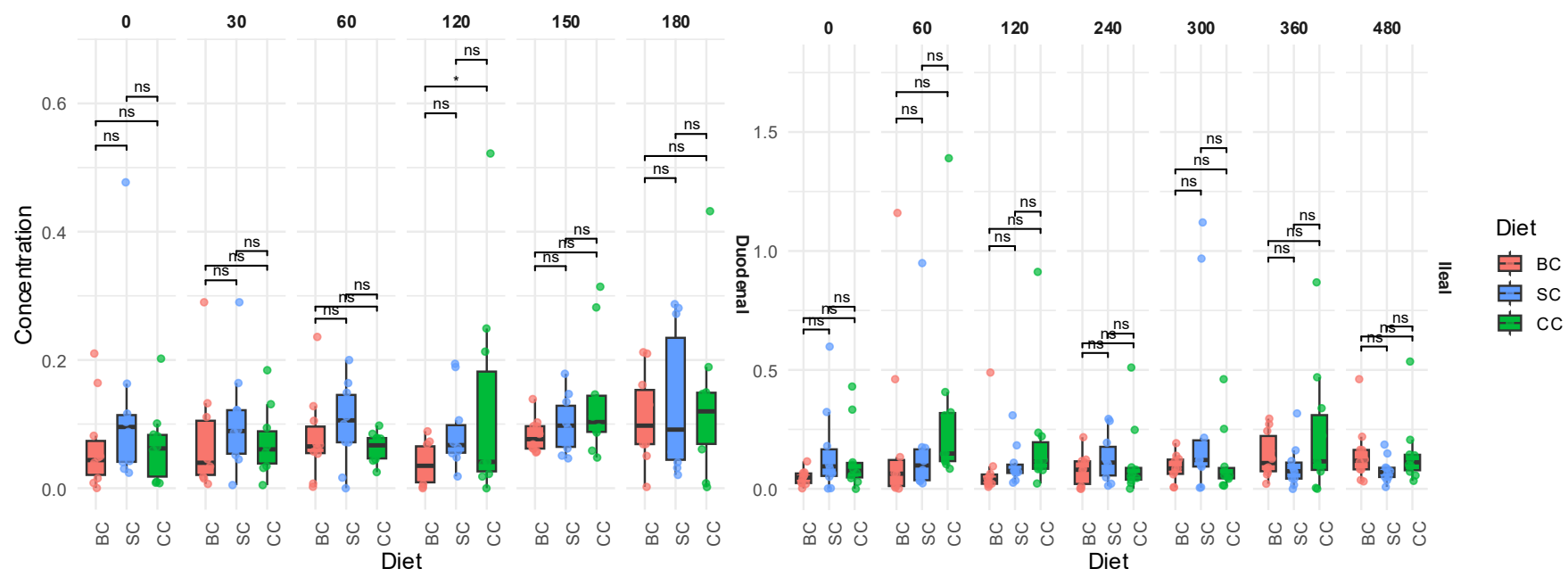

## (z) bile acid: gca

No oral and no gastric measurements

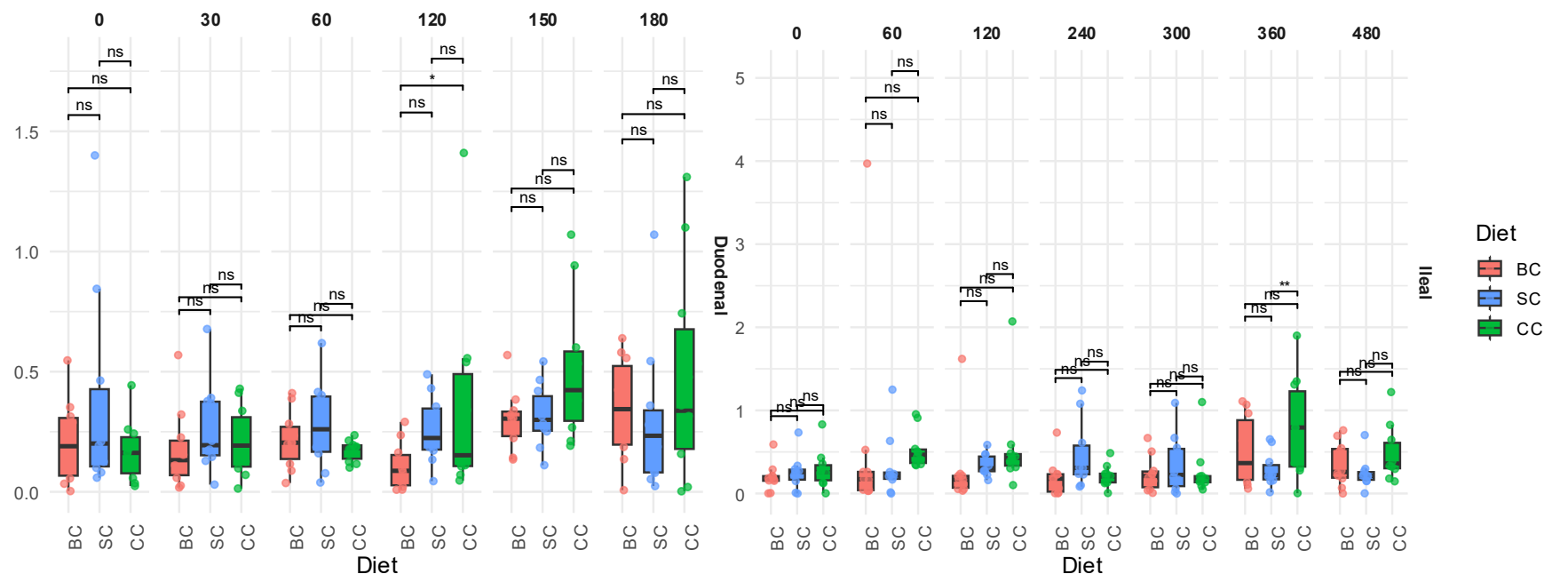


## (ai) ciceritol

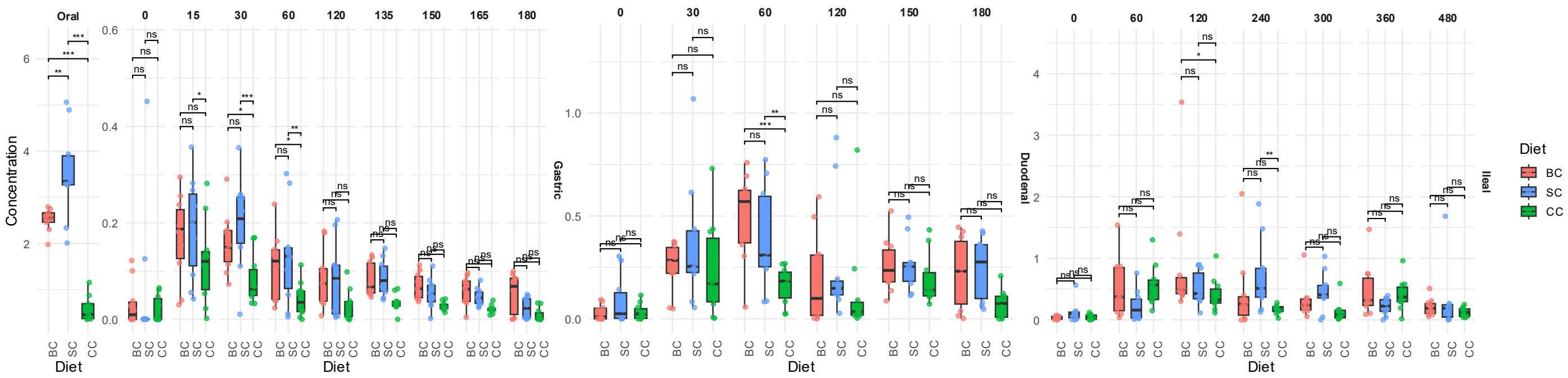


## (bi) sucrose

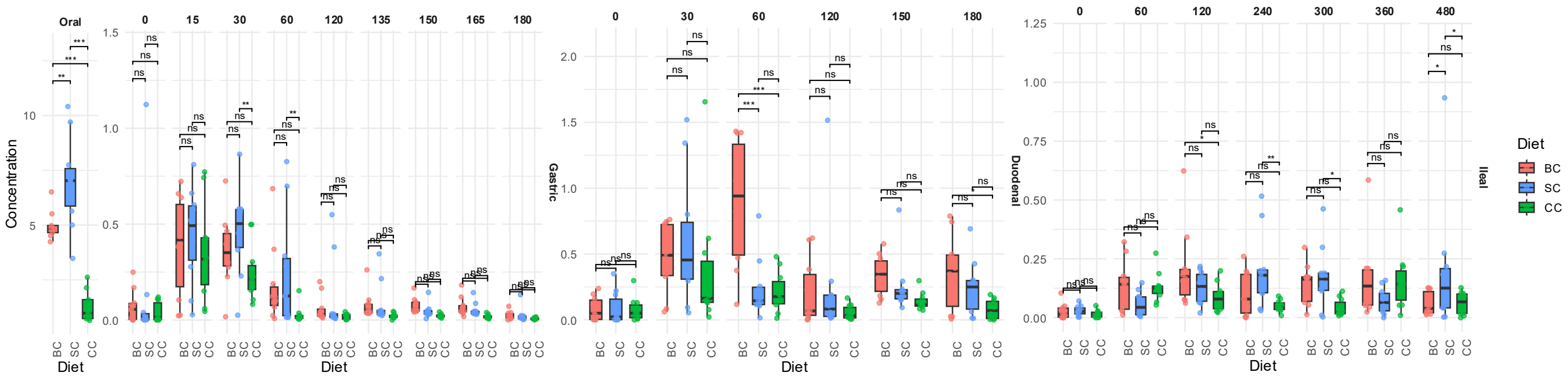


## (ci) metabolites with only oral samples

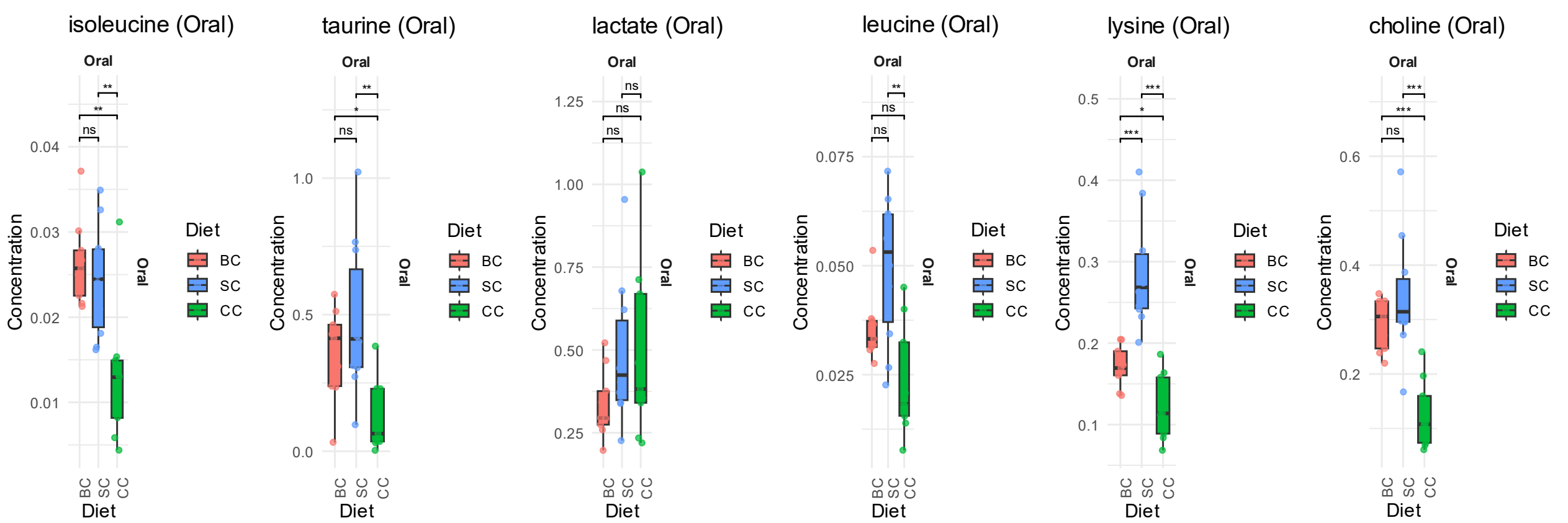

**Supplementary Figure 8: Observed peak-timepoint of maximum metabolite concentration.** Each point represents one participant's observed peak sampling time for the indicated metabolite, diet and gastrointestinal location. Boxes show medians and interquartile ranges; whiskers extend to the most extreme value within 1.5 × the interquartile range, with all observations shown individually. Diet effects were tested separately within each location using linear mixed-effects models (observed_peak_time ~ diet + (1 | participant)), followed by pairwise estimated-marginal-mean contrasts with Holm correction. $*P \leq 0.05$, $**P \leq 0.01$ and $***P \leq 0.001$; ns, not significant.

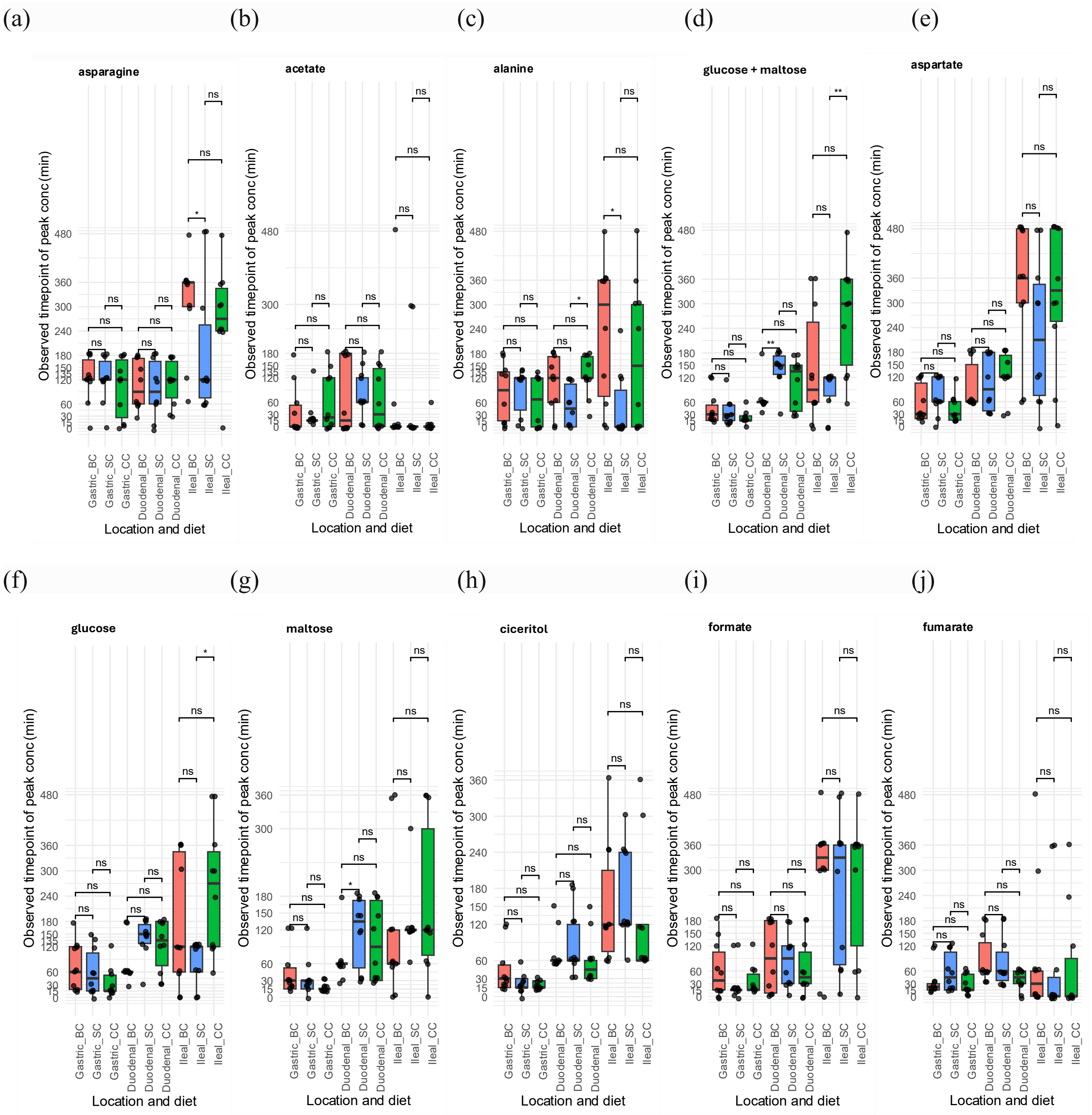

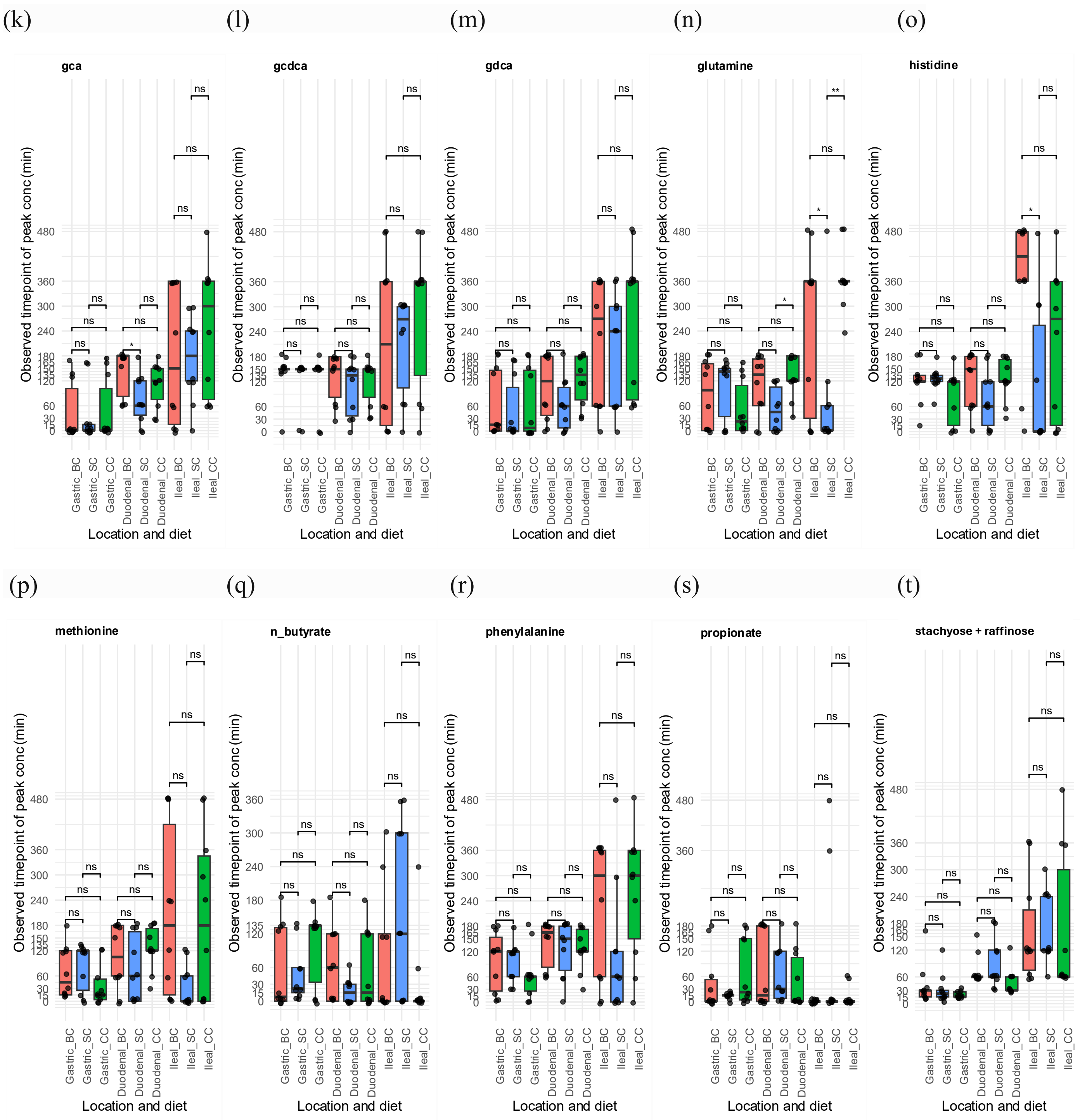

(k)
gca
(l)
gcdca
(m)
gdca
(n)
glutamine
(o)
histidine
(p)
methionine
(q)
n_butyrate
(r)
phenylalanine
(s)
propionate
(t)
stachyose + raffinose
Observed timepoint of peak conc (min)
Location and diet
Gastric_BC
Gastric_SC
Gastric_CC
Duodenal_BC
Duodenal_SC
Duodenal_CC
Ileal_BC
Ileal_SC
Ileal_CC
ns
*
**

(u)

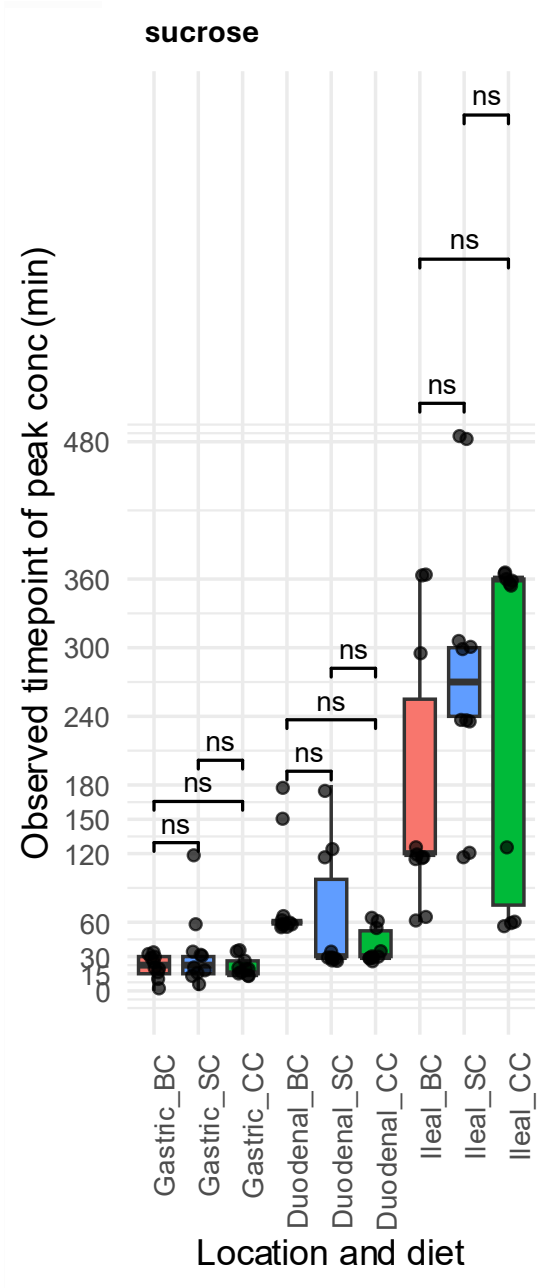
sucrose
Observed timepoint of peak conc (min)
Gastric_BC Gastric_SC Gastric_CC Duodenal_BC Duodenal_SC Duodenal_CC Ileal_BC Ileal_SC Ileal_CC
Location and diet

(v)

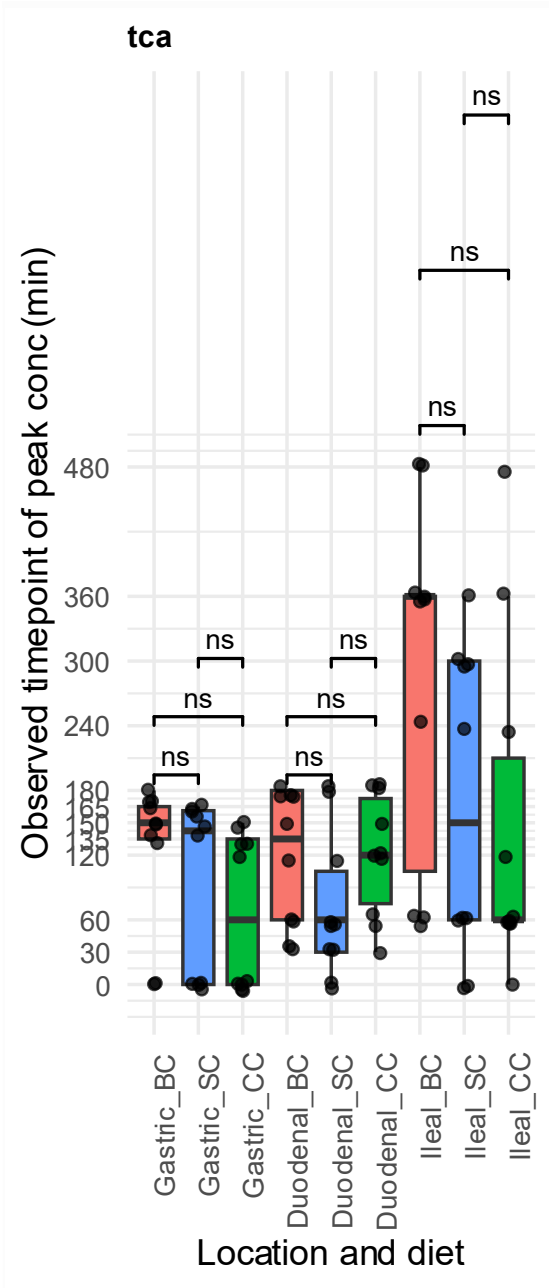
tca
Observed timepoint of peak conc (min)
Gastric_BC Gastric_SC Gastric_CC Duodenal_BC Duodenal_SC Duodenal_CC Ileal_BC Ileal_SC Ileal_CC
Location and diet

(w)

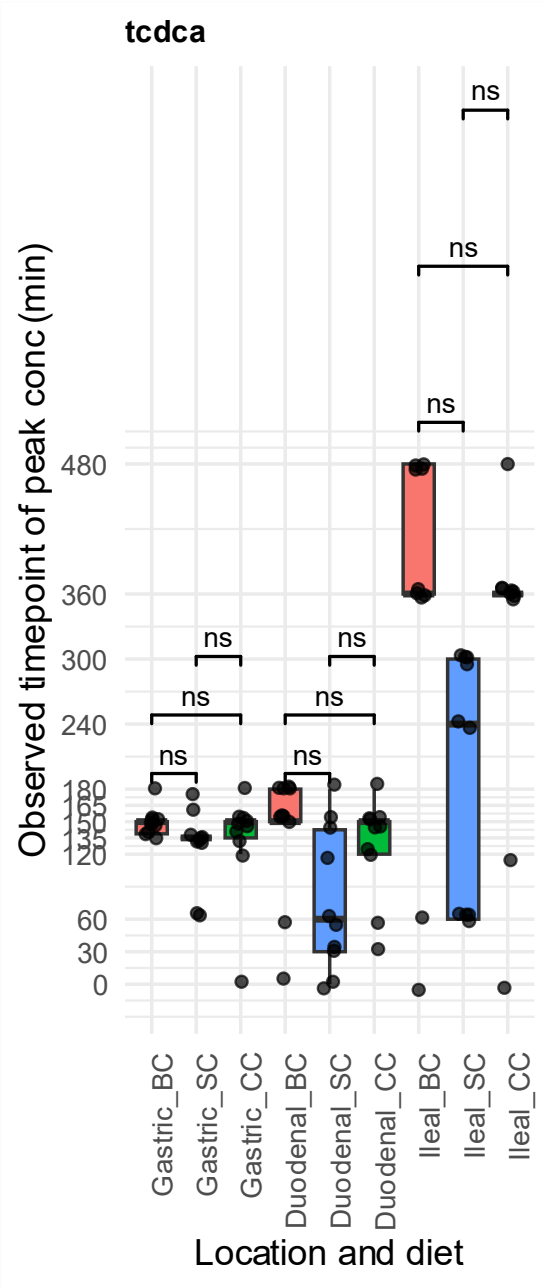
tcdca
Observed timepoint of peak conc (min)
Gastric_BC Gastric_SC Gastric_CC Duodenal_BC Duodenal_SC Duodenal_CC Ileal_BC Ileal_SC Ileal_CC
Location and diet

(x)

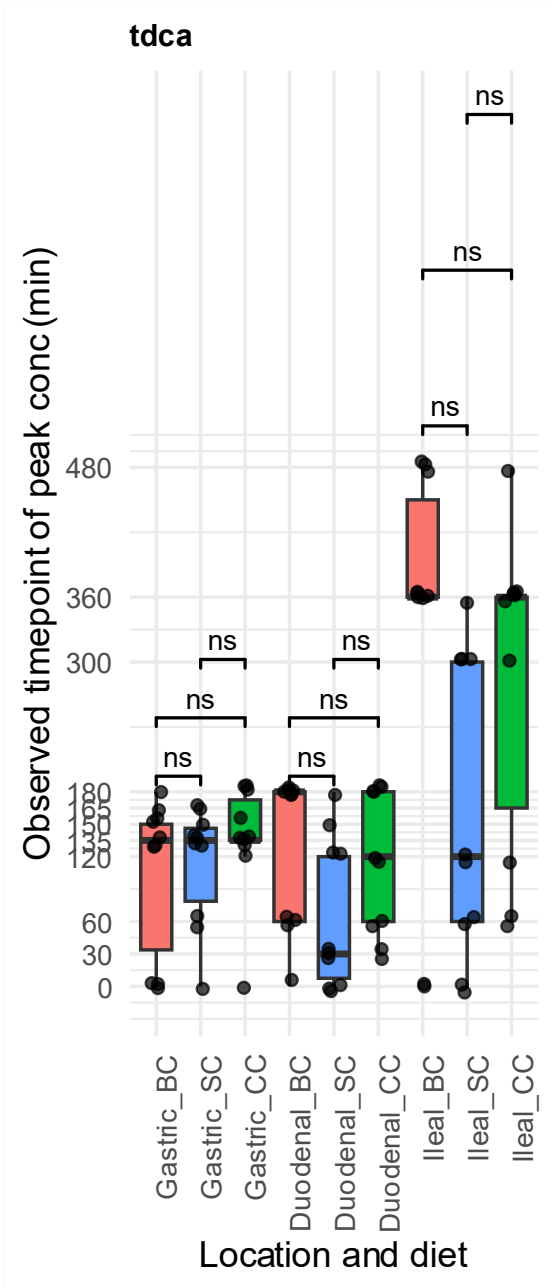
tdca
Observed timepoint of peak conc (min)
Gastric_BC Gastric_SC Gastric_CC Duodenal_BC Duodenal_SC Duodenal_CC Ileal_BC Ileal_SC Ileal_CC
Location and diet

(y)

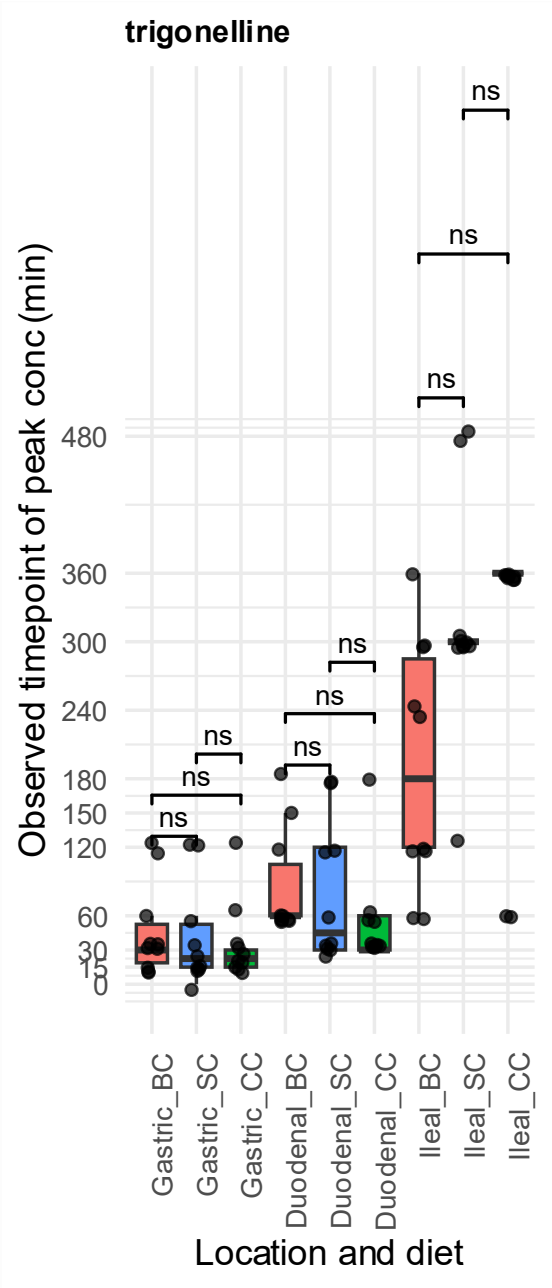
trigonelline
Observed timepoint of peak conc (min)
Gastric_BC Gastric_SC Gastric_CC Duodenal_BC Duodenal_SC Duodenal_CC Ileal_BC Ileal_SC Ileal_CC
Location and diet

(z)

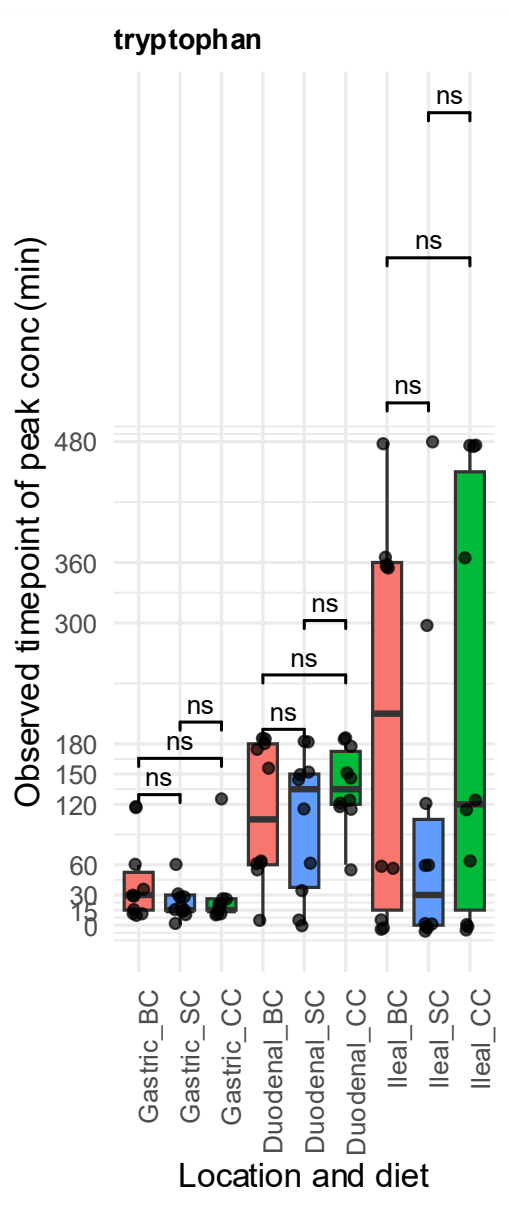
tryptophan
Observed timepoint of peak conc (min)
Gastric_BC Gastric_SC Gastric_CC Duodenal_BC Duodenal_SC Duodenal_CC Ileal_BC Ileal_SC Ileal_CC
Location and diet

(ai)

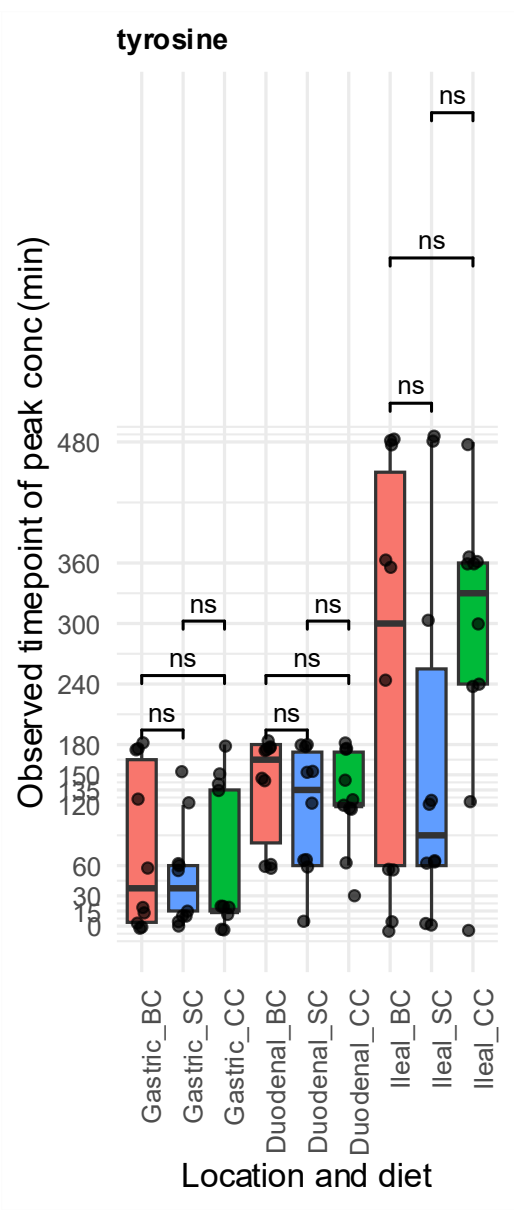
tyrosine
Observed timepoint of peak conc (min)
Gastric_BC Gastric_SC Gastric_CC Duodenal_BC Duodenal_SC Duodenal_CC Ileal_BC Ileal_SC Ileal_CC
Location and diet

(bi)

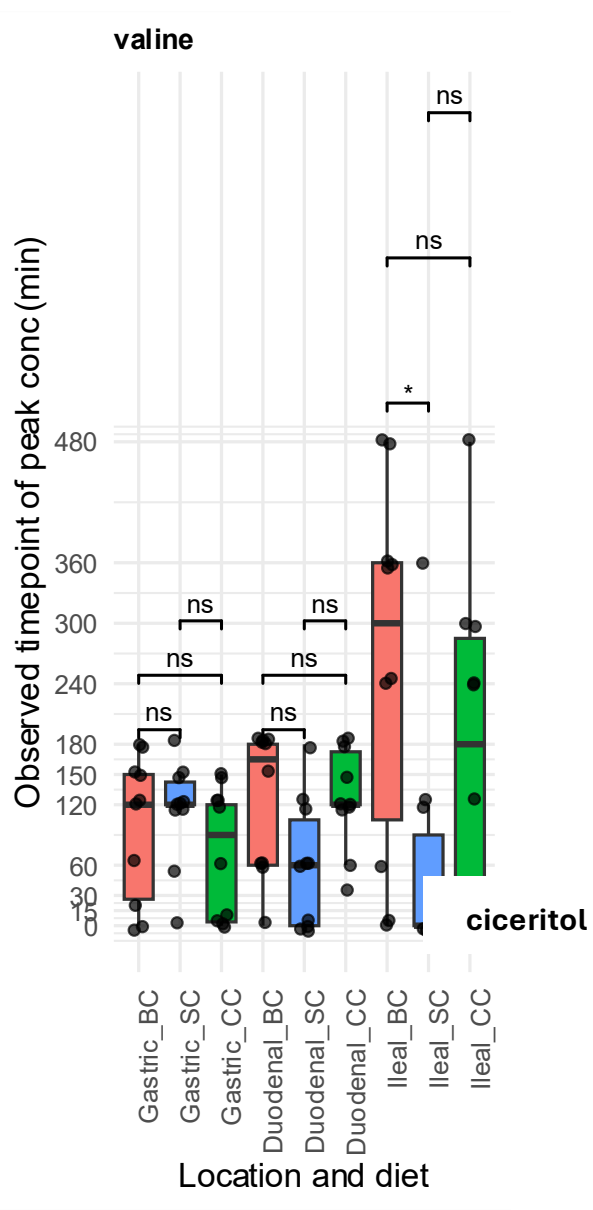
valine
Observed timepoint of peak conc (min)
ciceritol
Gastric_BC Gastric_SC Gastric_CC Duodenal_BC Duodenal_SC Duodenal_CC Ileal_BC Ileal_SC Ileal_CC
Location and diet

**Supplementary Figure 9**. **Breath hydrogen levels as marker of intestinal fermentation.** Time-course changes in breath hydrogen (ppm) following the consumption of chickpea meals (blue: Intact single cells (SC), green: cell clusters (CC), red: broken cells (BC)). Data are presented as mean ± SEM. Statistical differences between groups were analyzed using repeated measure two-way ANOVA followed by Tukey's post-hoc test ($p < 0.05$). Different letters (a, b, c) indicate significant differences between groups at the same time point: a – SC vs CC; b- SC vs BC; c-SC vs CC.

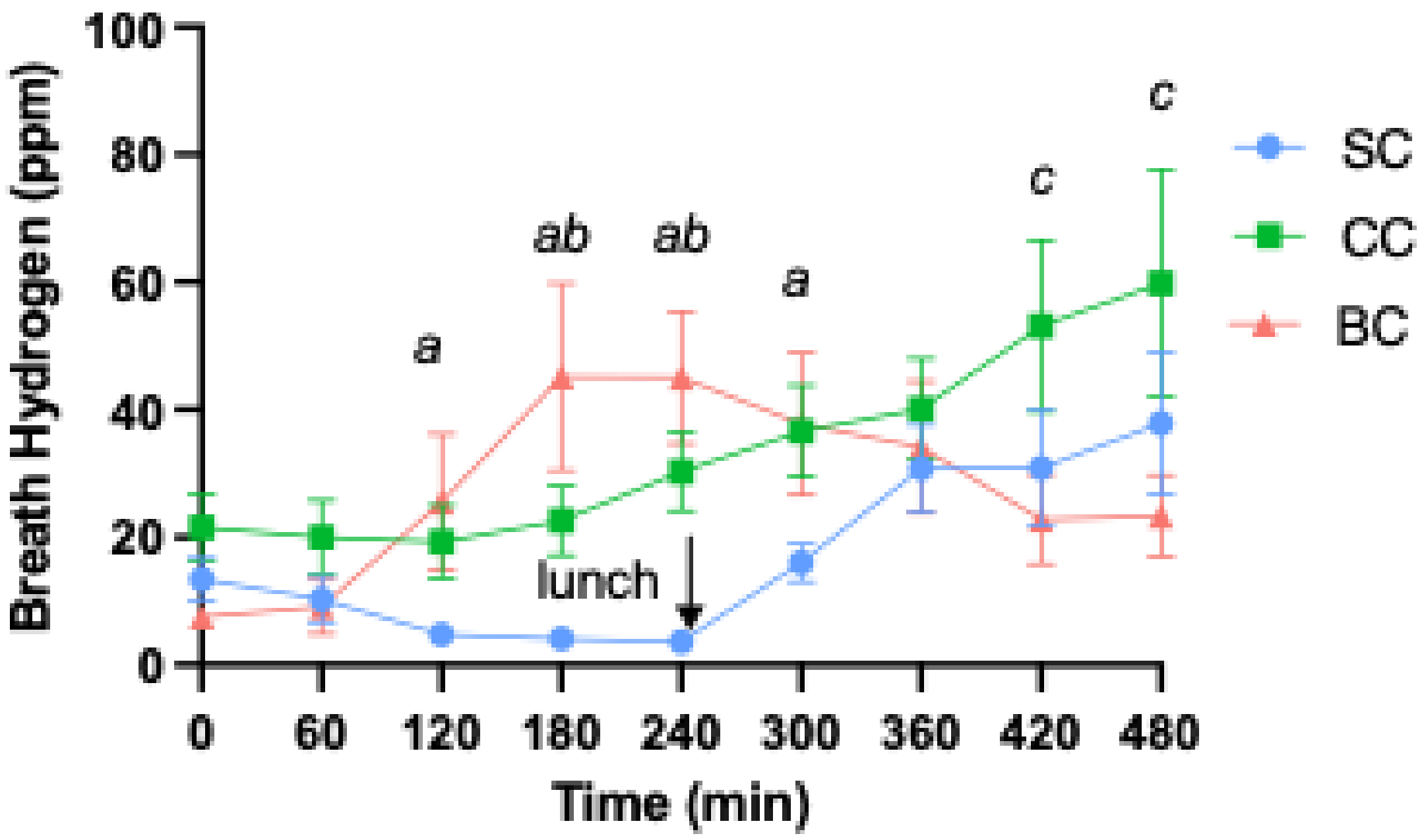

**Supplementary Figure 10. Composition of the ileal microbiome. (a)** Baseline family-level taxonomic composition of the ileal microbiome across participants and meal types. Stacked bar plots showing relative abundance of bacterial families in ileal aspirates collected at baseline (time 0, pre-meal) for each participant, faceted by meal type: single cell (SC), cell clusters (CC) and broken cells (BC). Taxonomy was assigned at family level, with the top 15 families by mean relative abundance shown individually and remaining taxa collapsed into 'Other'. The ileal microbiome at baseline was dominated by Pasteurellaceae and Streptococcaceae, with substantial inter-individual variation in community structure. Not all participants provided baseline samples for every meal due to the crossover design (SC, n = 5; CC, n = 7; BC, n = 5). Relative abundances were calculated from OTU counts normalised to proportions within each sample. **(b)** Temporal dynamics of ileal microbiome family-level composition following three meal types. Stacked bar plots showing mean relative abundance of bacterial families in ileal aspirates sampled at nine time points (0, 60, 120, 180, 240, 300, 360, 420 and 480 min post-meal), averaged across participants for each meal type: single cell (SC), cell clusters (CC) and broken cells (BC). The top 15 families by overall mean relative abundance are shown individually; remaining taxa are collapsed into 'Other'. Pasteurellaceae and Streptococcaceae remained the dominant families across all meals and time points. Enterobacteriaceae showed a notable increase in relative abundance at later time points, particularly in the SC and CC meals, whereas the BC meal exhibited a distinct compositional profile characterised by higher Lachnospiraceae and lower Pasteurellaceae abundance at baseline that shifted over the postprandial period. Sample sizes varied across time points due to sample availability (n = 5–8 per time point per meal).

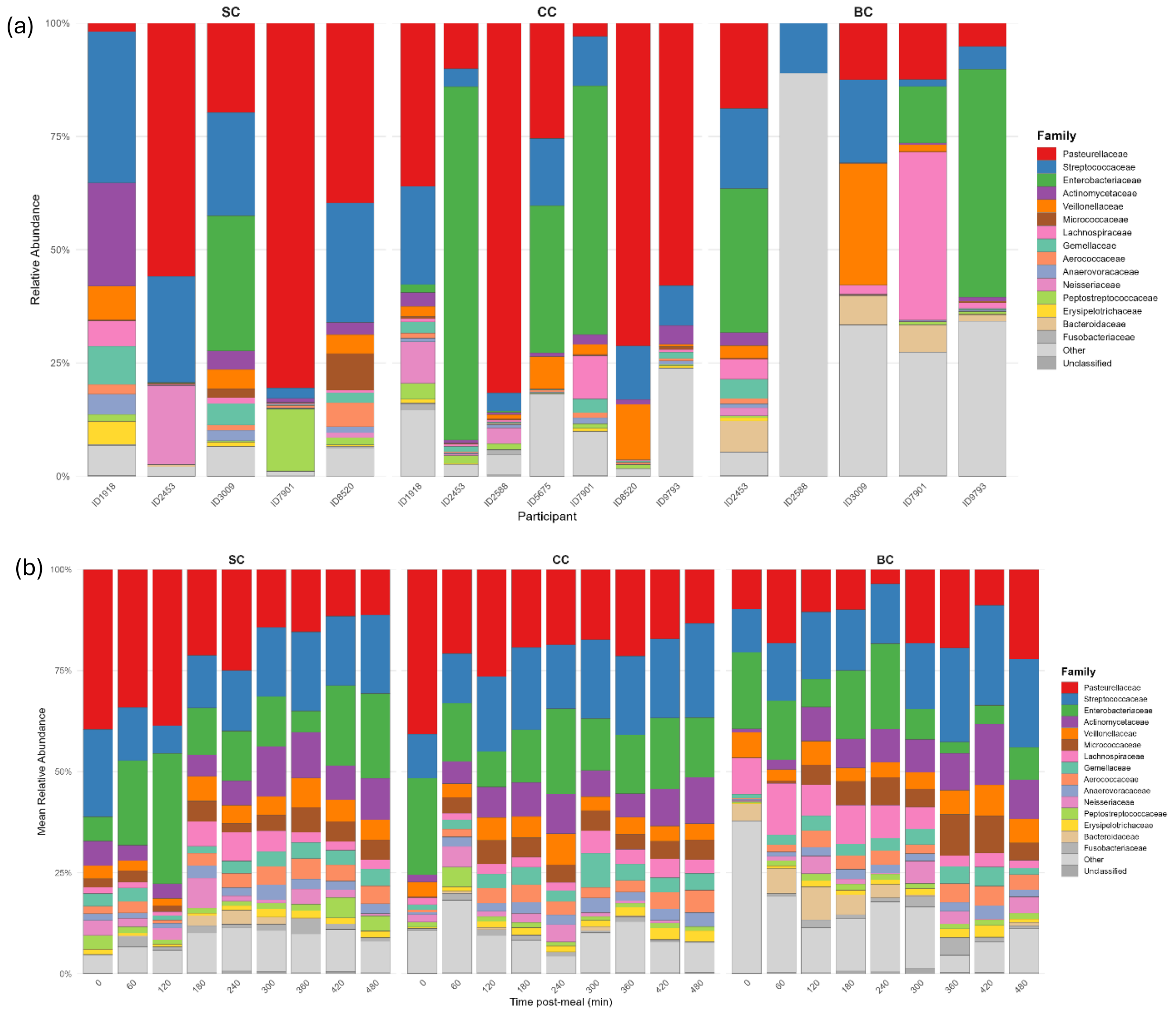

**Supplementary Figure 11: Sequencing read composition varies with meal arrival, not with catheter dwell time.** Mean proportion of sequencing reads classified as chickpea-derived (mapped to Cicer arietinum), human (mapped to the human reference genome), or "other" (predominantly bacterial) in ileal samples at each timepoint, shown per meal (BC, broken cells; SC, single cells; CC, cell clusters). Read mates were averaged per biological sample and the three fractions renormalised to sum to 100%.

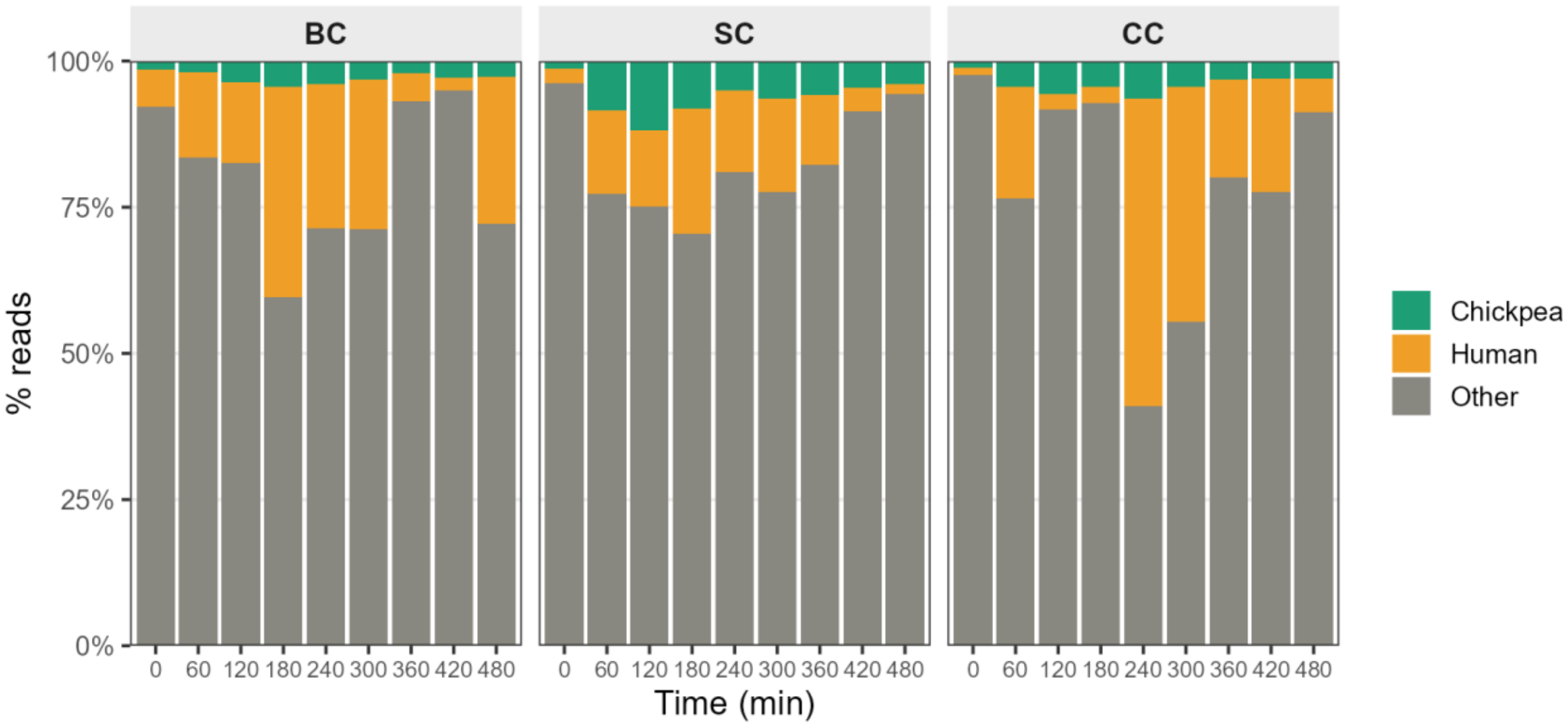


**Supplementary Figure 12: Correlations between gut hormones (GLP1 and PYY) and metagenome abundances.** Associations are determined by linear modelling, with standard error shown by the error bars. Each point is coloured by the proportion of strain sharing, with red indicating that strain sharing has not been identified between the oral and ileal compartments, while blue indicates that in all instances strains are shared between oral and ileal compartments.

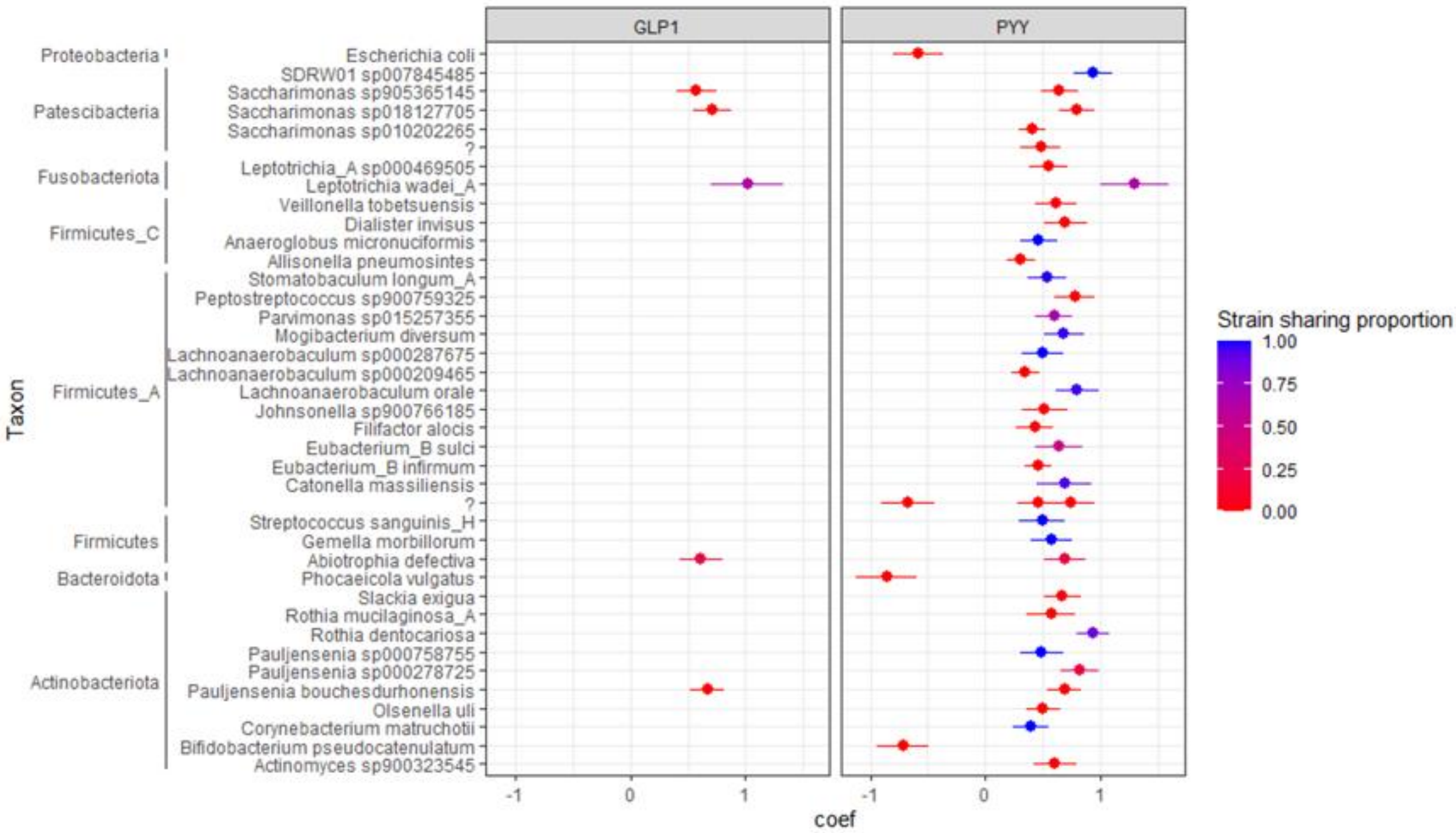

**Supplementary Figure 13. The fasted ileal community does not drift with visit order.** Principal coordinates analysis (PCoA) of Bray–Curtis dissimilarities among fasted (0 min) ileal samples from the eight participants, coloured by the order in which each participant's three Phase 2 visits occurred (Visit 1–3); ellipses denote 68% (1 s.d.) normal data ellipses per visit. Each Phase 2 visit comprised a single chickpea meal, separated by a minimum 7-day washout. Visit order explained a small, non-significant fraction of baseline compositional variation (PERMANOVA restricted within participant, $R^2 = 0.176$, $F_{2,13} = 1.39$, $P = 0.20$.

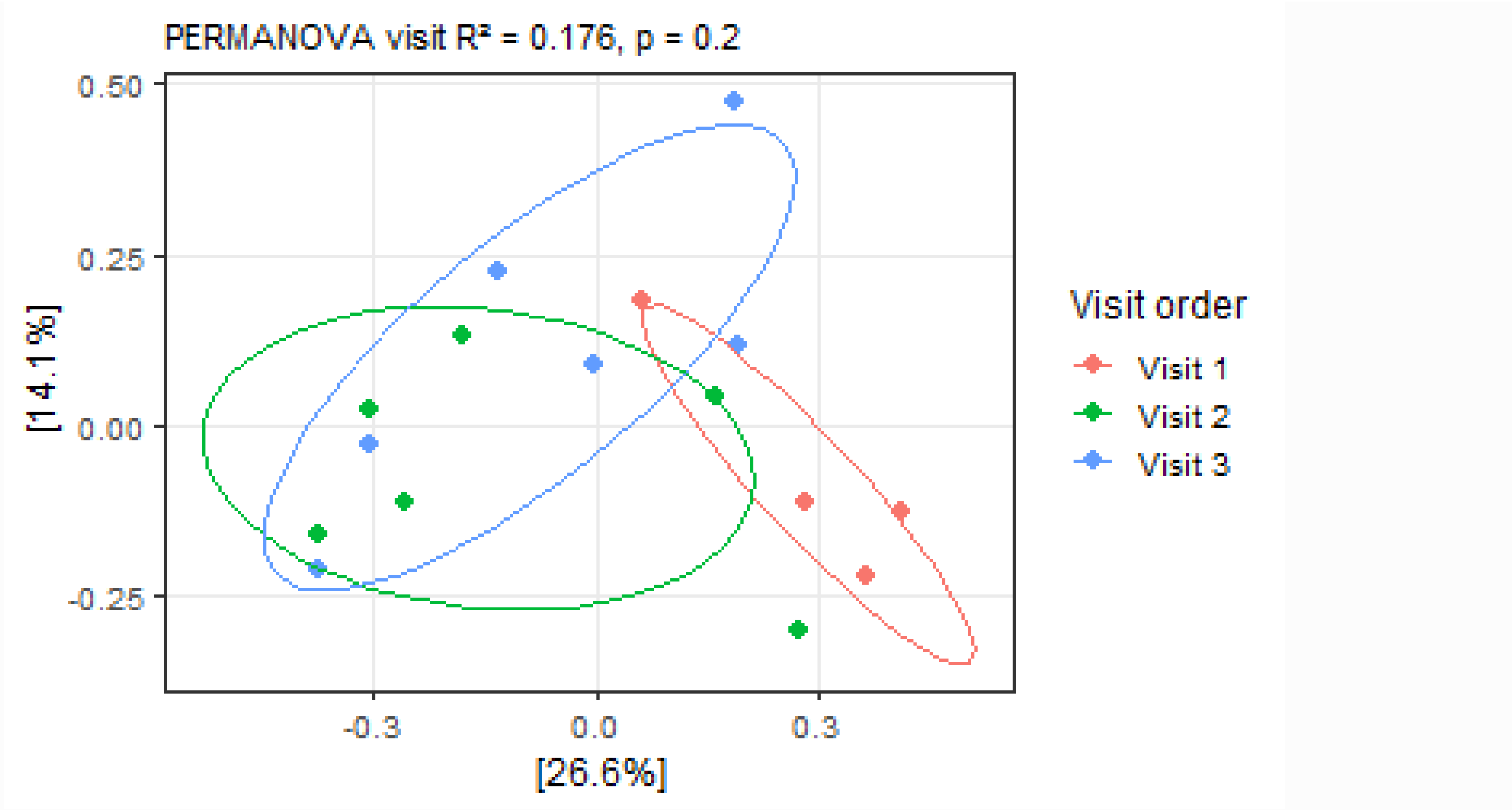

**Supplementary Figure 14: Exploratory taxon and gut-hormone associations with ileal metabolites by diet.** Panels show BC (0, left), SC (1, middle) and CC (2, right). Tile colour represents the standardized association estimate from separate observed-variable SEM regressions fitted using robust maximum likelihood (red, positive; blue, negative). Taxa were retained if detected (abundance > 0) in at least 30% of linked microbiome samples and if their mean log1p-transformed abundance exceeded 1. Taxon-metabolite associations are shown for two-sided BH-adjusted $P < 0.05$ and $|\beta| > 0.5$; hormone-metabolite associations are shown for two-sided BH-adjusted $P < 0.05$. Participants were the biological units, technical replicates were not treated as independent observations. Peak timing varied among participants according to bolus transit, meaning that absolute sampling times did not necessarily represent equivalent physiological stages. These findings are therefore exploratory. A sensitivity analysis using participant random intercepts and absolute timepoint as a fixed effect yielded no associations after BH correction, although this model did not account for participant-specific temporal alignment of peaks.

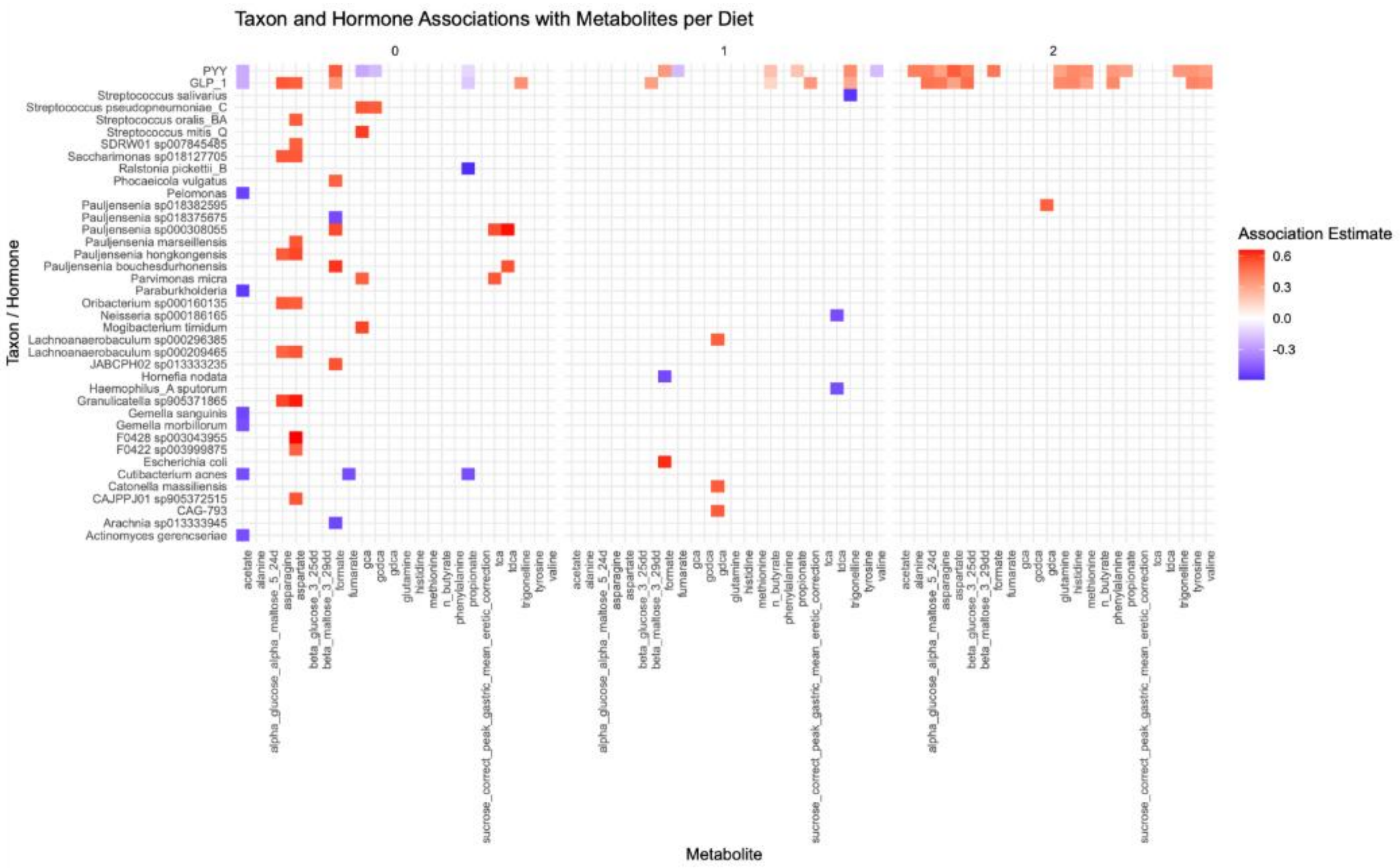

Supplement: Supplementary file 1 [file 41467_2026_77714_MOESM1_ESM.pdf]
