# Supplementary material for: Effect of dietary chickpea structures on the small intestine metabolome and microbiome: a randomised crossover trial
Source: Nat Commun. 2026 Sep 15;17:10840. doi: 10.1038/s41467-026-77714-7 (PMC13672355; doi:10.1038/s41467-026-77714-7)
Supplement: Supplementary file 2 [file 41467_2026_77714_MOESM2_ESM.pdf]

nature portfolio

Corresponding author(s): Gary Frost

Last updated by author(s): 17/Aug/2026

Reporting Summary

Nature Portfolio wishes to improve the reproducibility of the work that we publish. This form provides structure for consistency and transparency in reporting. For further information on Nature Portfolio policies, see our Editorial Policies and the Editorial Policy Checklist.

Statistics

For all statistical analyses, confirm that the following items are present in the figure legend, table legend, main text, or Methods section.

n/a	Confirmed	
☐	☒	The exact sample size (n) for each experimental group/condition, given as a discrete number and unit of measurement
☐	☒	A statement on whether measurements were taken from distinct samples or whether the same sample was measured repeatedly
☐	☒	The statistical test(s) used AND whether they are one- or two-sided<br>*Only common tests should be described solely by name; describe more complex techniques in the Methods section.*
☐	☒	A description of all covariates tested
☐	☒	A description of any assumptions or corrections, such as tests of normality and adjustment for multiple comparisons
☐	☒	A full description of the statistical parameters including central tendency (e.g. means) or other basic estimates (e.g. regression coefficient) AND variation (e.g. standard deviation) or associated estimates of uncertainty (e.g. confidence intervals)
☐	☒	For null hypothesis testing, the test statistic (e.g. F, t, r) with confidence intervals, effect sizes, degrees of freedom and P value noted<br>*Give P values as exact values whenever suitable.*
☒	☐	For Bayesian analysis, information on the choice of priors and Markov chain Monte Carlo settings
☒	☐	For hierarchical and complex designs, identification of the appropriate level for tests and full reporting of outcomes
☒	☐	Estimates of effect sizes (e.g. Cohen's d, Pearson's r), indicating how they were calculated

Our web collection on statistics for biologists contains articles on many of the points above.

Software and code

Policy information about availability of computer code

Data collection	no software used for data collection
Data analysis	Metabolomic data were processed in MATLAB R2021a (MathWorks) using in-house scripts for spectral processing and multivariate analysis. Metabolite quantification was performed using custom JavaScript and R scripts following Bruker IVDr procedures, with model fitting based on a Levenberg–Marquardt optimisation algorithm. Imputation, principal component analysis, dynamic time warping, and visualisation were performed in R.<br><br>Metabolomic data were imputed separately for each metabolite in R using missForest, with diet, participant identity, gastrointestinal location and sampling time as predictors. Principal component analysis was performed using stats::prcomp, with variables centered and scaled, and visualised using ggfortify and ggplot2. Structural equation/path models were fitted using lavaan, while metabolite concentration data were analysed using linear mixed-effects models implemented in lme4, with pairwise estimated-marginal-mean contrasts calculated using emmeans. Two-dimensional figures were generated using ggplot2, ggpubr, ComplexHeatmap, circlize and patchwork. Data manipulation and model-output processing used dplyr, tidyr, readr, purrr, janitor and broom. Data matrices were exported from R, and three-dimensional visualisations were generated using OriginPro 2025.<br><br>Metagenomic data were analysed using the MG-TK pipeline (formerly MATAFILER), incorporating the following open-source tools: sdm, Kraken2, MEGAHIT, SemiBin, Bowtie2, Prodigal, bcftools, GTDB-Tk, IQ-TREE, and SPIEC-EASI. Additional custom code was used for strain-sharing analysis, multi-omics integration, and figure generation.

For manuscripts utilizing custom algorithms or software that are central to the research but not yet described in published literature, software must be made available to editors and reviewers. We strongly encourage code deposition in a community repository (e.g. GitHub). See the Nature Portfolio guidelines for submitting code & software for further information.

Data

Policy information about availability of data

All manuscripts must include a data availability statement. This statement should provide the following information, where applicable:

- Accession codes, unique identifiers, or web links for publicly available datasets
- A description of any restrictions on data availability
- For clinical datasets or third party data, please ensure that the statement adheres to our policy

The enteroendocrine hormone and metabolomic data generated in this study have been deposited in Zenodo at: 10.5281/zenodo.20679139. Metagenomic sequencing data is available through the NCBI SRA through project code PRJNA1240928.

Custom codes used in this study are available from https://github.com/Nerdobyte/Diet_Metabolome_Microbiome. The code has been deposited in Code Ocean: Provisional DOI is 10.24433/CO.3003445.v1

Research involving human participants, their data, or biological material

Policy information about studies with human participants or human data. See also policy information about sex, gender (identity/presentation), and sexual orientation and race, ethnicity and racism.

Reporting on sex and gender	Sex/gender was recorded by participant self-report during screening. The gender of participants was determined based on self-report. Sex/gender was considered during recruitment to include both female and male participants. The study was not designed or powered to detect sex- or gender-specific effects, and therefore no post hoc sex- or gender-stratified analyses were performed.<br><br>Six male and four female completed phase 1; four female and four male completed phase 2; five female and five male completed phase 3.
Reporting on race, ethnicity, or other socially relevant groupings	No socially relevant categorization was made in the section of study participants.
Population characteristics	We admitted 6 male and 4 female adult participants aged (mean ± SEM) 30.8 y ± 2.4 years with BMI 24.9 ± 0.8 kg/m2. Participants with an abnormal ECG, screening blood values outside the clinical reference range, a history of cancer, diabetes, gastrointestinal disease and/or requiring medication likely to interfere with metabolic and hormone responses were excluded.
Recruitment	All participants were recruited from the healthy volunteer database of NIHR Imperial Clinical Research Facility. Individuals who expressed interest were invited to complete a pre-screening form and attend an in-person screening visit, during which eligibility was assessed by a medical doctor based on health history, anthropometric measurements, ECGs, and blood test results. This recruitment approach may introduce self-selection bias, as individuals who are more health-conscious or motivated to participate in research may be overrepresented. However, given that this was an acute study conducted in a highly controlled clinical environment, with standardized meals, procedures, and close monitoring, the influence of self-selection bias on the the results is likely to be minimal.
Ethics oversight	This study was approved by the Health Research Authority and London-Camden and King's Cross Research Ethics Committee (REC 19/LO/0962) before the commencement of any study procedures.

Note that full information on the approval of the study protocol must also be provided in the manuscript.

Field-specific reporting

Please select the one below that is the best fit for your research. If you are not sure, read the appropriate sections before making your selection.

☒ Life sciences ☐ Behavioural & social sciences ☐ Ecological, evolutionary & environmental sciences

For a reference copy of the document with all sections, see nature.com/documents/nr-reporting-summary-flat.pdf

Life sciences study design

All studies must disclose on these points even when the disclosure is negative.

Sample size	As no prior studies had investigated the spatiotemporal metabolome and microbiome of the human gut in regard to food structure, sample size calculation was not feasible. Using similar intubation methods, Cai et al.(https://www.nature.com/articles/s42255-025-01309-7) and Dagbasi et al.(https://www.science.org/doi/10.1126/scitranslmed.adm8132) investigated duodenal and ileal metabolite profiles, respectively, observed significant differences between dietary interventions with ten subjects. Thus, ten participants were recruited for this study.
Data exclusions	No data was excluded from the analysis
Replication	Experiments were conducted in duplicate. All attempts at replication were successful.

Randomization	Participants were randomly allocated to the sequence of dietary interventions in this crossover study. Randomization was performed using a sealed-envelope system (Sealed Envelope Ltd., 2022) by an independent researcher who was not involved in the trial. Each participant received all dietary interventions, so between-participant covariates were controlled by the within-participant crossover design.
Blinding	This study followed a double-blinded, randomized crossover design.

Reporting for specific materials, systems and methods

We require information from authors about some types of materials, experimental systems and methods used in many studies. Here, indicate whether each material, system or method listed is relevant to your study. If you are not sure if a list item applies to your research, read the appropriate section before selecting a response.

Materials & experimental systems

n/a	Involved in the study
☐	☒ Antibodies
☒	☐ Eukaryotic cell lines
☒	☐ Palaeontology and archaeology
☒	☐ Animals and other organisms
☐	☒ Clinical data
☒	☐ Dual use research of concern
☒	☐ Plants

Methods

n/a	Involved in the study
☒	☐ ChIP-seq
☒	☐ Flow cytometry
☒	☐ MRI-based neuroimaging

Antibodies

Antibodies used	Plasma GIP concentrations were quantified using a commercial human GIP ELISA kit (EZHGIP-54K; Merck, UK) according to the manufacturer's instructions. Kit antibodies and reagents were used at the concentrations/dilutions supplied by the manufacturer. GLP-1 and PYY concentrations were measured using validated in-house radioimmunoassays. Synthetic GLP-1 and PYY peptide standards were obtained from Bachem: GLP-1 (CAS 106612-94-6; Bachem product no. 4034865) and PYY (CAS 123583-37-9; Bachem product no. 4018880).
Validation	GLP-1:https://shop.bachem.com/product/4034865/<br>PYY: https://shop.bachem.com/product/4018880/

Clinical data

Policy information about clinical studies

All manuscripts should comply with the ICMJE guidelines for publication of clinical research and a completed CONSORT checklist must be included with all submissions.

Clinical trial registration	Trial registration: ISRCTN18097249
Study protocol	Study protocol has been submitted with the manuscript
Data collection	Location: Imperial College London; Study Start Date: February 2020; Study End Date: June 2024
Outcomes	The primary outcome was the postprandial gut hormone responses. Co-secondary outcomes included spatiotemporal profiling of luminal metabolites across the gastrointestinal tract and ileal microbiome. Intestinal metabolomics was performed using proton nuclear magnetic resonance (1H NMR) spectroscopy. Plasma GIP concentrations were quantified using a commercial ELISA (EZHGIP-54K, Merck, UK), while GLP-1 and PYY were measured using validated in-house radioimmunoassays (RIA). Microbiota composition was characterised using shotgun metagenomic sequencing, with taxonomic profiling, metagenome-assembled genome (MAG) reconstruction, and strain-level analysis performed using established bioinformatic pipelines.

Plants

Seed stocks	*Report on the source of all seed stocks or other plant material used. If applicable, state the seed stock centre and catalogue number. If plant specimens were collected from the field, describe the collection location, date and sampling procedures.*
Novel plant genotypes	*Describe the methods by which all novel plant genotypes were produced. This includes those generated by transgenic approaches, gene editing, chemical/radiation-based mutagenesis and hybridization. For transgenic lines, describe the transformation method, the number of independent lines analyzed and the generation upon which experiments were performed. For gene-edited lines, describe the editor used, the endogenous sequence targeted for editing, the targeting guide RNA sequence (if applicable) and how the editor was applied.*
Authentication	*Describe any authentication procedures for each seed stock used or novel genotype generated. Describe any experiments used to assess the effect of a mutation and, where applicable, how potential secondary effects (e.g. second site T-DNA insertions, mosiacism, off-target gene editing) were examined.*
