# Supplementary material for: Effect of dietary chickpea structures on the small intestine metabolome and microbiome: a randomised crossover trial
Source: Nat Commun. 2026 Sep 15;17:10840. doi: 10.1038/s41467-026-77714-7 (PMC13672355; doi:10.1038/s41467-026-77714-7)
Supplement: Supplementary file 3 [file 41467_2026_77714_MOESM3_ESM.pdf]

Description of Additional Supplementary Files

File Name: Supplementary Data 1

Description: Taxonomic details of the oral-ileal shared strains.
