# Supplementary material for: Effect of dietary chickpea structures on the small intestine metabolome and microbiome: a randomised crossover trial
Source: Nat Commun. 2026 Sep 15;17:10840. doi: 10.1038/s41467-026-77714-7 (PMC13672355; doi:10.1038/s41467-026-77714-7)
Supplement: Supplementary file 4 [file 41467_2026_77714_MOESM4_ESM.pdf]

nature portfolio

Peer Review File

Effect of dietary chickpea structures on the gut metabolome and microbiome: A randomised cross over trial

Corresponding Author: Professor Gary Frost

This manuscript has been previously reviewed at another journal. This document only contains information relating to versions considered at Nature Communications.

This file contains all reviewer reports in order by version, followed by all author rebuttals in order by version.

Version 0:

Reviewer comments:

Reviewer #2

(Remarks to the Author)
The manuscript has improved, and the major concerns have been addressed. However, the Discussion would still benefit from a more substantive consideration of key studies examining small-intestinal microbiota composition and function in health and disease. We raised this point previously, but the response did not adequately address how this literature may contextualize the present findings. To facilitate revision, we now provide the relevant references explicitly: PMID: 35810781; PMID: 37315761; and PMID: 41917007.
Although much of the available evidence derives from disease-specific cohorts and their controls, these studies provide important context for interpreting the present findings. They should be discussed where they support, extend, or contrast with the results reported here. Doing so would strengthen the interpretation and increase the impact of the current study.

We also appreciate the challenges of recruiting healthy volunteers and acknowledge the authors' explanation that the study was powered according to its primary outcomes. Nevertheless, the microbiome and metabolome analyses are central components of the manuscript. The limitations associated with the sample size for these datasets should therefore be acknowledged more explicitly, including the potential implications for statistical robustness, reproducibility, and generalizability. A clearer discussion of these considerations would help readers appropriately interpret the strength and scope of the reported findings.

Elena Verdu

Open Access This Peer Review File is licensed under a Creative Commons Attribution 4.0 International License, which permits use, sharing, adaptation, distribution and reproduction in any medium or format, as long as you give appropriate credit to the original author(s) and the source, provide a link to the Creative Commons license, and indicate if changes were made.
In cases where reviewers are anonymous, credit should be given to 'Anonymous Referee' and the source.
The images or other third party material in this Peer Review File are included in the article's Creative Commons license, unless indicated otherwise in a credit line to the material. If material is not included in the article's Creative Commons license and your intended use is not permitted by statutory regulation or exceeds the permitted use, you will need to obtain permission directly from the copyright holder.
To view a copy of this license, visit https://creativecommons.org/licenses/by/4.0/

REVIEWERS' COMMENTS

Reviewer #2 (Remarks to the Author):

The manuscript has improved, and the major concerns have been addressed. However, the Discussion would still benefit from a more substantive consideration of key studies examining small-intestinal microbiota composition and function in health and disease. We raised this point previously, but the response did not adequately address how this literature may contextualize the present findings. To facilitate revision, we now provide the relevant references explicitly: PMID: 35810781; PMID: 37315761; and PMID: 41917007.

Although much of the available evidence derives from disease-specific cohorts and their controls, these studies provide important context for interpreting the present findings. They should be discussed where they support, extend, or contrast with the results reported here. Doing so would strengthen the interpretation and increase the impact of the current study.

We also appreciate the challenges of recruiting healthy volunteers and acknowledge the authors' explanation that the study was powered according to its primary outcomes. Nevertheless, the microbiome and metabolome analyses are central components of the manuscript. The limitations associated with the sample size for these datasets should therefore be acknowledged more explicitly, including the potential implications for statistical robustness, reproducibility, and generalizability. A clearer discussion of these considerations would help readers appropriately interpret the strength and scope of the reported findings.

Elena Verdu

Response: We thank Reviewer #2 for this comment. We agree that the Discussion should better contextualise our findings within recent studies of small-intestinal microbiota composition and function in health and disease. We have therefore expanded the Discussion to include the studies highlighted by the reviewer together with additional relevant literature. We added in Discussion section line 582-592: Our findings can be contrasted against the small number of recent studies which have characterised the small intestinal (primarily duodenal) microbiome in disease states [33]. Recent studies have demonstrated that across a range of conditions including obesity, SIBO and irritable bowel syndrome the duodenal microbiota is relatively stable under fasted conditions, but rapidly increases in species richness following addition of a nutrient bolus [34] , closely matching our findings that microbial diversity rises following ingestion of a meal, as predicted by metabolic modelling of small intestinal microbiome

dynamics [35]. Ectopic colonisation of the small intestine by oral bacterial species has been causally linked to mucosal inflammation in susceptible hosts, and overgrowth of oral associated bacteria in the small intestine has been described in environmental enteric dysfunction [36,37] and coeliac disease, where there is evidence for altered fibre fermentation in small intestine [38].

We have also expanded the limitations section to acknowledge more explicitly that the metabolome and microbiome analyses were performed in a small cohort. We have added in line 642-645 : Lastly, although the repeated-measures crossover design provided high temporal resolution within participants, the metabolome and microbiome analyses were performed in a relatively small cohort. Further validation in larger cohorts will be required.
